# Supplementary material for: RNA sequencing-based exploration of the effects of far-red light on microRNAs involved in the shade-avoidance response of D. officinale
Source: PeerJ. 2023 Mar 20;11:e15001. doi: 10.7717/peerj.15001 (PMC10035421; doi:10.7717/peerj.15001)
Supplement: Table S1 [file peerj-11-15001-s001.pdf]

Table S1 The power analysis in *D. officinale* under different light treatments

miRNAs	CK1 TPM	CK2 TPM	CK3 TPM	FR2-1 TPM	FR2-2 TPM	FR2-3 TPM	FR8-1 TPM	FR8-2 TPM	FR8-3 TPM	CK1_CK2_CK3_vs_FR2-1_FR2-2_FR2-3 power value	FR2-1_FR2-2_FR2-3_vs_FR8-1_FR8-2_FR8-3 power value	CK1_CK2_CK3_vs_FR8-1_FR8-2_FR8-3 power value
hbr-miR6485	34.79	0.00	16.34	19.55	11.25	9.41	13.76	44.44	30.89	0.06	0.30	0.20
osa-miR5523	0.00	6.46	0.00	3.26	30.01	12.55	0.00	3.70	0.00	0.37	0.42	0.05
peu-miR2916	0.00	0.00	0.00	0.00	0.00	3.14	3.44	0.00	0.00	0.14	0.05	0.16
rgl-miR5141	8.70	32.30	9.80	16.29	41.26	12.55	17.19	7.41	0.00	0.09	0.30	0.13
novel_miR_1	1113.30	1369.70	901.74	811.41	1046.44	1041.24	1017.92	1103.69	1008.98	0.25	0.09	0.10
novel_miR_10	162.36	122.76	163.36	175.97	243.79	200.72	141.00	125.92	102.96	0.37	0.65	0.11
novel_miR_100	78.28	90.45	124.15	156.42	198.79	128.59	141.00	103.70	72.07	0.48	0.39	0.06
novel_miR_101	565.35	1285.71	522.75	661.51	877.66	856.20	787.51	970.36	813.36	0.05	0.06	0.06
novel_miR_102	391.40	458.72	411.66	684.32	1087.70	668.03	663.71	618.51	730.99	0.62	0.13	0.30
novel_miR_103	1185.78	1653.98	1146.78	2310.39	2587.97	2524.70	2259.36	1996.27	2069.43	0.75	0.13	0.43
miR447c_3p_3	179.75	290.74	251.57	293.28	502.59	354.40	364.52	300.00	298.58	0.50	0.14	0.20
novel_miR_105	205.84	161.52	150.29	348.68	341.31	254.04	151.31	207.40	205.91	0.63	0.54	0.06
novel_miR_106	556.65	710.70	575.02	589.82	697.63	599.03	536.47	581.47	494.19	0.06	0.36	0.27
novel_miR_107	385.60	542.71	280.98	482.28	502.59	470.44	402.35	544.44	514.78	0.21	0.05	0.22
novel_miR_108	136.26	174.44	101.28	195.52	210.04	213.27	196.02	185.18	133.84	0.58	0.19	0.19
novel_miR_109	637.83	936.83	578.29	700.61	1005.18	1198.06	863.17	907.39	648.63	0.32	0.16	0.08
novel_miR_11	5.80	6.46	9.80	19.55	22.50	37.64	27.51	7.41	10.30	0.56	0.24	0.14
novel_miR_110	423.29	452.26	437.80	668.03	480.09	551.98	649.95	577.77	535.38	0.42	0.06	0.53
miR5208a	603.04	923.90	545.62	707.13	1233.98	1213.74	1107.33	1133.32	772.18	0.38	0.06	0.30
miR399b_1	26794.62	28815.46	37833.86	9235.06	9673.02	11550.89	29729.36	38232.77	36868.87	0.56	0.70	0.07
novel_miR_113	142.06	271.36	94.75	241.14	262.55	338.72	192.58	188.89	247.10	0.46	0.22	0.10
novel_miR_114	165.26	245.51	137.22	149.90	258.80	275.99	175.38	218.52	154.44	0.19	0.19	0.05
novel_miR_115	495.77	704.23	450.87	355.19	476.34	639.80	636.20	677.77	628.04	0.09	0.36	0.17
miR5595a_1	7969.96	9251.96	7994.77	6628.13	7471.36	9596.99	7610.30	7907.29	7793.84	0.11	0.05	0.14

miR3444a_5p	391.40	762.38	251.57	309.57	506.34	567.67	433.30	370.36	463.31	0.05	0.06	0.07
novel_miR_118	55.09	83.99	78.41	182.49	165.03	78.41	68.78	170.37	72.07	0.39	0.15	0.12
novel_miR_119	391.40	497.49	264.64	479.02	708.88	489.26	646.51	640.73	483.90	0.34	0.06	0.45
novel_miR_12	878.46	1673.36	771.05	1111.21	1597.79	1198.06	1358.37	1670.35	1441.40	0.11	0.11	0.29
novel_miR_120	240.64	303.66	251.57	276.99	397.57	335.58	405.79	407.40	329.46	0.27	0.13	0.58
novel_miR_121	22321.12	29254.80	18910.40	14113.28	26389.82	38303.28	36184.19	33836.54	34058.15	0.07	0.24	0.38
miR166_2	7975.76	8114.85	11637.67	17150.36	14155.08	15279.91	18432.55	12673.89	13209.37	0.57	0.06	0.47
novel_miR_123	107.27	213.21	120.89	146.64	198.79	203.86	220.09	162.96	175.03	0.19	0.05	0.22
miR162_2	7532.18	9510.40	10304.66	18883.97	15857.89	12654.85	15984.04	14396.08	13600.61	0.63	0.07	0.47
novel_miR_125	376.90	788.23	359.39	485.54	885.16	799.75	698.10	599.99	823.66	0.26	0.05	0.23
novel_miR_126	3519.66	5692.02	3711.51	5738.51	6661.22	6586.17	5430.04	5122.15	4293.30	0.58	0.31	0.11
novel_miR_127	556.65	710.70	575.02	589.82	697.63	599.03	536.47	581.47	494.19	0.06	0.36	0.27
miR862_3p	2186.01	3185.21	1633.59	3731.17	3941.97	2957.50	2740.81	3785.13	2707.77	0.48	0.11	0.22
novel_miR_129	536.36	956.21	532.55	570.27	1042.69	849.93	835.65	703.69	669.22	0.16	0.09	0.07
novel_miR_13	57.98	58.15	120.89	159.67	262.55	147.40	141.00	129.63	92.66	0.59	0.27	0.13
miR9563b_5p_3	144.96	264.90	156.82	270.47	408.82	279.13	261.36	270.37	226.51	0.55	0.19	0.17
novel_miR_131	292.82	368.27	245.04	485.54	532.60	382.63	471.13	566.66	339.76	0.45	0.05	0.41
miR5225a_2	327.61	542.71	329.98	664.77	873.91	599.03	563.98	522.21	442.71	0.62	0.31	0.12
niR1520f_5p_1	1342.34	2209.62	1006.29	1081.88	1297.74	1292.14	1299.91	1785.16	1245.78	0.16	0.11	0.06
miR6151e	37.69	122.76	68.61	156.42	131.27	147.40	127.24	74.07	41.18	0.45	0.41	0.05
miR395d_3p_2	150.76	193.83	111.08	117.31	176.28	194.45	202.90	188.89	154.44	0.07	0.11	0.19
novel_miR_136	3722.60	4348.16	4694.93	5777.62	8018.96	8028.85	6434.20	8296.17	6022.98	0.59	0.06	0.48
miR1520f_5p_1	359.50	465.18	264.64	511.61	453.83	366.94	323.26	488.88	319.17	0.21	0.16	0.05
miR167f_3	1629.36	1356.78	2349.10	2734.02	1477.77	2013.49	1815.74	1588.87	1945.89	0.13	0.13	0.05
miR7504h	652.33	1589.37	705.71	469.25	1083.95	1840.99	962.89	955.54	792.77	0.07	0.10	0.06
novel_miR_14	10022.61	12766.67	9265.70	14067.66	16367.99	14743.61	13146.94	12336.85	10336.88	0.63	0.37	0.10
novel_miR_140	872.67	839.91	810.26	1231.78	836.40	1116.51	1447.78	1755.53	1441.40	0.12	0.41	0.72
novel_miR_141	1954.08	2952.62	1300.33	2290.84	3544.40	4212.01	4253.93	3870.31	3572.61	0.32	0.10	0.57
novel_miR_142	246.43	303.66	202.56	492.06	435.08	401.44	240.72	348.14	195.62	0.59	0.55	0.05
miR9783	278.33	594.40	310.38	540.94	791.39	777.80	667.15	599.99	566.26	0.56	0.10	0.32
miR172k	429.09	348.89	787.39	3180.46	1582.79	310.49	918.19	744.43	648.63	0.35	0.24	0.06
miR8743e	191.35	374.73	140.49	270.47	326.31	244.63	230.41	314.81	277.98	0.12	0.05	0.10

novel_miR_146	771.19	885.14	722.05	843.99	847.65	762.11	622.44	777.77	782.47	0.07	0.30	0.18
miR160g_4	81.18	64.61	84.95	94.50	63.76	97.22	72.22	70.37	51.48	0.10	0.38	0.17
novel_miR_149	142.06	316.58	150.29	303.06	281.30	228.95	281.99	174.07	226.51	0.24	0.13	0.07
novel_miR_15	426.19	652.55	362.66	765.79	825.15	790.34	821.90	785.17	761.88	0.59	0.05	0.58
novel_miR_150	40.59	77.53	42.47	97.76	123.77	125.45	127.24	107.41	72.07	0.61	0.08	0.42
miR5568g_3p	991.53	1505.38	833.13	1202.45	1323.99	1194.92	1217.37	1314.80	988.39	0.12	0.07	0.07
novel_miR_152	2661.49	4038.04	2329.49	2815.49	4065.74	4469.19	3996.01	4036.98	4056.50	0.23	0.07	0.36
novel_miR_153	307.32	361.81	421.47	413.85	525.10	454.76	632.76	614.81	494.19	0.21	0.26	0.68
novel_miR_154	60.88	142.14	55.54	45.62	123.77	232.08	168.51	100.00	82.37	0.16	0.06	0.09
novel_miR_156	1064.01	788.23	1519.24	1544.61	1023.94	1935.08	1265.52	940.73	988.39	0.24	0.30	0.05
miR530a	95.67	122.76	104.55	286.76	228.79	297.95	230.41	303.70	216.21	0.68	0.06	0.56
miR5291b	231.94	426.42	202.56	221.59	341.31	250.90	278.55	314.81	216.21	0.06	0.05	0.06
novel_miR_159	649.43	678.39	676.30	651.73	483.84	661.75	433.30	555.55	391.24	0.12	0.32	0.61
miR8552b	110.17	116.30	94.75	283.50	135.02	185.04	213.21	203.70	154.44	0.47	0.06	0.39
novel_miR_160	7137.89	11558.49	6599.69	12118.98	13810.02	10202.29	8583.51	12533.15	8545.42	0.41	0.18	0.11
novel_miR_161	150.76	206.75	212.37	290.02	255.05	272.86	199.46	185.18	185.32	0.60	0.60	0.05
novel_miR_162	191.35	329.50	248.31	211.81	435.08	319.90	257.92	159.26	216.21	0.16	0.36	0.10
miR4387e	78.28	96.91	120.89	202.04	168.78	517.48	220.09	118.52	133.84	0.44	0.25	0.08
novel_miR_164	113.07	187.37	143.76	185.74	157.53	329.31	230.41	196.29	226.51	0.32	0.05	0.28
novel_miR_165	185.55	316.58	127.42	316.09	363.82	310.49	147.87	211.11	185.32	0.40	0.56	0.07
miR319b_1	2009.16	1918.88	3551.42	2861.11	2115.39	3434.22	2331.58	2133.30	2872.50	0.09	0.11	0.05
novel_miR_167	200.05	607.32	284.24	273.73	738.88	542.57	388.60	362.96	226.51	0.17	0.24	0.06
miR538c	565.35	930.36	640.37	1042.77	1012.68	1132.19	1327.42	996.28	1276.67	0.39	0.10	0.65
novel_miR_169	171.05	135.68	212.37	195.52	101.27	166.22	244.16	196.29	216.21	0.08	0.44	0.25
novel_miR_17	1609.07	2416.36	1692.40	1795.53	2062.88	1458.37	1461.54	2011.08	1585.54	0.08	0.06	0.13
novel_miR_170	556.65	710.70	575.02	586.56	701.38	602.16	536.47	581.47	483.90	0.06	0.36	0.27
novel_miR_171	724.81	1246.95	607.69	853.77	1226.47	1492.86	1234.57	1144.43	1153.12	0.29	0.05	0.27
novel_miR_172	530.56	807.61	519.48	593.08	753.89	727.61	790.95	566.66	555.97	0.12	0.09	0.05
novel_miR_173	374.00	762.38	307.11	351.94	585.11	1050.65	670.59	533.33	442.71	0.15	0.09	0.06
novel_miR_174	1017.63	2119.16	797.19	951.53	1500.27	1568.14	1434.02	1399.98	1317.85	0.05	0.05	0.06
miR474b_2	150.76	213.21	101.28	221.59	210.04	266.58	240.72	244.44	205.91	0.46	0.05	0.44
novel_miR_176	110.17	161.52	120.89	172.71	300.05	439.08	302.62	177.78	113.25	0.47	0.21	0.11

novel_miR_177	803.08	1318.02	771.05	2215.89	1991.61	1289.01	1306.78	1933.30	1235.48	0.55	0.13	0.24
novel_miR_178	591.44	956.21	450.87	459.47	656.37	1034.97	856.29	796.28	823.66	0.06	0.10	0.15
novel_miR_179	431.98	704.23	437.80	798.37	982.68	602.16	553.66	640.73	463.31	0.44	0.37	0.05
miR167f_1	37979.82	41420.62	52006.86	59985.53	55937.71	50108.20	48904.71	47032.64	46948.36	0.55	0.29	0.11
novel_miR_180	10518.38	14142.84	10360.21	7631.80	10561.93	11820.61	12032.74	10647.99	15144.96	0.15	0.30	0.08
novel_miR_181	1011.83	1246.95	986.69	1554.38	1496.52	1477.18	1079.82	1207.39	1091.34	0.66	0.57	0.06
miR393c_3p	484.17	730.08	323.45	573.53	626.36	884.43	718.73	766.66	947.20	0.21	0.11	0.47
miR5272c	455.18	516.87	457.40	462.73	731.38	504.94	508.96	525.92	442.71	0.24	0.18	0.06
novel_miR_184	182.65	329.50	166.63	211.81	255.05	241.49	247.60	222.22	236.80	0.06	0.05	0.06
novel_miR_185	142.06	200.29	84.95	143.38	251.30	185.04	127.24	137.04	185.32	0.25	0.19	0.05
novel_miR_186	434.88	458.72	444.34	677.80	502.59	567.67	670.59	588.88	555.97	0.45	0.06	0.56
miR390b_3p	208.74	277.82	111.08	169.45	240.04	413.99	312.94	222.22	247.10	0.19	0.05	0.14
miR845a_5p_1	942.25	1427.85	973.62	1371.90	1432.76	2060.53	1571.58	1137.02	926.61	0.39	0.27	0.06
novel_miR_189	4108.20	2829.86	1535.57	280.25	266.30	272.86	433.30	529.62	617.74	0.62	0.06	0.53
miR168a	2215.01	1854.27	3143.02	6787.80	3566.90	3120.59	2947.14	2885.14	2954.86	0.44	0.27	0.07
miR8565g_2	594.34	697.77	568.49	765.79	885.16	1019.29	1038.55	992.58	906.02	0.45	0.09	0.68
novel_miR_191	1113.30	1369.70	901.74	811.41	1046.44	1041.24	1017.92	1103.69	1008.98	0.25	0.09	0.10
novel_miR_192	301.52	497.49	326.72	602.85	1057.69	969.11	880.36	685.18	730.99	0.63	0.08	0.43
miR6445a_2	200.05	284.28	153.56	185.74	318.81	385.76	340.45	285.18	288.28	0.27	0.05	0.31
novel_miR_194	1055.32	1821.96	1231.72	1730.35	2565.47	1448.96	1932.67	1537.01	1564.95	0.33	0.10	0.14
miR812h	611.74	943.29	777.59	1026.48	960.18	1097.69	859.73	888.88	607.45	0.43	0.41	0.05
miR160g_3	81.18	51.69	75.14	94.50	60.01	94.09	72.22	62.96	51.48	0.17	0.34	0.08
miR5998a	362.40	962.67	470.47	664.77	1046.44	1056.92	756.56	785.17	761.88	0.38	0.12	0.14
miR5225a_3	327.61	542.71	329.98	664.77	873.91	599.03	563.98	522.21	442.71	0.62	0.31	0.12
novel_miR_199	84.08	206.75	104.55	120.57	157.53	144.27	103.17	118.52	205.91	0.06	0.05	0.06
novel_miR_2	765.39	1473.08	679.57	778.82	1308.99	1110.24	811.58	948.13	669.22	0.07	0.19	0.11
miR5565g_5p_2	345.01	323.04	225.43	436.66	348.81	376.35	347.33	448.14	380.94	0.39	0.05	0.42
miR166d_5p_2	284.12	452.26	320.18	547.46	633.87	379.49	436.74	429.62	422.12	0.49	0.18	0.14
novel_miR_201	243.53	510.41	192.76	267.21	375.07	504.94	371.40	370.36	298.58	0.12	0.07	0.06
novel_miR_202	75.38	96.91	65.34	211.81	191.28	175.63	123.80	92.59	72.07	0.70	0.56	0.07
novel_miR_203	982.84	1175.88	891.94	2026.89	2269.16	1555.59	1660.99	1733.31	1595.83	0.70	0.12	0.41
novel_miR_204	437.78	600.86	483.54	1818.34	1601.54	947.15	839.09	907.39	689.81	0.67	0.37	0.12

miR171g_5p	388.50	730.08	258.11	309.57	510.09	708.80	608.69	433.33	277.98	0.06	0.08	0.05
novel_miR_206	179.75	297.20	173.16	296.54	228.79	269.72	264.80	300.00	205.91	0.21	0.05	0.16
novel_miR_207	313.12	374.73	343.05	384.52	521.35	454.76	398.91	322.22	339.76	0.51	0.44	0.05
novel_miR_208	371.10	885.14	490.08	443.18	618.86	495.53	639.64	774.06	751.59	0.07	0.30	0.17
miR3434_5p_2	568.25	1117.73	516.21	615.89	1020.19	1000.47	976.65	1133.32	1008.98	0.11	0.13	0.33
novel_miR_21	530.56	749.46	329.98	645.22	813.90	774.66	581.18	688.88	494.19	0.37	0.23	0.07
miR533b_5p_3	365.30	497.49	271.18	589.82	558.85	511.21	422.99	629.62	401.53	0.46	0.11	0.20
novel_miR_211	437.78	465.18	434.53	664.77	472.59	558.26	649.95	577.77	525.08	0.39	0.06	0.49
miR399e_5p_1	150.76	187.37	160.09	140.12	247.55	147.40	171.95	159.26	144.14	0.07	0.11	0.06
miR5225	231.94	374.73	228.70	446.44	521.35	382.63	392.04	366.66	308.87	0.60	0.23	0.17
novel_miR_214	359.50	323.04	529.28	739.72	408.82	526.89	323.26	470.36	473.60	0.30	0.25	0.05
novel_miR_215	405.89	562.10	375.72	338.90	480.09	316.76	326.70	381.48	391.24	0.19	0.05	0.24
miR2619b_5p	211.64	342.43	153.56	195.52	311.31	263.45	220.09	274.07	226.51	0.07	0.06	0.05
miR474b_3	194.25	297.20	143.76	306.31	288.80	310.49	312.94	351.85	257.39	0.39	0.05	0.43
miR6274a	434.88	620.24	388.79	397.56	476.34	479.85	598.37	859.25	689.81	0.06	0.55	0.45
novel_miR_219	185.55	342.43	294.05	449.70	363.82	329.31	422.99	337.03	308.87	0.40	0.07	0.26
miR5225a_1	327.61	542.71	329.98	664.77	873.91	599.03	563.98	522.21	442.71	0.62	0.31	0.12
novel_miR_220	582.74	859.30	483.54	410.59	528.85	749.57	739.37	759.25	700.11	0.10	0.28	0.12
novel_miR_221	765.39	1337.40	568.49	723.42	1125.21	1176.10	1035.11	1022.21	689.81	0.09	0.07	0.05
novel_miR_222	9726.89	12372.56	8978.19	13308.39	15764.13	14276.31	12734.28	11788.71	10007.41	0.61	0.36	0.10
novel_miR_223	86.98	193.83	65.34	149.90	232.54	332.44	233.85	185.18	257.39	0.43	0.05	0.36
miR6234b_5p	400.09	626.70	490.08	540.94	798.90	514.35	536.47	514.81	483.90	0.23	0.21	0.05
novel_miR_225	3502.26	5524.04	3678.84	5692.89	6522.44	6488.94	5385.33	5092.52	4283.01	0.60	0.31	0.12
miR8565d	1426.42	2177.31	1065.10	2127.91	2921.78	3299.36	3222.26	2399.96	2553.33	0.51	0.05	0.47
miR3434_5p_3	298.62	374.73	212.37	283.50	300.05	404.58	299.18	274.07	257.39	0.11	0.20	0.07
novel_miR_228	629.13	445.80	865.80	1309.98	716.38	1326.64	980.09	822.21	864.84	0.51	0.16	0.18
novel_miR_229	318.91	245.51	192.76	283.50	251.30	272.86	220.09	337.03	236.80	0.07	0.05	0.06
novel_miR_23	287.02	458.72	209.10	117.31	273.80	366.94	371.40	244.44	494.19	0.10	0.22	0.08
miR5208a_1	69.58	206.75	98.02	123.83	202.54	200.72	202.90	200.00	164.73	0.22	0.06	0.32
miR8565g_1	756.70	904.52	715.51	1065.58	1226.47	1226.28	1289.59	1351.83	1142.82	0.52	0.08	0.70
novel_miR_232	246.43	471.64	202.56	263.95	251.30	288.54	326.70	244.44	216.21	0.09	0.05	0.10
novel_miR_233	118.87	445.80	173.16	286.76	641.37	646.07	402.35	166.66	154.44	0.38	0.39	0.05

novel_miR_234	28.99	64.61	29.40	16.29	45.01	65.86	41.27	37.04	30.89	0.05	0.07	0.06
novel_miR_235	1145.19	2054.56	1182.72	1775.97	2329.18	1900.58	1540.63	1070.35	1369.33	0.32	0.46	0.07
novel_miR_236	197.15	310.12	133.95	329.13	420.08	586.48	502.08	385.18	298.58	0.52	0.07	0.35
novel_miR_237	434.88	949.75	437.80	583.30	843.90	646.07	845.97	688.88	586.85	0.09	0.05	0.10
miR534b	110.17	155.06	29.40	55.40	90.02	232.08	82.53	70.37	61.77	0.09	0.19	0.08
miR8007a_5p_1	258.03	755.92	225.43	417.11	746.39	548.85	570.86	444.44	628.04	0.17	0.05	0.14
novel_miR_240	527.66	749.46	457.40	404.07	521.35	686.84	708.42	777.77	648.63	0.07	0.35	0.23
miR166d_5p_4	153.66	226.13	186.23	299.80	363.82	288.54	264.80	285.18	205.91	0.67	0.23	0.22
novel_miR_242	565.35	439.34	614.23	648.47	408.82	332.44	316.38	403.70	277.98	0.11	0.22	0.47
novel_miR_243	156.56	187.37	111.08	218.33	232.54	210.13	199.46	177.78	164.73	0.62	0.26	0.16
novel_miR_244	46.39	32.30	19.60	110.79	82.52	75.27	116.92	129.63	154.44	0.32	0.21	0.76
miR7717c_3p	649.43	1111.27	699.18	1485.95	1616.55	1031.83	1207.06	1118.50	761.88	0.53	0.25	0.12
novel_miR_246	185.55	381.19	120.89	241.14	337.56	366.94	268.23	155.55	133.84	0.18	0.35	0.08
miR6105b	217.44	303.66	307.11	583.30	566.35	564.53	330.14	433.33	380.94	0.77	0.41	0.16
miR447c_3p_4	179.75	290.74	251.57	293.28	502.59	354.40	364.52	300.00	298.58	0.50	0.14	0.20
miR396b_3	2858.63	3327.35	3518.75	8560.52	6953.77	5341.07	5598.54	5577.70	5981.80	0.70	0.13	0.38
novel_miR_25	1122.00	2093.32	1257.86	1658.66	2306.67	2176.57	2114.93	1874.05	1719.38	0.38	0.07	0.23
miR533b_5p_4	72.48	122.76	127.42	133.61	168.78	163.09	99.73	103.70	144.14	0.47	0.34	0.06
miR9563b_5p_2	623.33	936.83	470.47	1059.07	1436.51	934.61	1148.60	988.87	854.54	0.53	0.10	0.29
novel_miR_252	310.22	316.58	114.35	312.83	506.34	341.85	261.36	418.51	226.51	0.34	0.15	0.09
novel_miR_253	9990.72	12721.45	9229.76	14012.27	16315.48	14709.11	13081.61	12281.30	10326.58	0.63	0.37	0.10
novel_miR_254	542.15	956.21	539.08	576.78	1053.94	862.47	852.85	703.69	700.11	0.17	0.08	0.08
miR2873b	229.04	445.80	231.97	355.19	427.58	439.08	615.56	511.10	298.58	0.17	0.10	0.38
novel_miR_256	371.10	471.64	329.98	319.35	390.07	435.94	550.23	570.36	555.97	0.05	0.60	0.55
miR812o_5p_2	130.47	258.43	166.63	211.81	311.31	228.95	154.75	85.18	61.77	0.17	0.62	0.25
miR9563b_5p_1	147.86	277.82	169.89	273.73	423.83	282.26	275.11	281.48	226.51	0.51	0.17	0.16
miR6297a	110.17	129.22	107.82	146.64	217.54	203.86	130.68	107.41	41.18	0.40	0.60	0.08
miR820c	197.15	232.59	294.05	325.87	416.33	467.30	398.91	292.59	267.69	0.60	0.21	0.19
miR408_5p	49.29	142.14	179.69	104.28	41.26	31.36	141.00	207.40	154.44	0.24	0.55	0.13
miR845a_5p_2	942.25	1427.85	973.62	1375.16	1432.76	2063.67	1571.58	1137.02	926.61	0.39	0.28	0.06
novel_miR_262	576.95	239.05	686.11	42.36	22.50	56.45	34.39	37.04	102.96	0.60	0.05	0.57
miR1520f_5p_7	434.88	704.23	428.00	576.78	626.36	586.48	484.89	885.17	658.92	0.10	0.10	0.26

novel_miR_264	431.98	639.63	447.60	1107.95	1391.50	1301.55	1272.40	1244.43	1204.60	0.68	0.05	0.65
novel_miR_265	487.07	1027.28	375.72	762.53	971.43	564.53	512.40	762.95	504.49	0.11	0.15	0.05
miR167a_3p	2522.32	3734.38	1921.10	2668.85	2978.04	2860.28	2579.18	3688.83	2769.54	0.06	0.07	0.09
miR166d_5p_1	226.14	394.11	287.51	430.14	547.60	451.62	433.30	399.99	380.94	0.63	0.16	0.27
novel_miR_268	750.90	1188.80	679.57	749.49	1057.69	1125.92	1348.05	1233.32	1338.44	0.08	0.34	0.53
novel_miR_269	5.80	6.46	9.80	19.55	22.50	37.64	27.51	7.41	10.30	0.56	0.24	0.14
miR7484c_4	516.06	768.84	372.46	899.39	1020.19	743.30	756.56	655.55	597.15	0.56	0.28	0.11
novel_miR_270	194.25	251.97	169.89	374.75	378.82	228.95	271.67	399.99	267.69	0.43	0.06	0.35
novel_miR_272	1794.62	2571.42	1770.81	2049.70	2347.93	1627.72	1578.46	2196.26	1739.97	0.05	0.09	0.11
novel_miR_273	959.64	2073.94	1382.01	1205.71	1987.86	1289.01	1351.49	1255.54	1132.53	0.05	0.13	0.11
miR533b_5p_2	287.02	452.26	254.84	495.32	465.08	517.48	409.23	399.99	411.83	0.61	0.22	0.18
novel_miR_275	1110.40	1027.28	1003.02	1254.59	1391.50	1110.24	832.22	796.28	782.47	0.23	0.75	0.30
novel_miR_276	968.34	2338.83	901.74	1202.45	2392.94	4133.61	2469.14	1351.83	1564.95	0.29	0.15	0.08
miR5225b	1901.89	3127.06	1623.79	2385.34	3210.59	2703.47	2816.47	2996.25	2573.92	0.24	0.05	0.26
miR7122a_3p_1	805.98	1195.26	712.24	1466.40	1616.55	1103.97	887.24	1403.68	957.50	0.48	0.23	0.11
miR164e_5p_2	43.49	45.23	49.01	101.02	93.77	65.86	92.85	100.00	113.25	0.45	0.10	0.71
novel_miR_28	675.52	826.99	568.49	866.81	1072.70	1182.37	1083.26	1055.54	1111.93	0.52	0.06	0.61
novel_miR_280	1942.48	1473.08	2646.41	1906.32	967.68	1317.23	1217.37	1133.32	2491.56	0.24	0.07	0.13
miR172d_3	139.16	122.76	179.69	687.58	270.05	191.31	251.04	303.70	257.39	0.40	0.13	0.15
niR1520f_5p_1	768.29	1040.20	548.89	720.17	1035.19	1213.74	925.07	770.36	576.56	0.20	0.24	0.05
novel_miR_283	1667.05	2500.36	1728.33	1912.84	2149.14	1511.68	1502.80	2107.38	1637.02	0.07	0.07	0.12
miR396b_1	1991.77	2610.19	2633.34	6429.35	6079.86	4710.68	4879.81	4699.93	4838.98	0.74	0.11	0.47
bantam_5p	5030.15	8063.16	4423.75	3402.05	5989.84	7818.72	7376.46	6251.76	6208.30	0.05	0.10	0.09
miR5269b	371.10	846.37	300.58	329.13	630.11	815.43	732.49	622.21	463.31	0.08	0.05	0.09
novel_miR_287	1110.40	1369.70	901.74	811.41	1050.19	1056.92	1017.92	1103.69	1008.98	0.23	0.09	0.10
miR6151c	37.69	122.76	68.61	169.45	138.78	147.40	127.24	74.07	41.18	0.48	0.44	0.05
miR164e_5p_1	43.49	45.23	49.01	101.02	97.52	65.86	89.41	100.00	113.25	0.47	0.09	0.69
novel_miR_29	556.65	710.70	571.76	586.56	701.38	599.03	536.47	596.29	483.90	0.06	0.33	0.24
miR399c	2365.77	2480.97	4492.36	6738.92	5093.43	4005.02	4618.45	4336.97	4149.16	0.52	0.14	0.22
novel_miR_291	98.57	167.98	68.61	55.40	153.78	87.82	147.87	155.55	123.55	0.07	0.25	0.15
miR1888b	284.12	503.95	209.10	263.95	393.82	664.89	330.14	233.33	277.98	0.15	0.26	0.07
novel_miR_293	852.37	1356.78	1022.63	1570.68	2149.14	1746.90	1657.55	1603.68	1111.93	0.61	0.20	0.21

novel_miR_294	417.49	691.31	437.80	775.56	967.68	577.07	536.47	629.62	453.01	0.42	0.35	0.05
novel_miR_295	429.09	452.26	444.34	668.03	483.84	561.39	660.27	577.77	535.38	0.42	0.06	0.53
novel_miR_296	449.38	374.73	349.59	606.11	420.08	633.53	467.69	466.66	277.98	0.41	0.36	0.05
novel_miR_297	185.55	310.12	238.50	228.11	363.82	385.76	388.60	259.26	277.98	0.27	0.06	0.19
miR844_5p_2	86.98	187.37	101.28	218.33	180.03	116.04	158.19	122.22	92.66	0.23	0.23	0.05
miR482d_5p	150.76	329.50	212.37	198.78	277.55	392.03	285.43	237.03	164.73	0.15	0.16	0.05
miR951	162.36	290.74	107.82	143.38	191.28	232.08	154.75	107.41	133.84	0.05	0.21	0.20
novel_miR_300	539.26	736.54	450.87	443.18	851.41	733.89	1059.18	840.73	823.66	0.09	0.28	0.51
novel_miR_301	991.53	1802.58	901.74	1091.65	1361.50	1279.60	1619.73	1599.98	1338.44	0.05	0.20	0.21
novel_miR_302	437.78	381.19	411.66	697.35	626.36	467.30	443.62	655.55	483.90	0.50	0.11	0.24
novel_miR_303	307.32	251.97	316.92	413.85	502.59	335.58	333.57	351.85	308.87	0.56	0.31	0.10
novel_miR_304	150.76	109.83	78.41	123.83	183.78	188.18	288.87	270.37	236.80	0.14	0.39	0.73
miR3434_5p_4	304.42	323.04	163.36	241.14	240.04	304.22	226.97	229.63	205.91	0.05	0.16	0.17
miR5167b_3p	101.47	193.83	140.49	143.38	210.04	232.08	165.07	162.96	92.66	0.26	0.30	0.05
novel_miR_307	5.80	6.46	9.80	19.55	22.50	37.64	27.51	7.41	10.30	0.56	0.24	0.14
novel_miR_308	371.10	471.64	329.98	319.35	390.07	435.94	550.23	570.36	555.97	0.05	0.60	0.55
novel_miR_309	202.95	277.82	254.84	221.59	435.08	435.94	526.15	270.37	370.64	0.25	0.06	0.34
miR8032f_3p	89.88	142.14	91.48	149.90	123.77	131.72	68.78	88.89	72.07	0.19	0.65	0.24
miR533b_5p_5	405.89	633.16	382.26	710.39	675.12	624.12	598.37	622.21	648.63	0.55	0.08	0.35
novel_miR_312	1620.67	2061.02	1558.44	1639.11	2171.65	1972.71	2290.31	2433.30	2172.39	0.11	0.30	0.57
miR8762a	60.88	174.44	120.89	234.62	225.04	213.27	220.09	111.11	92.66	0.50	0.34	0.07
novel_miR_314	37.69	103.37	52.27	52.14	78.76	103.50	106.61	96.29	113.25	0.09	0.22	0.42
miR844_5p_3	86.98	187.37	101.28	218.33	180.03	116.04	158.19	118.52	92.66	0.22	0.24	0.05
novel_miR_316	316.02	368.27	297.31	417.11	457.58	599.03	436.74	370.36	463.31	0.60	0.15	0.25
novel_miR_317	556.65	710.70	575.02	586.56	697.63	599.03	536.47	581.47	483.90	0.06	0.36	0.27
novel_miR_318	863.97	659.01	708.98	984.12	986.43	768.39	766.88	785.17	689.81	0.41	0.39	0.05
miR166d_5p_3	255.13	432.88	251.57	426.89	633.87	388.90	392.04	318.51	308.87	0.43	0.32	0.06
miR2636	229.04	381.19	310.38	439.92	618.86	423.40	467.69	300.00	329.46	0.51	0.28	0.10
novel_miR_320	1704.74	2545.58	1770.81	1974.75	2205.40	1546.18	1557.83	2166.63	1698.79	0.06	0.07	0.11
novel_miR_321	8054.04	12114.12	5622.80	5360.51	8078.97	12046.42	8855.19	8725.80	8926.37	0.05	0.05	0.05
novel_miR_322	243.53	316.58	254.84	293.28	420.08	344.99	409.23	425.92	350.05	0.30	0.12	0.59
novel_miR_323	188.45	161.52	147.02	332.38	307.56	260.31	151.31	196.29	195.62	0.67	0.57	0.06

miR395m	21373.07	12295.03	20380.63	5533.22	5029.67	8427.16	2696.10	2666.63	1904.70	0.48	0.10	0.72
novel_miR_325	431.98	704.23	437.80	798.37	982.68	602.16	553.66	640.73	463.31	0.44	0.37	0.05
novel_miR_326	3519.66	5692.02	3711.51	5738.51	6661.22	6586.17	5430.04	5122.15	4293.30	0.58	0.31	0.11
novel_miR_327	121.77	155.06	130.69	290.02	67.51	178.77	257.92	162.96	82.37	0.11	0.05	0.08
novel_miR_328	1704.74	2545.58	1770.81	1974.75	2205.40	1546.18	1557.83	2166.63	1698.79	0.06	0.07	0.11
novel_miR_329	542.15	956.21	529.28	563.75	1031.44	846.79	821.90	696.29	658.92	0.15	0.09	0.06
miR171d	3835.67	3101.22	3424.00	3415.08	2771.76	3584.76	3882.53	3629.58	4118.28	0.09	0.45	0.24
miR5181c_3p	1826.51	3185.21	1911.30	2489.62	2479.20	2361.61	2090.86	2144.41	1770.86	0.07	0.23	0.14
novel_miR_331	785.69	755.92	555.42	615.89	573.85	1100.83	952.58	1114.80	978.09	0.06	0.29	0.42
novel_miR_332	208.74	264.90	202.56	306.31	341.31	338.72	306.06	344.44	267.69	0.64	0.08	0.44
novel_miR_333	75.38	90.45	58.81	74.95	165.03	141.13	106.61	74.07	123.55	0.43	0.14	0.15
novel_miR_334	211.64	264.90	169.89	182.49	412.58	304.22	347.33	225.92	277.98	0.26	0.06	0.18
miR162_1	7532.18	9510.40	10304.66	18883.97	15857.89	12654.85	15984.04	14396.08	13600.61	0.63	0.07	0.47
novel_miR_336	182.65	251.97	173.16	110.79	303.81	376.35	299.18	240.74	144.14	0.14	0.08	0.07
miR396b_3p	37284.01	36445.75	32717.47	141517.37	68082.43	65996.55	55902.89	55114.00	46783.63	0.55	0.31	0.10
novel_miR_338	5.80	6.46	9.80	19.55	22.50	37.64	27.51	7.41	10.30	0.56	0.24	0.14
miR7994a	246.43	368.27	235.24	524.65	491.34	558.26	605.25	633.32	288.28	0.47	0.05	0.42
miR4383_1	168.15	277.82	186.23	166.19	285.05	269.72	281.99	362.96	360.35	0.08	0.34	0.53
miR902j_5p	536.36	982.05	473.74	654.99	1065.19	837.38	828.78	844.43	833.95	0.22	0.05	0.19
miR447c_3p_2	431.98	697.77	506.41	674.54	941.42	821.70	663.71	574.07	607.45	0.56	0.34	0.09
novel_miR_343	2281.69	3055.99	3028.67	4493.70	5611.02	4600.91	5134.29	4440.67	3685.86	0.66	0.08	0.45
novel_miR_344	115.97	239.05	127.42	254.18	273.80	254.04	223.53	181.48	185.32	0.57	0.28	0.12
novel_miR_345	89.88	155.06	81.68	84.73	153.78	128.59	110.05	148.15	123.55	0.09	0.05	0.12
miR7494f	2510.73	3624.55	2417.71	4249.30	5007.16	4616.59	5072.39	4636.97	4077.09	0.59	0.05	0.58
novel_miR_347	40.59	90.45	49.01	97.76	131.27	122.31	120.36	107.41	82.37	0.59	0.08	0.39
miR395d_3p_1	1733.74	2623.11	1444.09	1655.40	2535.46	2593.70	2114.93	2081.45	1688.49	0.15	0.13	0.05
novel_miR_349	556.65	710.70	575.02	586.56	701.38	602.16	536.47	581.47	483.90	0.06	0.36	0.27
miR319b_2	2020.76	1925.34	3564.49	2867.63	2130.39	3434.22	2331.58	2133.30	2872.50	0.09	0.11	0.05
miR164e_5p_3	43.49	45.23	49.01	101.02	93.77	65.86	92.85	100.00	113.25	0.45	0.10	0.71
novel_miR_351	3539.95	5956.92	2871.85	3395.53	4969.66	5250.12	4515.29	4192.53	3932.95	0.08	0.07	0.05
miR7484m_3	78.28	193.83	137.22	260.69	236.29	194.45	209.77	166.66	205.91	0.56	0.13	0.26
novel_miR_353	481.27	652.55	294.05	407.33	765.14	768.39	605.25	522.21	370.64	0.23	0.19	0.05

miR827_5p	153.66	206.75	163.36	202.04	255.05	313.63	326.70	248.14	216.21	0.39	0.05	0.44
novel_miR_355	629.13	1078.96	454.14	524.65	877.66	743.30	639.64	777.77	710.40	0.05	0.05	0.05
bantam_c	211.64	264.90	287.51	306.31	495.09	417.12	281.99	214.81	205.91	0.47	0.57	0.06
miR9746g	626.23	891.60	392.06	472.51	656.37	708.80	674.03	662.95	535.38	0.05	0.05	0.05
miR319a_3p_2	2020.76	1925.34	3564.49	2867.63	2130.39	3434.22	2331.58	2133.30	2872.50	0.09	0.11	0.05
novel_miR_359	269.63	335.97	94.75	241.14	435.08	614.71	354.21	262.96	144.14	0.34	0.28	0.05
novel_miR_36	197.15	129.22	173.16	198.78	135.02	138.00	285.43	225.92	1153.12	0.05	0.32	0.31
novel_miR_360	530.56	923.90	460.67	599.59	855.16	859.34	643.08	777.77	555.97	0.16	0.13	0.05
miR399t_3p	3722.60	1376.16	1715.27	5474.56	4474.57	4139.88	27738.23	41099.39	37393.95	0.05	0.63	0.70
miR812o_5p_1	130.47	258.43	166.63	211.81	311.31	228.95	154.75	85.18	61.77	0.17	0.62	0.25
miR5770a	211.64	355.35	124.15	312.83	416.33	746.43	491.76	288.88	442.71	0.43	0.09	0.23
miR394a_3p	104.37	148.60	143.76	267.21	210.04	254.04	302.62	218.52	144.14	0.53	0.07	0.37
novel_miR_365	150.76	206.75	212.37	290.02	255.05	272.86	199.46	185.18	185.32	0.60	0.60	0.05
miR7494	10779.31	15570.69	9772.11	9492.50	14162.58	22195.39	15578.25	14429.42	15000.82	0.17	0.05	0.15
miR844_5p_1	86.98	187.37	101.28	218.33	180.03	116.04	158.19	118.52	92.66	0.22	0.24	0.05
novel_miR_368	176.85	219.67	241.77	172.71	363.82	263.45	199.46	192.59	247.10	0.20	0.20	0.05
novel_miR_37	446.48	484.56	375.72	475.77	588.86	508.08	464.25	699.99	494.19	0.22	0.07	0.34
miR6462f	727.70	1059.58	506.41	563.75	821.40	827.98	942.26	825.91	823.66	0.05	0.15	0.11
miR2600c	1246.67	1731.51	1068.37	1616.30	1721.56	1467.77	1516.56	1825.90	1637.02	0.24	0.06	0.34
miR172d_2	133.36	116.30	166.63	668.03	262.55	185.04	247.60	300.00	257.39	0.41	0.12	0.16
miR5638b	133.36	245.51	98.02	110.79	116.27	188.18	147.87	133.33	277.98	0.07	0.15	0.08
novel_miR_374	1629.36	969.13	1976.64	3402.05	2032.87	2857.14	2696.10	2303.67	2368.01	0.57	0.08	0.36
novel_miR_375	101.47	361.81	147.02	276.99	401.32	410.85	281.99	155.55	175.03	0.39	0.38	0.05
miR5084_5p	533.46	775.30	411.66	583.30	727.63	1041.24	1152.03	1118.50	710.40	0.16	0.16	0.49
novel_miR_377	139.16	155.06	163.36	306.31	243.79	206.99	206.33	118.52	123.55	0.50	0.53	0.05
novel_miR_378	785.69	2351.76	764.52	850.51	1350.25	1056.92	1227.69	996.28	1256.07	0.08	0.05	0.06
novel_miR_379	316.02	652.55	509.68	772.30	918.92	658.62	515.84	429.62	401.53	0.46	0.57	0.06
miR393b_3p	2272.99	3837.75	2192.27	5754.80	6548.70	5557.47	5928.68	5503.62	4519.81	0.69	0.08	0.50
novel_miR_381	144.96	277.82	98.02	123.83	228.79	232.08	206.33	222.22	205.91	0.07	0.06	0.12
miR5163a_3p	1954.08	3747.30	2035.45	2665.59	3983.23	5839.74	4838.54	3866.61	4221.23	0.33	0.05	0.38
miR159k_3p_2	16183.46	12857.12	17397.70	11695.35	11998.44	15819.35	17308.02	16514.57	19572.11	0.18	0.55	0.18
novel_miR_384	318.91	594.40	290.78	449.70	903.91	812.29	890.68	803.69	720.70	0.37	0.07	0.54

miR1509a_3p	426.19	768.84	457.40	742.98	1128.96	947.15	928.51	840.73	710.40	0.56	0.09	0.32
miR159k_3p_1	16183.46	12857.12	17397.70	11695.35	11998.44	15819.35	17308.02	16514.57	19572.11	0.18	0.55	0.18
miR1520f_5p_3	347.91	452.26	264.64	498.58	423.83	363.81	326.70	485.18	319.17	0.20	0.12	0.06
niR1520f_5p_1	342.11	335.97	303.85	325.87	562.60	555.12	402.35	411.11	247.10	0.41	0.30	0.06
miR474b_1	150.76	213.21	101.28	224.85	210.04	254.04	240.72	244.44	205.91	0.46	0.05	0.46
miR5302b_3p	220.34	335.97	271.18	364.97	626.36	407.72	285.43	296.29	185.32	0.44	0.51	0.05
novel_miR_390	576.95	239.05	686.11	42.36	22.50	56.45	34.39	37.04	102.96	0.60	0.05	0.57
miR1520f_5p_2	974.14	1330.94	927.88	1316.50	1912.85	1621.45	1430.59	1337.02	1235.48	0.59	0.21	0.18
novel_miR_392	1333.64	1789.66	937.68	1117.72	1616.55	1859.81	1719.45	1540.72	1492.88	0.11	0.06	0.15
novel_miR_393	605.94	891.60	395.33	580.04	877.66	947.15	684.34	751.84	566.26	0.21	0.15	0.06
novel_miR_394	487.07	898.06	581.56	423.63	607.61	768.39	443.62	581.47	525.08	0.07	0.10	0.20
novel_miR_395	10990.95	16190.93	9703.50	17639.16	20302.46	16010.66	15234.36	18751.57	15515.61	0.53	0.08	0.33
novel_miR_396	60.88	142.14	55.54	45.62	123.77	232.08	168.51	100.00	82.37	0.16	0.06	0.09
novel_miR_397	1214.77	1557.07	1150.04	1541.35	1642.80	1671.63	1502.80	1759.23	1431.10	0.47	0.06	0.34
miR2600b	1310.45	1854.27	1123.91	1785.75	1871.59	1555.59	1605.97	1844.42	1698.79	0.30	0.05	0.27
novel_miR_399	243.53	510.41	192.76	267.21	375.07	504.94	371.40	370.36	298.58	0.12	0.07	0.06
miR5038b	1203.18	1977.03	1176.18	1997.56	2471.70	1935.08	1980.81	2174.04	1606.13	0.49	0.09	0.27
miR6137a	556.65	1040.20	568.49	723.42	832.65	987.93	918.19	781.47	638.33	0.14	0.08	0.07
miR533b_5p_6	1165.49	1925.34	1261.13	1710.80	2779.26	2336.52	1791.67	1414.79	1111.93	0.44	0.44	0.05
miR827	805.98	581.48	1228.46	1766.20	1447.76	1160.42	4704.43	4922.15	5827.36	0.06	0.60	0.73
miR447c_3p_1	629.13	1001.43	774.32	1039.51	1369.00	1260.78	1100.45	966.65	833.95	0.60	0.27	0.14
miR167d	37025.98	40399.80	50938.50	58251.92	54092.37	49070.10	47821.45	45895.62	45743.76	0.55	0.28	0.11
novel_miR_404	936.45	1066.04	882.14	1450.11	1308.99	1351.73	997.28	970.36	885.43	0.63	0.66	0.05
novel_miR_405	516.06	575.02	699.18	892.87	577.61	555.12	732.49	618.51	926.61	0.10	0.11	0.27
miR1862c	350.81	652.55	287.51	332.38	457.58	558.26	636.20	607.40	514.78	0.05	0.23	0.28
novel_miR_407	316.02	568.56	382.26	700.61	765.14	975.38	835.65	755.54	803.06	0.60	0.05	0.57
novel_miR_408	40.59	51.69	58.81	81.47	67.51	90.95	51.58	85.18	41.18	0.48	0.27	0.09
novel_miR_409	626.23	1085.43	382.26	472.51	862.66	900.11	594.93	611.10	432.42	0.06	0.18	0.12
miR164e_5p_4	43.49	45.23	49.01	101.02	97.52	65.86	89.41	100.00	113.25	0.47	0.09	0.69
novel_miR_410	423.29	542.71	267.91	208.55	390.07	454.76	395.47	333.33	339.76	0.11	0.05	0.10
miR5565g_5p_1	188.45	219.67	147.02	215.07	165.03	178.77	171.95	222.22	236.80	0.05	0.16	0.17
miR8762d	1406.12	2435.75	1016.09	1368.64	1995.36	2239.30	2751.13	2040.71	2192.98	0.08	0.17	0.34

novel_miR_413	5.80	6.46	9.80	19.55	22.50	37.64	27.51	7.41	10.30	0.56	0.24	0.14
novel_miR_414	2502.03	3521.17	2287.02	3597.57	3428.13	3089.23	3119.09	3299.95	2707.77	0.35	0.14	0.11
miR4383_2	121.77	316.58	111.08	172.71	161.28	238.36	202.90	174.07	113.25	0.05	0.08	0.07
miR2087_5p	287.02	348.89	228.70	501.83	712.63	504.94	381.72	296.29	411.83	0.66	0.41	0.10
novel_miR_417	411.69	516.87	463.94	1635.85	1425.26	859.34	804.70	829.62	638.33	0.68	0.35	0.13
novel_miR_418	1704.74	2545.58	1770.81	1974.75	2205.40	1546.18	1557.83	2166.63	1698.79	0.06	0.07	0.11
novel_miR_419	171.05	135.68	212.37	195.52	101.27	166.22	244.16	196.29	216.21	0.08	0.44	0.25
miR1520f_5p_8	1919.29	2881.55	1558.44	2150.72	2918.03	2966.91	2345.34	2570.33	2295.94	0.29	0.11	0.11
novel_miR_420	408.79	510.41	454.14	1622.82	1406.51	856.20	787.51	837.02	638.33	0.68	0.35	0.14
miR171	156.56	167.98	205.83	175.97	150.03	203.86	409.23	255.55	247.10	0.05	0.49	0.49
novel_miR_422	171.05	206.75	133.95	280.25	333.81	210.13	147.87	181.48	247.10	0.50	0.35	0.07
novel_miR_423	60.88	277.82	68.61	166.19	180.03	272.86	202.90	148.15	144.14	0.20	0.10	0.08
miR5079a	379.80	555.63	300.58	345.42	600.11	495.53	416.11	488.88	401.53	0.13	0.09	0.06
novel_miR_425	287.02	368.27	186.23	492.06	705.13	539.44	464.25	496.29	267.69	0.62	0.25	0.17
miR1024a	382.70	652.55	512.95	821.18	948.92	642.94	577.74	651.84	648.63	0.58	0.27	0.13
novel_miR_427	208.74	297.20	173.16	293.28	360.07	360.67	447.06	392.59	319.17	0.35	0.11	0.62
miR396b_2	2841.24	3346.73	3505.68	8518.15	6946.27	5278.34	5591.66	5562.88	5971.50	0.69	0.13	0.38
miR845a_5p_3	933.55	1382.63	944.21	1323.02	1421.51	2029.17	1533.75	1077.76	895.73	0.39	0.29	0.06
novel_miR_43	629.13	445.80	862.53	1296.95	712.63	1314.10	969.77	811.10	864.84	0.51	0.16	0.17
novel_miR_430	643.63	1040.20	669.77	625.66	1312.74	1304.69	1072.94	1088.87	823.66	0.27	0.07	0.16
novel_miR_431	173.95	277.82	81.68	270.47	337.56	301.08	299.18	388.88	308.87	0.39	0.07	0.54
novel_miR_432	159.46	226.13	114.35	205.30	311.31	319.90	196.02	188.89	164.73	0.52	0.41	0.06
novel_miR_433	1295.95	1279.25	1368.95	1199.19	1297.74	2007.21	2335.02	1796.27	2388.60	0.08	0.41	0.59
novel_miR_434	913.26	1285.71	767.79	1130.76	1481.52	1273.33	904.43	792.58	700.11	0.28	0.60	0.14
novel_miR_435	591.44	956.21	450.87	459.47	656.37	1034.97	856.29	796.28	823.66	0.06	0.10	0.15
miR7484m_1	1727.94	2493.89	1290.53	2593.90	2794.26	2176.57	1777.92	2522.18	1966.48	0.40	0.19	0.10
novel_miR_437	63.78	200.29	137.22	185.74	191.28	197.59	92.85	100.00	82.37	0.25	0.60	0.15
novel_miR_438	1968.57	1873.65	2630.07	3030.56	2269.16	2816.37	3043.43	2770.33	2501.85	0.35	0.05	0.42
novel_miR_439	478.37	568.56	414.93	622.41	1053.94	780.93	639.64	907.39	803.06	0.50	0.06	0.42
novel_miR_44	40.59	90.45	49.01	104.28	131.27	122.31	120.36	118.52	82.37	0.60	0.07	0.42
miR395b	78299.32	38952.56	61203.95	13966.64	17721.98	8721.97	7544.96	6633.24	3366.69	0.55	0.06	0.68
niR1520f_5p_1:	1510.50	2442.21	1022.63	2039.93	2404.19	1859.81	1764.16	2788.85	1822.34	0.17	0.05	0.19

novel_miR_442	1272.76	1272.79	1391.82	2026.89	1489.02	1326.64	1657.55	1848.12	1976.77	0.23	0.14	0.55
miR857	318.91	445.80	542.35	423.63	412.58	241.49	478.01	329.62	453.01	0.17	0.13	0.05
miR1919b	574.05	1066.04	627.30	857.03	847.65	1107.10	1114.21	1040.73	906.02	0.20	0.08	0.37
novel_miR_445	1110.40	1369.70	901.74	811.41	1046.44	1041.24	1017.92	1099.98	1008.98	0.25	0.09	0.10
miR845a_5p_4	321.81	568.56	222.17	133.61	341.31	508.08	422.99	388.88	370.64	0.07	0.09	0.06
miR7532a	516.06	962.67	467.21	1551.13	2047.87	2095.03	1124.52	911.10	823.66	0.72	0.48	0.09
novel_miR_448	142.06	187.37	107.82	215.07	240.04	225.81	199.46	200.00	164.73	0.66	0.20	0.23
miR1222c	127.57	193.83	156.82	175.97	251.30	163.09	168.51	200.00	113.25	0.20	0.19	0.05
novel_miR_45	81.18	122.76	49.01	162.93	195.04	250.90	361.09	292.59	123.55	0.29	0.10	0.55
novel_miR_450	142.06	232.59	163.36	312.83	363.82	191.31	244.16	366.66	226.51	0.37	0.05	0.32
novel_miR_451	1725.04	1977.03	1502.90	1000.41	1369.00	1555.59	1815.74	1855.53	1523.76	0.42	0.42	0.05
miR7726a_5p	150.76	335.97	189.50	237.88	431.33	344.99	254.48	237.03	164.73	0.32	0.35	0.05
novel_miR_453	1203.18	1318.02	931.14	1815.08	2389.19	2430.61	2386.60	2448.11	2234.17	0.57	0.06	0.68
novel_miR_454	57.98	116.30	58.81	156.42	157.53	128.59	106.61	129.63	144.14	0.63	0.10	0.36
novel_miR_455	5.80	6.46	9.80	19.55	22.50	37.64	27.51	7.41	10.30	0.56	0.24	0.14
miR4360	130.47	122.76	101.28	143.38	202.54	297.95	123.80	137.04	195.62	0.49	0.24	0.10
novel_miR_458	3255.83	4839.19	2688.88	5301.85	5325.97	4459.78	4081.98	5022.15	4087.39	0.49	0.14	0.19
miR1520f_5p_6	600.14	691.31	483.54	703.87	967.68	862.47	835.65	840.73	689.81	0.55	0.07	0.37
novel_miR_46	118.87	193.83	160.09	319.35	255.05	200.72	275.11	170.37	144.14	0.46	0.21	0.11
miR1520f_5p_5	553.75	969.13	356.12	759.27	903.91	871.88	711.85	1233.32	751.59	0.19	0.06	0.27
miR6019a	292.82	445.80	209.10	469.25	667.62	865.61	591.49	511.10	442.71	0.60	0.16	0.24
miR4401a	516.06	768.84	450.87	798.37	825.15	846.79	735.93	688.88	597.15	0.57	0.25	0.13
miR166l_5p	104.37	277.82	199.30	97.76	161.28	232.08	206.33	166.66	205.91	0.10	0.09	0.05
miR528_5p	1139.39	3773.15	5126.19	1036.26	423.83	627.25	1478.73	2374.04	2090.03	0.55	0.17	0.19
miR166_1	7546.68	7656.13	10928.69	16247.71	13262.42	14326.49	17321.78	12081.30	12498.97	0.57	0.06	0.48
novel_miR_466	168.15	271.36	140.49	149.90	285.05	291.67	178.82	229.63	216.21	0.18	0.11	0.06
miR160g_1	63.78	38.77	65.34	65.17	45.01	59.59	58.46	62.96	41.18	0.05	0.06	0.05
miR4378a	1426.42	2390.52	1215.39	1049.29	1642.80	3227.22	2104.61	1940.71	1873.82	0.08	0.05	0.08
novel_miR_469	371.10	471.64	329.98	319.35	390.07	435.94	550.23	570.36	555.97	0.05	0.60	0.55
miR7122a_3p_2	805.98	1195.26	712.24	1466.40	1616.55	1103.97	887.24	1403.68	957.50	0.48	0.23	0.11
miR157d_3p	66.68	64.61	94.75	221.59	63.76	169.36	27.51	44.44	61.77	0.31	0.54	0.09
novel_miR_471	513.16	1053.12	532.55	589.82	993.93	984.79	725.61	503.70	453.01	0.12	0.32	0.11

novel_miR_472	913.26	1285.71	767.79	1130.76	1481.52	1273.33	904.43	792.58	700.11	0.28	0.60	0.14
novel_miR_473	1823.61	2610.19	1819.82	2108.36	2396.69	1634.00	1633.48	2240.71	1760.56	0.05	0.09	0.11
novel_miR_474	2481.73	3689.15	1927.63	3564.98	4568.33	3512.62	3304.79	3444.39	2748.95	0.47	0.21	0.12
novel_miR_475	234.84	271.36	235.24	400.82	491.34	316.76	361.09	488.88	370.64	0.50	0.05	0.52
novel_miR_476	5.80	6.46	9.80	19.55	22.50	37.64	27.51	7.41	10.30	0.56	0.24	0.14
niR1520f_5p_1:	1023.43	1440.77	999.75	1456.62	2002.87	1731.22	1530.31	1481.46	1235.48	0.60	0.23	0.17
novel_miR_478	8054.04	12114.12	5622.80	5360.51	8078.97	12046.42	8855.19	8725.80	8926.37	0.05	0.05	0.05
novel_miR_479	246.43	503.95	225.43	459.47	510.09	504.94	371.40	329.62	236.80	0.39	0.44	0.05
novel_miR_48	307.32	529.79	323.45	439.92	435.08	470.44	481.45	555.55	339.76	0.13	0.05	0.17
miR8007b_5p_2	791.49	1537.69	610.96	977.60	1459.02	1643.41	1348.05	1377.76	1328.14	0.26	0.05	0.25
miR5225a_4	327.61	542.71	329.98	664.77	873.91	599.03	563.98	522.21	442.71	0.62	0.31	0.12
novel_miR_482	28.99	64.61	29.40	16.29	45.01	65.86	41.27	37.04	30.89	0.05	0.07	0.06
novel_miR_483	1116.20	814.07	604.43	1081.88	1106.45	1332.92	2114.93	2996.25	2913.68	0.07	0.54	0.71
novel_miR_484	#######	83881.43	########	11551.97	10651.94	13947.00	12957.80	14840.52	58294.21	0.64	0.06	0.54
novel_miR_485	2139.63	3760.22	1620.52	1922.61	2659.24	2653.29	3332.30	3359.21	3232.85	0.05	0.32	0.27
novel_miR_486	197.15	310.12	163.36	312.83	296.30	363.81	237.28	237.03	226.51	0.49	0.41	0.05
miR399b_2	3951.64	2784.63	3469.74	5422.42	4264.53	3224.09	15760.51	21907.08	16370.15	0.05	0.63	0.69
novel_miR_488	263.83	497.49	303.85	420.37	498.84	420.26	350.77	377.77	308.87	0.27	0.31	0.05
novel_miR_489	252.23	374.73	231.97	172.71	345.06	348.13	367.96	255.55	339.76	0.05	0.09	0.09
miR6445a_1	263.83	413.50	192.76	267.21	427.58	413.99	419.55	340.74	319.17	0.21	0.05	0.17
novel_miR_490	687.12	1066.04	405.13	622.41	1091.45	840.52	804.70	1125.91	895.73	0.10	0.07	0.21
novel_miR_491	591.44	904.52	395.33	1072.10	1005.18	1085.15	942.26	762.95	638.33	0.58	0.29	0.12
novel_miR_492	629.13	445.80	865.80	1309.98	716.38	1326.64	980.09	822.21	864.84	0.51	0.16	0.18
miR399e_5p_2	420.39	639.63	346.32	935.24	1654.05	1878.63	1413.39	929.62	761.88	0.64	0.18	0.25
miR8007b_5p_1	791.49	1537.69	610.96	977.60	1459.02	1643.41	1348.05	1377.76	1328.14	0.26	0.05	0.25
miR167f_2	147.86	142.14	202.56	329.13	356.32	169.36	268.23	151.85	175.03	0.44	0.25	0.08
novel_miR_496	63.78	174.44	71.88	205.30	288.80	188.18	123.80	151.85	102.96	0.57	0.41	0.07
miR7729a_3p	339.21	387.65	336.52	413.85	382.57	388.90	770.32	607.40	483.90	0.06	0.48	0.62
miR4401b	318.91	400.57	454.14	609.37	472.59	708.80	766.88	599.99	648.63	0.39	0.09	0.63
novel_miR_499	829.18	1544.15	722.05	1046.03	1391.50	1119.65	1234.57	1599.98	1389.92	0.09	0.14	0.33
miR160g_5	81.18	51.69	75.14	94.50	60.01	94.09	72.22	62.96	51.48	0.17	0.34	0.08
miR7484m_2	81.18	200.29	150.29	263.95	236.29	203.86	220.09	166.66	205.91	0.55	0.14	0.23

miR2907a	292.82	491.03	173.16	296.54	461.33	680.57	398.91	433.33	432.42	0.28	0.08	0.14
novel_miR_501	231.94	303.66	267.91	658.25	547.60	498.67	481.45	362.96	216.21	0.65	0.39	0.10
novel_miR_502	165.26	264.90	153.56	250.92	307.56	225.81	240.72	266.66	205.91	0.38	0.09	0.19
novel_miR_503	800.19	1395.55	620.76	1186.15	1552.78	928.34	766.88	1114.80	1029.57	0.21	0.17	0.05
miR1520f_5p_4	463.88	620.24	362.66	635.44	630.11	558.26	505.52	874.06	607.45	0.19	0.08	0.34
novel_miR_505	147.86	271.36	156.82	166.19	438.83	404.58	319.82	218.52	133.84	0.34	0.22	0.06
novel_miR_506	1942.48	1473.08	2646.41	1906.32	967.68	1317.23	1217.37	1133.32	2491.56	0.24	0.07	0.13
miR2084	1481.50	2364.68	1558.44	2662.33	3405.62	2828.92	3005.61	2936.99	2913.68	0.58	0.05	0.57
miR1919_5p	1272.76	1692.75	1218.66	3265.18	2925.53	1521.09	2077.10	2581.44	2172.39	0.51	0.08	0.32
novel_miR_51	1548.19	2112.70	1215.39	2417.93	2674.24	2571.74	2228.41	1962.93	1842.93	0.66	0.29	0.17
novel_miR_52	866.87	1027.28	937.68	1280.66	1357.75	1191.78	1076.38	1370.35	875.13	0.54	0.18	0.17
novel_miR_53	50530.56	28130.61	43002.53	6670.49	5044.67	7276.15	6086.87	4459.19	27561.57	0.65	0.07	0.48
miR773b_3p	374.00	523.33	274.44	342.16	540.10	715.07	529.59	570.36	483.90	0.25	0.05	0.24
miR5208a_2	69.58	206.75	98.02	123.83	202.54	200.72	202.90	200.00	164.73	0.22	0.06	0.32
novel_miR_56	8619.39	12262.72	7837.95	8570.29	11878.42	16192.57	15031.47	15318.29	12478.38	0.18	0.13	0.45
miR4381	289.92	562.10	261.37	544.20	540.10	379.49	412.67	614.81	257.39	0.17	0.08	0.08
miR1520f_5p_9	1490.20	2455.13	1130.44	1606.52	2325.42	2380.43	1619.73	1911.08	1472.28	0.19	0.21	0.05
miR166k	7975.76	8114.85	11637.67	17150.36	14155.08	15279.91	18432.55	12673.89	13209.37	0.57	0.06	0.47
miR533b_5p_1	481.27	678.39	362.66	857.03	750.14	708.80	560.54	799.99	525.08	0.51	0.19	0.15
novel_miR_60	690.02	575.02	673.04	681.06	697.63	699.39	715.29	666.66	442.71	0.10	0.22	0.08
miR1887	817.58	1240.49	565.22	586.56	1001.43	1282.73	1141.72	881.47	803.06	0.07	0.05	0.06
miR399c_5p	185.55	71.07	147.02	547.46	131.27	72.13	440.18	966.65	669.22	0.07	0.40	0.58
novel_miR_64	52.19	51.69	62.08	101.02	71.26	97.22	55.02	96.29	41.18	0.46	0.28	0.08
miR160g_2	63.78	38.77	65.34	65.17	45.01	59.59	58.46	62.96	41.18	0.05	0.06	0.05
novel_miR_66	788.59	1343.86	767.79	749.49	1005.18	1267.05	1031.67	985.17	1050.16	0.06	0.05	0.06
niR1520f_5p_1(	1090.11	1544.15	797.19	1137.27	1601.54	1762.58	1447.78	1618.49	1431.10	0.29	0.05	0.29
miR162_3	7532.18	9510.40	10304.66	18883.97	15857.89	12654.85	15984.04	14396.08	13600.61	0.63	0.07	0.47
novel_miR_7	753.80	1053.12	748.18	896.13	1226.47	1129.06	1375.56	1388.87	1111.93	0.22	0.19	0.62
novel_miR_70	663.92	1020.82	646.90	870.06	1282.73	1038.11	1038.55	888.88	730.99	0.39	0.18	0.10
novel_miR_71	46.39	32.30	19.60	110.79	82.52	75.27	116.92	129.63	154.44	0.32	0.21	0.76
miR394d	301.52	193.83	411.66	540.94	225.04	338.72	1251.76	396.29	535.38	0.06	0.28	0.37
novel_miR_73	118.87	142.14	39.21	78.21	105.02	128.59	185.70	155.55	113.25	0.05	0.27	0.31

miR5595a_2	7984.46	9251.96	7994.77	6634.64	7490.12	9603.26	7610.30	7910.99	7793.84	0.10	0.05	0.14
novel_miR_76	556.65	710.70	575.02	586.56	701.38	602.16	536.47	581.47	483.90	0.06	0.36	0.27
novel_miR_77	1716.34	2655.42	1610.72	1837.89	2685.49	2110.71	2331.58	2766.63	2388.60	0.09	0.13	0.29
miR319	646.53	407.03	1016.09	759.27	442.58	733.89	550.23	688.88	730.99	0.06	0.05	0.06
miR1516a_5p	159.46	465.18	130.69	394.30	551.35	780.93	632.76	344.44	380.94	0.47	0.11	0.22
miR3434_5p_1	295.72	374.73	212.37	283.50	296.30	395.17	292.31	274.07	257.39	0.10	0.20	0.07
miR8007a_5p_2	6401.48	10931.79	4871.36	4910.81	9102.91	9891.80	9756.18	8292.47	8895.48	0.06	0.09	0.14
miR172d_1	133.36	116.30	166.63	668.03	262.55	185.04	247.60	300.00	257.39	0.41	0.12	0.16
novel_miR_82	568.25	710.70	575.02	586.56	697.63	599.03	539.91	581.47	483.90	0.05	0.36	0.30
novel_miR_83	817.58	788.23	754.72	1160.09	757.64	1050.65	1396.20	1674.05	1389.92	0.11	0.44	0.72
novel_miR_84	1707.64	2061.02	1372.21	1397.97	2122.89	2392.97	1918.91	1896.27	1606.13	0.15	0.09	0.06
novel_miR_85	37.69	103.37	52.27	52.14	78.76	103.50	106.61	96.29	113.25	0.09	0.22	0.42
novel_miR_86	57.98	116.30	58.81	156.42	157.53	128.59	106.61	129.63	144.14	0.63	0.10	0.36
novel_miR_87	1113.30	1382.63	905.01	821.18	1042.69	1047.51	1017.92	1111.09	1008.98	0.25	0.09	0.11
miR4383_3	1756.93	2513.28	1061.83	1674.95	2351.68	2392.97	2561.99	2540.70	2316.53	0.14	0.12	0.38
novel_miR_89	139.16	155.06	163.36	306.31	243.79	206.99	206.33	118.52	123.55	0.50	0.53	0.05
miR319a_3p_1	2009.16	1918.88	3551.42	2861.11	2115.39	3434.22	2331.58	2133.30	2872.50	0.09	0.11	0.05
miR6459a_3p	371.10	743.00	388.79	514.87	671.37	464.17	584.61	640.73	545.67	0.08	0.07	0.14
novel_miR_91	318.91	226.13	196.03	267.21	251.30	269.72	206.33	325.92	236.80	0.07	0.05	0.06
miR5285a	156.56	245.51	104.55	254.18	247.55	144.27	168.51	237.03	195.62	0.18	0.06	0.11
novel_miR_93	287.02	471.64	274.44	364.97	510.09	379.49	450.50	340.74	339.76	0.20	0.09	0.08
novel_miR_94	2502.03	3521.17	2287.02	3597.57	3428.13	3089.23	3119.09	3299.95	2707.77	0.35	0.14	0.11
miR169c_3p	307.32	471.64	156.82	312.83	506.34	467.30	392.04	274.07	247.10	0.23	0.25	0.05
novel_miR_96	443.58	775.30	346.32	446.44	772.64	693.12	433.30	544.44	514.78	0.15	0.19	0.05
novel_miR_97	1962.77	2345.29	1672.79	1306.73	1920.35	1819.04	2101.17	2262.93	1832.63	0.23	0.32	0.06
novel_miR_98	260.93	342.43	271.18	514.87	581.36	489.26	440.18	451.85	329.46	0.73	0.26	0.24
novel_miR_99	313.12	555.63	336.52	599.59	1267.73	655.48	677.46	737.03	576.56	0.49	0.12	0.21

Note: CK1-FR8-3 indicated TPM value of miRNA expression in different samples. CK1_CK2_CK3_vs_FR2-1_FR2-2_FR2-3, FR2-1_FR2-2_FR2-3_vs_FR8-1_FR8-2_FR8-3, CK1_CK2_CK3_vs_FR8-1_FR8-2_FR8-3 indicated The power value of miRNA expression between different treatments was compared, respectively.
