# Supplementary material for: RNA sequencing-based exploration of the effects of far-red light on microRNAs involved in the shade-avoidance response of D. officinale
Source: PeerJ. 2023 Mar 20;11:e15001. doi: 10.7717/peerj.15001 (PMC10035421; doi:10.7717/peerj.15001)
Supplement: Table S2 [file peerj-11-15001-s002.docx]

| Table S2 Primers information used for real-time PCR analysis of *D. officinale* genes | | | |
| --- | --- | --- | --- |
| **Gene name** | **Primer sequences（5’→3’）** | **Size（bp）** | **TM（°C）** |
| novel_miR_53 | ATTGGGGGATTGCTACCTT | 19 | 60 |
| miR395b | CTGAAGTGTTTGGGGGAACTC | 21 | 60 |
| miR399c | GGCAAATCTCCTTTGGCAGA | 20 | 60 |
| miR399t_3p | CCAAAGGAGATTTGCCCAG | 19 | 60 |
| novel_miR_36 | GCGCCGAGCTTTGTTGTGTA | 20 | 60 |
| novel_miR_483 | GCTTCTGATTGATCTCTGAACGG | 23 | 60 |
| novel_miR_390 | GCTGGTCTTATTTGAGCATGA | 21 | 60 |
| novel_miR_159 | GCTGGATGTGAAATTTATGCAT | 22 | 60 |
| novel_miR_178 | TTCCATCTGAAACGTTTGAATC | 22 | 60 |
| novel_miR_405 | GCGCTTACGTGATGAAGAAAACT | 23 | 60 |
| 5.8 s rRNA | GTCTGCCTGGGTGTCACAA | 19 | 60 |
| *THF*2-F | GAATCCATAAGGAGAACCTGGC | 183 | 60 |
| *THF*2-R | GAGCATAGTTGGTGACGGCT |  |  |
| *ASA*1-F | TCACCAGGGAGAAGCCAGTA | 183 | 60 |
| *ASA*1-R | CTCAGTTTCCTCTCATCGCAG |  |  |
| *GGP*1-F | GCTATGCTGAGAAACAGGCG | 121 | 60 |
| *GGP*1-R | CTCATCATAATCTTTCCTCCGC |  |  |
| *CYP*86*B*1-F | TCCTGATGGAACTGTGCTGAA | 229 | 60 |
| *CYP*86*B*1-R | CTGCGACGAACTTCATCTGG |  |  |
| *YUCCA*2-F | AGTTCTTGATGTTGGCACCCT | 163 | 60 |
| *YUCCA*2-R | TTTAGGCTTCGTTTGGGATG |  |  |
| *PPT*11-F | GTGGTGTTGAGAGCAAACGAC | 209 | 60 |
| *PPT*11-R | GCGTTAGGTAGTTGACAGCCAG |  |  |
| *DCL*2a-F | TTTCGTCGTTGCTGATTCG | 101 | 60 |
| *DCL*2a-R | TCTATTCTCCTCCTGCGATGC |  |  |
| *PHYA*-F | ACATCAAGACCCTCACAGTCCT | 191 | 60 |
| *PHYA*-R | TCTGTTCCAATGCCGAGTG |  |  |
| *PIF*3-F | AAGATTTAGGCTCATTCGGGAC | 131 | 60 |
| *PIF*3-R | TTTGGGACAGGCTTTCTTACTG |  |  |
| *PIF*4-F | CAAATGCCTCCTCAGGTTTCT | 203 | 60 |
| *PIF*4-R | AAAGCGTCACACTCATCCCA |  |  |
| *SPA*1-F | TTTGGGCTCACTAACGCTC | 169 | 60 |
| *SPA*1-R | TCCTCAGATGTTGACGGAAC |  |  |
| *actin*-F | AGCCATACTGTCCCAATCTACG | 114 | 60 |
| *actin*-R | AGCCACGCTCGGTAAGAATC |  |  |
