# Supplementary material for: RNA sequencing-based exploration of the effects of far-red light on microRNAs involved in the shade-avoidance response of D. officinale
Source: PeerJ. 2023 Mar 20;11:e15001. doi: 10.7717/peerj.15001 (PMC10035421; doi:10.7717/peerj.15001)
Supplement: Table S3 [file peerj-11-15001-s003.pdf]

Table S3 miRNAs up- or downregulated in FR2-CK

#ID	CK1_TPM	CK2_TPM	CK3_TPM	FR2-1_TPM	FR2-2_TPM	FR2-3_TPM	Pvalue	FDR	log2FC	regulated
novel_miR_189	4108.199	2829.85954	1535.57135	280.245312	266.2986	272.855575	1.32E-22	6.60E-20	-3.82493503	down
novel_miR_484	191966.253	83881.4302	180681.206	11551.9725	10651.944	13946.997	1.52E-20	3.80E-18	-4.20486469	down
novel_miR_53	50530.5578	28130.6129	43002.5321	6670.49017	5044.67065	7276.14866	9.96E-16	1.66E-13	-3.19123694	down
novel_miR_262	576.945379	239.052062	686.106347	42.3626635	22.504107	56.4528775	4.71E-15	5.41E-13	-4.15939936	down
novel_miR_390	576.945379	239.052062	686.106347	42.3626635	22.504107	56.4528775	5.42E-15	5.41E-13	-4.15939723	down
miR399b_1	26794.619	28815.4647	37833.8643	9235.06064	9673.01533	11550.886	1.03E-10	8.54E-09	-2.10309458	down
miR395b	78299.3158	38952.5643	61203.9533	13966.6443	17721.9843	8721.96958	1.42E-10	1.01E-08	-2.67436258	down
miR395m	21373.072	12295.029	20380.6257	5533.21559	5029.66791	8427.16011	1.56E-07	8.72E-06	-2.03510748	down
miR528_5p	1139.39464	3773.14605	5126.19456	1036.25592	423.827349	627.254195	1.57E-07	8.72E-06	-2.67460242	down
miR399e_5p_2	420.387336	639.625787	346.320346	935.237264	1654.05186	1878.62631	1.89E-06	9.42E-05	1.249181458	up
miR7532a	516.061696	962.669113	467.20575	1551.12522	2047.87374	2095.02901	4.62E-06	0.00020938	1.168860011	up
novel_miR_264	431.984228	639.625787	447.602712	1107.94658	1391.50395	1301.55245	4.43E-05	0.0018408	0.897247319	up
novel_miR_204	437.782674	600.860587	483.541616	1818.33586	1601.54228	947.153834	0.00016149	0.00619887	1.10059286	up
novel_miR_420	408.790444	510.408456	454.137058	1622.81588	1406.50669	856.201976	0.00021664	0.00772178	1.066066015	up
novel_miR_417	411.689667	516.869323	463.938577	1635.85055	1425.26011	859.338247	0.00024511	0.00815392	1.058538515	up
novel_miR_192	301.519193	497.486723	326.717308	602.853288	1057.69303	969.107731	0.00073819	0.02302224	0.798895311	up
novel_miR_451	1725.03769	1977.02516	1502.89962	1000.41059	1368.99984	1555.5904	0.0010395	0.03051251	-0.86075914	down
novel_miR_1	1113.30164	1369.70371	901.73977	811.407939	1046.44098	1041.24196	0.0046899	0.12057268	-0.65888591	down
novel_miR_445	1110.40241	1369.70371	901.73977	811.407939	1046.44098	1041.24196	0.00476336	0.12057268	-0.65744678	down
miR530a	95.6743593	122.756464	104.549539	286.762645	228.791755	297.945743	0.00492977	0.12057268	0.89183093	up
novel_miR_191	1113.30164	1369.70371	901.73977	811.407939	1046.44098	1041.24196	0.00528307	0.12057268	-0.6588524	down
novel_miR_215	405.891221	562.095388	375.724904	338.901308	480.087616	316.763368	0.00531583	0.12057268	-0.67421221	down
miR396b_1	1991.76621	2610.19008	2633.3415	6429.34885	6079.85957	4710.679	0.00603334	0.12399955	0.805439951	up
novel_miR_87	1113.30164	1382.62544	905.006943	821.183939	1042.69029	1047.51451	0.00625145	0.12399955	-0.65781755	down
miR393b_3p	2272.99084	3837.75472	2192.27314	5754.8049	6548.69514	5557.47217	0.00644209	0.12399955	0.711314138	up
novel_miR_97	1962.77398	2345.29455	1672.79262	1306.72524	1920.35046	1819.03717	0.0064609	0.12399955	-0.69321229	down
miR1520f_5p_11	1342.34025	2209.61635	1006.28931	1081.87725	1297.73684	1292.14364	0.00696369	0.12647831	-0.6941246	down

novel_miR_280	1942.47942	1473.07757	2646.41019	1906.31986	967.676601	1317.23381	0.0075567	0.12647831	-1.02076278	down
novel_miR_287	1110.40241	1369.70371	901.73977	811.407939	1050.19166	1056.92332	0.00757106	0.12647831	-0.64794768	down
novel_miR_506	1942.47942	1473.07757	2646.41019	1906.31986	967.676601	1317.23381	0.00760391	0.12647831	-1.02076046	down
miR1516a_5p	159.457266	465.18239	130.686923	394.298637	551.350622	780.931473	0.00941992	0.14977144	0.896053117	up
miR408_5p	49.2867911	142.139064	179.694519	104.277326	41.2575295	31.3627097	0.00981683	0.14977144	-1.45887466	down
novel_miR_202	75.3797982	96.912998	65.3434616	211.813318	191.28491	175.631175	0.0100251	0.14977144	0.855751983	up
novel_miR_45	81.1782442	122.756464	49.0075962	162.933321	195.035594	250.901678	0.01029385	0.14977144	0.886643802	up
miR6019a	292.821524	445.799791	209.099077	469.247965	667.621841	865.610789	0.01050501	0.14977144	0.680547195	up
miR396b_3p	37284.0079	36445.7481	32717.4712	141517.365	68082.425	65996.5501	0.01113538	0.1543487	0.953858207	up
miR4387e	78.2790212	96.912998	120.885404	202.037318	168.780803	517.484711	0.01283683	0.17312378	1.098733759	up
novel_miR_220	582.743825	859.295249	483.541616	410.591969	528.846515	749.568763	0.01358069	0.17833591	-0.59936991	down
novel_miR_501	231.937841	303.660727	267.908192	658.250618	547.599937	498.667085	0.01582455	0.1947051	0.648918365	up
novel_miR_180	10518.3811	14142.8368	10360.2058	7631.79676	10561.9276	11820.6053	0.01605214	0.1947051	-0.66310859	down
novel_miR_115	495.767134	704.234452	450.869885	355.19464	476.336932	639.799279	0.01619067	0.1947051	-0.590319	down
novel_miR_425	287.023078	368.269392	186.228866	492.05863	705.128686	539.438608	0.01666733	0.1947051	0.62445181	up
novel_miR_99	313.116085	555.634522	336.518827	599.594622	1267.73136	655.480634	0.0167782	0.1947051	0.645554446	up
novel_miR_496	63.7829062	174.443396	71.8778077	205.295985	288.802707	188.176258	0.01955219	0.21681201	0.817053762	up
miR6105b	217.441726	303.660727	307.114269	583.30129	566.35336	564.528775	0.02050811	0.21941069	0.597730206	up
miR857	318.914531	445.799791	542.350731	423.626635	412.575295	241.492865	0.02121393	0.21941069	-0.73941256	down
novel_miR_159	649.425954	678.390986	676.304827	651.733285	483.838301	661.753176	0.02311816	0.2261905	-0.61264256	down
miR159k_3p_1	16183.4628	12857.1244	17397.6966	11695.3538	11998.4397	15819.3508	0.02357095	0.2261905	-0.73495503	down
miR159k_3p_2	16183.4628	12857.1244	17397.6966	11695.3538	11998.4397	15819.3508	0.02465879	0.22739438	-0.7349526	down
novel_miR_71	46.3875681	32.3043327	19.6030385	110.794658	82.515059	75.2705034	0.02795339	0.24321271	0.946415698	up
novel_miR_236	197.147165	310.121594	133.954096	329.125309	420.076664	586.482672	0.02810736	0.24321271	0.673287052	up
novel_miR_244	46.3875681	32.3043327	19.6030385	110.794658	82.515059	75.2705034	0.02826921	0.24321271	0.946575693	up
novel_miR_410	423.286559	542.712789	267.908192	208.554651	390.071188	454.759291	0.03001584	0.25386281	-0.65444099	down
novel_miR_176	110.170474	161.521663	120.885404	172.70932	300.05476	439.077936	0.03158816	0.26270823	0.769252124	up
novel_miR_23	287.023078	458.721524	209.099077	117.311991	273.799969	366.943704	0.03396834	0.27376884	-0.73778696	down
novel_miR_233	118.868143	445.799791	173.160173	286.762645	641.36705	646.071821	0.03421041	0.27376884	0.771104321	up
osa-miR5523	0	6.46086653	0	3.25866642	30.005476	12.5450839	0.034564	0.27376884	2.720618247	up

miR396b_3	2858.63389	3327.34626	3518.74541	8560.51669	6953.76906	5341.06947	0.0352718	0.27500981	0.656895787	up
miR396b_2	2841.23855	3346.72886	3505.67671	8518.15403	6946.26769	5278.34405	0.03597913	0.27620904	0.652023813	up
miR5770a	211.64328	355.347659	124.152577	312.831977	416.32598	746.432492	0.03770717	0.28508908	0.725492852	up
novel_miR_242	565.348487	439.338924	614.228539	648.474618	408.824611	332.444723	0.04156139	0.30498724	-0.69923177	down
miR172d_1	133.364258	116.295598	166.625827	668.026617	262.547915	185.039988	0.04391459	0.31441142	0.969357365	up
miR172d_2	133.364258	116.295598	166.625827	668.026617	262.547915	185.039988	0.04410581	0.31441142	0.969375799	up
miR172k	429.085005	348.886793	787.388712	3180.45843	1582.78886	310.490826	0.04673754	0.31600602	1.219742133	up
