# Supplementary material for: RNA sequencing-based exploration of the effects of far-red light on microRNAs involved in the shade-avoidance response of D. officinale
Source: PeerJ. 2023 Mar 20;11:e15001. doi: 10.7717/peerj.15001 (PMC10035421; doi:10.7717/peerj.15001)
Supplement: Table S4 [file peerj-11-15001-s004.pdf]

Table S4 miRNAs up- or downregulated in FR8-CK

#ID	CK1_TPM	CK2_TPM	CK3_TPM	FR8-1_TPM	FR8-2_TPM	FR8-3_TPM	Pvalue	FDR	log2FC	regulated
miR395m	21373.072	12295.029	20380.6257	2696.10372	2666.62716	1904.70307	7.61E-18	2.10E-15	-3.25772511	down
miR395b	78299.3158	38952.5643	61203.9533	7544.96372	6633.23506	3366.69138	8.41E-18	2.10E-15	-3.72604981	down
miR399t_3p	3722.60234	1376.16457	1715.26587	27738.2303	41099.3911	37393.9544	3.59E-15	5.97E-13	3.606930859	up
novel_miR_189	4108.199	2829.85954	1535.57135	433.302383	529.621783	617.741537	5.23E-15	6.53E-13	-2.71785953	down
novel_miR_390	576.945379	239.052062	686.106347	34.389078	37.0364884	102.956923	2.93E-11	2.53E-09	-3.66901295	down
novel_miR_262	576.945379	239.052062	686.106347	34.389078	37.0364884	102.956923	3.05E-11	2.53E-09	-3.66877199	down
miR399b_2	3951.64096	2784.63348	3469.73781	15760.5145	21907.0829	16370.1507	2.36E-10	1.69E-08	2.072480945	up
novel_miR_264	431.984228	639.625787	447.602712	1272.39589	1244.42601	1204.596	1.31E-09	8.20E-08	1.037907144	up
miR827	805.983996	581.477988	1228.45708	4704.42587	4922.1493	5827.36183	2.15E-09	1.19E-07	2.205017066	up
miR399c_5p	185.550273	71.0695319	147.022789	440.180199	966.652346	669.219998	5.40E-07	2.70E-05	1.954215127	up
novel_miR_483	1116.20086	814.069183	604.42702	2114.9283	2996.25191	2913.68092	9.88E-07	4.48E-05	1.363671654	up
novel_miR_484	191966.253	83881.4302	180681.206	12957.8046	14840.5209	58294.2097	5.85E-06	0.00024335	-2.72166856	down
novel_miR_244	46.3875681	32.3043327	19.6030385	116.922865	129.627709	154.435384	1.29E-05	0.00049533	1.601838276	up
novel_miR_71	46.3875681	32.3043327	19.6030385	116.922865	129.627709	154.435384	1.41E-05	0.00050395	1.602882269	up
miR399e_5p_2	420.387336	639.625787	346.320346	1413.39111	929.615858	761.881229	2.65E-05	0.00088144	0.923894241	up
novel_miR_45	81.1782442	122.756464	49.0075962	361.085319	292.588258	123.548307	3.12E-05	0.0009735	1.461548561	up
novel_miR_384	318.914531	594.399721	290.778404	890.677121	803.691797	720.69846	4.60E-05	0.00134988	0.809933153	up
novel_miR_192	301.519193	497.486723	326.717308	880.360398	685.175034	730.994152	0.00015875	0.00440101	0.790235609	up
novel_miR_453	1203.17755	1318.01677	931.144327	2386.60202	2448.11188	2234.16523	0.00018804	0.00493861	0.761802771	up
novel_miR_159	649.425954	678.390986	676.304827	433.302383	555.547325	391.236307	0.00021725	0.00542048	-0.83192569	down
miR530a	95.6743593	122.756464	104.549539	230.406823	303.699205	216.209538	0.00029505	0.00701105	0.940135919	up
novel_miR_242	565.348487	439.338924	614.228539	316.379518	403.697723	277.983692	0.00031837	0.00722124	-1.03059885	down
novel_miR_407	316.015308	568.556255	382.25925	835.654596	755.544362	803.063998	0.0004921	0.01067646	0.679898141	up
miR812o_5p_2	130.465035	258.434661	166.625827	154.750851	85.1839232	61.7741537	0.00069089	0.01379054	-1.01136364	down
miR812o_5p_1	130.465035	258.434661	166.625827	154.750851	85.1839232	61.7741537	0.00069091	0.01379054	-1.0113639	down
novel_miR_53	50530.5578	28130.6129	43002.5321	6086.86681	4459.1932	27561.5682	0.00123217	0.02364822	-1.95637273	down
novel_miR_304	150.759596	109.834731	78.4121539	288.868255	270.366365	236.800923	0.00162133	0.02996458	0.901669585	up

novel_miR_275	1110.40241	1027.27778	1003.02214	832.215688	796.2845	782.472614	0.00178505	0.0318122	-0.69121516	down
miR393b_3p	2272.99084	3837.75472	2192.27314	5928.67705	5503.62217	4519.80891	0.00372965	0.06003527	0.715544357	up
novel_miR_83	817.580888	788.225717	754.716981	1396.19657	1674.04927	1389.91846	0.00641173	0.09410155	0.614603692	up
novel_miR_141	1954.07631	2952.61601	1300.33489	4253.92895	3870.31303	3572.60522	0.00663337	0.09457284	0.701457284	up
miR396b_1	1991.76621	2610.19008	2633.3415	4879.81017	4699.93037	4838.97537	0.00687841	0.09534238	0.71194909	up
novel_miR_36	197.147165	129.217331	173.160173	285.429348	225.922579	1153.11754	0.00759419	0.10241896	1.415174644	up
novel_miR_223	86.9766903	193.825996	65.3434616	233.845731	185.182442	257.392307	0.00891167	0.11402373	0.785835068	up
novel_miR_236	197.147165	310.121594	133.954096	502.080539	385.179479	298.575076	0.00937661	0.11697317	0.700468644	up
miR157d_3p	66.6821292	64.6086653	94.7480193	27.5112624	44.443786	61.7741537	0.00962609	0.11715653	-1.25610897	down
miR528_5p	1139.39464	3773.14605	5126.19456	1478.73036	2374.0389	2090.02553	0.01628801	0.15630227	-1.03459963	down
novel_miR_431	173.953381	277.817261	81.679327	299.184979	388.883128	308.870769	0.0176173	0.15859478	0.722194154	up
miR8565d	1426.41772	2177.31202	1065.09842	3222.25661	2399.96445	2553.33169	0.01775842	0.15859478	0.589107991	up
miR8032f_3p	89.8759133	142.139064	91.4808462	68.7781561	88.887572	72.069846	0.0204301	0.17128081	-0.73555345	down
miR7994a	246.433956	368.269392	235.236462	605.247773	633.323951	288.279384	0.02099145	0.17171696	0.631452312	up
novel_miR_150	40.5891221	77.5303984	42.47325	127.239589	107.405816	72.069846	0.02421348	0.18495476	0.799435066	up
miR951	162.356489	290.738994	107.816712	154.750851	107.405816	133.844	0.02446296	0.18495476	-0.69752873	down
miR164e_5p_3	43.4883451	45.2260657	49.0075962	92.8505107	99.9985185	113.252615	0.02634025	0.19109398	0.756720901	up
miR164e_5p_2	43.4883451	45.2260657	49.0075962	92.8505107	99.9985185	113.252615	0.0268365	0.19109398	0.757229161	up
miR1516a_5p	159.457266	465.18239	130.686923	632.759036	344.439342	380.940614	0.0298945	0.20434733	0.737413215	up
novel_miR_437	63.7829062	200.286863	137.221269	92.8505107	99.9985185	82.3655383	0.03333428	0.20988663	-0.72075913	down
miR164e_5p_1	43.4883451	45.2260657	49.0075962	89.4116029	99.9985185	113.252615	0.03355521	0.20988663	0.73741028	up
miR164e_5p_4	43.4883451	45.2260657	49.0075962	89.4116029	99.9985185	113.252615	0.03364916	0.20988663	0.73749685	up
miR394d	301.519193	193.825996	411.663808	1251.76244	396.290425	535.375999	0.0349353	0.21126388	0.883926599	up
miR5770a	211.64328	355.347659	124.152577	491.763816	288.884609	442.714768	0.03678822	0.21853957	0.626160554	up
miR172d_2	133.364258	116.295598	166.625827	247.601362	299.995556	257.392307	0.04865205	0.25555128	0.593718429	up
miR172d_1	133.364258	116.295598	166.625827	247.601362	299.995556	257.392307	0.04916835	0.25557299	0.593829576	up
