# Supplementary material for: RNA sequencing-based exploration of the effects of far-red light on microRNAs involved in the shade-avoidance response of D. officinale
Source: PeerJ. 2023 Mar 20;11:e15001. doi: 10.7717/peerj.15001 (PMC10035421; doi:10.7717/peerj.15001)
Supplement: Table S5 [file peerj-11-15001-s005.pdf]

Table S5 The TPM value of differentially expressed miRNAs of *D. officinale* under different light treatments

#ID	CK1	CK2	CK3	FR2-1	FR2-2	FR2-3	FR8-1	FR8-2	FR8-3
miR5523	0	6.460866531	0	3.258666423	30.005476	12.5450839	0	3.703648835	0
miR399b_1	26794.61904	28815.46473	37833.86425	9235.060644	9673.015325	11550.886	29729.35796	38232.76692	36868.87406
miR1520f_5p_1	1342.340253	2209.616354	1006.289308	1081.877253	1297.736837	1292.143641	1299.907149	1785.158738	1245.778766
miR172k	429.0850052	348.8867927	787.3887119	3180.458429	1582.788859	310.4908264	918.1883834	744.4334158	648.6286138
miR530a	95.67435927	122.7564641	104.5495385	286.7626453	228.7917545	297.9457425	230.4068228	303.6992045	216.2095379
miR4387e	78.27902122	96.91299797	120.8854039	202.0373182	168.7808025	517.4847107	220.0900994	118.5167627	133.8439997
miR6274a	434.8834512	620.243187	388.7935963	397.5573036	476.3369315	479.849459	598.3699577	859.2465297	689.8113829
miR8565d	1426.41772	2177.312021	1065.098424	2127.909174	2921.783225	3299.357064	3222.256611	2399.964445	2553.331686
miR6105b	217.4417256	303.660727	307.1142694	583.3012898	566.3533595	564.5287753	330.1351491	433.3269137	380.9406144
miR396b_3	2858.633886	3327.346264	3518.745406	8560.516694	6953.769063	5341.069468	5598.541903	5577.695145	5981.797216
miR812o_5p_2	130.4650354	258.4346613	166.625827	211.8133175	311.3068135	228.9477811	154.7508511	85.1839232	61.77415369
miR6297a	110.1704743	129.2173306	107.8167116	146.6399891	217.539701	203.8576133	130.6784965	107.4058162	41.18276913
miR408_5p	49.28679114	142.1390637	179.6945193	104.2773255	41.2575295	31.36270974	140.9952199	207.4043348	154.4353842
miR164e_5p_2	43.48834512	45.22606572	49.00759618	101.0186591	93.7671125	65.86169045	92.85051068	99.99851854	113.2526151
miR396b_1	1991.766207	2610.190079	2633.341501	6429.348853	6079.859574	4710.679003	4879.810172	4699.930371	4838.975373
miR6151c	37.68989911	122.7564641	68.61063465	169.450654	138.7753265	147.4047358	127.2395887	74.0729767	41.18276913
miR164e_5p_1	43.48834512	45.22606572	49.00759618	101.0186591	97.517797	65.86169045	89.41160287	99.99851854	113.2526151
miR951	162.3564885	290.7389939	107.8167116	143.3813226	191.2849095	232.0840521	154.7508511	107.4058162	133.8439997
miR8032f_3p	89.87591326	142.1390637	91.4808462	149.8986555	123.7725885	131.7233809	68.77815606	88.88757204	72.06984598
miR395m	21373.07202	12295.02901	20380.62566	5533.215587	5029.667914	8427.160107	2696.103717	2666.627161	1904.703072
miR396b_3p	37284.00789	36445.7481	32717.47121	141517.3654	68082.42504	65996.5501	55902.88524	55113.99831	46783.62573
miR7994a	246.4339557	368.2693923	235.2364617	524.6452942	491.3396695	558.2562333	605.2477733	633.3239508	288.2793839
miR4383_1	168.1549345	277.8172609	186.2288655	166.1919876	285.052022	269.7193037	281.9904398	362.9575858	360.3492299
miR164e_5p_3	43.48834512	45.22606572	49.00759618	101.0186591	93.7671125	65.86169045	92.85051068	99.99851854	113.2526151
miR399t_3p	3722.602343	1376.164571	1715.265866	5474.559591	4474.566608	4139.877685	27738.23034	41099.39112	37393.95437
miR812o_5p_1	130.4650354	258.4346613	166.625827	211.8133175	311.3068135	228.9477811	154.7508511	85.1839232	61.77415369

miR5770a	211.6432796	355.3476592	124.152577	312.8319766	416.3259795	746.4324918	491.7638158	288.8846091	442.7147681
miR172d_2	133.3642584	116.2955976	166.625827	668.0266168	262.547915	185.0399875	247.6013618	299.9955556	257.3923071
miR393b_3p	2272.990838	3837.75472	2192.273136	5754.804904	6548.695137	5557.472166	5928.677052	5503.622169	4519.808912
miR159k_3p_2	16183.46283	12857.1244	17397.69664	11695.35379	11998.43972	15819.35079	17308.02297	16514.57015	19572.11103
miR159k_3p_1	16183.46283	12857.1244	17397.69664	11695.35379	11998.43972	15819.35079	17308.02297	16514.57015	19572.11103
miR5302b_3p	220.3409486	335.9650596	271.1753655	364.9706394	626.3643115	407.7152266	285.4293476	296.2919068	185.3224611
miR827	805.9839963	581.4779878	1228.457078	1766.197201	1447.764217	1160.42026	4704.425874	4922.149301	5827.361832
miR164e_5p_4	43.48834512	45.22606572	49.00759618	101.0186591	97.517797	65.86169045	89.41160287	99.99851854	113.2526151
miR171	156.5580424	167.9825298	205.8319039	175.9679869	150.02738	203.8576133	409.2300285	255.5517696	247.0966148
miR396b_2	2841.238548	3346.728863	3505.676713	8518.154031	6946.267694	5278.344049	5591.664087	5562.88055	5971.501524
miR395b	78299.31578	38952.56432	61203.95328	13966.64429	17721.98426	8721.969578	7544.96372	6633.235063	3366.691376
miR857	318.9145309	445.7997907	542.350731	423.626635	412.575295	241.492865	478.0081846	329.6247463	453.0104604
miR7532a	516.0616955	962.6691132	467.2057502	1551.125218	2047.873737	2095.029011	1124.522852	911.0976134	823.6553826
miR6019a	292.8215238	445.7997907	209.099077	469.247965	667.621841	865.6107888	591.4921421	511.1035392	442.7147681
miR528_5p	1139.394642	3773.146054	5126.19456	1036.255923	423.8273485	627.2541948	1478.730355	2374.038903	2090.025533
miR157d_3p	66.68212919	64.60866531	94.74801928	221.5893168	63.7616365	169.3586326	27.51126242	44.44378602	61.77415369
miR399b_2	3951.64096	2784.633475	3469.737809	5422.420928	4264.528276	3224.086561	15760.51446	21907.08286	16370.15073
miR399e_5p_2	420.3873362	639.6257866	346.3203463	935.2372635	1654.051864	1878.626313	1413.391107	929.6158575	761.8812289
miR7729a_3p	339.209092	387.6519919	336.5188271	413.8506358	382.569819	388.8976008	770.3153478	607.3984089	483.8975373
miR399c_5p	185.5502725	71.06953185	147.0227885	547.4559591	131.2739575	72.1342324	440.1801988	966.6523459	669.2199984
miR394d	301.5191929	193.8259959	411.6638079	540.9386263	225.04107	338.7172652	1251.76244	396.2904253	535.3759987
miR1516a_5p	159.4572655	465.1823903	130.6869231	394.2986372	551.3506215	780.9314725	632.7590357	344.4393416	380.9406144
miR172d_1	133.3642584	116.2955976	166.625827	668.0266168	262.547915	185.0399875	247.6013618	299.9955556	257.3923071
