# Supplementary material for: RNA sequencing-based exploration of the effects of far-red light on microRNAs involved in the shade-avoidance response of D. officinale
Source: PeerJ. 2023 Mar 20;11:e15001. doi: 10.7717/peerj.15001 (PMC10035421; doi:10.7717/peerj.15001)
Supplement: Table S6 [file peerj-11-15001-s006.pdf]

Table S6 The TPM value of differentially expressed novel miRNAs of *D. officinale* under different light treatments

#ID	CK1	CK2	CK3	FR2-1	FR2-2	FR2-3	FR8-1	FR8-2	FR8-3
novel_miR_1	1113.301635	1369.703705	901.7397697	811.4079394	1046.440975	1041.241963	1017.91671	1103.687353	1008.977844
novel_miR_10	205.8448336	161.5216633	150.2899616	348.6773073	341.3122895	254.0379489	151.3119433	207.4043348	205.9138456
novel_miR_11	495.7671344	704.2344519	450.8698848	355.1946401	476.3369315	639.7992787	636.1979435	677.7677368	628.0372292
novel_miR_14	872.6661255	839.9126491	810.2589235	1231.775908	836.4026435	1116.512467	1447.780185	1755.529548	1441.39692
novel_miR_14	1954.076308	2952.616005	1300.334885	2290.842496	3544.396852	4212.011918	4253.928952	3870.313032	3572.605222
novel_miR_14	246.4339557	303.660727	202.5647309	492.0586299	435.079402	401.4426846	240.7235462	348.1429905	195.6181534
novel_miR_15	40.58912212	77.53039838	42.47325002	97.7599927	123.7725885	125.450839	127.2395887	107.4058162	72.06984598
novel_miR_15	649.4259538	678.3909858	676.3048272	651.7332847	483.8383005	661.7531755	433.3023832	555.5473252	391.2363067
novel_miR_16	185.5502725	316.58246	127.4197501	316.0906431	363.8163965	310.4908264	147.8730355	211.1079836	185.3224611
novel_miR_16	171.0541575	135.6781972	212.3662501	195.5199854	101.2684815	166.2223616	244.162454	196.2933882	216.2095379
novel_miR_17	110.1704743	161.5216633	120.8854039	172.7093204	300.05476	439.0779363	302.6238867	177.7751441	113.2526151
novel_miR_18	10518.38107	14142.83684	10360.20583	7631.796763	10561.92755	11820.6053	12032.7384	10647.9904	15144.96335
novel_miR_18	4108.199003	2829.859541	1535.571347	280.2453124	266.2985995	272.8555747	433.3023832	529.6217834	617.7415369
novel_miR_19	1113.301635	1369.703705	901.7397697	811.4079394	1046.440975	1041.241963	1017.91671	1103.687353	1008.977844
novel_miR_19	301.5191929	497.4867229	326.7173078	602.8532883	1057.693029	969.1077309	880.3603975	685.1750344	730.994152
novel_miR_20	75.37979821	96.91299797	65.34346157	211.8133175	191.2849095	175.6311745	123.8006809	92.59122087	72.06984598
novel_miR_20	437.7826742	600.8605874	483.5416156	1818.335864	1601.542281	947.1538341	839.0935039	907.3939645	689.8113829
novel_miR_20	371.1005451	885.1387148	490.0759618	443.1786336	618.8629425	495.5308139	639.6368513	774.0626065	751.5855366
novel_miR_21	405.8912212	562.0953882	375.724904	338.901308	480.087616	316.7633684	326.6962413	381.47583	391.2363067
novel_miR_22	582.7438247	859.2952487	483.5416156	410.5919693	528.8465145	749.5687627	739.3651776	759.2480111	700.1070752
novel_miR_22	86.97669025	193.8259959	65.34346157	149.8986555	232.542439	332.4447232	233.8457306	185.1824417	257.3923071
novel_miR_23	287.0230778	458.7215237	209.099077	117.3119912	273.7999685	366.9437039	371.4020427	244.4408231	494.1932296
novel_miR_23	118.8681433	445.7997907	173.1601732	286.7626453	641.3670495	646.0718206	402.3522129	166.6641976	154.4353842
novel_miR_23	197.1471646	310.1215935	133.9540962	329.1253088	420.076664	586.4826721	502.0805392	385.1794788	298.5750762
novel_miR_24	565.3484866	439.3389241	614.2285388	648.4746182	408.8246105	332.4447232	316.3795179	403.697723	277.9836916
novel_miR_24	46.38756813	32.30433266	19.60303847	110.7946584	82.515059	75.27050337	116.9228653	129.6277092	154.4353842

novel_miR_25	371.1005451	471.6432568	329.9844809	319.3493095	390.071188	435.9416654	550.2252485	570.3619206	555.9673832
novel_miR_26	576.9453786	239.0520617	686.1063465	42.3626635	22.504107	56.45287753	34.38907803	37.03648835	102.9569228
novel_miR_26	431.9842282	639.6257866	447.6027118	1107.946584	1391.503949	1301.552454	1272.395887	1244.426009	1204.595997
novel_miR_27	1110.402412	1027.277778	1003.022135	1254.586573	1391.503949	1110.239925	832.2156883	796.2844995	782.4726135
novel_miR_28	1942.479416	1473.077569	2646.410194	1906.319858	967.676601	1317.233809	1217.373362	1133.316543	2491.557532
novel_miR_28	1110.402412	1369.703705	901.7397697	811.4079394	1050.19166	1056.923318	1017.91671	1103.687353	1008.977844
novel_miR_29	98.57358228	167.9825298	68.61063465	55.3973292	153.7780645	87.81558727	147.8730355	155.5532511	123.5483074
novel_miR_30	539.2554795	736.5387846	450.8698848	443.1786336	851.4053815	733.8874079	1059.183603	840.7282855	823.6553826
novel_miR_30	150.7595964	109.834731	78.41215388	123.8293241	183.7835405	188.1762584	288.8682554	270.3663649	236.8009225
novel_miR_30	371.1005451	471.6432568	329.9844809	319.3493095	390.071188	435.9416654	550.2252485	570.3619206	555.9673832
novel_miR_32	188.4494955	161.5216633	147.0227885	332.3839752	307.556129	260.3104908	151.3119433	196.2933882	195.6181534
novel_miR_36	197.1471646	129.2173306	173.1601732	198.7786518	135.024642	137.9959228	285.4293476	225.9225789	1153.117536
novel_miR_37	101.4728053	361.8085258	147.0227885	276.986646	401.3232415	410.8514976	281.9904398	155.5532511	175.0267688
novel_miR_37	316.0153079	652.5475197	509.6790002	772.3039423	918.9177025	658.6169045	515.8361704	429.6232648	401.531999
novel_miR_38	318.9145309	594.3997209	290.778404	449.6959664	903.9149645	812.2941822	890.6771209	803.6917972	720.6984598
novel_miR_39	576.9453786	239.0520617	686.1063465	42.3626635	22.504107	56.45287753	34.38907803	37.03648835	102.9569228
novel_miR_40	316.0153079	568.5562548	382.2592502	700.613281	765.139638	975.3802729	835.6545961	755.5443623	803.063998
novel_miR_41	423.2865592	542.7127886	267.9081924	208.5546511	390.071188	454.7592912	395.4743973	333.3283951	339.7578453
novel_miR_41	411.6896672	516.8693225	463.9385771	1635.850545	1425.26011	859.3382468	804.7044259	829.617339	638.3329215
novel_miR_41	171.0541575	135.6781972	212.3662501	195.5199854	101.2684815	166.2223616	244.162454	196.2933882	216.2095379
novel_miR_42	408.7904442	510.408456	454.1370579	1622.815879	1406.506687	856.2019759	787.5098869	837.0246367	638.3329215
novel_miR_42	287.0230778	368.2693923	186.2288655	492.0586299	705.128686	539.4386075	464.2525534	496.2889439	267.6879993
novel_miR_43	173.9533805	277.8172609	81.67932696	270.4693131	337.561605	301.0820135	299.1849789	388.8831277	308.8707685
novel_miR_43	1295.952685	1279.251573	1368.94552	1199.189244	1297.736837	2007.213423	2335.018398	1796.269685	2388.60061
novel_miR_43	63.78290618	200.2868625	137.2212693	185.7439861	191.2849095	197.5850714	92.85051068	99.99851854	82.36553826
novel_miR_44	1110.402412	1369.703705	901.7397697	811.4079394	1046.440975	1041.241963	1017.91671	1099.983704	1008.977844
novel_miR_45	81.17824423	122.7564641	49.00759618	162.9333212	195.035594	250.9016779	361.0853193	292.588258	123.5483074
novel_miR_45	1725.03769	1977.025159	1502.899616	1000.410592	1368.999842	1555.590403	1815.74332	1855.528066	1523.762458

novel_miR_45	1203.177548	1318.016772	931.1443274	1815.077198	2389.186026	2430.610005	2386.602015	2448.11188	2234.165225
novel_miR_46	371.1005451	471.6432568	329.9844809	319.3493095	390.071188	435.9416654	550.2252485	570.3619206	555.9673832
novel_miR_48	1116.200858	814.069183	604.4270195	1081.877253	1106.451927	1332.915164	2114.928299	2996.251907	2913.680916
novel_miR_48	191966.253	83881.43018	180681.2056	11551.97247	10651.94398	13946.99702	12957.8046	14840.52088	58294.2097
novel_miR_48	2139.62658	3760.224321	1620.517847	1922.61319	2659.23531	2653.285244	3332.301661	3359.209493	3232.847377
novel_miR_49	63.78290618	174.4433963	71.87780773	205.2959847	288.8027065	188.1762584	123.8006809	151.8496022	102.9569228
novel_miR_50	231.9378407	303.660727	267.9081924	658.2506175	547.599937	498.6670848	481.4470924	362.9575858	216.2095379
novel_miR_50	1942.479416	1473.077569	2646.410194	1906.319858	967.676601	1317.233809	1217.373362	1133.316543	2491.557532
novel_miR_53	50530.55781	28130.61288	43002.53206	6670.490169	5044.670652	7276.148659	6086.866811	4459.193197	27561.56824
novel_miR_71	46.38756813	32.30433266	19.60303847	110.7946584	82.515059	75.27050337	116.9228653	129.6277092	154.4353842
novel_miR_73	118.8681433	142.1390637	39.20607694	78.20799416	105.019166	128.5871099	185.7010214	155.5532511	113.2526151
novel_miR_83	817.5808883	788.2257168	754.7169811	1160.085247	757.638269	1050.650776	1396.196568	1674.049273	1389.918458
novel_miR_87	1113.301635	1382.625438	905.0069427	821.1839387	1042.690291	1047.514505	1017.91671	1111.09465	1008.977844
novel_miR_97	1962.773977	2345.294551	1672.792616	1306.725236	1920.350464	1819.037165	2101.172668	2262.929438	1832.633226
novel_miR_99	313.1160849	555.6345217	336.5188271	599.5946219	1267.731361	655.4806335	677.4648372	737.0261181	576.5587678
