# Supplementary material for: RNA sequencing-based exploration of the effects of far-red light on microRNAs involved in the shade-avoidance response of D. officinale
Source: PeerJ. 2023 Mar 20;11:e15001. doi: 10.7717/peerj.15001 (PMC10035421; doi:10.7717/peerj.15001)
Supplement: Table S7 [file peerj-11-15001-s007.pdf]

Table S7 Interaction miRNAs and mRNAs of FR2-CK

miRNA ID	log_2FC	Targets
novel_miR_189	-3.824935034	gene-MA16_Dca023326
novel_miR_189	-3.824935034	gene-MA16_Dca011459
novel_miR_189	-3.824935034	gene-MA16_Dca010804
novel_miR_189	-3.824935034	gene-MA16_Dca024400
novel_miR_189	-3.824935034	gene-MA16_Dca022787
novel_miR_189	-3.824935034	gene-MA16_Dca025882
novel_miR_189	-3.824935034	gene-MA16_Dca015722
novel_miR_189	-3.824935034	gene-MA16_Dca014786
novel_miR_189	-3.824935034	gene-MA16_Dca026995
novel_miR_189	-3.824935034	gene-MA16_Dca004836
novel_miR_189	-3.824935034	gene-MA16_Dca001649
novel_miR_189	-3.824935034	gene-MA16_Dca018219
novel_miR_189	-3.824935034	gene-MA16_Dca010516
novel_miR_189	-3.824935034	gene-MA16_Dca024704
novel_miR_189	-3.824935034	gene-MA16_Dca024902
novel_miR_189	-3.824935034	gene-MA16_Dca001622
novel_miR_189	-3.824935034	gene-MA16_Dca021278
novel_miR_189	-3.824935034	gene-MA16_Dca022838
novel_miR_189	-3.824935034	gene-MA16_Dca022812
novel_miR_189	-3.824935034	gene-MA16_Dca005118
novel_miR_189	-3.824935034	gene-MA16_Dca011344
novel_miR_189	-3.824935034	gene-MA16_Dca017709
novel_miR_189	-3.824935034	gene-MA16_Dca018325
novel_miR_189	-3.824935034	gene-MA16_Dca003816
novel_miR_189	-3.824935034	gene-MA16_Dca014554
novel_miR_189	-3.824935034	gene-MA16_Dca018212
novel_miR_189	-3.824935034	gene-MA16_Dca010241
novel_miR_189	-3.824935034	gene-MA16_Dca005791
novel_miR_189	-3.824935034	gene-MA16_Dca002499
novel_miR_189	-3.824935034	gene-MA16_Dca013892
novel_miR_189	-3.824935034	gene-MA16_Dca000024
novel_miR_189	-3.824935034	gene-MA16_Dca009944
novel_miR_189	-3.824935034	gene-MA16_Dca003956
novel_miR_189	-3.824935034	gene-MA16_Dca028126
novel_miR_189	-3.824935034	gene-MA16_Dca024385
novel_miR_189	-3.824935034	gene-MA16_Dca014761
novel_miR_189	-3.824935034	gene-MA16_Dca021441
novel_miR_189	-3.824935034	gene-MA16_Dca009034
novel_miR_189	-3.824935034	gene-MA16_Dca003052
novel_miR_189	-3.824935034	gene-MA16_Dca027145
novel_miR_189	-3.824935034	gene-MA16_Dca014804
novel_miR_189	-3.824935034	gene-MA16_Dca021649
novel_miR_189	-3.824935034	gene-MA16_Dca010408
novel_miR_189	-3.824935034	gene-MA16_Dca011938

novel_miR_189	-3.824935034	gene-MA16_Dca003122
novel_miR_189	-3.824935034	gene-MA16_Dca010402
novel_miR_189	-3.824935034	gene-MA16_Dca016865
novel_miR_189	-3.824935034	gene-MA16_Dca011525
novel_miR_189	-3.824935034	gene-MA16_Dca001400
novel_miR_189	-3.824935034	gene-MA16_Dca007799
novel_miR_189	-3.824935034	gene-MA16_Dca010456
novel_miR_189	-3.824935034	gene-MA16_Dca013722
novel_miR_189	-3.824935034	gene-MA16_Dca005175
novel_miR_189	-3.824935034	gene-MA16_Dca008000
novel_miR_189	-3.824935034	gene-MA16_Dca013243
novel_miR_189	-3.824935034	gene-MA16_Dca026494
novel_miR_484	-4.204864689	gene-MA16_Dca013703
novel_miR_484	-4.204864689	gene-MA16_Dca011847
novel_miR_484	-4.204864689	gene-MA16_Dca028665
novel_miR_484	-4.204864689	gene-MA16_Dca022384
novel_miR_484	-4.204864689	gene-MA16_Dca018928
novel_miR_484	-4.204864689	gene-MA16_Dca013338
novel_miR_484	-4.204864689	gene-MA16_Dca009144
novel_miR_484	-4.204864689	gene-MA16_Dca015180
novel_miR_484	-4.204864689	gene-MA16_Dca027734
novel_miR_484	-4.204864689	gene-MA16_Dca019875
novel_miR_484	-4.204864689	gene-MA16_Dca016458
novel_miR_484	-4.204864689	gene-MA16_Dca016490
novel_miR_484	-4.204864689	gene-MA16_Dca019561
novel_miR_484	-4.204864689	gene-MA16_Dca006494
novel_miR_484	-4.204864689	gene-MA16_Dca015083
novel_miR_484	-4.204864689	gene-MA16_Dca011199
novel_miR_484	-4.204864689	gene-MA16_Dca022649
novel_miR_484	-4.204864689	gene-MA16_Dca014915
novel_miR_484	-4.204864689	gene-MA16_Dca010212
novel_miR_484	-4.204864689	gene-MA16_Dca008125
novel_miR_484	-4.204864689	gene-MA16_Dca017780
novel_miR_484	-4.204864689	gene-MA16_Dca019194
novel_miR_484	-4.204864689	gene-MA16_Dca008674
novel_miR_484	-4.204864689	gene-MA16_Dca016409
novel_miR_484	-4.204864689	gene-MA16_Dca017451
novel_miR_484	-4.204864689	gene-MA16_Dca021955
novel_miR_484	-4.204864689	gene-MA16_Dca021243
novel_miR_484	-4.204864689	gene-MA16_Dca027391
novel_miR_484	-4.204864689	gene-MA16_Dca018115
novel_miR_484	-4.204864689	gene-MA16_Dca000965
novel_miR_484	-4.204864689	gene-MA16_Dca006776
novel_miR_484	-4.204864689	gene-MA16_Dca007667
novel_miR_484	-4.204864689	gene-MA16_Dca003224
novel_miR_484	-4.204864689	gene-MA16_Dca004503

novel_miR_484	-4.204864689	gene-MA16_Dca023412
novel_miR_484	-4.204864689	gene-MA16_Dca012030
novel_miR_484	-4.204864689	gene-MA16_Dca020389
novel_miR_484	-4.204864689	gene-MA16_Dca028340
novel_miR_484	-4.204864689	gene-MA16_Dca002133
novel_miR_484	-4.204864689	gene-MA16_Dca007417
novel_miR_484	-4.204864689	gene-MA16_Dca012435
novel_miR_484	-4.204864689	gene-MA16_Dca015045
novel_miR_484	-4.204864689	gene-MA16_Dca019567
novel_miR_484	-4.204864689	gene-MA16_Dca001581
novel_miR_484	-4.204864689	gene-MA16_Dca022185
novel_miR_484	-4.204864689	gene-MA16_Dca009281
novel_miR_484	-4.204864689	gene-MA16_Dca028824
novel_miR_484	-4.204864689	gene-MA16_Dca011516
novel_miR_484	-4.204864689	gene-MA16_Dca011253
novel_miR_484	-4.204864689	gene-MA16_Dca015359
novel_miR_484	-4.204864689	gene-MA16_Dca002926
novel_miR_484	-4.204864689	gene-MA16_Dca005762
novel_miR_484	-4.204864689	gene-MA16_Dca017160
novel_miR_484	-4.204864689	gene-MA16_Dca022638
novel_miR_484	-4.204864689	gene-MA16_Dca015933
novel_miR_484	-4.204864689	gene-MA16_Dca016882
novel_miR_484	-4.204864689	gene-MA16_Dca025576
novel_miR_484	-4.204864689	gene-MA16_Dca008661
novel_miR_484	-4.204864689	gene-MA16_Dca010144
novel_miR_484	-4.204864689	gene-MA16_Dca008955
novel_miR_484	-4.204864689	gene-MA16_Dca013055
novel_miR_484	-4.204864689	gene-MA16_Dca016304
novel_miR_484	-4.204864689	gene-MA16_Dca024277
novel_miR_484	-4.204864689	gene-MA16_Dca019975
novel_miR_484	-4.204864689	gene-MA16_Dca003651
novel_miR_484	-4.204864689	gene-MA16_Dca017446
novel_miR_484	-4.204864689	gene-MA16_Dca013631
novel_miR_484	-4.204864689	gene-MA16_Dca000685
novel_miR_484	-4.204864689	gene-MA16_Dca014673
novel_miR_484	-4.204864689	gene-MA16_Dca018194
novel_miR_484	-4.204864689	gene-MA16_Dca006033
novel_miR_484	-4.204864689	gene-MA16_Dca001538
novel_miR_484	-4.204864689	gene-MA16_Dca026681
novel_miR_484	-4.204864689	gene-MA16_Dca025504
novel_miR_484	-4.204864689	gene-MA16_Dca006006
novel_miR_484	-4.204864689	gene-MA16_Dca019891
novel_miR_484	-4.204864689	gene-MA16_Dca020179
novel_miR_484	-4.204864689	gene-MA16_Dca015057
novel_miR_484	-4.204864689	gene-MA16_Dca012781
novel_miR_484	-4.204864689	gene-MA16_Dca004540

novel_miR_484	-4.204864689	gene-MA16_Dca018330
novel_miR_484	-4.204864689	gene-MA16_Dca001333
novel_miR_484	-4.204864689	gene-MA16_Dca014007
novel_miR_484	-4.204864689	gene-MA16_Dca028410
novel_miR_484	-4.204864689	gene-MA16_Dca028352
novel_miR_484	-4.204864689	gene-MA16_Dca010058
novel_miR_484	-4.204864689	gene-MA16_Dca000100
novel_miR_484	-4.204864689	gene-MA16_Dca021773
novel_miR_484	-4.204864689	gene-MA16_Dca001992
novel_miR_484	-4.204864689	gene-MA16_Dca008877
novel_miR_484	-4.204864689	gene-MA16_Dca028562
novel_miR_484	-4.204864689	gene-MA16_Dca021141
novel_miR_484	-4.204864689	gene-MA16_Dca024139
novel_miR_484	-4.204864689	gene-MA16_Dca005319
novel_miR_484	-4.204864689	gene-MA16_Dca011443
novel_miR_484	-4.204864689	gene-MA16_Dca022957
novel_miR_484	-4.204864689	gene-MA16_Dca014398
novel_miR_484	-4.204864689	gene-MA16_Dca003585
novel_miR_484	-4.204864689	gene-MA16_Dca008889
novel_miR_484	-4.204864689	gene-MA16_Dca026047
novel_miR_484	-4.204864689	gene-MA16_Dca019039
novel_miR_484	-4.204864689	gene-MA16_Dca012296
novel_miR_484	-4.204864689	gene-MA16_Dca011298
novel_miR_484	-4.204864689	gene-MA16_Dca023407
novel_miR_484	-4.204864689	gene-MA16_Dca017598
novel_miR_484	-4.204864689	gene-MA16_Dca023855
novel_miR_484	-4.204864689	gene-MA16_Dca004340
novel_miR_484	-4.204864689	gene-MA16_Dca007240
novel_miR_484	-4.204864689	gene-MA16_Dca005346
novel_miR_484	-4.204864689	gene-MA16_Dca009990
novel_miR_484	-4.204864689	gene-MA16_Dca014237
novel_miR_484	-4.204864689	gene-MA16_Dca024325
novel_miR_484	-4.204864689	gene-MA16_Dca022905
novel_miR_484	-4.204864689	gene-MA16_Dca019790
novel_miR_484	-4.204864689	gene-MA16_Dca010093
novel_miR_484	-4.204864689	gene-MA16_Dca015921
novel_miR_484	-4.204864689	gene-MA16_Dca017338
novel_miR_484	-4.204864689	gene-MA16_Dca027164
novel_miR_484	-4.204864689	gene-MA16_Dca022795
novel_miR_484	-4.204864689	gene-MA16_Dca012251
novel_miR_53	-3.191236942	gene-MA16_Dca005089
novel_miR_53	-3.191236942	gene-MA16_Dca020713
novel_miR_53	-3.191236942	gene-MA16_Dca022778
novel_miR_53	-3.191236942	gene-MA16_Dca001003
novel_miR_53	-3.191236942	gene-MA16_Dca007171
novel_miR_53	-3.191236942	gene-MA16_Dca014115

novel_miR_53	-3.191236942	gene-MA16_Dca000122
novel_miR_53	-3.191236942	gene-MA16_Dca012806
novel_miR_53	-3.191236942	gene-MA16_Dca003261
novel_miR_53	-3.191236942	gene-MA16_Dca000505
novel_miR_53	-3.191236942	gene-MA16_Dca009084
novel_miR_53	-3.191236942	gene-MA16_Dca012781
novel_miR_53	-3.191236942	gene-MA16_Dca010665
novel_miR_53	-3.191236942	gene-MA16_Dca018178
novel_miR_53	-3.191236942	gene-MA16_Dca011106
novel_miR_53	-3.191236942	gene-MA16_Dca023559
novel_miR_53	-3.191236942	gene-MA16_Dca017541
novel_miR_53	-3.191236942	gene-MA16_Dca002207
novel_miR_53	-3.191236942	gene-MA16_Dca017663
novel_miR_53	-3.191236942	gene-MA16_Dca017043
novel_miR_53	-3.191236942	gene-MA16_Dca010639
novel_miR_53	-3.191236942	gene-MA16_Dca000121
novel_miR_53	-3.191236942	gene-MA16_Dca023432
novel_miR_53	-3.191236942	gene-MA16_Dca015909
novel_miR_53	-3.191236942	gene-MA16_Dca002218
novel_miR_53	-3.191236942	gene-MA16_Dca011246
novel_miR_53	-3.191236942	gene-MA16_Dca003197
novel_miR_53	-3.191236942	gene-MA16_Dca012420
novel_miR_53	-3.191236942	gene-MA16_Dca001258
novel_miR_53	-3.191236942	gene-MA16_Dca006414
novel_miR_53	-3.191236942	gene-MA16_Dca021885
novel_miR_53	-3.191236942	gene-MA16_Dca023972
novel_miR_53	-3.191236942	gene-MA16_Dca014156
novel_miR_53	-3.191236942	gene-MA16_Dca007605
novel_miR_53	-3.191236942	gene-MA16_Dca022196
novel_miR_53	-3.191236942	gene-MA16_Dca015291
novel_miR_262	-4.159399363	gene-MA16_Dca011294
novel_miR_262	-4.159399363	gene-MA16_Dca002242
novel_miR_262	-4.159399363	gene-MA16_Dca001955
novel_miR_262	-4.159399363	gene-MA16_Dca010163
novel_miR_262	-4.159399363	gene-MA16_Dca010619
novel_miR_262	-4.159399363	gene-MA16_Dca020583
novel_miR_262	-4.159399363	gene-MA16_Dca012128
novel_miR_262	-4.159399363	gene-MA16_Dca026927
novel_miR_262	-4.159399363	gene-MA16_Dca006631
novel_miR_262	-4.159399363	gene-MA16_Dca004093
novel_miR_262	-4.159399363	gene-MA16_Dca002377
novel_miR_262	-4.159399363	gene-MA16_Dca001199
novel_miR_262	-4.159399363	gene-MA16_Dca006179
novel_miR_262	-4.159399363	gene-MA16_Dca024442
novel_miR_262	-4.159399363	gene-MA16_Dca001936
novel_miR_262	-4.159399363	gene-MA16_Dca001785

novel_miR_262	-4.159399363	gene-MA16_Dca001920
novel_miR_262	-4.159399363	gene-MA16_Dca024581
novel_miR_262	-4.159399363	gene-MA16_Dca018172
novel_miR_262	-4.159399363	gene-MA16_Dca012093
novel_miR_262	-4.159399363	gene-MA16_Dca000754
novel_miR_262	-4.159399363	gene-MA16_Dca018548
novel_miR_262	-4.159399363	gene-MA16_Dca013497
novel_miR_262	-4.159399363	gene-MA16_Dca023379
novel_miR_262	-4.159399363	gene-MA16_Dca019943
novel_miR_262	-4.159399363	gene-MA16_Dca006898
novel_miR_262	-4.159399363	gene-MA16_Dca019207
novel_miR_262	-4.159399363	gene-MA16_Dca019153
novel_miR_262	-4.159399363	gene-MA16_Dca010222
novel_miR_262	-4.159399363	gene-MA16_Dca021850
novel_miR_262	-4.159399363	gene-MA16_Dca000152
novel_miR_262	-4.159399363	gene-MA16_Dca022211
novel_miR_262	-4.159399363	gene-MA16_Dca010315
novel_miR_262	-4.159399363	gene-MA16_Dca023840
novel_miR_262	-4.159399363	gene-MA16_Dca006059
novel_miR_262	-4.159399363	gene-MA16_Dca018725
novel_miR_262	-4.159399363	gene-MA16_Dca023691
novel_miR_262	-4.159399363	gene-MA16_Dca005799
novel_miR_262	-4.159399363	gene-MA16_Dca021863
novel_miR_262	-4.159399363	gene-MA16_Dca022508
novel_miR_262	-4.159399363	gene-MA16_Dca012576
novel_miR_262	-4.159399363	gene-MA16_Dca003694
novel_miR_262	-4.159399363	gene-MA16_Dca004139
novel_miR_262	-4.159399363	gene-MA16_Dca014631
novel_miR_262	-4.159399363	gene-MA16_Dca021860
novel_miR_262	-4.159399363	gene-MA16_Dca018722
novel_miR_262	-4.159399363	gene-MA16_Dca008028
novel_miR_262	-4.159399363	gene-MA16_Dca004158
novel_miR_262	-4.159399363	gene-MA16_Dca023002
novel_miR_262	-4.159399363	gene-MA16_Dca004133
novel_miR_262	-4.159399363	gene-MA16_Dca025782
novel_miR_262	-4.159399363	gene-MA16_Dca024909
novel_miR_262	-4.159399363	gene-MA16_Dca022287
novel_miR_262	-4.159399363	gene-MA16_Dca016598
novel_miR_262	-4.159399363	gene-MA16_Dca014260
novel_miR_262	-4.159399363	gene-MA16_Dca001930
novel_miR_262	-4.159399363	gene-MA16_Dca014266
novel_miR_262	-4.159399363	gene-MA16_Dca017977
novel_miR_262	-4.159399363	gene-MA16_Dca007307
novel_miR_262	-4.159399363	gene-MA16_Dca005596
novel_miR_262	-4.159399363	gene-MA16_Dca016354
novel_miR_262	-4.159399363	gene-MA16_Dca013418

novel_miR_262	-4.159399363	gene-MA16_Dca005154
novel_miR_262	-4.159399363	gene-MA16_Dca011751
novel_miR_262	-4.159399363	gene-MA16_Dca015263
novel_miR_262	-4.159399363	gene-MA16_Dca011523
novel_miR_262	-4.159399363	gene-MA16_Dca011783
novel_miR_262	-4.159399363	gene-MA16_Dca005151
novel_miR_262	-4.159399363	gene-MA16_Dca011967
novel_miR_262	-4.159399363	gene-MA16_Dca008222
novel_miR_262	-4.159399363	gene-MA16_Dca007694
novel_miR_262	-4.159399363	gene-MA16_Dca000885
novel_miR_262	-4.159399363	gene-MA16_Dca006666
novel_miR_262	-4.159399363	gene-MA16_Dca020166
novel_miR_262	-4.159399363	gene-MA16_Dca018345
novel_miR_262	-4.159399363	gene-MA16_Dca002241
novel_miR_262	-4.159399363	gene-MA16_Dca019707
novel_miR_262	-4.159399363	gene-MA16_Dca012938
novel_miR_262	-4.159399363	gene-MA16_Dca020801
novel_miR_262	-4.159399363	gene-MA16_Dca007955
novel_miR_262	-4.159399363	gene-MA16_Dca022302
novel_miR_262	-4.159399363	gene-MA16_Dca005617
novel_miR_262	-4.159399363	gene-MA16_Dca000619
novel_miR_262	-4.159399363	gene-MA16_Dca002697
novel_miR_262	-4.159399363	gene-MA16_Dca024737
novel_miR_262	-4.159399363	gene-MA16_Dca019222
novel_miR_262	-4.159399363	gene-MA16_Dca014751
novel_miR_262	-4.159399363	gene-MA16_Dca023601
novel_miR_262	-4.159399363	gene-MA16_Dca004403
novel_miR_262	-4.159399363	gene-MA16_Dca024281
novel_miR_262	-4.159399363	gene-MA16_Dca015409
novel_miR_262	-4.159399363	gene-MA16_Dca025223
novel_miR_262	-4.159399363	gene-MA16_Dca009226
novel_miR_262	-4.159399363	gene-MA16_Dca013322
novel_miR_262	-4.159399363	gene-MA16_Dca016956
novel_miR_262	-4.159399363	gene-MA16_Dca011603
novel_miR_262	-4.159399363	gene-MA16_Dca007949
novel_miR_262	-4.159399363	gene-MA16_Dca020207
novel_miR_262	-4.159399363	gene-MA16_Dca021446
novel_miR_262	-4.159399363	gene-MA16_Dca024988
novel_miR_262	-4.159399363	gene-MA16_Dca003177
novel_miR_262	-4.159399363	gene-MA16_Dca027492
novel_miR_262	-4.159399363	gene-MA16_Dca011959
novel_miR_262	-4.159399363	gene-MA16_Dca011627
novel_miR_262	-4.159399363	gene-MA16_Dca020910
novel_miR_262	-4.159399363	gene-MA16_Dca004692
novel_miR_262	-4.159399363	gene-MA16_Dca010708
novel_miR_262	-4.159399363	gene-MA16_Dca012099

novel_miR_262	-4.159399363	gene-MA16_Dca011382
novel_miR_262	-4.159399363	gene-MA16_Dca025262
novel_miR_262	-4.159399363	gene-MA16_Dca028471
novel_miR_262	-4.159399363	gene-MA16_Dca003911
novel_miR_390	-4.159397226	gene-MA16_Dca014079
novel_miR_390	-4.159397226	gene-MA16_Dca010045
novel_miR_390	-4.159397226	gene-MA16_Dca016601
novel_miR_390	-4.159397226	gene-MA16_Dca028500
novel_miR_390	-4.159397226	gene-MA16_Dca008125
novel_miR_390	-4.159397226	gene-MA16_Dca010799
novel_miR_390	-4.159397226	gene-MA16_Dca028410
novel_miR_390	-4.159397226	gene-MA16_Dca018794
novel_miR_390	-4.159397226	gene-MA16_Dca005769
novel_miR_390	-4.159397226	gene-MA16_Dca003585
novel_miR_390	-4.159397226	gene-MA16_Dca021544
novel_miR_390	-4.159397226	gene-MA16_Dca013966
novel_miR_390	-4.159397226	gene-MA16_Dca014033
novel_miR_390	-4.159397226	gene-MA16_Dca027734
novel_miR_390	-4.159397226	gene-MA16_Dca014296
novel_miR_390	-4.159397226	gene-MA16_Dca024277
novel_miR_390	-4.159397226	gene-MA16_Dca011847
novel_miR_390	-4.159397226	gene-MA16_Dca013703
novel_miR_390	-4.159397226	gene-MA16_Dca014007
novel_miR_390	-4.159397226	gene-MA16_Dca014915
novel_miR_390	-4.159397226	gene-MA16_Dca004540
novel_miR_390	-4.159397226	gene-MA16_Dca011199
novel_miR_390	-4.159397226	gene-MA16_Dca022649
novel_miR_390	-4.159397226	gene-MA16_Dca018160
novel_miR_390	-4.159397226	gene-MA16_Dca002338
novel_miR_390	-4.159397226	gene-MA16_Dca015057
novel_miR_390	-4.159397226	gene-MA16_Dca016490
novel_miR_390	-4.159397226	gene-MA16_Dca025504
novel_miR_390	-4.159397226	gene-MA16_Dca016699
novel_miR_390	-4.159397226	gene-MA16_Dca014673
novel_miR_390	-4.159397226	gene-MA16_Dca022905
novel_miR_390	-4.159397226	gene-MA16_Dca015359
novel_miR_390	-4.159397226	gene-MA16_Dca011253
novel_miR_390	-4.159397226	gene-MA16_Dca014237
novel_miR_390	-4.159397226	gene-MA16_Dca021241
novel_miR_390	-4.159397226	gene-MA16_Dca000483
novel_miR_390	-4.159397226	gene-MA16_Dca009990
novel_miR_390	-4.159397226	gene-MA16_Dca005346
novel_miR_390	-4.159397226	gene-MA16_Dca022795
novel_miR_390	-4.159397226	gene-MA16_Dca008661
novel_miR_390	-4.159397226	gene-MA16_Dca017160
novel_miR_390	-4.159397226	gene-MA16_Dca022638

novel_miR_390	-4.159397226	gene-MA16_Dca013961
novel_miR_390	-4.159397226	gene-MA16_Dca010093
novel_miR_390	-4.159397226	gene-MA16_Dca002089
novel_miR_390	-4.159397226	gene-MA16_Dca021165
novel_miR_390	-4.159397226	gene-MA16_Dca004503
novel_miR_390	-4.159397226	gene-MA16_Dca007957
novel_miR_390	-4.159397226	gene-MA16_Dca020534
novel_miR_390	-4.159397226	gene-MA16_Dca004340
novel_miR_390	-4.159397226	gene-MA16_Dca017598
novel_miR_390	-4.159397226	gene-MA16_Dca012435
novel_miR_390	-4.159397226	gene-MA16_Dca007417
novel_miR_390	-4.159397226	gene-MA16_Dca010932
novel_miR_390	-4.159397226	gene-MA16_Dca023407
novel_miR_390	-4.159397226	gene-MA16_Dca002781
novel_miR_390	-4.159397226	gene-MA16_Dca008350
novel_miR_390	-4.159397226	gene-MA16_Dca002133
novel_miR_390	-4.159397226	gene-MA16_Dca015811
novel_miR_390	-4.159397226	gene-MA16_Dca023276
miR399b_1	-2.103094579	gene-MA16_Dca015394
miR399b_1	-2.103094579	gene-MA16_Dca002693
miR399b_1	-2.103094579	gene-MA16_Dca009131
miR399b_1	-2.103094579	gene-MA16_Dca010687
miR399b_1	-2.103094579	gene-MA16_Dca028429
miR399b_1	-2.103094579	gene-MA16_Dca015044
miR399b_1	-2.103094579	gene-MA16_Dca008850
miR399b_1	-2.103094579	gene-MA16_Dca013506
miR399b_1	-2.103094579	gene-MA16_Dca001351
miR399b_1	-2.103094579	gene-MA16_Dca001177
miR399b_1	-2.103094579	gene-MA16_Dca011813
miR399b_1	-2.103094579	gene-MA16_Dca010182
miR399b_1	-2.103094579	gene-MA16_Dca010688
miR399b_1	-2.103094579	gene-MA16_Dca026575
miR395b	-2.674362584	gene-MA16_Dca015429
miR395b	-2.674362584	gene-MA16_Dca003297
miR395b	-2.674362584	gene-MA16_Dca007732
miR395b	-2.674362584	gene-MA16_Dca011968
miR395b	-2.674362584	gene-MA16_Dca003285
miR395b	-2.674362584	gene-MA16_Dca007731
miR395b	-2.674362584	gene-MA16_Dca004386
miR395b	-2.674362584	gene-MA16_Dca009355
miR395b	-2.674362584	gene-MA16_Dca019636
miR395b	-2.674362584	gene-MA16_Dca001411
miR395b	-2.674362584	gene-MA16_Dca025511
miR395m	-2.035107482	gene-MA16_Dca017889
miR395m	-2.035107482	gene-MA16_Dca007732
miR395m	-2.035107482	gene-MA16_Dca021033

miR395m	-2.035107482	gene-MA16_Dca007646
miR395m	-2.035107482	gene-MA16_Dca026271
miR395m	-2.035107482	gene-MA16_Dca003297
miR395m	-2.035107482	gene-MA16_Dca025511
miR395m	-2.035107482	gene-MA16_Dca001411
miR395m	-2.035107482	gene-MA16_Dca007731
miR395m	-2.035107482	gene-MA16_Dca004386
miR395m	-2.035107482	gene-MA16_Dca009355
miR395m	-2.035107482	gene-MA16_Dca003285
miR395m	-2.035107482	gene-MA16_Dca005635
miR395m	-2.035107482	gene-MA16_Dca011968
miR528_5p	-2.674602419	gene-MA16_Dca020077
miR528_5p	-2.674602419	gene-MA16_Dca010331
miR528_5p	-2.674602419	gene-MA16_Dca002770
miR528_5p	-2.674602419	gene-MA16_Dca004667
miR528_5p	-2.674602419	gene-MA16_Dca000891
miR528_5p	-2.674602419	gene-MA16_Dca027730
miR528_5p	-2.674602419	gene-MA16_Dca012338
miR528_5p	-2.674602419	gene-MA16_Dca015008
miR528_5p	-2.674602419	gene-MA16_Dca021334
miR528_5p	-2.674602419	gene-MA16_Dca013643
miR528_5p	-2.674602419	gene-MA16_Dca022587
miR528_5p	-2.674602419	gene-MA16_Dca014181
miR528_5p	-2.674602419	gene-MA16_Dca015948
miR528_5p	-2.674602419	gene-MA16_Dca013418
miR528_5p	-2.674602419	gene-MA16_Dca028734
miR528_5p	-2.674602419	gene-MA16_Dca020299
miR528_5p	-2.674602419	gene-MA16_Dca009198
miR528_5p	-2.674602419	gene-MA16_Dca021947
miR528_5p	-2.674602419	gene-MA16_Dca026005
miR528_5p	-2.674602419	gene-MA16_Dca011035
miR528_5p	-2.674602419	gene-MA16_Dca010931
miR528_5p	-2.674602419	gene-MA16_Dca019975
miR528_5p	-2.674602419	gene-MA16_Dca001951
miR528_5p	-2.674602419	gene-MA16_Dca000698
miR528_5p	-2.674602419	gene-MA16_Dca011273
miR528_5p	-2.674602419	gene-MA16_Dca002672
miR528_5p	-2.674602419	gene-MA16_Dca026199
miR528_5p	-2.674602419	gene-MA16_Dca020850
miR528_5p	-2.674602419	gene-MA16_Dca003559
miR528_5p	-2.674602419	gene-MA16_Dca026841
miR528_5p	-2.674602419	gene-MA16_Dca026559
miR399e_5p_2	1.249181458	gene-MA16_Dca007637
miR7532a	1.168860011	gene-MA16_Dca009494
novel_miR_264	0.897247319	gene-MA16_Dca017476
novel_miR_264	0.897247319	gene-MA16_Dca018659

novel_miR_264	0.897247319	gene-MA16_Dca019290
novel_miR_264	0.897247319	gene-MA16_Dca015518
novel_miR_204	1.10059286	gene-MA16_Dca028017
novel_miR_204	1.10059286	gene-MA16_Dca010066
novel_miR_204	1.10059286	gene-MA16_Dca028670
novel_miR_204	1.10059286	gene-MA16_Dca025840
novel_miR_204	1.10059286	gene-MA16_Dca028781
novel_miR_204	1.10059286	gene-MA16_Dca009114
novel_miR_204	1.10059286	gene-MA16_Dca002142
novel_miR_204	1.10059286	gene-MA16_Dca010312
novel_miR_204	1.10059286	gene-MA16_Dca022915
novel_miR_204	1.10059286	gene-MA16_Dca025628
novel_miR_204	1.10059286	gene-MA16_Dca006665
novel_miR_204	1.10059286	gene-MA16_Dca002985
novel_miR_204	1.10059286	gene-MA16_Dca004156
novel_miR_204	1.10059286	gene-MA16_Dca015210
novel_miR_204	1.10059286	gene-MA16_Dca022493
novel_miR_204	1.10059286	gene-MA16_Dca020190
novel_miR_204	1.10059286	gene-MA16_Dca024943
novel_miR_204	1.10059286	gene-MA16_Dca027918
novel_miR_204	1.10059286	gene-MA16_Dca028785
novel_miR_204	1.10059286	gene-MA16_Dca028825
novel_miR_204	1.10059286	gene-MA16_Dca017679
novel_miR_204	1.10059286	gene-MA16_Dca009386
novel_miR_204	1.10059286	gene-MA16_Dca015874
novel_miR_204	1.10059286	gene-MA16_Dca021724
novel_miR_204	1.10059286	gene-MA16_Dca028230
novel_miR_204	1.10059286	gene-MA16_Dca017511
novel_miR_204	1.10059286	gene-MA16_Dca004131
novel_miR_204	1.10059286	gene-MA16_Dca010485
novel_miR_204	1.10059286	gene-MA16_Dca018453
novel_miR_204	1.10059286	gene-MA16_Dca019753
novel_miR_204	1.10059286	gene-MA16_Dca017455
novel_miR_204	1.10059286	gene-MA16_Dca000613
novel_miR_204	1.10059286	gene-MA16_Dca022492
novel_miR_204	1.10059286	gene-MA16_Dca004721
novel_miR_204	1.10059286	gene-MA16_Dca028949
novel_miR_204	1.10059286	gene-MA16_Dca003873
novel_miR_204	1.10059286	gene-MA16_Dca000766
novel_miR_204	1.10059286	gene-MA16_Dca003896
novel_miR_204	1.10059286	gene-MA16_Dca021252
novel_miR_420	1.066066015	gene-MA16_Dca024943
novel_miR_420	1.066066015	gene-MA16_Dca027918
novel_miR_420	1.066066015	gene-MA16_Dca028785
novel_miR_420	1.066066015	gene-MA16_Dca017679
novel_miR_420	1.066066015	gene-MA16_Dca028825

novel_miR_420	1.066066015	gene-MA16_Dca004156
novel_miR_420	1.066066015	gene-MA16_Dca015210
novel_miR_420	1.066066015	gene-MA16_Dca022493
novel_miR_420	1.066066015	gene-MA16_Dca020190
novel_miR_420	1.066066015	gene-MA16_Dca002142
novel_miR_420	1.066066015	gene-MA16_Dca010312
novel_miR_420	1.066066015	gene-MA16_Dca022915
novel_miR_420	1.066066015	gene-MA16_Dca025628
novel_miR_420	1.066066015	gene-MA16_Dca002985
novel_miR_420	1.066066015	gene-MA16_Dca006665
novel_miR_420	1.066066015	gene-MA16_Dca010066
novel_miR_420	1.066066015	gene-MA16_Dca028017
novel_miR_420	1.066066015	gene-MA16_Dca028670
novel_miR_420	1.066066015	gene-MA16_Dca025840
novel_miR_420	1.066066015	gene-MA16_Dca028781
novel_miR_420	1.066066015	gene-MA16_Dca009114
novel_miR_420	1.066066015	gene-MA16_Dca000766
novel_miR_420	1.066066015	gene-MA16_Dca003873
novel_miR_420	1.066066015	gene-MA16_Dca003896
novel_miR_420	1.066066015	gene-MA16_Dca021252
novel_miR_420	1.066066015	gene-MA16_Dca000613
novel_miR_420	1.066066015	gene-MA16_Dca017455
novel_miR_420	1.066066015	gene-MA16_Dca022492
novel_miR_420	1.066066015	gene-MA16_Dca004721
novel_miR_420	1.066066015	gene-MA16_Dca028949
novel_miR_420	1.066066015	gene-MA16_Dca004131
novel_miR_420	1.066066015	gene-MA16_Dca010485
novel_miR_420	1.066066015	gene-MA16_Dca018453
novel_miR_420	1.066066015	gene-MA16_Dca019753
novel_miR_420	1.066066015	gene-MA16_Dca009386
novel_miR_420	1.066066015	gene-MA16_Dca015874
novel_miR_420	1.066066015	gene-MA16_Dca021724
novel_miR_420	1.066066015	gene-MA16_Dca028230
novel_miR_420	1.066066015	gene-MA16_Dca017511
novel_miR_417	1.058538515	gene-MA16_Dca028949
novel_miR_417	1.058538515	gene-MA16_Dca004721
novel_miR_417	1.058538515	gene-MA16_Dca017455
novel_miR_417	1.058538515	gene-MA16_Dca000613
novel_miR_417	1.058538515	gene-MA16_Dca022492
novel_miR_417	1.058538515	gene-MA16_Dca000766
novel_miR_417	1.058538515	gene-MA16_Dca003873
novel_miR_417	1.058538515	gene-MA16_Dca021252
novel_miR_417	1.058538515	gene-MA16_Dca003896
novel_miR_417	1.058538515	gene-MA16_Dca021724
novel_miR_417	1.058538515	gene-MA16_Dca017511
novel_miR_417	1.058538515	gene-MA16_Dca028230

novel_miR_417	1.058538515	gene-MA16_Dca009386
novel_miR_417	1.058538515	gene-MA16_Dca015874
novel_miR_417	1.058538515	gene-MA16_Dca010485
novel_miR_417	1.058538515	gene-MA16_Dca018453
novel_miR_417	1.058538515	gene-MA16_Dca019753
novel_miR_417	1.058538515	gene-MA16_Dca004131
novel_miR_417	1.058538515	gene-MA16_Dca022493
novel_miR_417	1.058538515	gene-MA16_Dca015210
novel_miR_417	1.058538515	gene-MA16_Dca020190
novel_miR_417	1.058538515	gene-MA16_Dca004156
novel_miR_417	1.058538515	gene-MA16_Dca028785
novel_miR_417	1.058538515	gene-MA16_Dca017679
novel_miR_417	1.058538515	gene-MA16_Dca028825
novel_miR_417	1.058538515	gene-MA16_Dca024943
novel_miR_417	1.058538515	gene-MA16_Dca027918
novel_miR_417	1.058538515	gene-MA16_Dca009114
novel_miR_417	1.058538515	gene-MA16_Dca028781
novel_miR_417	1.058538515	gene-MA16_Dca028670
novel_miR_417	1.058538515	gene-MA16_Dca010066
novel_miR_417	1.058538515	gene-MA16_Dca028017
novel_miR_417	1.058538515	gene-MA16_Dca025840
novel_miR_417	1.058538515	gene-MA16_Dca025628
novel_miR_417	1.058538515	gene-MA16_Dca006665
novel_miR_417	1.058538515	gene-MA16_Dca002985
novel_miR_417	1.058538515	gene-MA16_Dca002142
novel_miR_417	1.058538515	gene-MA16_Dca022915
novel_miR_417	1.058538515	gene-MA16_Dca010312
novel_miR_192	0.798895311	gene-MA16_Dca010818
novel_miR_192	0.798895311	gene-MA16_Dca005939
novel_miR_451	-0.860759143	gene-MA16_Dca004841
novel_miR_451	-0.860759143	gene-MA16_Dca009490
novel_miR_451	-0.860759143	gene-MA16_Dca003193
novel_miR_1	-0.658885913	gene-MA16_Dca006730
novel_miR_1	-0.658885913	gene-MA16_Dca011627
novel_miR_1	-0.658885913	gene-MA16_Dca023929
novel_miR_1	-0.658885913	gene-MA16_Dca024649
novel_miR_1	-0.658885913	gene-MA16_Dca009490
novel_miR_1	-0.658885913	gene-MA16_Dca000440
novel_miR_1	-0.658885913	gene-MA16_Dca006916
novel_miR_1	-0.658885913	gene-MA16_Dca024036
novel_miR_1	-0.658885913	gene-MA16_Dca021780
novel_miR_445	-0.657446775	gene-MA16_Dca021780
novel_miR_445	-0.657446775	gene-MA16_Dca024036
novel_miR_445	-0.657446775	gene-MA16_Dca006916
novel_miR_445	-0.657446775	gene-MA16_Dca000440
novel_miR_445	-0.657446775	gene-MA16_Dca009490

novel_miR_445	-0.657446775	gene-MA16_Dca024649
novel_miR_445	-0.657446775	gene-MA16_Dca023929
novel_miR_445	-0.657446775	gene-MA16_Dca006730
novel_miR_445	-0.657446775	gene-MA16_Dca011627
miR530a	0.89183093	gene-MA16_Dca022268
miR530a	0.89183093	gene-MA16_Dca019882
miR530a	0.89183093	gene-MA16_Dca013836
miR530a	0.89183093	gene-MA16_Dca011594
miR530a	0.89183093	gene-MA16_Dca010385
novel_miR_191	-0.658852397	gene-MA16_Dca006916
novel_miR_191	-0.658852397	gene-MA16_Dca021780
novel_miR_191	-0.658852397	gene-MA16_Dca024036
novel_miR_191	-0.658852397	gene-MA16_Dca011627
novel_miR_191	-0.658852397	gene-MA16_Dca023929
novel_miR_191	-0.658852397	gene-MA16_Dca006730
novel_miR_191	-0.658852397	gene-MA16_Dca024649
novel_miR_191	-0.658852397	gene-MA16_Dca000440
novel_miR_191	-0.658852397	gene-MA16_Dca009490
novel_miR_215	-0.674212206	gene-MA16_Dca023237
novel_miR_215	-0.674212206	gene-MA16_Dca021366
novel_miR_215	-0.674212206	gene-MA16_Dca009502
novel_miR_215	-0.674212206	gene-MA16_Dca020878
novel_miR_215	-0.674212206	gene-MA16_Dca011564
novel_miR_215	-0.674212206	gene-MA16_Dca005261
novel_miR_215	-0.674212206	gene-MA16_Dca006086
novel_miR_215	-0.674212206	gene-MA16_Dca008215
novel_miR_215	-0.674212206	gene-MA16_Dca003056
miR396b_1	0.805439951	gene-MA16_Dca005596
miR396b_1	0.805439951	gene-MA16_Dca016354
miR396b_1	0.805439951	gene-MA16_Dca013418
miR396b_1	0.805439951	gene-MA16_Dca007307
miR396b_1	0.805439951	gene-MA16_Dca017977
miR396b_1	0.805439951	gene-MA16_Dca014266
miR396b_1	0.805439951	gene-MA16_Dca016598
miR396b_1	0.805439951	gene-MA16_Dca001930
miR396b_1	0.805439951	gene-MA16_Dca014260
miR396b_1	0.805439951	gene-MA16_Dca022287
miR396b_1	0.805439951	gene-MA16_Dca024909
miR396b_1	0.805439951	gene-MA16_Dca004133
miR396b_1	0.805439951	gene-MA16_Dca025782
miR396b_1	0.805439951	gene-MA16_Dca004158
miR396b_1	0.805439951	gene-MA16_Dca023002
miR396b_1	0.805439951	gene-MA16_Dca008028
miR396b_1	0.805439951	gene-MA16_Dca014631
miR396b_1	0.805439951	gene-MA16_Dca004139
miR396b_1	0.805439951	gene-MA16_Dca018722

miR396b_1	0.805439951	gene-MA16_Dca021860
miR396b_1	0.805439951	gene-MA16_Dca012576
miR396b_1	0.805439951	gene-MA16_Dca003694
miR396b_1	0.805439951	gene-MA16_Dca021863
miR396b_1	0.805439951	gene-MA16_Dca005799
miR396b_1	0.805439951	gene-MA16_Dca022508
miR396b_1	0.805439951	gene-MA16_Dca018725
miR396b_1	0.805439951	gene-MA16_Dca023691
miR396b_1	0.805439951	gene-MA16_Dca023840
miR396b_1	0.805439951	gene-MA16_Dca010315
miR396b_1	0.805439951	gene-MA16_Dca006059
miR396b_1	0.805439951	gene-MA16_Dca022211
miR396b_1	0.805439951	gene-MA16_Dca000152
miR396b_1	0.805439951	gene-MA16_Dca021850
miR396b_1	0.805439951	gene-MA16_Dca006898
miR396b_1	0.805439951	gene-MA16_Dca019153
miR396b_1	0.805439951	gene-MA16_Dca019207
miR396b_1	0.805439951	gene-MA16_Dca010222
miR396b_1	0.805439951	gene-MA16_Dca023379
miR396b_1	0.805439951	gene-MA16_Dca019943
miR396b_1	0.805439951	gene-MA16_Dca000754
miR396b_1	0.805439951	gene-MA16_Dca013497
miR396b_1	0.805439951	gene-MA16_Dca018548
miR396b_1	0.805439951	gene-MA16_Dca018172
miR396b_1	0.805439951	gene-MA16_Dca001920
miR396b_1	0.805439951	gene-MA16_Dca012093
miR396b_1	0.805439951	gene-MA16_Dca024581
miR396b_1	0.805439951	gene-MA16_Dca001936
miR396b_1	0.805439951	gene-MA16_Dca024442
miR396b_1	0.805439951	gene-MA16_Dca001785
miR396b_1	0.805439951	gene-MA16_Dca006179
miR396b_1	0.805439951	gene-MA16_Dca001199
miR396b_1	0.805439951	gene-MA16_Dca004093
miR396b_1	0.805439951	gene-MA16_Dca002377
miR396b_1	0.805439951	gene-MA16_Dca026927
miR396b_1	0.805439951	gene-MA16_Dca006631
miR396b_1	0.805439951	gene-MA16_Dca012128
miR396b_1	0.805439951	gene-MA16_Dca002242
miR396b_1	0.805439951	gene-MA16_Dca011294
miR396b_1	0.805439951	gene-MA16_Dca001955
miR396b_1	0.805439951	gene-MA16_Dca010163
miR396b_1	0.805439951	gene-MA16_Dca010619
miR396b_1	0.805439951	gene-MA16_Dca020583
miR396b_1	0.805439951	gene-MA16_Dca003911
miR396b_1	0.805439951	gene-MA16_Dca028471
miR396b_1	0.805439951	gene-MA16_Dca025262

miR396b_1	0.805439951	gene-MA16_Dca011382
miR396b_1	0.805439951	gene-MA16_Dca012099
miR396b_1	0.805439951	gene-MA16_Dca020910
miR396b_1	0.805439951	gene-MA16_Dca010708
miR396b_1	0.805439951	gene-MA16_Dca004692
miR396b_1	0.805439951	gene-MA16_Dca003177
miR396b_1	0.805439951	gene-MA16_Dca024988
miR396b_1	0.805439951	gene-MA16_Dca027492
miR396b_1	0.805439951	gene-MA16_Dca011959
miR396b_1	0.805439951	gene-MA16_Dca011627
miR396b_1	0.805439951	gene-MA16_Dca021446
miR396b_1	0.805439951	gene-MA16_Dca020207
miR396b_1	0.805439951	gene-MA16_Dca007949
miR396b_1	0.805439951	gene-MA16_Dca016956
miR396b_1	0.805439951	gene-MA16_Dca011603
miR396b_1	0.805439951	gene-MA16_Dca013322
miR396b_1	0.805439951	gene-MA16_Dca025223
miR396b_1	0.805439951	gene-MA16_Dca009226
miR396b_1	0.805439951	gene-MA16_Dca024281
miR396b_1	0.805439951	gene-MA16_Dca015409
miR396b_1	0.805439951	gene-MA16_Dca023601
miR396b_1	0.805439951	gene-MA16_Dca004403
miR396b_1	0.805439951	gene-MA16_Dca019222
miR396b_1	0.805439951	gene-MA16_Dca024737
miR396b_1	0.805439951	gene-MA16_Dca014751
miR396b_1	0.805439951	gene-MA16_Dca022302
miR396b_1	0.805439951	gene-MA16_Dca005617
miR396b_1	0.805439951	gene-MA16_Dca000619
miR396b_1	0.805439951	gene-MA16_Dca002697
miR396b_1	0.805439951	gene-MA16_Dca007955
miR396b_1	0.805439951	gene-MA16_Dca020801
miR396b_1	0.805439951	gene-MA16_Dca012938
miR396b_1	0.805439951	gene-MA16_Dca002241
miR396b_1	0.805439951	gene-MA16_Dca019707
miR396b_1	0.805439951	gene-MA16_Dca020166
miR396b_1	0.805439951	gene-MA16_Dca018345
miR396b_1	0.805439951	gene-MA16_Dca008222
miR396b_1	0.805439951	gene-MA16_Dca006666
miR396b_1	0.805439951	gene-MA16_Dca007694
miR396b_1	0.805439951	gene-MA16_Dca000885
miR396b_1	0.805439951	gene-MA16_Dca011783
miR396b_1	0.805439951	gene-MA16_Dca005151
miR396b_1	0.805439951	gene-MA16_Dca011967
miR396b_1	0.805439951	gene-MA16_Dca011523
miR396b_1	0.805439951	gene-MA16_Dca011751
miR396b_1	0.805439951	gene-MA16_Dca015263

miR396b_1	0.805439951	gene-MA16_Dca005154
novel_miR_87	-0.657817551	gene-MA16_Dca011627
novel_miR_87	-0.657817551	gene-MA16_Dca006730
novel_miR_87	-0.657817551	gene-MA16_Dca023929
novel_miR_87	-0.657817551	gene-MA16_Dca024649
novel_miR_87	-0.657817551	gene-MA16_Dca000440
novel_miR_87	-0.657817551	gene-MA16_Dca009490
novel_miR_87	-0.657817551	gene-MA16_Dca006916
novel_miR_87	-0.657817551	gene-MA16_Dca024036
novel_miR_87	-0.657817551	gene-MA16_Dca021780
miR393b_3p	0.711314138	gene-MA16_Dca024562
novel_miR_97	-0.69321229	gene-MA16_Dca009490
novel_miR_97	-0.69321229	gene-MA16_Dca004841
novel_miR_97	-0.69321229	gene-MA16_Dca003193
miR1520f_5p_11	-0.694124597	gene-MA16_Dca004841
novel_miR_280	-1.020762776	gene-MA16_Dca023581
novel_miR_280	-1.020762776	gene-MA16_Dca026290
novel_miR_280	-1.020762776	gene-MA16_Dca006234
novel_miR_280	-1.020762776	gene-MA16_Dca019870
novel_miR_280	-1.020762776	gene-MA16_Dca022292
novel_miR_280	-1.020762776	gene-MA16_Dca019243
novel_miR_280	-1.020762776	gene-MA16_Dca021320
novel_miR_280	-1.020762776	gene-MA16_Dca008936
novel_miR_280	-1.020762776	gene-MA16_Dca023290
novel_miR_280	-1.020762776	gene-MA16_Dca008635
novel_miR_280	-1.020762776	gene-MA16_Dca000122
novel_miR_280	-1.020762776	gene-MA16_Dca000505
novel_miR_280	-1.020762776	gene-MA16_Dca014483
novel_miR_280	-1.020762776	gene-MA16_Dca012806
novel_miR_280	-1.020762776	gene-MA16_Dca006228
novel_miR_280	-1.020762776	gene-MA16_Dca005089
novel_miR_280	-1.020762776	gene-MA16_Dca022914
novel_miR_280	-1.020762776	gene-MA16_Dca009985
novel_miR_280	-1.020762776	gene-MA16_Dca009642
novel_miR_280	-1.020762776	gene-MA16_Dca013592
novel_miR_280	-1.020762776	gene-MA16_Dca019648
novel_miR_280	-1.020762776	gene-MA16_Dca020263
novel_miR_280	-1.020762776	gene-MA16_Dca005939
novel_miR_280	-1.020762776	gene-MA16_Dca003381
novel_miR_280	-1.020762776	gene-MA16_Dca004513
novel_miR_280	-1.020762776	gene-MA16_Dca025963
novel_miR_280	-1.020762776	gene-MA16_Dca012896
novel_miR_280	-1.020762776	gene-MA16_Dca021885
novel_miR_280	-1.020762776	gene-MA16_Dca007184
novel_miR_280	-1.020762776	gene-MA16_Dca007682
novel_miR_280	-1.020762776	gene-MA16_Dca003096

novel_miR_280	-1.020762776	gene-MA16_Dca010390
novel_miR_280	-1.020762776	gene-MA16_Dca022181
novel_miR_280	-1.020762776	gene-MA16_Dca004775
novel_miR_280	-1.020762776	gene-MA16_Dca023756
novel_miR_280	-1.020762776	gene-MA16_Dca007605
novel_miR_280	-1.020762776	gene-MA16_Dca005159
novel_miR_280	-1.020762776	gene-MA16_Dca023943
novel_miR_280	-1.020762776	gene-MA16_Dca020860
novel_miR_280	-1.020762776	gene-MA16_Dca026072
novel_miR_280	-1.020762776	gene-MA16_Dca012781
novel_miR_280	-1.020762776	gene-MA16_Dca003665
novel_miR_280	-1.020762776	gene-MA16_Dca001757
novel_miR_280	-1.020762776	gene-MA16_Dca010749
novel_miR_280	-1.020762776	gene-MA16_Dca020671
novel_miR_280	-1.020762776	gene-MA16_Dca017663
novel_miR_280	-1.020762776	gene-MA16_Dca012780
novel_miR_280	-1.020762776	gene-MA16_Dca004095
novel_miR_280	-1.020762776	gene-MA16_Dca023432
novel_miR_280	-1.020762776	gene-MA16_Dca018979
novel_miR_280	-1.020762776	gene-MA16_Dca002218
novel_miR_280	-1.020762776	gene-MA16_Dca011246
novel_miR_280	-1.020762776	gene-MA16_Dca003404
novel_miR_280	-1.020762776	gene-MA16_Dca007171
novel_miR_280	-1.020762776	gene-MA16_Dca019854
novel_miR_280	-1.020762776	gene-MA16_Dca010102
novel_miR_280	-1.020762776	gene-MA16_Dca009576
novel_miR_280	-1.020762776	gene-MA16_Dca019643
novel_miR_280	-1.020762776	gene-MA16_Dca009701
novel_miR_280	-1.020762776	gene-MA16_Dca003212
novel_miR_280	-1.020762776	gene-MA16_Dca015741
novel_miR_280	-1.020762776	gene-MA16_Dca011329
novel_miR_280	-1.020762776	gene-MA16_Dca008190
novel_miR_280	-1.020762776	gene-MA16_Dca009084
novel_miR_280	-1.020762776	gene-MA16_Dca013734
novel_miR_280	-1.020762776	gene-MA16_Dca002597
novel_miR_280	-1.020762776	gene-MA16_Dca002337
novel_miR_280	-1.020762776	gene-MA16_Dca004293
novel_miR_280	-1.020762776	gene-MA16_Dca003118
novel_miR_280	-1.020762776	gene-MA16_Dca016948
novel_miR_280	-1.020762776	gene-MA16_Dca015250
novel_miR_280	-1.020762776	gene-MA16_Dca005798
novel_miR_280	-1.020762776	gene-MA16_Dca001003
novel_miR_280	-1.020762776	gene-MA16_Dca016504
novel_miR_280	-1.020762776	gene-MA16_Dca009267
novel_miR_280	-1.020762776	gene-MA16_Dca013779
novel_miR_280	-1.020762776	gene-MA16_Dca006414

novel_miR_280	-1.020762776	gene-MA16_Dca019173
novel_miR_280	-1.020762776	gene-MA16_Dca017709
novel_miR_280	-1.020762776	gene-MA16_Dca016510
novel_miR_280	-1.020762776	gene-MA16_Dca015291
novel_miR_280	-1.020762776	gene-MA16_Dca005992
novel_miR_280	-1.020762776	gene-MA16_Dca013294
novel_miR_280	-1.020762776	gene-MA16_Dca022196
novel_miR_280	-1.020762776	gene-MA16_Dca018178
novel_miR_280	-1.020762776	gene-MA16_Dca010665
novel_miR_280	-1.020762776	gene-MA16_Dca002756
novel_miR_280	-1.020762776	gene-MA16_Dca011027
novel_miR_280	-1.020762776	gene-MA16_Dca017043
novel_miR_280	-1.020762776	gene-MA16_Dca004102
novel_miR_280	-1.020762776	gene-MA16_Dca027631
novel_miR_280	-1.020762776	gene-MA16_Dca023559
novel_miR_280	-1.020762776	gene-MA16_Dca011106
novel_miR_280	-1.020762776	gene-MA16_Dca017541
novel_miR_280	-1.020762776	gene-MA16_Dca000121
novel_miR_280	-1.020762776	gene-MA16_Dca016374
novel_miR_280	-1.020762776	gene-MA16_Dca008843
novel_miR_280	-1.020762776	gene-MA16_Dca018560
novel_miR_280	-1.020762776	gene-MA16_Dca001258
novel_miR_280	-1.020762776	gene-MA16_Dca003197
novel_miR_280	-1.020762776	gene-MA16_Dca012420
novel_miR_287	-0.647947681	gene-MA16_Dca021780
novel_miR_287	-0.647947681	gene-MA16_Dca024036
novel_miR_287	-0.647947681	gene-MA16_Dca006916
novel_miR_287	-0.647947681	gene-MA16_Dca024649
novel_miR_287	-0.647947681	gene-MA16_Dca000440
novel_miR_287	-0.647947681	gene-MA16_Dca009490
novel_miR_287	-0.647947681	gene-MA16_Dca011627
novel_miR_287	-0.647947681	gene-MA16_Dca006730
novel_miR_287	-0.647947681	gene-MA16_Dca023929
novel_miR_506	-1.020760459	gene-MA16_Dca003212
novel_miR_506	-1.020760459	gene-MA16_Dca015741
novel_miR_506	-1.020760459	gene-MA16_Dca011329
novel_miR_506	-1.020760459	gene-MA16_Dca008190
novel_miR_506	-1.020760459	gene-MA16_Dca009084
novel_miR_506	-1.020760459	gene-MA16_Dca003404
novel_miR_506	-1.020760459	gene-MA16_Dca007171
novel_miR_506	-1.020760459	gene-MA16_Dca019854
novel_miR_506	-1.020760459	gene-MA16_Dca010102
novel_miR_506	-1.020760459	gene-MA16_Dca009576
novel_miR_506	-1.020760459	gene-MA16_Dca009701
novel_miR_506	-1.020760459	gene-MA16_Dca019643
novel_miR_506	-1.020760459	gene-MA16_Dca016948

novel_miR_506	-1.020760459	gene-MA16_Dca015250
novel_miR_506	-1.020760459	gene-MA16_Dca005798
novel_miR_506	-1.020760459	gene-MA16_Dca016504
novel_miR_506	-1.020760459	gene-MA16_Dca001003
novel_miR_506	-1.020760459	gene-MA16_Dca013779
novel_miR_506	-1.020760459	gene-MA16_Dca009267
novel_miR_506	-1.020760459	gene-MA16_Dca002597
novel_miR_506	-1.020760459	gene-MA16_Dca013734
novel_miR_506	-1.020760459	gene-MA16_Dca004293
novel_miR_506	-1.020760459	gene-MA16_Dca002337
novel_miR_506	-1.020760459	gene-MA16_Dca003118
novel_miR_506	-1.020760459	gene-MA16_Dca016510
novel_miR_506	-1.020760459	gene-MA16_Dca017709
novel_miR_506	-1.020760459	gene-MA16_Dca015291
novel_miR_506	-1.020760459	gene-MA16_Dca013294
novel_miR_506	-1.020760459	gene-MA16_Dca005992
novel_miR_506	-1.020760459	gene-MA16_Dca022196
novel_miR_506	-1.020760459	gene-MA16_Dca006414
novel_miR_506	-1.020760459	gene-MA16_Dca019173
novel_miR_506	-1.020760459	gene-MA16_Dca000121
novel_miR_506	-1.020760459	gene-MA16_Dca016374
novel_miR_506	-1.020760459	gene-MA16_Dca008843
novel_miR_506	-1.020760459	gene-MA16_Dca018560
novel_miR_506	-1.020760459	gene-MA16_Dca001258
novel_miR_506	-1.020760459	gene-MA16_Dca003197
novel_miR_506	-1.020760459	gene-MA16_Dca012420
novel_miR_506	-1.020760459	gene-MA16_Dca018178
novel_miR_506	-1.020760459	gene-MA16_Dca010665
novel_miR_506	-1.020760459	gene-MA16_Dca002756
novel_miR_506	-1.020760459	gene-MA16_Dca011027
novel_miR_506	-1.020760459	gene-MA16_Dca017043
novel_miR_506	-1.020760459	gene-MA16_Dca004102
novel_miR_506	-1.020760459	gene-MA16_Dca011106
novel_miR_506	-1.020760459	gene-MA16_Dca027631
novel_miR_506	-1.020760459	gene-MA16_Dca023559
novel_miR_506	-1.020760459	gene-MA16_Dca017541
novel_miR_506	-1.020760459	gene-MA16_Dca023290
novel_miR_506	-1.020760459	gene-MA16_Dca008635
novel_miR_506	-1.020760459	gene-MA16_Dca000122
novel_miR_506	-1.020760459	gene-MA16_Dca000505
novel_miR_506	-1.020760459	gene-MA16_Dca014483
novel_miR_506	-1.020760459	gene-MA16_Dca012806
novel_miR_506	-1.020760459	gene-MA16_Dca023581
novel_miR_506	-1.020760459	gene-MA16_Dca026290
novel_miR_506	-1.020760459	gene-MA16_Dca006234
novel_miR_506	-1.020760459	gene-MA16_Dca019870

novel_miR_506	-1.020760459	gene-MA16_Dca019243
novel_miR_506	-1.020760459	gene-MA16_Dca022292
novel_miR_506	-1.020760459	gene-MA16_Dca008936
novel_miR_506	-1.020760459	gene-MA16_Dca021320
novel_miR_506	-1.020760459	gene-MA16_Dca006228
novel_miR_506	-1.020760459	gene-MA16_Dca005089
novel_miR_506	-1.020760459	gene-MA16_Dca022914
novel_miR_506	-1.020760459	gene-MA16_Dca009985
novel_miR_506	-1.020760459	gene-MA16_Dca007184
novel_miR_506	-1.020760459	gene-MA16_Dca003096
novel_miR_506	-1.020760459	gene-MA16_Dca007682
novel_miR_506	-1.020760459	gene-MA16_Dca010390
novel_miR_506	-1.020760459	gene-MA16_Dca022181
novel_miR_506	-1.020760459	gene-MA16_Dca004775
novel_miR_506	-1.020760459	gene-MA16_Dca023756
novel_miR_506	-1.020760459	gene-MA16_Dca007605
novel_miR_506	-1.020760459	gene-MA16_Dca009642
novel_miR_506	-1.020760459	gene-MA16_Dca020263
novel_miR_506	-1.020760459	gene-MA16_Dca019648
novel_miR_506	-1.020760459	gene-MA16_Dca013592
novel_miR_506	-1.020760459	gene-MA16_Dca005939
novel_miR_506	-1.020760459	gene-MA16_Dca003381
novel_miR_506	-1.020760459	gene-MA16_Dca004513
novel_miR_506	-1.020760459	gene-MA16_Dca012896
novel_miR_506	-1.020760459	gene-MA16_Dca025963
novel_miR_506	-1.020760459	gene-MA16_Dca021885
novel_miR_506	-1.020760459	gene-MA16_Dca018979
novel_miR_506	-1.020760459	gene-MA16_Dca023432
novel_miR_506	-1.020760459	gene-MA16_Dca011246
novel_miR_506	-1.020760459	gene-MA16_Dca002218
novel_miR_506	-1.020760459	gene-MA16_Dca005159
novel_miR_506	-1.020760459	gene-MA16_Dca023943
novel_miR_506	-1.020760459	gene-MA16_Dca020860
novel_miR_506	-1.020760459	gene-MA16_Dca026072
novel_miR_506	-1.020760459	gene-MA16_Dca012781
novel_miR_506	-1.020760459	gene-MA16_Dca010749
novel_miR_506	-1.020760459	gene-MA16_Dca003665
novel_miR_506	-1.020760459	gene-MA16_Dca001757
novel_miR_506	-1.020760459	gene-MA16_Dca017663
novel_miR_506	-1.020760459	gene-MA16_Dca020671
novel_miR_506	-1.020760459	gene-MA16_Dca012780
novel_miR_506	-1.020760459	gene-MA16_Dca004095
miR408_5p	-1.45887466	gene-MA16_Dca006187
miR408_5p	-1.45887466	gene-MA16_Dca025324
miR408_5p	-1.45887466	gene-MA16_Dca020801
miR408_5p	-1.45887466	gene-MA16_Dca018282

miR408_5p	-1.45887466	gene-MA16_Dca007809
miR408_5p	-1.45887466	gene-MA16_Dca002968
miR408_5p	-1.45887466	gene-MA16_Dca014182
miR408_5p	-1.45887466	gene-MA16_Dca013452
miR408_5p	-1.45887466	gene-MA16_Dca023283
miR408_5p	-1.45887466	gene-MA16_Dca022320
miR408_5p	-1.45887466	gene-MA16_Dca022503
miR408_5p	-1.45887466	gene-MA16_Dca012776
miR408_5p	-1.45887466	gene-MA16_Dca021620
		novel_miR_202 0.855751983
novel_miR_202	0.855751983	gene-MA16_Dca003551
novel_miR_202	0.855751983	gene-MA16_Dca023726
novel_miR_202	0.855751983	gene-MA16_Dca027179
novel_miR_202	0.855751983	gene-MA16_Dca015527
novel_miR_202	0.855751983	gene-MA16_Dca025989
novel_miR_202	0.855751983	gene-MA16_Dca027946
novel_miR_202	0.855751983	gene-MA16_Dca015912
novel_miR_202	0.855751983	gene-MA16_Dca027976
novel_miR_202	0.855751983	gene-MA16_Dca003689
novel_miR_202	0.855751983	gene-MA16_Dca012698
novel_miR_202	0.855751983	gene-MA16_Dca004912
novel_miR_202	0.855751983	gene-MA16_Dca016948
novel_miR_202	0.855751983	gene-MA16_Dca005929
novel_miR_202	0.855751983	gene-MA16_Dca017812
novel_miR_202	0.855751983	gene-MA16_Dca005529
novel_miR_202	0.855751983	gene-MA16_Dca028117
novel_miR_202	0.855751983	gene-MA16_Dca015720
novel_miR_202	0.855751983	gene-MA16_Dca016419
novel_miR_202	0.855751983	gene-MA16_Dca026801
novel_miR_202	0.855751983	gene-MA16_Dca019346
novel_miR_202	0.855751983	gene-MA16_Dca022009
novel_miR_202	0.855751983	gene-MA16_Dca026108
novel_miR_202	0.855751983	gene-MA16_Dca028824
novel_miR_202	0.855751983	gene-MA16_Dca017373
novel_miR_202	0.855751983	gene-MA16_Dca017912
novel_miR_202	0.855751983	gene-MA16_Dca024245
novel_miR_202	0.855751983	gene-MA16_Dca011928
novel_miR_202	0.855751983	gene-MA16_Dca021654
novel_miR_45	0.886643802	gene-MA16_Dca005043
novel_miR_45	0.886643802	gene-MA16_Dca019234
novel_miR_45	0.886643802	gene-MA16_Dca024771
novel_miR_45	0.886643802	gene-MA16_Dca009889
novel_miR_45	0.886643802	gene-MA16_Dca007090
novel_miR_45	0.886643802	gene-MA16_Dca003489
novel_miR_45	0.886643802	gene-MA16_Dca008938

novel_miR_45	0.886643802	gene-MA16_Dca011854
novel_miR_45	0.886643802	gene-MA16_Dca012991
novel_miR_45	0.886643802	gene-MA16_Dca025486
novel_miR_45	0.886643802	gene-MA16_Dca001590
novel_miR_45	0.886643802	gene-MA16_Dca012774
novel_miR_45	0.886643802	gene-MA16_Dca010838
novel_miR_45	0.886643802	gene-MA16_Dca008335
miR396b_3p	0.953858207	gene-MA16_Dca026208
miR396b_3p	0.953858207	gene-MA16_Dca008181
miR396b_3p	0.953858207	gene-MA16_Dca015968
miR396b_3p	0.953858207	gene-MA16_Dca006688
miR396b_3p	0.953858207	gene-MA16_Dca018822
miR396b_3p	0.953858207	gene-MA16_Dca014117
miR396b_3p	0.953858207	gene-MA16_Dca015480
miR396b_3p	0.953858207	gene-MA16_Dca001294
miR396b_3p	0.953858207	gene-MA16_Dca003662
miR396b_3p	0.953858207	gene-MA16_Dca022078
miR396b_3p	0.953858207	gene-MA16_Dca020272
miR396b_3p	0.953858207	gene-MA16_Dca017621
miR396b_3p	0.953858207	gene-MA16_Dca009793
miR396b_3p	0.953858207	gene-MA16_Dca003664
miR396b_3p	0.953858207	gene-MA16_Dca010894
miR396b_3p	0.953858207	gene-MA16_Dca010653
miR396b_3p	0.953858207	gene-MA16_Dca022876
miR396b_3p	0.953858207	gene-MA16_Dca005699
miR396b_3p	0.953858207	gene-MA16_Dca027789
miR396b_3p	0.953858207	gene-MA16_Dca005341
miR396b_3p	0.953858207	gene-MA16_Dca012062
miR396b_3p	0.953858207	gene-MA16_Dca004712
miR396b_3p	0.953858207	gene-MA16_Dca013400
miR396b_3p	0.953858207	gene-MA16_Dca007582
miR396b_3p	0.953858207	gene-MA16_Dca023402
miR396b_3p	0.953858207	gene-MA16_Dca003014
miR396b_3p	0.953858207	gene-MA16_Dca006076
miR396b_3p	0.953858207	gene-MA16_Dca014625
miR396b_3p	0.953858207	gene-MA16_Dca020802
miR396b_3p	0.953858207	gene-MA16_Dca001239
miR396b_3p	0.953858207	gene-MA16_Dca027486
miR396b_3p	0.953858207	gene-MA16_Dca001761
miR396b_3p	0.953858207	gene-MA16_Dca022596
miR396b_3p	0.953858207	gene-MA16_Dca005778
miR396b_3p	0.953858207	gene-MA16_Dca024206
miR396b_3p	0.953858207	gene-MA16_Dca017707
miR396b_3p	0.953858207	gene-MA16_Dca013600
miR4387e	1.098733759	gene-MA16_Dca010295
miR4387e	1.098733759	gene-MA16_Dca012689

novel_miR_220	-0.59936991	gene-MA16_Dca017059
novel_miR_220	-0.59936991	gene-MA16_Dca013211
novel_miR_501	0.648918365	gene-MA16_Dca023667
novel_miR_501	0.648918365	gene-MA16_Dca020360
novel_miR_501	0.648918365	gene-MA16_Dca021751
novel_miR_501	0.648918365	gene-MA16_Dca020021
novel_miR_501	0.648918365	gene-MA16_Dca026376
novel_miR_501	0.648918365	gene-MA16_Dca020924
novel_miR_501	0.648918365	gene-MA16_Dca024999
novel_miR_501	0.648918365	gene-MA16_Dca005077
novel_miR_501	0.648918365	gene-MA16_Dca022668
novel_miR_501	0.648918365	gene-MA16_Dca010934
novel_miR_501	0.648918365	gene-MA16_Dca023672
novel_miR_501	0.648918365	gene-MA16_Dca022482
novel_miR_501	0.648918365	gene-MA16_Dca015559
novel_miR_501	0.648918365	gene-MA16_Dca020386
novel_miR_501	0.648918365	gene-MA16_Dca014238
novel_miR_501	0.648918365	gene-MA16_Dca005251
novel_miR_501	0.648918365	gene-MA16_Dca017447
novel_miR_180	-0.663108588	gene-MA16_Dca009629
novel_miR_180	-0.663108588	gene-MA16_Dca009628
novel_miR_180	-0.663108588	gene-MA16_Dca023796
novel_miR_180	-0.663108588	gene-MA16_Dca002782
novel_miR_180	-0.663108588	gene-MA16_Dca020882
novel_miR_180	-0.663108588	gene-MA16_Dca011590
novel_miR_180	-0.663108588	gene-MA16_Dca013202
novel_miR_180	-0.663108588	gene-MA16_Dca021909
novel_miR_180	-0.663108588	gene-MA16_Dca021285
novel_miR_180	-0.663108588	gene-MA16_Dca026967
novel_miR_180	-0.663108588	gene-MA16_Dca016402
novel_miR_180	-0.663108588	gene-MA16_Dca015434
novel_miR_180	-0.663108588	gene-MA16_Dca024328
novel_miR_180	-0.663108588	gene-MA16_Dca017666
novel_miR_180	-0.663108588	gene-MA16_Dca017578
novel_miR_180	-0.663108588	gene-MA16_Dca001496
novel_miR_180	-0.663108588	gene-MA16_Dca024154
novel_miR_180	-0.663108588	gene-MA16_Dca021423
novel_miR_180	-0.663108588	gene-MA16_Dca000889
novel_miR_180	-0.663108588	gene-MA16_Dca005161
novel_miR_180	-0.663108588	gene-MA16_Dca025505
novel_miR_180	-0.663108588	gene-MA16_Dca009052
novel_miR_180	-0.663108588	gene-MA16_Dca000422
novel_miR_180	-0.663108588	gene-MA16_Dca001153
novel_miR_180	-0.663108588	gene-MA16_Dca013631
novel_miR_180	-0.663108588	gene-MA16_Dca007347
novel_miR_180	-0.663108588	gene-MA16_Dca011965

novel_miR_180	-0.663108588	gene-MA16_Dca027199
novel_miR_180	-0.663108588	gene-MA16_Dca005930
novel_miR_180	-0.663108588	gene-MA16_Dca011869
novel_miR_180	-0.663108588	gene-MA16_Dca020020
novel_miR_180	-0.663108588	gene-MA16_Dca020331
novel_miR_180	-0.663108588	gene-MA16_Dca008388
novel_miR_180	-0.663108588	gene-MA16_Dca017689
novel_miR_180	-0.663108588	gene-MA16_Dca011409
novel_miR_180	-0.663108588	gene-MA16_Dca003158
novel_miR_180	-0.663108588	gene-MA16_Dca009868
novel_miR_180	-0.663108588	gene-MA16_Dca011489
novel_miR_180	-0.663108588	gene-MA16_Dca003339
novel_miR_180	-0.663108588	gene-MA16_Dca011481
novel_miR_180	-0.663108588	gene-MA16_Dca007171
novel_miR_180	-0.663108588	gene-MA16_Dca024966
novel_miR_180	-0.663108588	gene-MA16_Dca003354
novel_miR_180	-0.663108588	gene-MA16_Dca007191
novel_miR_180	-0.663108588	gene-MA16_Dca026850
novel_miR_180	-0.663108588	gene-MA16_Dca003270
novel_miR_180	-0.663108588	gene-MA16_Dca000617
novel_miR_180	-0.663108588	gene-MA16_Dca006230
novel_miR_180	-0.663108588	gene-MA16_Dca023911
novel_miR_180	-0.663108588	gene-MA16_Dca016806
novel_miR_180	-0.663108588	gene-MA16_Dca021754
novel_miR_180	-0.663108588	gene-MA16_Dca003102
novel_miR_180	-0.663108588	gene-MA16_Dca016487
novel_miR_180	-0.663108588	gene-MA16_Dca008238
novel_miR_180	-0.663108588	gene-MA16_Dca008911
novel_miR_180	-0.663108588	gene-MA16_Dca011166
novel_miR_180	-0.663108588	gene-MA16_Dca007583
novel_miR_180	-0.663108588	gene-MA16_Dca021760
novel_miR_180	-0.663108588	gene-MA16_Dca020137
novel_miR_180	-0.663108588	gene-MA16_Dca015677
novel_miR_180	-0.663108588	gene-MA16_Dca017986
novel_miR_180	-0.663108588	gene-MA16_Dca007177
novel_miR_180	-0.663108588	gene-MA16_Dca006167
novel_miR_180	-0.663108588	gene-MA16_Dca016488
novel_miR_180	-0.663108588	gene-MA16_Dca018635
novel_miR_180	-0.663108588	gene-MA16_Dca006505
novel_miR_180	-0.663108588	gene-MA16_Dca027198
novel_miR_180	-0.663108588	gene-MA16_Dca014467
novel_miR_180	-0.663108588	gene-MA16_Dca011374
novel_miR_180	-0.663108588	gene-MA16_Dca002239
novel_miR_115	-0.590319003	gene-MA16_Dca013211
novel_miR_115	-0.590319003	gene-MA16_Dca017059
novel_miR_425	0.62445181	gene-MA16_Dca012177

novel_miR_425	0.62445181	gene-MA16_Dca021600
novel_miR_425	0.62445181	gene-MA16_Dca022152
novel_miR_425	0.62445181	gene-MA16_Dca022444
novel_miR_425	0.62445181	gene-MA16_Dca024641
novel_miR_99	0.645554446	gene-MA16_Dca021498
novel_miR_99	0.645554446	gene-MA16_Dca011199
novel_miR_496	0.817053762	gene-MA16_Dca011647
miR857	-0.739412556	gene-MA16_Dca011056
miR857	-0.739412556	gene-MA16_Dca023413
miR857	-0.739412556	gene-MA16_Dca013080
miR857	-0.739412556	gene-MA16_Dca021249
miR857	-0.739412556	gene-MA16_Dca011207
miR857	-0.739412556	gene-MA16_Dca007591
miR857	-0.739412556	gene-MA16_Dca005380
miR857	-0.739412556	gene-MA16_Dca027103
novel_miR_159	-0.612642562	gene-MA16_Dca013669
novel_miR_159	-0.612642562	gene-MA16_Dca015045
novel_miR_159	-0.612642562	gene-MA16_Dca016284
novel_miR_159	-0.612642562	gene-MA16_Dca018922
novel_miR_159	-0.612642562	gene-MA16_Dca007765
novel_miR_159	-0.612642562	gene-MA16_Dca007257
novel_miR_159	-0.612642562	gene-MA16_Dca026264
novel_miR_159	-0.612642562	gene-MA16_Dca020115
novel_miR_159	-0.612642562	gene-MA16_Dca001020
novel_miR_159	-0.612642562	gene-MA16_Dca001828
novel_miR_159	-0.612642562	gene-MA16_Dca016652
novel_miR_159	-0.612642562	gene-MA16_Dca027076
novel_miR_159	-0.612642562	gene-MA16_Dca013843
novel_miR_159	-0.612642562	gene-MA16_Dca008856
novel_miR_159	-0.612642562	gene-MA16_Dca006592
novel_miR_159	-0.612642562	gene-MA16_Dca021824
novel_miR_159	-0.612642562	gene-MA16_Dca006324
novel_miR_159	-0.612642562	gene-MA16_Dca014974
novel_miR_159	-0.612642562	gene-MA16_Dca022392
novel_miR_159	-0.612642562	gene-MA16_Dca010817
novel_miR_159	-0.612642562	gene-MA16_Dca007613
novel_miR_159	-0.612642562	gene-MA16_Dca017065
novel_miR_159	-0.612642562	gene-MA16_Dca007050
novel_miR_159	-0.612642562	gene-MA16_Dca005372
novel_miR_159	-0.612642562	gene-MA16_Dca022401
novel_miR_159	-0.612642562	gene-MA16_Dca007408
novel_miR_159	-0.612642562	gene-MA16_Dca016389
novel_miR_159	-0.612642562	gene-MA16_Dca016013
novel_miR_159	-0.612642562	gene-MA16_Dca012490
novel_miR_159	-0.612642562	gene-MA16_Dca021414
novel_miR_159	-0.612642562	gene-MA16_Dca011522

novel_miR_159	-0.612642562	gene-MA16_Dca015083
novel_miR_159	-0.612642562	gene-MA16_Dca006829
novel_miR_159	-0.612642562	gene-MA16_Dca014007
novel_miR_159	-0.612642562	gene-MA16_Dca006179
novel_miR_159	-0.612642562	gene-MA16_Dca019278
novel_miR_159	-0.612642562	gene-MA16_Dca001444
novel_miR_159	-0.612642562	gene-MA16_Dca014143
novel_miR_159	-0.612642562	gene-MA16_Dca019818
novel_miR_159	-0.612642562	gene-MA16_Dca015975
novel_miR_159	-0.612642562	gene-MA16_Dca006828
novel_miR_159	-0.612642562	gene-MA16_Dca023636
novel_miR_159	-0.612642562	gene-MA16_Dca027407
novel_miR_159	-0.612642562	gene-MA16_Dca002029
novel_miR_159	-0.612642562	gene-MA16_Dca001924
novel_miR_159	-0.612642562	gene-MA16_Dca022572
novel_miR_159	-0.612642562	gene-MA16_Dca010252
novel_miR_159	-0.612642562	gene-MA16_Dca023553
novel_miR_159	-0.612642562	gene-MA16_Dca005107
novel_miR_159	-0.612642562	gene-MA16_Dca024023
novel_miR_159	-0.612642562	gene-MA16_Dca024366
novel_miR_159	-0.612642562	gene-MA16_Dca002789
novel_miR_159	-0.612642562	gene-MA16_Dca019273
novel_miR_159	-0.612642562	gene-MA16_Dca011278
novel_miR_159	-0.612642562	gene-MA16_Dca018789
novel_miR_159	-0.612642562	gene-MA16_Dca018071
novel_miR_159	-0.612642562	gene-MA16_Dca021047
novel_miR_159	-0.612642562	gene-MA16_Dca003342
novel_miR_159	-0.612642562	gene-MA16_Dca005616
novel_miR_159	-0.612642562	gene-MA16_Dca025367
novel_miR_159	-0.612642562	gene-MA16_Dca028626
novel_miR_159	-0.612642562	gene-MA16_Dca012922
miR159k_3p_1	-0.734955031	gene-MA16_Dca006397
miR159k_3p_1	-0.734955031	gene-MA16_Dca010165
miR159k_3p_1	-0.734955031	gene-MA16_Dca001340
miR159k_3p_1	-0.734955031	gene-MA16_Dca011035
miR159k_3p_1	-0.734955031	gene-MA16_Dca024728
miR159k_3p_1	-0.734955031	gene-MA16_Dca027107
miR159k_3p_1	-0.734955031	gene-MA16_Dca015701
miR159k_3p_1	-0.734955031	gene-MA16_Dca023804
miR159k_3p_1	-0.734955031	gene-MA16_Dca007257
miR159k_3p_1	-0.734955031	gene-MA16_Dca012195
miR159k_3p_1	-0.734955031	gene-MA16_Dca022241
miR159k_3p_1	-0.734955031	gene-MA16_Dca012890
miR159k_3p_1	-0.734955031	gene-MA16_Dca016851
miR159k_3p_1	-0.734955031	gene-MA16_Dca014760
miR159k_3p_1	-0.734955031	gene-MA16_Dca016112

miR159k_3p_1	-0.734955031	gene-MA16_Dca027066
miR159k_3p_1	-0.734955031	gene-MA16_Dca019334
miR159k_3p_1	-0.734955031	gene-MA16_Dca008623
miR159k_3p_1	-0.734955031	gene-MA16_Dca018586
miR159k_3p_1	-0.734955031	gene-MA16_Dca000984
miR159k_3p_1	-0.734955031	gene-MA16_Dca028065
miR159k_3p_1	-0.734955031	gene-MA16_Dca025400
miR159k_3p_1	-0.734955031	gene-MA16_Dca020624
miR159k_3p_1	-0.734955031	gene-MA16_Dca024208
miR159k_3p_1	-0.734955031	gene-MA16_Dca000350
miR159k_3p_1	-0.734955031	gene-MA16_Dca005171
miR159k_3p_1	-0.734955031	gene-MA16_Dca008624
miR159k_3p_1	-0.734955031	gene-MA16_Dca011457
miR159k_3p_1	-0.734955031	gene-MA16_Dca019561
miR159k_3p_1	-0.734955031	gene-MA16_Dca026772
miR159k_3p_1	-0.734955031	gene-MA16_Dca019205
miR159k_3p_1	-0.734955031	gene-MA16_Dca028002
miR159k_3p_1	-0.734955031	gene-MA16_Dca001826
miR159k_3p_1	-0.734955031	gene-MA16_Dca024127
miR159k_3p_1	-0.734955031	gene-MA16_Dca000803
miR159k_3p_1	-0.734955031	gene-MA16_Dca007034
miR159k_3p_1	-0.734955031	gene-MA16_Dca024152
miR159k_3p_1	-0.734955031	gene-MA16_Dca019932
miR159k_3p_1	-0.734955031	gene-MA16_Dca010603
miR159k_3p_1	-0.734955031	gene-MA16_Dca019600
miR159k_3p_1	-0.734955031	gene-MA16_Dca024876
miR159k_3p_1	-0.734955031	gene-MA16_Dca012038
miR159k_3p_1	-0.734955031	gene-MA16_Dca020483
miR159k_3p_1	-0.734955031	gene-MA16_Dca003039
miR159k_3p_1	-0.734955031	gene-MA16_Dca027323
miR159k_3p_1	-0.734955031	gene-MA16_Dca020254
miR159k_3p_1	-0.734955031	gene-MA16_Dca025429
miR159k_3p_2	-0.734952601	gene-MA16_Dca008624
miR159k_3p_2	-0.734952601	gene-MA16_Dca000350
miR159k_3p_2	-0.734952601	gene-MA16_Dca005171
miR159k_3p_2	-0.734952601	gene-MA16_Dca001826
miR159k_3p_2	-0.734952601	gene-MA16_Dca028002
miR159k_3p_2	-0.734952601	gene-MA16_Dca019561
miR159k_3p_2	-0.734952601	gene-MA16_Dca011457
miR159k_3p_2	-0.734952601	gene-MA16_Dca019205
miR159k_3p_2	-0.734952601	gene-MA16_Dca026772
miR159k_3p_2	-0.734952601	gene-MA16_Dca019600
miR159k_3p_2	-0.734952601	gene-MA16_Dca010603
miR159k_3p_2	-0.734952601	gene-MA16_Dca000803
miR159k_3p_2	-0.734952601	gene-MA16_Dca024127
miR159k_3p_2	-0.734952601	gene-MA16_Dca019932

miR159k_3p_2	-0.734952601	gene-MA16_Dca024152
miR159k_3p_2	-0.734952601	gene-MA16_Dca007034
miR159k_3p_2	-0.734952601	gene-MA16_Dca027323
miR159k_3p_2	-0.734952601	gene-MA16_Dca003039
miR159k_3p_2	-0.734952601	gene-MA16_Dca025429
miR159k_3p_2	-0.734952601	gene-MA16_Dca020254
miR159k_3p_2	-0.734952601	gene-MA16_Dca012038
miR159k_3p_2	-0.734952601	gene-MA16_Dca024876
miR159k_3p_2	-0.734952601	gene-MA16_Dca020483
miR159k_3p_2	-0.734952601	gene-MA16_Dca027107
miR159k_3p_2	-0.734952601	gene-MA16_Dca024728
miR159k_3p_2	-0.734952601	gene-MA16_Dca011035
miR159k_3p_2	-0.734952601	gene-MA16_Dca023804
miR159k_3p_2	-0.734952601	gene-MA16_Dca015701
miR159k_3p_2	-0.734952601	gene-MA16_Dca010165
miR159k_3p_2	-0.734952601	gene-MA16_Dca006397
miR159k_3p_2	-0.734952601	gene-MA16_Dca001340
miR159k_3p_2	-0.734952601	gene-MA16_Dca012890
miR159k_3p_2	-0.734952601	gene-MA16_Dca014760
miR159k_3p_2	-0.734952601	gene-MA16_Dca016851
miR159k_3p_2	-0.734952601	gene-MA16_Dca012195
miR159k_3p_2	-0.734952601	gene-MA16_Dca007257
miR159k_3p_2	-0.734952601	gene-MA16_Dca022241
miR159k_3p_2	-0.734952601	gene-MA16_Dca008623
miR159k_3p_2	-0.734952601	gene-MA16_Dca018586
miR159k_3p_2	-0.734952601	gene-MA16_Dca016112
miR159k_3p_2	-0.734952601	gene-MA16_Dca019334
miR159k_3p_2	-0.734952601	gene-MA16_Dca027066
miR159k_3p_2	-0.734952601	gene-MA16_Dca025400
miR159k_3p_2	-0.734952601	gene-MA16_Dca020624
miR159k_3p_2	-0.734952601	gene-MA16_Dca024208
miR159k_3p_2	-0.734952601	gene-MA16_Dca028065
miR159k_3p_2	-0.734952601	gene-MA16_Dca000984
novel_miR_71	0.946415698	gene-MA16_Dca025956
novel_miR_236	0.673287052	gene-MA16_Dca026583
novel_miR_236	0.673287052	gene-MA16_Dca017549
novel_miR_236	0.673287052	gene-MA16_Dca002308
novel_miR_236	0.673287052	gene-MA16_Dca004606
novel_miR_236	0.673287052	gene-MA16_Dca007852
novel_miR_236	0.673287052	gene-MA16_Dca011099
novel_miR_236	0.673287052	gene-MA16_Dca007249
novel_miR_236	0.673287052	gene-MA16_Dca003804
novel_miR_236	0.673287052	gene-MA16_Dca014769
novel_miR_244	0.946575693	gene-MA16_Dca025956
novel_miR_23	-0.737786958	gene-MA16_Dca021085
novel_miR_23	-0.737786958	gene-MA16_Dca006313

novel_miR_233	0.771104321	gene-MA16_Dca009077
novel_miR_233	0.771104321	gene-MA16_Dca003862
novel_miR_233	0.771104321	gene-MA16_Dca016068
novel_miR_233	0.771104321	gene-MA16_Dca002910
miR5523	2.720618247	gene-MA16_Dca014098
miR5523	2.720618247	gene-MA16_Dca026585
miR5523	2.720618247	gene-MA16_Dca021919
miR5523	2.720618247	gene-MA16_Dca015175
miR5523	2.720618247	gene-MA16_Dca004307
miR5523	2.720618247	gene-MA16_Dca006944
miR5523	2.720618247	gene-MA16_Dca007917
miR5523	2.720618247	gene-MA16_Dca028513
miR5523	2.720618247	gene-MA16_Dca012642
miR5523	2.720618247	gene-MA16_Dca026781
miR5523	2.720618247	gene-MA16_Dca022690
miR396b_3	0.656895787	gene-MA16_Dca011294
miR396b_3	0.656895787	gene-MA16_Dca002242
miR396b_3	0.656895787	gene-MA16_Dca001955
miR396b_3	0.656895787	gene-MA16_Dca010163
miR396b_3	0.656895787	gene-MA16_Dca010619
miR396b_3	0.656895787	gene-MA16_Dca020583
miR396b_3	0.656895787	gene-MA16_Dca012128
miR396b_3	0.656895787	gene-MA16_Dca026927
miR396b_3	0.656895787	gene-MA16_Dca006631
miR396b_3	0.656895787	gene-MA16_Dca004093
miR396b_3	0.656895787	gene-MA16_Dca002377
miR396b_3	0.656895787	gene-MA16_Dca001199
miR396b_3	0.656895787	gene-MA16_Dca006179
miR396b_3	0.656895787	gene-MA16_Dca024442
miR396b_3	0.656895787	gene-MA16_Dca001936
miR396b_3	0.656895787	gene-MA16_Dca001785
miR396b_3	0.656895787	gene-MA16_Dca001920
miR396b_3	0.656895787	gene-MA16_Dca024581
miR396b_3	0.656895787	gene-MA16_Dca018172
miR396b_3	0.656895787	gene-MA16_Dca012093
miR396b_3	0.656895787	gene-MA16_Dca000754
miR396b_3	0.656895787	gene-MA16_Dca018548
miR396b_3	0.656895787	gene-MA16_Dca013497
miR396b_3	0.656895787	gene-MA16_Dca023379
miR396b_3	0.656895787	gene-MA16_Dca019943
miR396b_3	0.656895787	gene-MA16_Dca006898
miR396b_3	0.656895787	gene-MA16_Dca019207
miR396b_3	0.656895787	gene-MA16_Dca019153
miR396b_3	0.656895787	gene-MA16_Dca010222
miR396b_3	0.656895787	gene-MA16_Dca021850
miR396b_3	0.656895787	gene-MA16_Dca000152

miR396b_3	0.656895787	gene-MA16_Dca022211
miR396b_3	0.656895787	gene-MA16_Dca010315
miR396b_3	0.656895787	gene-MA16_Dca023840
miR396b_3	0.656895787	gene-MA16_Dca006059
miR396b_3	0.656895787	gene-MA16_Dca018725
miR396b_3	0.656895787	gene-MA16_Dca023691
miR396b_3	0.656895787	gene-MA16_Dca005799
miR396b_3	0.656895787	gene-MA16_Dca021863
miR396b_3	0.656895787	gene-MA16_Dca022508
miR396b_3	0.656895787	gene-MA16_Dca012576
miR396b_3	0.656895787	gene-MA16_Dca003694
miR396b_3	0.656895787	gene-MA16_Dca004139
miR396b_3	0.656895787	gene-MA16_Dca014631
miR396b_3	0.656895787	gene-MA16_Dca021860
miR396b_3	0.656895787	gene-MA16_Dca018722
miR396b_3	0.656895787	gene-MA16_Dca008028
miR396b_3	0.656895787	gene-MA16_Dca004158
miR396b_3	0.656895787	gene-MA16_Dca023002
miR396b_3	0.656895787	gene-MA16_Dca004133
miR396b_3	0.656895787	gene-MA16_Dca025782
miR396b_3	0.656895787	gene-MA16_Dca024909
miR396b_3	0.656895787	gene-MA16_Dca022287
miR396b_3	0.656895787	gene-MA16_Dca016598
miR396b_3	0.656895787	gene-MA16_Dca014260
miR396b_3	0.656895787	gene-MA16_Dca001930
miR396b_3	0.656895787	gene-MA16_Dca014266
miR396b_3	0.656895787	gene-MA16_Dca017977
miR396b_3	0.656895787	gene-MA16_Dca007307
miR396b_3	0.656895787	gene-MA16_Dca005596
miR396b_3	0.656895787	gene-MA16_Dca016354
miR396b_3	0.656895787	gene-MA16_Dca013418
miR396b_3	0.656895787	gene-MA16_Dca005154
miR396b_3	0.656895787	gene-MA16_Dca011751
miR396b_3	0.656895787	gene-MA16_Dca015263
miR396b_3	0.656895787	gene-MA16_Dca011523
miR396b_3	0.656895787	gene-MA16_Dca011783
miR396b_3	0.656895787	gene-MA16_Dca005151
miR396b_3	0.656895787	gene-MA16_Dca011967
miR396b_3	0.656895787	gene-MA16_Dca008222
miR396b_3	0.656895787	gene-MA16_Dca007694
miR396b_3	0.656895787	gene-MA16_Dca000885
miR396b_3	0.656895787	gene-MA16_Dca006666
miR396b_3	0.656895787	gene-MA16_Dca020166
miR396b_3	0.656895787	gene-MA16_Dca018345
miR396b_3	0.656895787	gene-MA16_Dca002241
miR396b_3	0.656895787	gene-MA16_Dca019707

miR396b_3	0.656895787	gene-MA16_Dca012938
miR396b_3	0.656895787	gene-MA16_Dca020801
miR396b_3	0.656895787	gene-MA16_Dca007955
miR396b_3	0.656895787	gene-MA16_Dca022302
miR396b_3	0.656895787	gene-MA16_Dca005617
miR396b_3	0.656895787	gene-MA16_Dca000619
miR396b_3	0.656895787	gene-MA16_Dca002697
miR396b_3	0.656895787	gene-MA16_Dca024737
miR396b_3	0.656895787	gene-MA16_Dca019222
miR396b_3	0.656895787	gene-MA16_Dca014751
miR396b_3	0.656895787	gene-MA16_Dca023601
miR396b_3	0.656895787	gene-MA16_Dca004403
miR396b_3	0.656895787	gene-MA16_Dca024281
miR396b_3	0.656895787	gene-MA16_Dca015409
miR396b_3	0.656895787	gene-MA16_Dca025223
miR396b_3	0.656895787	gene-MA16_Dca009226
miR396b_3	0.656895787	gene-MA16_Dca013322
miR396b_3	0.656895787	gene-MA16_Dca016956
miR396b_3	0.656895787	gene-MA16_Dca011603
miR396b_3	0.656895787	gene-MA16_Dca007949
miR396b_3	0.656895787	gene-MA16_Dca020207
miR396b_3	0.656895787	gene-MA16_Dca021446
miR396b_3	0.656895787	gene-MA16_Dca024988
miR396b_3	0.656895787	gene-MA16_Dca003177
miR396b_3	0.656895787	gene-MA16_Dca027492
miR396b_3	0.656895787	gene-MA16_Dca011959
miR396b_3	0.656895787	gene-MA16_Dca011627
miR396b_3	0.656895787	gene-MA16_Dca020910
miR396b_3	0.656895787	gene-MA16_Dca004692
miR396b_3	0.656895787	gene-MA16_Dca010708
miR396b_3	0.656895787	gene-MA16_Dca012099
miR396b_3	0.656895787	gene-MA16_Dca011382
miR396b_3	0.656895787	gene-MA16_Dca025262
miR396b_3	0.656895787	gene-MA16_Dca028471
miR396b_3	0.656895787	gene-MA16_Dca003911
miR396b_2	0.652023813	gene-MA16_Dca004093
miR396b_2	0.652023813	gene-MA16_Dca002377
miR396b_2	0.652023813	gene-MA16_Dca006179
miR396b_2	0.652023813	gene-MA16_Dca001199
miR396b_2	0.652023813	gene-MA16_Dca001936
miR396b_2	0.652023813	gene-MA16_Dca024442
miR396b_2	0.652023813	gene-MA16_Dca001785
miR396b_2	0.652023813	gene-MA16_Dca002242
miR396b_2	0.652023813	gene-MA16_Dca011294
miR396b_2	0.652023813	gene-MA16_Dca010163
miR396b_2	0.652023813	gene-MA16_Dca010619

miR396b_2	0.652023813	gene-MA16_Dca020583
miR396b_2	0.652023813	gene-MA16_Dca001955
miR396b_2	0.652023813	gene-MA16_Dca012128
miR396b_2	0.652023813	gene-MA16_Dca026927
miR396b_2	0.652023813	gene-MA16_Dca006631
miR396b_2	0.652023813	gene-MA16_Dca021850
miR396b_2	0.652023813	gene-MA16_Dca000152
miR396b_2	0.652023813	gene-MA16_Dca022211
miR396b_2	0.652023813	gene-MA16_Dca010315
miR396b_2	0.652023813	gene-MA16_Dca023840
miR396b_2	0.652023813	gene-MA16_Dca006059
miR396b_2	0.652023813	gene-MA16_Dca001920
miR396b_2	0.652023813	gene-MA16_Dca018172
miR396b_2	0.652023813	gene-MA16_Dca024581
miR396b_2	0.652023813	gene-MA16_Dca012093
miR396b_2	0.652023813	gene-MA16_Dca000754
miR396b_2	0.652023813	gene-MA16_Dca018548
miR396b_2	0.652023813	gene-MA16_Dca013497
miR396b_2	0.652023813	gene-MA16_Dca023379
miR396b_2	0.652023813	gene-MA16_Dca019943
miR396b_2	0.652023813	gene-MA16_Dca006898
miR396b_2	0.652023813	gene-MA16_Dca010222
miR396b_2	0.652023813	gene-MA16_Dca019153
miR396b_2	0.652023813	gene-MA16_Dca019207
miR396b_2	0.652023813	gene-MA16_Dca008028
miR396b_2	0.652023813	gene-MA16_Dca004158
miR396b_2	0.652023813	gene-MA16_Dca023002
miR396b_2	0.652023813	gene-MA16_Dca004133
miR396b_2	0.652023813	gene-MA16_Dca025782
miR396b_2	0.652023813	gene-MA16_Dca024909
miR396b_2	0.652023813	gene-MA16_Dca023691
miR396b_2	0.652023813	gene-MA16_Dca018725
miR396b_2	0.652023813	gene-MA16_Dca005799
miR396b_2	0.652023813	gene-MA16_Dca021863
miR396b_2	0.652023813	gene-MA16_Dca022508
miR396b_2	0.652023813	gene-MA16_Dca003694
miR396b_2	0.652023813	gene-MA16_Dca012576
miR396b_2	0.652023813	gene-MA16_Dca014631
miR396b_2	0.652023813	gene-MA16_Dca004139
miR396b_2	0.652023813	gene-MA16_Dca021860
miR396b_2	0.652023813	gene-MA16_Dca018722
miR396b_2	0.652023813	gene-MA16_Dca014266
miR396b_2	0.652023813	gene-MA16_Dca017977
miR396b_2	0.652023813	gene-MA16_Dca007307
miR396b_2	0.652023813	gene-MA16_Dca016354
miR396b_2	0.652023813	gene-MA16_Dca005596

miR396b_2	0.652023813	gene-MA16_Dca013418
miR396b_2	0.652023813	gene-MA16_Dca022287
miR396b_2	0.652023813	gene-MA16_Dca016598
miR396b_2	0.652023813	gene-MA16_Dca014260
miR396b_2	0.652023813	gene-MA16_Dca001930
miR396b_2	0.652023813	gene-MA16_Dca011523
miR396b_2	0.652023813	gene-MA16_Dca011783
miR396b_2	0.652023813	gene-MA16_Dca011967
miR396b_2	0.652023813	gene-MA16_Dca005151
miR396b_2	0.652023813	gene-MA16_Dca008222
miR396b_2	0.652023813	gene-MA16_Dca007694
miR396b_2	0.652023813	gene-MA16_Dca000885
miR396b_2	0.652023813	gene-MA16_Dca006666
miR396b_2	0.652023813	gene-MA16_Dca005154
miR396b_2	0.652023813	gene-MA16_Dca011751
miR396b_2	0.652023813	gene-MA16_Dca015263
miR396b_2	0.652023813	gene-MA16_Dca007955
miR396b_2	0.652023813	gene-MA16_Dca005617
miR396b_2	0.652023813	gene-MA16_Dca000619
miR396b_2	0.652023813	gene-MA16_Dca022302
miR396b_2	0.652023813	gene-MA16_Dca002697
miR396b_2	0.652023813	gene-MA16_Dca019222
miR396b_2	0.652023813	gene-MA16_Dca024737
miR396b_2	0.652023813	gene-MA16_Dca014751
miR396b_2	0.652023813	gene-MA16_Dca023601
miR396b_2	0.652023813	gene-MA16_Dca004403
miR396b_2	0.652023813	gene-MA16_Dca020166
miR396b_2	0.652023813	gene-MA16_Dca018345
miR396b_2	0.652023813	gene-MA16_Dca002241
miR396b_2	0.652023813	gene-MA16_Dca019707
miR396b_2	0.652023813	gene-MA16_Dca012938
miR396b_2	0.652023813	gene-MA16_Dca020801
miR396b_2	0.652023813	gene-MA16_Dca011603
miR396b_2	0.652023813	gene-MA16_Dca016956
miR396b_2	0.652023813	gene-MA16_Dca007949
miR396b_2	0.652023813	gene-MA16_Dca020207
miR396b_2	0.652023813	gene-MA16_Dca021446
miR396b_2	0.652023813	gene-MA16_Dca024281
miR396b_2	0.652023813	gene-MA16_Dca015409
miR396b_2	0.652023813	gene-MA16_Dca025223
miR396b_2	0.652023813	gene-MA16_Dca009226
miR396b_2	0.652023813	gene-MA16_Dca013322
miR396b_2	0.652023813	gene-MA16_Dca011382
miR396b_2	0.652023813	gene-MA16_Dca025262
miR396b_2	0.652023813	gene-MA16_Dca028471
miR396b_2	0.652023813	gene-MA16_Dca003911

miR396b_2	0.652023813	gene-MA16_Dca024988
miR396b_2	0.652023813	gene-MA16_Dca003177
miR396b_2	0.652023813	gene-MA16_Dca027492
miR396b_2	0.652023813	gene-MA16_Dca011627
miR396b_2	0.652023813	gene-MA16_Dca011959
miR396b_2	0.652023813	gene-MA16_Dca020910
miR396b_2	0.652023813	gene-MA16_Dca004692
miR396b_2	0.652023813	gene-MA16_Dca010708
miR396b_2	0.652023813	gene-MA16_Dca012099
novel_miR_242	-0.699231768	gene-MA16_Dca015938
novel_miR_242	-0.699231768	gene-MA16_Dca002874
novel_miR_242	-0.699231768	gene-MA16_Dca020985
novel_miR_242	-0.699231768	gene-MA16_Dca006366
novel_miR_242	-0.699231768	gene-MA16_Dca013024
novel_miR_242	-0.699231768	gene-MA16_Dca023978
novel_miR_242	-0.699231768	gene-MA16_Dca003396
miR172d_1	0.969357365	gene-MA16_Dca021336
miR172d_1	0.969357365	gene-MA16_Dca022520
miR172d_1	0.969357365	gene-MA16_Dca006530
miR172d_1	0.969357365	gene-MA16_Dca016851
miR172d_1	0.969357365	gene-MA16_Dca022452
miR172d_1	0.969357365	gene-MA16_Dca013460
miR172d_1	0.969357365	gene-MA16_Dca022454
miR172d_1	0.969357365	gene-MA16_Dca009661
miR172d_1	0.969357365	gene-MA16_Dca013779
miR172d_1	0.969357365	gene-MA16_Dca008031
miR172d_1	0.969357365	gene-MA16_Dca002934
miR172d_1	0.969357365	gene-MA16_Dca022453
miR172d_1	0.969357365	gene-MA16_Dca020018
miR172d_1	0.969357365	gene-MA16_Dca000260
miR172d_1	0.969357365	gene-MA16_Dca010160
miR172d_1	0.969357365	gene-MA16_Dca017222
miR172d_1	0.969357365	gene-MA16_Dca002039
miR172d_1	0.969357365	gene-MA16_Dca018756
miR172d_1	0.969357365	gene-MA16_Dca013337
miR172d_1	0.969357365	gene-MA16_Dca016049
miR172d_1	0.969357365	gene-MA16_Dca015839
miR172d_1	0.969357365	gene-MA16_Dca020825
miR172d_1	0.969357365	gene-MA16_Dca020826
miR172d_1	0.969357365	gene-MA16_Dca018027
miR172d_1	0.969357365	gene-MA16_Dca020870
miR172d_1	0.969357365	gene-MA16_Dca020793
miR172d_1	0.969357365	gene-MA16_Dca011850
miR172d_1	0.969357365	gene-MA16_Dca001782
miR172d_1	0.969357365	gene-MA16_Dca013902
miR172d_1	0.969357365	gene-MA16_Dca018995

miR172d_1	0.969357365	gene-MA16_Dca004101
miR172d_1	0.969357365	gene-MA16_Dca017910
miR172d_1	0.969357365	gene-MA16_Dca007639
miR172d_1	0.969357365	gene-MA16_Dca012666
miR172d_1	0.969357365	gene-MA16_Dca022455
miR172d_2	0.969375799	gene-MA16_Dca010160
miR172d_2	0.969375799	gene-MA16_Dca017222
miR172d_2	0.969375799	gene-MA16_Dca002039
miR172d_2	0.969375799	gene-MA16_Dca018756
miR172d_2	0.969375799	gene-MA16_Dca008031
miR172d_2	0.969375799	gene-MA16_Dca020018
miR172d_2	0.969375799	gene-MA16_Dca000260
miR172d_2	0.969375799	gene-MA16_Dca002934
miR172d_2	0.969375799	gene-MA16_Dca022453
miR172d_2	0.969375799	gene-MA16_Dca009661
miR172d_2	0.969375799	gene-MA16_Dca013779
miR172d_2	0.969375799	gene-MA16_Dca022454
miR172d_2	0.969375799	gene-MA16_Dca021336
miR172d_2	0.969375799	gene-MA16_Dca022520
miR172d_2	0.969375799	gene-MA16_Dca016851
miR172d_2	0.969375799	gene-MA16_Dca013460
miR172d_2	0.969375799	gene-MA16_Dca022452
miR172d_2	0.969375799	gene-MA16_Dca006530
miR172d_2	0.969375799	gene-MA16_Dca017910
miR172d_2	0.969375799	gene-MA16_Dca022455
miR172d_2	0.969375799	gene-MA16_Dca007639
miR172d_2	0.969375799	gene-MA16_Dca012666
miR172d_2	0.969375799	gene-MA16_Dca004101
miR172d_2	0.969375799	gene-MA16_Dca001782
miR172d_2	0.969375799	gene-MA16_Dca018995
miR172d_2	0.969375799	gene-MA16_Dca013902
miR172d_2	0.969375799	gene-MA16_Dca020870
miR172d_2	0.969375799	gene-MA16_Dca020793
miR172d_2	0.969375799	gene-MA16_Dca011850
miR172d_2	0.969375799	gene-MA16_Dca016049
miR172d_2	0.969375799	gene-MA16_Dca015839
miR172d_2	0.969375799	gene-MA16_Dca013337
miR172d_2	0.969375799	gene-MA16_Dca018027
miR172d_2	0.969375799	gene-MA16_Dca020826
miR172d_2	0.969375799	gene-MA16_Dca020825
miR172k	1.219742133	gene-MA16_Dca013337
miR172k	1.219742133	gene-MA16_Dca016049
miR172k	1.219742133	gene-MA16_Dca021336
miR172k	1.219742133	gene-MA16_Dca006530
miR172k	1.219742133	gene-MA16_Dca020825
miR172k	1.219742133	gene-MA16_Dca016851

miR172k	1.219742133	gene-MA16_Dca018027
miR172k	1.219742133	gene-MA16_Dca020826
miR172k	1.219742133	gene-MA16_Dca005551
miR172k	1.219742133	gene-MA16_Dca012560
miR172k	1.219742133	gene-MA16_Dca002321
miR172k	1.219742133	gene-MA16_Dca022451
miR172k	1.219742133	gene-MA16_Dca003098
miR172k	1.219742133	gene-MA16_Dca022454
miR172k	1.219742133	gene-MA16_Dca009661
miR172k	1.219742133	gene-MA16_Dca008031
miR172k	1.219742133	gene-MA16_Dca000603
miR172k	1.219742133	gene-MA16_Dca018703
miR172k	1.219742133	gene-MA16_Dca013902
miR172k	1.219742133	gene-MA16_Dca013886
miR172k	1.219742133	gene-MA16_Dca017910
miR172k	1.219742133	gene-MA16_Dca022962
miR172k	1.219742133	gene-MA16_Dca005153
miR172k	1.219742133	gene-MA16_Dca012598
miR172k	1.219742133	gene-MA16_Dca002039
miR172k	1.219742133	gene-MA16_Dca010160
miR172k	1.219742133	gene-MA16_Dca018756
miR172k	1.219742133	gene-MA16_Dca001078

Note: FC indicate TPM value of miRNA in FR2/TPM value of miRNA in CK
