# Supplementary material for: RNA sequencing-based exploration of the effects of far-red light on microRNAs involved in the shade-avoidance response of D. officinale
Source: PeerJ. 2023 Mar 20;11:e15001. doi: 10.7717/peerj.15001 (PMC10035421; doi:10.7717/peerj.15001)
Supplement: Table S8 [file peerj-11-15001-s008.pdf]

Table S8 Interaction miRNAs and mRNAs of FR8-CK

miRNA ID	log_2FC	Targets
miR395m	-3.257725112	gene-MA16_Dca017889
miR395m	-3.257725112	gene-MA16_Dca007732
miR395m	-3.257725112	gene-MA16_Dca021033
miR395m	-3.257725112	gene-MA16_Dca007646
miR395m	-3.257725112	gene-MA16_Dca026271
miR395m	-3.257725112	gene-MA16_Dca003297
miR395m	-3.257725112	gene-MA16_Dca025511
miR395m	-3.257725112	gene-MA16_Dca001411
miR395m	-3.257725112	gene-MA16_Dca007731
miR395m	-3.257725112	gene-MA16_Dca004386
miR395m	-3.257725112	gene-MA16_Dca009355
miR395m	-3.257725112	gene-MA16_Dca003285
miR395m	-3.257725112	gene-MA16_Dca005635
miR395m	-3.257725112	gene-MA16_Dca011968
miR395b	-3.726049807	gene-MA16_Dca015429
miR395b	-3.726049807	gene-MA16_Dca003297
miR395b	-3.726049807	gene-MA16_Dca007732
miR395b	-3.726049807	gene-MA16_Dca011968
miR395b	-3.726049807	gene-MA16_Dca003285
miR395b	-3.726049807	gene-MA16_Dca007731
miR395b	-3.726049807	gene-MA16_Dca004386
miR395b	-3.726049807	gene-MA16_Dca009355
miR395b	-3.726049807	gene-MA16_Dca019636
miR395b	-3.726049807	gene-MA16_Dca001411
miR395b	-3.726049807	gene-MA16_Dca025511
miR399t_3p	3.606930859	gene-MA16_Dca021268
miR399t_3p	3.606930859	gene-MA16_Dca011813
miR399t_3p	3.606930859	gene-MA16_Dca008399
miR399t_3p	3.606930859	gene-MA16_Dca002672
miR399t_3p	3.606930859	gene-MA16_Dca015271
miR399t_3p	3.606930859	gene-MA16_Dca008935
miR399t_3p	3.606930859	gene-MA16_Dca022711

miR399t_3p	3.606930859	gene-MA16_Dca008220
miR399t_3p	3.606930859	gene-MA16_Dca008496
miR399t_3p	3.606930859	gene-MA16_Dca021979
miR399t_3p	3.606930859	gene-MA16_Dca016176
novel_miR_189	-2.71785953	gene-MA16_Dca023326
novel_miR_189	-2.71785953	gene-MA16_Dca011459
novel_miR_189	-2.71785953	gene-MA16_Dca010804
novel_miR_189	-2.71785953	gene-MA16_Dca024400
novel_miR_189	-2.71785953	gene-MA16_Dca022787
novel_miR_189	-2.71785953	gene-MA16_Dca025882
novel_miR_189	-2.71785953	gene-MA16_Dca015722
novel_miR_189	-2.71785953	gene-MA16_Dca014786
novel_miR_189	-2.71785953	gene-MA16_Dca026995
novel_miR_189	-2.71785953	gene-MA16_Dca004836
novel_miR_189	-2.71785953	gene-MA16_Dca001649
novel_miR_189	-2.71785953	gene-MA16_Dca018219
novel_miR_189	-2.71785953	gene-MA16_Dca010516
novel_miR_189	-2.71785953	gene-MA16_Dca024704
novel_miR_189	-2.71785953	gene-MA16_Dca024902
novel_miR_189	-2.71785953	gene-MA16_Dca001622
novel_miR_189	-2.71785953	gene-MA16_Dca021278
novel_miR_189	-2.71785953	gene-MA16_Dca022838
novel_miR_189	-2.71785953	gene-MA16_Dca022812
novel_miR_189	-2.71785953	gene-MA16_Dca005118
novel_miR_189	-2.71785953	gene-MA16_Dca011344
novel_miR_189	-2.71785953	gene-MA16_Dca017709
novel_miR_189	-2.71785953	gene-MA16_Dca018325
novel_miR_189	-2.71785953	gene-MA16_Dca003816
novel_miR_189	-2.71785953	gene-MA16_Dca014554
novel_miR_189	-2.71785953	gene-MA16_Dca018212
novel_miR_189	-2.71785953	gene-MA16_Dca010241
novel_miR_189	-2.71785953	gene-MA16_Dca005791
novel_miR_189	-2.71785953	gene-MA16_Dca002499
novel_miR_189	-2.71785953	gene-MA16_Dca013892

novel_miR_189	-2.71785953	gene-MA16_Dca000024
novel_miR_189	-2.71785953	gene-MA16_Dca009944
novel_miR_189	-2.71785953	gene-MA16_Dca003956
novel_miR_189	-2.71785953	gene-MA16_Dca028126
novel_miR_189	-2.71785953	gene-MA16_Dca024385
novel_miR_189	-2.71785953	gene-MA16_Dca014761
novel_miR_189	-2.71785953	gene-MA16_Dca021441
novel_miR_189	-2.71785953	gene-MA16_Dca009034
novel_miR_189	-2.71785953	gene-MA16_Dca003052
novel_miR_189	-2.71785953	gene-MA16_Dca027145
novel_miR_189	-2.71785953	gene-MA16_Dca014804
novel_miR_189	-2.71785953	gene-MA16_Dca021649
novel_miR_189	-2.71785953	gene-MA16_Dca010408
novel_miR_189	-2.71785953	gene-MA16_Dca011938
novel_miR_189	-2.71785953	gene-MA16_Dca003122
novel_miR_189	-2.71785953	gene-MA16_Dca010402
novel_miR_189	-2.71785953	gene-MA16_Dca016865
novel_miR_189	-2.71785953	gene-MA16_Dca011525
novel_miR_189	-2.71785953	gene-MA16_Dca001400
novel_miR_189	-2.71785953	gene-MA16_Dca007799
novel_miR_189	-2.71785953	gene-MA16_Dca010456
novel_miR_189	-2.71785953	gene-MA16_Dca013722
novel_miR_189	-2.71785953	gene-MA16_Dca005175
novel_miR_189	-2.71785953	gene-MA16_Dca008000
novel_miR_189	-2.71785953	gene-MA16_Dca013243
novel_miR_189	-2.71785953	gene-MA16_Dca026494
novel_miR_390	-3.669012947	gene-MA16_Dca014079
novel_miR_390	-3.669012947	gene-MA16_Dca010045
novel_miR_390	-3.669012947	gene-MA16_Dca016601
novel_miR_390	-3.669012947	gene-MA16_Dca028500
novel_miR_390	-3.669012947	gene-MA16_Dca008125
novel_miR_390	-3.669012947	gene-MA16_Dca010799
novel_miR_390	-3.669012947	gene-MA16_Dca028410
novel_miR_390	-3.669012947	gene-MA16_Dca018794

novel_miR_390	-3.669012947	gene-MA16_Dca005769
novel_miR_390	-3.669012947	gene-MA16_Dca003585
novel_miR_390	-3.669012947	gene-MA16_Dca021544
novel_miR_390	-3.669012947	gene-MA16_Dca013966
novel_miR_390	-3.669012947	gene-MA16_Dca014033
novel_miR_390	-3.669012947	gene-MA16_Dca027734
novel_miR_390	-3.669012947	gene-MA16_Dca014296
novel_miR_390	-3.669012947	gene-MA16_Dca024277
novel_miR_390	-3.669012947	gene-MA16_Dca011847
novel_miR_390	-3.669012947	gene-MA16_Dca013703
novel_miR_390	-3.669012947	gene-MA16_Dca014007
novel_miR_390	-3.669012947	gene-MA16_Dca014915
novel_miR_390	-3.669012947	gene-MA16_Dca004540
novel_miR_390	-3.669012947	gene-MA16_Dca011199
novel_miR_390	-3.669012947	gene-MA16_Dca022649
novel_miR_390	-3.669012947	gene-MA16_Dca018160
novel_miR_390	-3.669012947	gene-MA16_Dca002338
novel_miR_390	-3.669012947	gene-MA16_Dca015057
novel_miR_390	-3.669012947	gene-MA16_Dca016490
novel_miR_390	-3.669012947	gene-MA16_Dca025504
novel_miR_390	-3.669012947	gene-MA16_Dca016699
novel_miR_390	-3.669012947	gene-MA16_Dca014673
novel_miR_390	-3.669012947	gene-MA16_Dca022905
novel_miR_390	-3.669012947	gene-MA16_Dca015359
novel_miR_390	-3.669012947	gene-MA16_Dca011253
novel_miR_390	-3.669012947	gene-MA16_Dca014237
novel_miR_390	-3.669012947	gene-MA16_Dca021241
novel_miR_390	-3.669012947	gene-MA16_Dca000483
novel_miR_390	-3.669012947	gene-MA16_Dca009990
novel_miR_390	-3.669012947	gene-MA16_Dca005346
novel_miR_390	-3.669012947	gene-MA16_Dca022795
novel_miR_390	-3.669012947	gene-MA16_Dca008661
novel_miR_390	-3.669012947	gene-MA16_Dca017160
novel_miR_390	-3.669012947	gene-MA16_Dca022638

novel_miR_390	-3.669012947	gene-MA16_Dca013961
novel_miR_390	-3.669012947	gene-MA16_Dca010093
novel_miR_390	-3.669012947	gene-MA16_Dca002089
novel_miR_390	-3.669012947	gene-MA16_Dca021165
novel_miR_390	-3.669012947	gene-MA16_Dca004503
novel_miR_390	-3.669012947	gene-MA16_Dca007957
novel_miR_390	-3.669012947	gene-MA16_Dca020534
novel_miR_390	-3.669012947	gene-MA16_Dca004340
novel_miR_390	-3.669012947	gene-MA16_Dca017598
novel_miR_390	-3.669012947	gene-MA16_Dca012435
novel_miR_390	-3.669012947	gene-MA16_Dca007417
novel_miR_390	-3.669012947	gene-MA16_Dca010932
novel_miR_390	-3.669012947	gene-MA16_Dca023407
novel_miR_390	-3.669012947	gene-MA16_Dca002781
novel_miR_390	-3.669012947	gene-MA16_Dca008350
novel_miR_390	-3.669012947	gene-MA16_Dca002133
novel_miR_390	-3.669012947	gene-MA16_Dca015811
novel_miR_390	-3.669012947	gene-MA16_Dca023276
novel_miR_262	-3.668771987	gene-MA16_Dca023276
novel_miR_262	-3.668771987	gene-MA16_Dca015811
novel_miR_262	-3.668771987	gene-MA16_Dca002133
novel_miR_262	-3.668771987	gene-MA16_Dca002781
novel_miR_262	-3.668771987	gene-MA16_Dca008350
novel_miR_262	-3.668771987	gene-MA16_Dca007417
novel_miR_262	-3.668771987	gene-MA16_Dca010932
novel_miR_262	-3.668771987	gene-MA16_Dca023407
novel_miR_262	-3.668771987	gene-MA16_Dca012435
novel_miR_262	-3.668771987	gene-MA16_Dca017598
novel_miR_262	-3.668771987	gene-MA16_Dca004340
novel_miR_262	-3.668771987	gene-MA16_Dca020534
novel_miR_262	-3.668771987	gene-MA16_Dca007957
novel_miR_262	-3.668771987	gene-MA16_Dca004503
novel_miR_262	-3.668771987	gene-MA16_Dca021165
novel_miR_262	-3.668771987	gene-MA16_Dca002089

novel_miR_262	-3.668771987	gene-MA16_Dca010093
novel_miR_262	-3.668771987	gene-MA16_Dca013961
novel_miR_262	-3.668771987	gene-MA16_Dca022638
novel_miR_262	-3.668771987	gene-MA16_Dca017160
novel_miR_262	-3.668771987	gene-MA16_Dca008661
novel_miR_262	-3.668771987	gene-MA16_Dca022795
novel_miR_262	-3.668771987	gene-MA16_Dca005346
novel_miR_262	-3.668771987	gene-MA16_Dca009990
novel_miR_262	-3.668771987	gene-MA16_Dca000483
novel_miR_262	-3.668771987	gene-MA16_Dca021241
novel_miR_262	-3.668771987	gene-MA16_Dca014237
novel_miR_262	-3.668771987	gene-MA16_Dca011253
novel_miR_262	-3.668771987	gene-MA16_Dca015359
novel_miR_262	-3.668771987	gene-MA16_Dca022905
novel_miR_262	-3.668771987	gene-MA16_Dca014673
novel_miR_262	-3.668771987	gene-MA16_Dca016699
novel_miR_262	-3.668771987	gene-MA16_Dca025504
novel_miR_262	-3.668771987	gene-MA16_Dca016490
novel_miR_262	-3.668771987	gene-MA16_Dca015057
novel_miR_262	-3.668771987	gene-MA16_Dca002338
novel_miR_262	-3.668771987	gene-MA16_Dca018160
novel_miR_262	-3.668771987	gene-MA16_Dca011199
novel_miR_262	-3.668771987	gene-MA16_Dca004540
novel_miR_262	-3.668771987	gene-MA16_Dca022649
novel_miR_262	-3.668771987	gene-MA16_Dca014007
novel_miR_262	-3.668771987	gene-MA16_Dca014915
novel_miR_262	-3.668771987	gene-MA16_Dca011847
novel_miR_262	-3.668771987	gene-MA16_Dca013703
novel_miR_262	-3.668771987	gene-MA16_Dca024277
novel_miR_262	-3.668771987	gene-MA16_Dca027734
novel_miR_262	-3.668771987	gene-MA16_Dca014296
novel_miR_262	-3.668771987	gene-MA16_Dca014033
novel_miR_262	-3.668771987	gene-MA16_Dca013966
novel_miR_262	-3.668771987	gene-MA16_Dca003585

novel_miR_262	-3.668771987	gene-MA16_Dca021544
novel_miR_262	-3.668771987	gene-MA16_Dca005769
novel_miR_262	-3.668771987	gene-MA16_Dca018794
novel_miR_262	-3.668771987	gene-MA16_Dca028410
novel_miR_262	-3.668771987	gene-MA16_Dca010799
novel_miR_262	-3.668771987	gene-MA16_Dca028500
novel_miR_262	-3.668771987	gene-MA16_Dca008125
novel_miR_262	-3.668771987	gene-MA16_Dca016601
novel_miR_262	-3.668771987	gene-MA16_Dca010045
novel_miR_262	-3.668771987	gene-MA16_Dca014079
miR399b_2	2.072480945	gene-MA16_Dca001351
miR399b_2	2.072480945	gene-MA16_Dca003017
miR399b_2	2.072480945	gene-MA16_Dca018409
novel_miR_264	1.037907144	gene-MA16_Dca017476
novel_miR_264	1.037907144	gene-MA16_Dca018659
novel_miR_264	1.037907144	gene-MA16_Dca019290
novel_miR_264	1.037907144	gene-MA16_Dca015518
miR827	2.205017066	gene-MA16_Dca021565
miR827	2.205017066	gene-MA16_Dca024417
miR827	2.205017066	gene-MA16_Dca002484
miR827	2.205017066	gene-MA16_Dca008822
miR827	2.205017066	gene-MA16_Dca024114
miR827	2.205017066	gene-MA16_Dca022005
miR399c_5p	1.954215127	gene-MA16_Dca002003
miR399c_5p	1.954215127	gene-MA16_Dca019401
miR399c_5p	1.954215127	gene-MA16_Dca023610
miR399c_5p	1.954215127	gene-MA16_Dca006616
miR399c_5p	1.954215127	gene-MA16_Dca023997
miR399c_5p	1.954215127	gene-MA16_Dca025118
miR399c_5p	1.954215127	gene-MA16_Dca005971
miR399c_5p	1.954215127	gene-MA16_Dca027017
miR399c_5p	1.954215127	gene-MA16_Dca022511
miR399c_5p	1.954215127	gene-MA16_Dca021261
miR399c_5p	1.954215127	gene-MA16_Dca016271

miR399c_5p	1.954215127	gene-MA16_Dca000468
miR399c_5p	1.954215127	gene-MA16_Dca006711
miR399c_5p	1.954215127	gene-MA16_Dca025768
miR399c_5p	1.954215127	gene-MA16_Dca020196
miR399c_5p	1.954215127	gene-MA16_Dca005699
miR399c_5p	1.954215127	gene-MA16_Dca019405
miR399c_5p	1.954215127	gene-MA16_Dca007810
miR399c_5p	1.954215127	gene-MA16_Dca010441
miR399c_5p	1.954215127	gene-MA16_Dca024450
miR399c_5p	1.954215127	gene-MA16_Dca026041
miR399c_5p	1.954215127	gene-MA16_Dca016402
miR399c_5p	1.954215127	gene-MA16_Dca015690
miR399c_5p	1.954215127	gene-MA16_Dca028386
miR399c_5p	1.954215127	gene-MA16_Dca000976
miR399c_5p	1.954215127	gene-MA16_Dca004695
miR399c_5p	1.954215127	gene-MA16_Dca003355
miR399c_5p	1.954215127	gene-MA16_Dca018337
miR399c_5p	1.954215127	gene-MA16_Dca001276
miR399c_5p	1.954215127	gene-MA16_Dca018325
miR399c_5p	1.954215127	gene-MA16_Dca018237
miR399c_5p	1.954215127	gene-MA16_Dca001986
miR399c_5p	1.954215127	gene-MA16_Dca025044
miR399c_5p	1.954215127	gene-MA16_Dca002055
miR399c_5p	1.954215127	gene-MA16_Dca006221
miR399c_5p	1.954215127	gene-MA16_Dca011500
miR399c_5p	1.954215127	gene-MA16_Dca012031
miR399c_5p	1.954215127	gene-MA16_Dca022465
miR399c_5p	1.954215127	gene-MA16_Dca009831
miR399c_5p	1.954215127	gene-MA16_Dca026145
miR399c_5p	1.954215127	gene-MA16_Dca021763
novel_miR_483	1.363671654	gene-MA16_Dca001585
novel_miR_483	1.363671654	gene-MA16_Dca008798
novel_miR_483	1.363671654	gene-MA16_Dca011586
novel_miR_483	1.363671654	gene-MA16_Dca008229

novel_miR_483	1.363671654	gene-MA16_Dca001748
novel_miR_483	1.363671654	gene-MA16_Dca004547
novel_miR_483	1.363671654	gene-MA16_Dca024638
novel_miR_483	1.363671654	gene-MA16_Dca008180
novel_miR_483	1.363671654	gene-MA16_Dca012903
novel_miR_483	1.363671654	gene-MA16_Dca023073
novel_miR_483	1.363671654	gene-MA16_Dca001413
novel_miR_483	1.363671654	gene-MA16_Dca003302
novel_miR_483	1.363671654	gene-MA16_Dca014470
novel_miR_483	1.363671654	gene-MA16_Dca002554
novel_miR_483	1.363671654	gene-MA16_Dca021949
novel_miR_483	1.363671654	gene-MA16_Dca000383
novel_miR_483	1.363671654	gene-MA16_Dca017330
novel_miR_483	1.363671654	gene-MA16_Dca019516
novel_miR_483	1.363671654	gene-MA16_Dca014566
novel_miR_483	1.363671654	gene-MA16_Dca021950
novel_miR_483	1.363671654	gene-MA16_Dca023983
novel_miR_483	1.363671654	gene-MA16_Dca022074
novel_miR_483	1.363671654	gene-MA16_Dca012104
novel_miR_483	1.363671654	gene-MA16_Dca011997
novel_miR_483	1.363671654	gene-MA16_Dca013128
novel_miR_483	1.363671654	gene-MA16_Dca010674
novel_miR_483	1.363671654	gene-MA16_Dca007315
novel_miR_483	1.363671654	gene-MA16_Dca015910
novel_miR_484	-2.721668558	gene-MA16_Dca013703
novel_miR_484	-2.721668558	gene-MA16_Dca011847
novel_miR_484	-2.721668558	gene-MA16_Dca028665
novel_miR_484	-2.721668558	gene-MA16_Dca022384
novel_miR_484	-2.721668558	gene-MA16_Dca018928
novel_miR_484	-2.721668558	gene-MA16_Dca013338
novel_miR_484	-2.721668558	gene-MA16_Dca009144
novel_miR_484	-2.721668558	gene-MA16_Dca015180
novel_miR_484	-2.721668558	gene-MA16_Dca027734
novel_miR_484	-2.721668558	gene-MA16_Dca019875

novel_miR_484	-2.721668558	gene-MA16_Dca016458
novel_miR_484	-2.721668558	gene-MA16_Dca016490
novel_miR_484	-2.721668558	gene-MA16_Dca019561
novel_miR_484	-2.721668558	gene-MA16_Dca006494
novel_miR_484	-2.721668558	gene-MA16_Dca015083
novel_miR_484	-2.721668558	gene-MA16_Dca011199
novel_miR_484	-2.721668558	gene-MA16_Dca022649
novel_miR_484	-2.721668558	gene-MA16_Dca014915
novel_miR_484	-2.721668558	gene-MA16_Dca010212
novel_miR_484	-2.721668558	gene-MA16_Dca008125
novel_miR_484	-2.721668558	gene-MA16_Dca017780
novel_miR_484	-2.721668558	gene-MA16_Dca019194
novel_miR_484	-2.721668558	gene-MA16_Dca008674
novel_miR_484	-2.721668558	gene-MA16_Dca016409
novel_miR_484	-2.721668558	gene-MA16_Dca017451
novel_miR_484	-2.721668558	gene-MA16_Dca021955
novel_miR_484	-2.721668558	gene-MA16_Dca021243
novel_miR_484	-2.721668558	gene-MA16_Dca027391
novel_miR_484	-2.721668558	gene-MA16_Dca018115
novel_miR_484	-2.721668558	gene-MA16_Dca000965
novel_miR_484	-2.721668558	gene-MA16_Dca006776
novel_miR_484	-2.721668558	gene-MA16_Dca007667
novel_miR_484	-2.721668558	gene-MA16_Dca003224
novel_miR_484	-2.721668558	gene-MA16_Dca004503
novel_miR_484	-2.721668558	gene-MA16_Dca023412
novel_miR_484	-2.721668558	gene-MA16_Dca012030
novel_miR_484	-2.721668558	gene-MA16_Dca020389
novel_miR_484	-2.721668558	gene-MA16_Dca028340
novel_miR_484	-2.721668558	gene-MA16_Dca002133
novel_miR_484	-2.721668558	gene-MA16_Dca007417
novel_miR_484	-2.721668558	gene-MA16_Dca012435
novel_miR_484	-2.721668558	gene-MA16_Dca015045
novel_miR_484	-2.721668558	gene-MA16_Dca019567
novel_miR_484	-2.721668558	gene-MA16_Dca001581

novel_miR_484	-2.721668558	gene-MA16_Dca022185
novel_miR_484	-2.721668558	gene-MA16_Dca009281
novel_miR_484	-2.721668558	gene-MA16_Dca028824
novel_miR_484	-2.721668558	gene-MA16_Dca011516
novel_miR_484	-2.721668558	gene-MA16_Dca011253
novel_miR_484	-2.721668558	gene-MA16_Dca015359
novel_miR_484	-2.721668558	gene-MA16_Dca002926
novel_miR_484	-2.721668558	gene-MA16_Dca005762
novel_miR_484	-2.721668558	gene-MA16_Dca017160
novel_miR_484	-2.721668558	gene-MA16_Dca022638
novel_miR_484	-2.721668558	gene-MA16_Dca015933
novel_miR_484	-2.721668558	gene-MA16_Dca016882
novel_miR_484	-2.721668558	gene-MA16_Dca025576
novel_miR_484	-2.721668558	gene-MA16_Dca008661
novel_miR_484	-2.721668558	gene-MA16_Dca010144
novel_miR_484	-2.721668558	gene-MA16_Dca008955
novel_miR_484	-2.721668558	gene-MA16_Dca013055
novel_miR_484	-2.721668558	gene-MA16_Dca016304
novel_miR_484	-2.721668558	gene-MA16_Dca024277
novel_miR_484	-2.721668558	gene-MA16_Dca019975
novel_miR_484	-2.721668558	gene-MA16_Dca003651
novel_miR_484	-2.721668558	gene-MA16_Dca017446
novel_miR_484	-2.721668558	gene-MA16_Dca013631
novel_miR_484	-2.721668558	gene-MA16_Dca000685
novel_miR_484	-2.721668558	gene-MA16_Dca014673
novel_miR_484	-2.721668558	gene-MA16_Dca018194
novel_miR_484	-2.721668558	gene-MA16_Dca006033
novel_miR_484	-2.721668558	gene-MA16_Dca001538
novel_miR_484	-2.721668558	gene-MA16_Dca026681
novel_miR_484	-2.721668558	gene-MA16_Dca025504
novel_miR_484	-2.721668558	gene-MA16_Dca006006
novel_miR_484	-2.721668558	gene-MA16_Dca019891
novel_miR_484	-2.721668558	gene-MA16_Dca020179
novel_miR_484	-2.721668558	gene-MA16_Dca015057

novel_miR_484	-2.721668558	gene-MA16_Dca012781
novel_miR_484	-2.721668558	gene-MA16_Dca004540
novel_miR_484	-2.721668558	gene-MA16_Dca018330
novel_miR_484	-2.721668558	gene-MA16_Dca001333
novel_miR_484	-2.721668558	gene-MA16_Dca014007
novel_miR_484	-2.721668558	gene-MA16_Dca028410
novel_miR_484	-2.721668558	gene-MA16_Dca028352
novel_miR_484	-2.721668558	gene-MA16_Dca010058
novel_miR_484	-2.721668558	gene-MA16_Dca000100
novel_miR_484	-2.721668558	gene-MA16_Dca021773
novel_miR_484	-2.721668558	gene-MA16_Dca001992
novel_miR_484	-2.721668558	gene-MA16_Dca008877
novel_miR_484	-2.721668558	gene-MA16_Dca028562
novel_miR_484	-2.721668558	gene-MA16_Dca021141
novel_miR_484	-2.721668558	gene-MA16_Dca024139
novel_miR_484	-2.721668558	gene-MA16_Dca005319
novel_miR_484	-2.721668558	gene-MA16_Dca011443
novel_miR_484	-2.721668558	gene-MA16_Dca022957
novel_miR_484	-2.721668558	gene-MA16_Dca014398
novel_miR_484	-2.721668558	gene-MA16_Dca003585
novel_miR_484	-2.721668558	gene-MA16_Dca008889
novel_miR_484	-2.721668558	gene-MA16_Dca026047
novel_miR_484	-2.721668558	gene-MA16_Dca019039
novel_miR_484	-2.721668558	gene-MA16_Dca012296
novel_miR_484	-2.721668558	gene-MA16_Dca011298
novel_miR_484	-2.721668558	gene-MA16_Dca023407
novel_miR_484	-2.721668558	gene-MA16_Dca017598
novel_miR_484	-2.721668558	gene-MA16_Dca023855
novel_miR_484	-2.721668558	gene-MA16_Dca004340
novel_miR_484	-2.721668558	gene-MA16_Dca007240
novel_miR_484	-2.721668558	gene-MA16_Dca005346
novel_miR_484	-2.721668558	gene-MA16_Dca009990
novel_miR_484	-2.721668558	gene-MA16_Dca014237
novel_miR_484	-2.721668558	gene-MA16_Dca024325

novel_miR_484	-2.721668558	gene-MA16_Dca022905
novel_miR_484	-2.721668558	gene-MA16_Dca019790
novel_miR_484	-2.721668558	gene-MA16_Dca010093
novel_miR_484	-2.721668558	gene-MA16_Dca015921
novel_miR_484	-2.721668558	gene-MA16_Dca017338
novel_miR_484	-2.721668558	gene-MA16_Dca027164
novel_miR_484	-2.721668558	gene-MA16_Dca022795
novel_miR_484	-2.721668558	gene-MA16_Dca012251
novel_miR_244	1.601838276	gene-MA16_Dca025956
novel_miR_71	1.602882269	gene-MA16_Dca025956
miR399e_5p_2	0.923894241	gene-MA16_Dca007637
novel_miR_45	1.461548561	gene-MA16_Dca005043
novel_miR_45	1.461548561	gene-MA16_Dca019234
novel_miR_45	1.461548561	gene-MA16_Dca024771
novel_miR_45	1.461548561	gene-MA16_Dca009889
novel_miR_45	1.461548561	gene-MA16_Dca007090
novel_miR_45	1.461548561	gene-MA16_Dca003489
novel_miR_45	1.461548561	gene-MA16_Dca008938
novel_miR_45	1.461548561	gene-MA16_Dca011854
novel_miR_45	1.461548561	gene-MA16_Dca012991
novel_miR_45	1.461548561	gene-MA16_Dca025486
novel_miR_45	1.461548561	gene-MA16_Dca001590
novel_miR_45	1.461548561	gene-MA16_Dca012774
novel_miR_45	1.461548561	gene-MA16_Dca010838
novel_miR_45	1.461548561	gene-MA16_Dca008335
novel_miR_384	0.809933153	gene-MA16_Dca022279
novel_miR_384	0.809933153	gene-MA16_Dca013574
novel_miR_192	0.790235609	gene-MA16_Dca010818
novel_miR_192	0.790235609	gene-MA16_Dca005939
novel_miR_453	0.761802771	gene-MA16_Dca002332
novel_miR_453	0.761802771	gene-MA16_Dca015927
novel_miR_453	0.761802771	gene-MA16_Dca020219
novel_miR_453	0.761802771	gene-MA16_Dca014772
novel_miR_453	0.761802771	gene-MA16_Dca008206

novel_miR_159	-0.831925691	gene-MA16_Dca013669
novel_miR_159	-0.831925691	gene-MA16_Dca015045
novel_miR_159	-0.831925691	gene-MA16_Dca016284
novel_miR_159	-0.831925691	gene-MA16_Dca018922
novel_miR_159	-0.831925691	gene-MA16_Dca007765
novel_miR_159	-0.831925691	gene-MA16_Dca007257
novel_miR_159	-0.831925691	gene-MA16_Dca026264
novel_miR_159	-0.831925691	gene-MA16_Dca020115
novel_miR_159	-0.831925691	gene-MA16_Dca001020
novel_miR_159	-0.831925691	gene-MA16_Dca001828
novel_miR_159	-0.831925691	gene-MA16_Dca016652
novel_miR_159	-0.831925691	gene-MA16_Dca027076
novel_miR_159	-0.831925691	gene-MA16_Dca013843
novel_miR_159	-0.831925691	gene-MA16_Dca008856
novel_miR_159	-0.831925691	gene-MA16_Dca006592
novel_miR_159	-0.831925691	gene-MA16_Dca021824
novel_miR_159	-0.831925691	gene-MA16_Dca006324
novel_miR_159	-0.831925691	gene-MA16_Dca014974
novel_miR_159	-0.831925691	gene-MA16_Dca022392
novel_miR_159	-0.831925691	gene-MA16_Dca010817
novel_miR_159	-0.831925691	gene-MA16_Dca007613
novel_miR_159	-0.831925691	gene-MA16_Dca017065
novel_miR_159	-0.831925691	gene-MA16_Dca007050
novel_miR_159	-0.831925691	gene-MA16_Dca005372
novel_miR_159	-0.831925691	gene-MA16_Dca022401
novel_miR_159	-0.831925691	gene-MA16_Dca007408
novel_miR_159	-0.831925691	gene-MA16_Dca016389
novel_miR_159	-0.831925691	gene-MA16_Dca016013
novel_miR_159	-0.831925691	gene-MA16_Dca012490
novel_miR_159	-0.831925691	gene-MA16_Dca021414
novel_miR_159	-0.831925691	gene-MA16_Dca011522
novel_miR_159	-0.831925691	gene-MA16_Dca015083
novel_miR_159	-0.831925691	gene-MA16_Dca006829
novel_miR_159	-0.831925691	gene-MA16_Dca014007

novel_miR_159	-0.831925691	gene-MA16_Dca006179
novel_miR_159	-0.831925691	gene-MA16_Dca019278
novel_miR_159	-0.831925691	gene-MA16_Dca001444
novel_miR_159	-0.831925691	gene-MA16_Dca014143
novel_miR_159	-0.831925691	gene-MA16_Dca019818
novel_miR_159	-0.831925691	gene-MA16_Dca015975
novel_miR_159	-0.831925691	gene-MA16_Dca006828
novel_miR_159	-0.831925691	gene-MA16_Dca023636
novel_miR_159	-0.831925691	gene-MA16_Dca027407
novel_miR_159	-0.831925691	gene-MA16_Dca002029
novel_miR_159	-0.831925691	gene-MA16_Dca001924
novel_miR_159	-0.831925691	gene-MA16_Dca022572
novel_miR_159	-0.831925691	gene-MA16_Dca010252
novel_miR_159	-0.831925691	gene-MA16_Dca023553
novel_miR_159	-0.831925691	gene-MA16_Dca005107
novel_miR_159	-0.831925691	gene-MA16_Dca024023
novel_miR_159	-0.831925691	gene-MA16_Dca024366
novel_miR_159	-0.831925691	gene-MA16_Dca002789
novel_miR_159	-0.831925691	gene-MA16_Dca019273
novel_miR_159	-0.831925691	gene-MA16_Dca011278
novel_miR_159	-0.831925691	gene-MA16_Dca018789
novel_miR_159	-0.831925691	gene-MA16_Dca018071
novel_miR_159	-0.831925691	gene-MA16_Dca021047
novel_miR_159	-0.831925691	gene-MA16_Dca003342
novel_miR_159	-0.831925691	gene-MA16_Dca005616
novel_miR_159	-0.831925691	gene-MA16_Dca025367
novel_miR_159	-0.831925691	gene-MA16_Dca028626
novel_miR_159	-0.831925691	gene-MA16_Dca012922
miR530a	0.940135919	gene-MA16_Dca022268
miR530a	0.940135919	gene-MA16_Dca019882
miR530a	0.940135919	gene-MA16_Dca013836
miR530a	0.940135919	gene-MA16_Dca011594
miR530a	0.940135919	gene-MA16_Dca010385
novel_miR_242	-1.030598853	gene-MA16_Dca015938

novel_miR_242	-1.030598853	gene-MA16_Dca002874
novel_miR_242	-1.030598853	gene-MA16_Dca020985
novel_miR_242	-1.030598853	gene-MA16_Dca006366
novel_miR_242	-1.030598853	gene-MA16_Dca013024
novel_miR_242	-1.030598853	gene-MA16_Dca023978
novel_miR_242	-1.030598853	gene-MA16_Dca003396
novel_miR_407	0.679898141	gene-MA16_Dca007135
miR812o_5p_2	-1.01136364	gene-MA16_Dca015731
miR812o_5p_1	-1.011363902	gene-MA16_Dca015731
novel_miR_53	-1.956372727	gene-MA16_Dca005089
novel_miR_53	-1.956372727	gene-MA16_Dca020713
novel_miR_53	-1.956372727	gene-MA16_Dca022778
novel_miR_53	-1.956372727	gene-MA16_Dca001003
novel_miR_53	-1.956372727	gene-MA16_Dca007171
novel_miR_53	-1.956372727	gene-MA16_Dca014115
novel_miR_53	-1.956372727	gene-MA16_Dca000122
novel_miR_53	-1.956372727	gene-MA16_Dca012806
novel_miR_53	-1.956372727	gene-MA16_Dca003261
novel_miR_53	-1.956372727	gene-MA16_Dca000505
novel_miR_53	-1.956372727	gene-MA16_Dca009084
novel_miR_53	-1.956372727	gene-MA16_Dca012781
novel_miR_53	-1.956372727	gene-MA16_Dca010665
novel_miR_53	-1.956372727	gene-MA16_Dca018178
novel_miR_53	-1.956372727	gene-MA16_Dca011106
novel_miR_53	-1.956372727	gene-MA16_Dca023559
novel_miR_53	-1.956372727	gene-MA16_Dca017541
novel_miR_53	-1.956372727	gene-MA16_Dca002207
novel_miR_53	-1.956372727	gene-MA16_Dca017663
novel_miR_53	-1.956372727	gene-MA16_Dca017043
novel_miR_53	-1.956372727	gene-MA16_Dca010639
novel_miR_53	-1.956372727	gene-MA16_Dca000121
novel_miR_53	-1.956372727	gene-MA16_Dca023432
novel_miR_53	-1.956372727	gene-MA16_Dca015909
novel_miR_53	-1.956372727	gene-MA16_Dca002218

novel_miR_53	-1.956372727	gene-MA16_Dca011246
novel_miR_53	-1.956372727	gene-MA16_Dca003197
novel_miR_53	-1.956372727	gene-MA16_Dca012420
novel_miR_53	-1.956372727	gene-MA16_Dca001258
novel_miR_53	-1.956372727	gene-MA16_Dca006414
novel_miR_53	-1.956372727	gene-MA16_Dca021885
novel_miR_53	-1.956372727	gene-MA16_Dca023972
novel_miR_53	-1.956372727	gene-MA16_Dca014156
novel_miR_53	-1.956372727	gene-MA16_Dca007605
novel_miR_53	-1.956372727	gene-MA16_Dca022196
novel_miR_53	-1.956372727	gene-MA16_Dca015291
novel_miR_304	0.901669585	gene-MA16_Dca023243
novel_miR_304	0.901669585	gene-MA16_Dca026084
novel_miR_304	0.901669585	gene-MA16_Dca019935
novel_miR_304	0.901669585	gene-MA16_Dca014822
novel_miR_304	0.901669585	gene-MA16_Dca015763
novel_miR_304	0.901669585	gene-MA16_Dca005051
novel_miR_304	0.901669585	gene-MA16_Dca014450
novel_miR_304	0.901669585	gene-MA16_Dca027654
novel_miR_304	0.901669585	gene-MA16_Dca007509
novel_miR_304	0.901669585	gene-MA16_Dca005183
novel_miR_304	0.901669585	gene-MA16_Dca014980
novel_miR_304	0.901669585	gene-MA16_Dca014981
novel_miR_304	0.901669585	gene-MA16_Dca022317
novel_miR_304	0.901669585	gene-MA16_Dca014820
novel_miR_304	0.901669585	gene-MA16_Dca016634
novel_miR_275	-0.691215158	gene-MA16_Dca002588
novel_miR_275	-0.691215158	gene-MA16_Dca018663
novel_miR_275	-0.691215158	gene-MA16_Dca015366
novel_miR_275	-0.691215158	gene-MA16_Dca013677
novel_miR_275	-0.691215158	gene-MA16_Dca002895
novel_miR_275	-0.691215158	gene-MA16_Dca010185
novel_miR_275	-0.691215158	gene-MA16_Dca014504
novel_miR_275	-0.691215158	gene-MA16_Dca008901

novel_miR_275	-0.691215158	gene-MA16_Dca009632
novel_miR_275	-0.691215158	gene-MA16_Dca020488
miR393b_3p	0.715544357	gene-MA16_Dca024562
novel_miR_83	0.614603692	gene-MA16_Dca010287
novel_miR_83	0.614603692	gene-MA16_Dca008310
novel_miR_83	0.614603692	gene-MA16_Dca006555
novel_miR_83	0.614603692	gene-MA16_Dca023565
novel_miR_83	0.614603692	gene-MA16_Dca008336
novel_miR_83	0.614603692	gene-MA16_Dca026847
novel_miR_83	0.614603692	gene-MA16_Dca016013
novel_miR_83	0.614603692	gene-MA16_Dca010769
novel_miR_83	0.614603692	gene-MA16_Dca024729
novel_miR_83	0.614603692	gene-MA16_Dca001201
novel_miR_83	0.614603692	gene-MA16_Dca000143
novel_miR_83	0.614603692	gene-MA16_Dca000721
novel_miR_83	0.614603692	gene-MA16_Dca006432
novel_miR_141	0.701457284	gene-MA16_Dca026118
novel_miR_141	0.701457284	gene-MA16_Dca007698
miR396b_1	0.71194909	gene-MA16_Dca005596
miR396b_1	0.71194909	gene-MA16_Dca016354
miR396b_1	0.71194909	gene-MA16_Dca013418
miR396b_1	0.71194909	gene-MA16_Dca007307
miR396b_1	0.71194909	gene-MA16_Dca017977
miR396b_1	0.71194909	gene-MA16_Dca014266
miR396b_1	0.71194909	gene-MA16_Dca016598
miR396b_1	0.71194909	gene-MA16_Dca001930
miR396b_1	0.71194909	gene-MA16_Dca014260
miR396b_1	0.71194909	gene-MA16_Dca022287
miR396b_1	0.71194909	gene-MA16_Dca024909
miR396b_1	0.71194909	gene-MA16_Dca004133
miR396b_1	0.71194909	gene-MA16_Dca025782
miR396b_1	0.71194909	gene-MA16_Dca004158
miR396b_1	0.71194909	gene-MA16_Dca023002
miR396b_1	0.71194909	gene-MA16_Dca008028
miR396b_1	0.71194909	gene-MA16_Dca014631
miR396b_1	0.71194909	gene-MA16_Dca004139
miR396b_1	0.71194909	gene-MA16_Dca018722
miR396b_1	0.71194909	gene-MA16_Dca021860

miR396b_1	0.71194909	gene-MA16_Dca012576
miR396b_1	0.71194909	gene-MA16_Dca003694
miR396b_1	0.71194909	gene-MA16_Dca021863
miR396b_1	0.71194909	gene-MA16_Dca005799
miR396b_1	0.71194909	gene-MA16_Dca022508
miR396b_1	0.71194909	gene-MA16_Dca018725
miR396b_1	0.71194909	gene-MA16_Dca023691
miR396b_1	0.71194909	gene-MA16_Dca023840
miR396b_1	0.71194909	gene-MA16_Dca010315
miR396b_1	0.71194909	gene-MA16_Dca006059
miR396b_1	0.71194909	gene-MA16_Dca022211
miR396b_1	0.71194909	gene-MA16_Dca000152
miR396b_1	0.71194909	gene-MA16_Dca021850
miR396b_1	0.71194909	gene-MA16_Dca006898
miR396b_1	0.71194909	gene-MA16_Dca019153
miR396b_1	0.71194909	gene-MA16_Dca019207
miR396b_1	0.71194909	gene-MA16_Dca010222
miR396b_1	0.71194909	gene-MA16_Dca023379
miR396b_1	0.71194909	gene-MA16_Dca019943
miR396b_1	0.71194909	gene-MA16_Dca000754
miR396b_1	0.71194909	gene-MA16_Dca013497
miR396b_1	0.71194909	gene-MA16_Dca018548
miR396b_1	0.71194909	gene-MA16_Dca018172
miR396b_1	0.71194909	gene-MA16_Dca001920
miR396b_1	0.71194909	gene-MA16_Dca012093
miR396b_1	0.71194909	gene-MA16_Dca024581
miR396b_1	0.71194909	gene-MA16_Dca001936
miR396b_1	0.71194909	gene-MA16_Dca024442
miR396b_1	0.71194909	gene-MA16_Dca001785
miR396b_1	0.71194909	gene-MA16_Dca006179
miR396b_1	0.71194909	gene-MA16_Dca001199
miR396b_1	0.71194909	gene-MA16_Dca004093
miR396b_1	0.71194909	gene-MA16_Dca002377
miR396b_1	0.71194909	gene-MA16_Dca026927
miR396b_1	0.71194909	gene-MA16_Dca006631
miR396b_1	0.71194909	gene-MA16_Dca012128
miR396b_1	0.71194909	gene-MA16_Dca002242
miR396b_1	0.71194909	gene-MA16_Dca011294
miR396b_1	0.71194909	gene-MA16_Dca001955
miR396b_1	0.71194909	gene-MA16_Dca010163
miR396b_1	0.71194909	gene-MA16_Dca010619
miR396b_1	0.71194909	gene-MA16_Dca020583
miR396b_1	0.71194909	gene-MA16_Dca003911
miR396b_1	0.71194909	gene-MA16_Dca028471

miR396b_1	0.71194909	gene-MA16_Dca025262
miR396b_1	0.71194909	gene-MA16_Dca011382
miR396b_1	0.71194909	gene-MA16_Dca012099
miR396b_1	0.71194909	gene-MA16_Dca020910
miR396b_1	0.71194909	gene-MA16_Dca010708
miR396b_1	0.71194909	gene-MA16_Dca004692
miR396b_1	0.71194909	gene-MA16_Dca003177
miR396b_1	0.71194909	gene-MA16_Dca024988
miR396b_1	0.71194909	gene-MA16_Dca027492
miR396b_1	0.71194909	gene-MA16_Dca011959
miR396b_1	0.71194909	gene-MA16_Dca011627
miR396b_1	0.71194909	gene-MA16_Dca021446
miR396b_1	0.71194909	gene-MA16_Dca020207
miR396b_1	0.71194909	gene-MA16_Dca007949
miR396b_1	0.71194909	gene-MA16_Dca016956
miR396b_1	0.71194909	gene-MA16_Dca011603
miR396b_1	0.71194909	gene-MA16_Dca013322
miR396b_1	0.71194909	gene-MA16_Dca025223
miR396b_1	0.71194909	gene-MA16_Dca009226
miR396b_1	0.71194909	gene-MA16_Dca024281
miR396b_1	0.71194909	gene-MA16_Dca015409
miR396b_1	0.71194909	gene-MA16_Dca023601
miR396b_1	0.71194909	gene-MA16_Dca004403
miR396b_1	0.71194909	gene-MA16_Dca019222
miR396b_1	0.71194909	gene-MA16_Dca024737
miR396b_1	0.71194909	gene-MA16_Dca014751
miR396b_1	0.71194909	gene-MA16_Dca022302
miR396b_1	0.71194909	gene-MA16_Dca005617
miR396b_1	0.71194909	gene-MA16_Dca000619
miR396b_1	0.71194909	gene-MA16_Dca002697
miR396b_1	0.71194909	gene-MA16_Dca007955
miR396b_1	0.71194909	gene-MA16_Dca020801
miR396b_1	0.71194909	gene-MA16_Dca012938
miR396b_1	0.71194909	gene-MA16_Dca002241
miR396b_1	0.71194909	gene-MA16_Dca019707
miR396b_1	0.71194909	gene-MA16_Dca020166
miR396b_1	0.71194909	gene-MA16_Dca018345
miR396b_1	0.71194909	gene-MA16_Dca008222
miR396b_1	0.71194909	gene-MA16_Dca006666
miR396b_1	0.71194909	gene-MA16_Dca007694
miR396b_1	0.71194909	gene-MA16_Dca000885
miR396b_1	0.71194909	gene-MA16_Dca011783
miR396b_1	0.71194909	gene-MA16_Dca005151
miR396b_1	0.71194909	gene-MA16_Dca011967

miR396b_1	0.71194909	gene-MA16_Dca011523
miR396b_1	0.71194909	gene-MA16_Dca011751
miR396b_1	0.71194909	gene-MA16_Dca015263
miR396b_1	0.71194909	gene-MA16_Dca005154
novel_miR_36	1.415174644	gene-MA16_Dca019065
novel_miR_36	1.415174644	gene-MA16_Dca020471
novel_miR_36	1.415174644	gene-MA16_Dca000545
novel_miR_36	1.415174644	gene-MA16_Dca021431
novel_miR_36	1.415174644	gene-MA16_Dca012725
novel_miR_36	1.415174644	gene-MA16_Dca014216
novel_miR_36	1.415174644	gene-MA16_Dca011134
novel_miR_36	1.415174644	gene-MA16_Dca000756
novel_miR_36	1.415174644	gene-MA16_Dca000998
novel_miR_36	1.415174644	gene-MA16_Dca018248
novel_miR_36	1.415174644	gene-MA16_Dca000532
novel_miR_36	1.415174644	gene-MA16_Dca027962
novel_miR_36	1.415174644	gene-MA16_Dca010554
novel_miR_36	1.415174644	gene-MA16_Dca001894
novel_miR_36	1.415174644	gene-MA16_Dca013923
novel_miR_36	1.415174644	gene-MA16_Dca005787
novel_miR_36	1.415174644	gene-MA16_Dca007864
novel_miR_36	1.415174644	gene-MA16_Dca016860
novel_miR_36	1.415174644	gene-MA16_Dca024909
novel_miR_36	1.415174644	gene-MA16_Dca009506
novel_miR_36	1.415174644	gene-MA16_Dca002451
novel_miR_36	1.415174644	gene-MA16_Dca000981
novel_miR_36	1.415174644	gene-MA16_Dca002514
novel_miR_36	1.415174644	gene-MA16_Dca028508
novel_miR_36	1.415174644	gene-MA16_Dca003966
novel_miR_36	1.415174644	gene-MA16_Dca020080
novel_miR_36	1.415174644	gene-MA16_Dca018457
novel_miR_36	1.415174644	gene-MA16_Dca019557
novel_miR_36	1.415174644	gene-MA16_Dca023835
novel_miR_36	1.415174644	gene-MA16_Dca010684
novel_miR_36	1.415174644	gene-MA16_Dca024050
novel_miR_36	1.415174644	gene-MA16_Dca014331
novel_miR_36	1.415174644	gene-MA16_Dca012607
novel_miR_36	1.415174644	gene-MA16_Dca000014
novel_miR_36	1.415174644	gene-MA16_Dca021283
novel_miR_36	1.415174644	gene-MA16_Dca003117
novel_miR_36	1.415174644	gene-MA16_Dca003709
novel_miR_36	1.415174644	gene-MA16_Dca000891
novel_miR_36	1.415174644	gene-MA16_Dca006786

novel_miR_36	1.415174644	gene-MA16_Dca004003
novel_miR_36	1.415174644	gene-MA16_Dca021158
novel_miR_36	1.415174644	gene-MA16_Dca010535
novel_miR_36	1.415174644	gene-MA16_Dca013981
novel_miR_36	1.415174644	gene-MA16_Dca006650
novel_miR_36	1.415174644	gene-MA16_Dca019758
novel_miR_36	1.415174644	gene-MA16_Dca024982
novel_miR_36	1.415174644	gene-MA16_Dca009980
novel_miR_36	1.415174644	gene-MA16_Dca001915
novel_miR_36	1.415174644	gene-MA16_Dca027576
novel_miR_36	1.415174644	gene-MA16_Dca014756
novel_miR_36	1.415174644	gene-MA16_Dca004014
novel_miR_36	1.415174644	gene-MA16_Dca004713
novel_miR_36	1.415174644	gene-MA16_Dca014400
novel_miR_36	1.415174644	gene-MA16_Dca025030
novel_miR_36	1.415174644	gene-MA16_Dca006821
novel_miR_36	1.415174644	gene-MA16_Dca007063
novel_miR_36	1.415174644	gene-MA16_Dca021282
novel_miR_36	1.415174644	gene-MA16_Dca010505
novel_miR_36	1.415174644	gene-MA16_Dca006834
novel_miR_36	1.415174644	gene-MA16_Dca006592
novel_miR_36	1.415174644	gene-MA16_Dca016085
novel_miR_36	1.415174644	gene-MA16_Dca020908
novel_miR_36	1.415174644	gene-MA16_Dca008320
novel_miR_36	1.415174644	gene-MA16_Dca025409
novel_miR_36	1.415174644	gene-MA16_Dca027301
novel_miR_36	1.415174644	gene-MA16_Dca025553
novel_miR_36	1.415174644	gene-MA16_Dca024504
novel_miR_36	1.415174644	gene-MA16_Dca000308
novel_miR_36	1.415174644	gene-MA16_Dca016262
novel_miR_36	1.415174644	gene-MA16_Dca018766
novel_miR_36	1.415174644	gene-MA16_Dca009185
novel_miR_36	1.415174644	gene-MA16_Dca007565
novel_miR_36	1.415174644	gene-MA16_Dca006362
novel_miR_36	1.415174644	gene-MA16_Dca016370
novel_miR_36	1.415174644	gene-MA16_Dca021043
novel_miR_36	1.415174644	gene-MA16_Dca026698
novel_miR_36	1.415174644	gene-MA16_Dca020464
novel_miR_36	1.415174644	gene-MA16_Dca002971
novel_miR_36	1.415174644	gene-MA16_Dca004287
novel_miR_36	1.415174644	gene-MA16_Dca019933
novel_miR_36	1.415174644	gene-MA16_Dca023526
novel_miR_36	1.415174644	gene-MA16_Dca026606
novel_miR_36	1.415174644	gene-MA16_Dca021281

novel_miR_36	1.415174644	gene-MA16_Dca004093
novel_miR_36	1.415174644	gene-MA16_Dca021264
novel_miR_36	1.415174644	gene-MA16_Dca019418
novel_miR_36	1.415174644	gene-MA16_Dca024861
novel_miR_36	1.415174644	gene-MA16_Dca023178
novel_miR_36	1.415174644	gene-MA16_Dca018127
novel_miR_36	1.415174644	gene-MA16_Dca016809
novel_miR_36	1.415174644	gene-MA16_Dca023572
novel_miR_36	1.415174644	gene-MA16_Dca003059
novel_miR_36	1.415174644	gene-MA16_Dca021850
novel_miR_36	1.415174644	gene-MA16_Dca005952
novel_miR_36	1.415174644	gene-MA16_Dca007629
novel_miR_36	1.415174644	gene-MA16_Dca000832
novel_miR_36	1.415174644	gene-MA16_Dca014211
novel_miR_36	1.415174644	gene-MA16_Dca011125
novel_miR_36	1.415174644	gene-MA16_Dca010287
novel_miR_36	1.415174644	gene-MA16_Dca022643
novel_miR_36	1.415174644	gene-MA16_Dca005341
novel_miR_36	1.415174644	gene-MA16_Dca015149
novel_miR_36	1.415174644	gene-MA16_Dca009014
novel_miR_36	1.415174644	gene-MA16_Dca007413
novel_miR_36	1.415174644	gene-MA16_Dca009995
novel_miR_36	1.415174644	gene-MA16_Dca003869
novel_miR_36	1.415174644	gene-MA16_Dca006386
novel_miR_36	1.415174644	gene-MA16_Dca002837
novel_miR_36	1.415174644	gene-MA16_Dca000558
novel_miR_36	1.415174644	gene-MA16_Dca003718
novel_miR_36	1.415174644	gene-MA16_Dca023524
novel_miR_36	1.415174644	gene-MA16_Dca023411
novel_miR_36	1.415174644	gene-MA16_Dca006773
novel_miR_36	1.415174644	gene-MA16_Dca009122
novel_miR_36	1.415174644	gene-MA16_Dca019385
novel_miR_36	1.415174644	gene-MA16_Dca021058
novel_miR_36	1.415174644	gene-MA16_Dca008399
novel_miR_36	1.415174644	gene-MA16_Dca002388
novel_miR_36	1.415174644	gene-MA16_Dca000925
novel_miR_36	1.415174644	gene-MA16_Dca002118
novel_miR_36	1.415174644	gene-MA16_Dca022280
novel_miR_36	1.415174644	gene-MA16_Dca016304
novel_miR_36	1.415174644	gene-MA16_Dca017029
novel_miR_36	1.415174644	gene-MA16_Dca007845
novel_miR_36	1.415174644	gene-MA16_Dca002586
novel_miR_36	1.415174644	gene-MA16_Dca000247
novel_miR_36	1.415174644	gene-MA16_Dca016371

novel_miR_36	1.415174644	gene-MA16_Dca010730
novel_miR_36	1.415174644	gene-MA16_Dca025840
novel_miR_36	1.415174644	gene-MA16_Dca004007
novel_miR_36	1.415174644	gene-MA16_Dca015376
novel_miR_36	1.415174644	gene-MA16_Dca002952
novel_miR_36	1.415174644	gene-MA16_Dca012778
novel_miR_36	1.415174644	gene-MA16_Dca010799
novel_miR_36	1.415174644	gene-MA16_Dca001427
novel_miR_36	1.415174644	gene-MA16_Dca023966
novel_miR_36	1.415174644	gene-MA16_Dca023056
novel_miR_36	1.415174644	gene-MA16_Dca003169
novel_miR_36	1.415174644	gene-MA16_Dca011335
novel_miR_36	1.415174644	gene-MA16_Dca004266
novel_miR_36	1.415174644	gene-MA16_Dca006091
novel_miR_36	1.415174644	gene-MA16_Dca013596
novel_miR_36	1.415174644	gene-MA16_Dca027711
novel_miR_36	1.415174644	gene-MA16_Dca017554
novel_miR_36	1.415174644	gene-MA16_Dca026346
novel_miR_36	1.415174644	gene-MA16_Dca016864
novel_miR_36	1.415174644	gene-MA16_Dca016800
novel_miR_36	1.415174644	gene-MA16_Dca024809
novel_miR_36	1.415174644	gene-MA16_Dca021203
novel_miR_36	1.415174644	gene-MA16_Dca006479
novel_miR_36	1.415174644	gene-MA16_Dca004957
novel_miR_36	1.415174644	gene-MA16_Dca000326
novel_miR_36	1.415174644	gene-MA16_Dca020366
novel_miR_36	1.415174644	gene-MA16_Dca003650
novel_miR_223	0.785835068	gene-MA16_Dca022487
novel_miR_236	0.700468644	gene-MA16_Dca026583
novel_miR_236	0.700468644	gene-MA16_Dca017549
novel_miR_236	0.700468644	gene-MA16_Dca002308
novel_miR_236	0.700468644	gene-MA16_Dca004606
novel_miR_236	0.700468644	gene-MA16_Dca007852
novel_miR_236	0.700468644	gene-MA16_Dca011099
novel_miR_236	0.700468644	gene-MA16_Dca007249
novel_miR_236	0.700468644	gene-MA16_Dca003804
novel_miR_236	0.700468644	gene-MA16_Dca014769
miR157d_3p	-1.256108966	gene-MA16_Dca001937
miR157d_3p	-1.256108966	gene-MA16_Dca021175
miR157d_3p	-1.256108966	gene-MA16_Dca010207
miR157d_3p	-1.256108966	gene-MA16_Dca008286
miR157d_3p	-1.256108966	gene-MA16_Dca006136
miR157d_3p	-1.256108966	gene-MA16_Dca026671

miR157d_3p	-1.256108966	gene-MA16_Dca015720
miR157d_3p	-1.256108966	gene-MA16_Dca004199
miR157d_3p	-1.256108966	gene-MA16_Dca011973
miR157d_3p	-1.256108966	gene-MA16_Dca012946
miR157d_3p	-1.256108966	gene-MA16_Dca022543
miR157d_3p	-1.256108966	gene-MA16_Dca002954
miR157d_3p	-1.256108966	gene-MA16_Dca009889
miR157d_3p	-1.256108966	gene-MA16_Dca025192
miR157d_3p	-1.256108966	gene-MA16_Dca015957
miR528_5p	-1.034599625	gene-MA16_Dca020077
miR528_5p	-1.034599625	gene-MA16_Dca010331
miR528_5p	-1.034599625	gene-MA16_Dca002770
miR528_5p	-1.034599625	gene-MA16_Dca004667
miR528_5p	-1.034599625	gene-MA16_Dca000891
miR528_5p	-1.034599625	gene-MA16_Dca027730
miR528_5p	-1.034599625	gene-MA16_Dca012338
miR528_5p	-1.034599625	gene-MA16_Dca015008
miR528_5p	-1.034599625	gene-MA16_Dca021334
miR528_5p	-1.034599625	gene-MA16_Dca013643
miR528_5p	-1.034599625	gene-MA16_Dca022587
miR528_5p	-1.034599625	gene-MA16_Dca014181
miR528_5p	-1.034599625	gene-MA16_Dca015948
miR528_5p	-1.034599625	gene-MA16_Dca013418
miR528_5p	-1.034599625	gene-MA16_Dca028734
miR528_5p	-1.034599625	gene-MA16_Dca020299
miR528_5p	-1.034599625	gene-MA16_Dca009198
miR528_5p	-1.034599625	gene-MA16_Dca021947
miR528_5p	-1.034599625	gene-MA16_Dca026005
miR528_5p	-1.034599625	gene-MA16_Dca011035
miR528_5p	-1.034599625	gene-MA16_Dca010931
miR528_5p	-1.034599625	gene-MA16_Dca019975
miR528_5p	-1.034599625	gene-MA16_Dca001951
miR528_5p	-1.034599625	gene-MA16_Dca000698
miR528_5p	-1.034599625	gene-MA16_Dca011273
miR528_5p	-1.034599625	gene-MA16_Dca002672
miR528_5p	-1.034599625	gene-MA16_Dca026199
miR528_5p	-1.034599625	gene-MA16_Dca020850
miR528_5p	-1.034599625	gene-MA16_Dca003559
miR528_5p	-1.034599625	gene-MA16_Dca026841
miR528_5p	-1.034599625	gene-MA16_Dca026559
novel_miR_431	0.722194154	gene-MA16_Dca019557
miR8565d	0.589107991	gene-MA16_Dca009952
miR8032f_3p	-0.735553454	gene-MA16_Dca000347

miR7994a	0.631452312	gene-MA16_Dca009302
miR7994a	0.631452312	gene-MA16_Dca001651
miR7994a	0.631452312	gene-MA16_Dca024189
miR7994a	0.631452312	gene-MA16_Dca015708
miR7994a	0.631452312	gene-MA16_Dca027522
miR951	-0.697528726	gene-MA16_Dca004416
miR164e_5p_3	0.756720901	gene-MA16_Dca002744
miR164e_5p_3	0.756720901	gene-MA16_Dca021600
miR164e_5p_3	0.756720901	gene-MA16_Dca023179
miR164e_5p_3	0.756720901	gene-MA16_Dca016023
miR164e_5p_3	0.756720901	gene-MA16_Dca008993
miR164e_5p_3	0.756720901	gene-MA16_Dca005860
miR164e_5p_3	0.756720901	gene-MA16_Dca006933
miR164e_5p_3	0.756720901	gene-MA16_Dca000173
miR164e_5p_3	0.756720901	gene-MA16_Dca004236
miR164e_5p_3	0.756720901	gene-MA16_Dca015801
miR164e_5p_3	0.756720901	gene-MA16_Dca015159
miR164e_5p_3	0.756720901	gene-MA16_Dca022156
miR164e_5p_3	0.756720901	gene-MA16_Dca020570
miR164e_5p_3	0.756720901	gene-MA16_Dca004955
miR164e_5p_3	0.756720901	gene-MA16_Dca027794
miR164e_5p_3	0.756720901	gene-MA16_Dca020338
miR164e_5p_3	0.756720901	gene-MA16_Dca026959
miR164e_5p_3	0.756720901	gene-MA16_Dca025776
miR164e_5p_3	0.756720901	gene-MA16_Dca009952
miR164e_5p_3	0.756720901	gene-MA16_Dca021318
miR164e_5p_3	0.756720901	gene-MA16_Dca013976
miR164e_5p_3	0.756720901	gene-MA16_Dca020264
miR164e_5p_3	0.756720901	gene-MA16_Dca012661
miR164e_5p_2	0.757229161	gene-MA16_Dca015801
miR164e_5p_2	0.757229161	gene-MA16_Dca008993
miR164e_5p_2	0.757229161	gene-MA16_Dca016023
miR164e_5p_2	0.757229161	gene-MA16_Dca005860
miR164e_5p_2	0.757229161	gene-MA16_Dca006933
miR164e_5p_2	0.757229161	gene-MA16_Dca004236
miR164e_5p_2	0.757229161	gene-MA16_Dca000173
miR164e_5p_2	0.757229161	gene-MA16_Dca023179
miR164e_5p_2	0.757229161	gene-MA16_Dca002744
miR164e_5p_2	0.757229161	gene-MA16_Dca021600
miR164e_5p_2	0.757229161	gene-MA16_Dca013976
miR164e_5p_2	0.757229161	gene-MA16_Dca020264
miR164e_5p_2	0.757229161	gene-MA16_Dca012661

miR164e_5p_2	0.757229161	gene-MA16_Dca020338
miR164e_5p_2	0.757229161	gene-MA16_Dca026959
miR164e_5p_2	0.757229161	gene-MA16_Dca025776
miR164e_5p_2	0.757229161	gene-MA16_Dca021318
miR164e_5p_2	0.757229161	gene-MA16_Dca009952
miR164e_5p_2	0.757229161	gene-MA16_Dca020570
miR164e_5p_2	0.757229161	gene-MA16_Dca004955
miR164e_5p_2	0.757229161	gene-MA16_Dca027794
miR164e_5p_2	0.757229161	gene-MA16_Dca015159
miR164e_5p_2	0.757229161	gene-MA16_Dca022156
miR164e_5p_1	0.73741028	gene-MA16_Dca015159
miR164e_5p_1	0.73741028	gene-MA16_Dca022156
miR164e_5p_1	0.73741028	gene-MA16_Dca020570
miR164e_5p_1	0.73741028	gene-MA16_Dca004955
miR164e_5p_1	0.73741028	gene-MA16_Dca027794
miR164e_5p_1	0.73741028	gene-MA16_Dca026959
miR164e_5p_1	0.73741028	gene-MA16_Dca020338
miR164e_5p_1	0.73741028	gene-MA16_Dca025776
miR164e_5p_1	0.73741028	gene-MA16_Dca009952
miR164e_5p_1	0.73741028	gene-MA16_Dca021318
miR164e_5p_1	0.73741028	gene-MA16_Dca013976
miR164e_5p_1	0.73741028	gene-MA16_Dca012661
miR164e_5p_1	0.73741028	gene-MA16_Dca020264
miR164e_5p_1	0.73741028	gene-MA16_Dca002744
miR164e_5p_1	0.73741028	gene-MA16_Dca021600
miR164e_5p_1	0.73741028	gene-MA16_Dca023179
miR164e_5p_1	0.73741028	gene-MA16_Dca008993
miR164e_5p_1	0.73741028	gene-MA16_Dca016023
miR164e_5p_1	0.73741028	gene-MA16_Dca005860
miR164e_5p_1	0.73741028	gene-MA16_Dca006933
miR164e_5p_1	0.73741028	gene-MA16_Dca004236
miR164e_5p_1	0.73741028	gene-MA16_Dca000173
miR164e_5p_1	0.73741028	gene-MA16_Dca015801
miR164e_5p_4	0.73749685	gene-MA16_Dca023179
miR164e_5p_4	0.73749685	gene-MA16_Dca021600
miR164e_5p_4	0.73749685	gene-MA16_Dca002744
miR164e_5p_4	0.73749685	gene-MA16_Dca015801
miR164e_5p_4	0.73749685	gene-MA16_Dca006933
miR164e_5p_4	0.73749685	gene-MA16_Dca004236
miR164e_5p_4	0.73749685	gene-MA16_Dca000173
miR164e_5p_4	0.73749685	gene-MA16_Dca008993
miR164e_5p_4	0.73749685	gene-MA16_Dca016023
miR164e_5p_4	0.73749685	gene-MA16_Dca005860

miR164e_5p_4	0.73749685	gene-MA16_Dca027794
miR164e_5p_4	0.73749685	gene-MA16_Dca020570
miR164e_5p_4	0.73749685	gene-MA16_Dca004955
miR164e_5p_4	0.73749685	gene-MA16_Dca015159
miR164e_5p_4	0.73749685	gene-MA16_Dca022156
miR164e_5p_4	0.73749685	gene-MA16_Dca013976
miR164e_5p_4	0.73749685	gene-MA16_Dca012661
miR164e_5p_4	0.73749685	gene-MA16_Dca020264
miR164e_5p_4	0.73749685	gene-MA16_Dca021318
miR164e_5p_4	0.73749685	gene-MA16_Dca009952
miR164e_5p_4	0.73749685	gene-MA16_Dca020338
miR164e_5p_4	0.73749685	gene-MA16_Dca026959
miR164e_5p_4	0.73749685	gene-MA16_Dca025776
miR394d	0.883926599	gene-MA16_Dca010123
miR394d	0.883926599	gene-MA16_Dca020985
miR394d	0.883926599	gene-MA16_Dca004056
miR394d	0.883926599	gene-MA16_Dca009669
miR394d	0.883926599	gene-MA16_Dca007071
miR394d	0.883926599	gene-MA16_Dca012102
miR394d	0.883926599	gene-MA16_Dca014211
miR394d	0.883926599	gene-MA16_Dca011616
miR394d	0.883926599	gene-MA16_Dca002476
miR394d	0.883926599	gene-MA16_Dca006481
miR394d	0.883926599	gene-MA16_Dca015255
miR394d	0.883926599	gene-MA16_Dca014991
miR394d	0.883926599	gene-MA16_Dca011228
miR394d	0.883926599	gene-MA16_Dca008070
miR394d	0.883926599	gene-MA16_Dca024716
miR394d	0.883926599	gene-MA16_Dca003173
miR394d	0.883926599	gene-MA16_Dca021852
miR394d	0.883926599	gene-MA16_Dca024947
miR394d	0.883926599	gene-MA16_Dca003369
miR394d	0.883926599	gene-MA16_Dca009679
miR394d	0.883926599	gene-MA16_Dca011533
miR172d_2	0.593718429	gene-MA16_Dca010160
miR172d_2	0.593718429	gene-MA16_Dca017222
miR172d_2	0.593718429	gene-MA16_Dca002039
miR172d_2	0.593718429	gene-MA16_Dca018756
miR172d_2	0.593718429	gene-MA16_Dca008031
miR172d_2	0.593718429	gene-MA16_Dca020018
miR172d_2	0.593718429	gene-MA16_Dca000260
miR172d_2	0.593718429	gene-MA16_Dca002934
miR172d_2	0.593718429	gene-MA16_Dca022453

miR172d_2	0.593718429	gene-MA16_Dca009661
miR172d_2	0.593718429	gene-MA16_Dca013779
miR172d_2	0.593718429	gene-MA16_Dca022454
miR172d_2	0.593718429	gene-MA16_Dca021336
miR172d_2	0.593718429	gene-MA16_Dca022520
miR172d_2	0.593718429	gene-MA16_Dca016851
miR172d_2	0.593718429	gene-MA16_Dca013460
miR172d_2	0.593718429	gene-MA16_Dca022452
miR172d_2	0.593718429	gene-MA16_Dca006530
miR172d_2	0.593718429	gene-MA16_Dca017910
miR172d_2	0.593718429	gene-MA16_Dca022455
miR172d_2	0.593718429	gene-MA16_Dca007639
miR172d_2	0.593718429	gene-MA16_Dca012666
miR172d_2	0.593718429	gene-MA16_Dca004101
miR172d_2	0.593718429	gene-MA16_Dca001782
miR172d_2	0.593718429	gene-MA16_Dca018995
miR172d_2	0.593718429	gene-MA16_Dca013902
miR172d_2	0.593718429	gene-MA16_Dca020870
miR172d_2	0.593718429	gene-MA16_Dca020793
miR172d_2	0.593718429	gene-MA16_Dca011850
miR172d_2	0.593718429	gene-MA16_Dca016049
miR172d_2	0.593718429	gene-MA16_Dca015839
miR172d_2	0.593718429	gene-MA16_Dca013337
miR172d_2	0.593718429	gene-MA16_Dca018027
miR172d_2	0.593718429	gene-MA16_Dca020826
miR172d_2	0.593718429	gene-MA16_Dca020825
miR172d_1	0.593829576	gene-MA16_Dca021336
miR172d_1	0.593829576	gene-MA16_Dca022520
miR172d_1	0.593829576	gene-MA16_Dca006530
miR172d_1	0.593829576	gene-MA16_Dca016851
miR172d_1	0.593829576	gene-MA16_Dca022452
miR172d_1	0.593829576	gene-MA16_Dca013460
miR172d_1	0.593829576	gene-MA16_Dca022454
miR172d_1	0.593829576	gene-MA16_Dca009661
miR172d_1	0.593829576	gene-MA16_Dca013779
miR172d_1	0.593829576	gene-MA16_Dca008031
miR172d_1	0.593829576	gene-MA16_Dca002934
miR172d_1	0.593829576	gene-MA16_Dca022453
miR172d_1	0.593829576	gene-MA16_Dca020018
miR172d_1	0.593829576	gene-MA16_Dca000260
miR172d_1	0.593829576	gene-MA16_Dca010160
miR172d_1	0.593829576	gene-MA16_Dca017222
miR172d_1	0.593829576	gene-MA16_Dca002039
miR172d_1	0.593829576	gene-MA16_Dca018756

miR172d_1	0.593829576	gene-MA16_Dca013337
miR172d_1	0.593829576	gene-MA16_Dca016049
miR172d_1	0.593829576	gene-MA16_Dca015839
miR172d_1	0.593829576	gene-MA16_Dca020825
miR172d_1	0.593829576	gene-MA16_Dca020826
miR172d_1	0.593829576	gene-MA16_Dca018027
miR172d_1	0.593829576	gene-MA16_Dca020870
miR172d_1	0.593829576	gene-MA16_Dca020793
miR172d_1	0.593829576	gene-MA16_Dca011850
miR172d_1	0.593829576	gene-MA16_Dca001782
miR172d_1	0.593829576	gene-MA16_Dca013902
miR172d_1	0.593829576	gene-MA16_Dca018995
miR172d_1	0.593829576	gene-MA16_Dca004101
miR172d_1	0.593829576	gene-MA16_Dca017910
miR172d_1	0.593829576	gene-MA16_Dca007639
miR172d_1	0.593829576	gene-MA16_Dca012666
miR172d_1	0.593829576	gene-MA16_Dca022455

Note: FC indicate TPM value of miRNA in FR8/TPM value of miRNA in CK
