# Supplementary material for: RNA sequencing-based exploration of the effects of far-red light on microRNAs involved in the shade-avoidance response of D. officinale
Source: PeerJ. 2023 Mar 20;11:e15001. doi: 10.7717/peerj.15001 (PMC10035421; doi:10.7717/peerj.15001)
Supplement: Table S9 [file peerj-11-15001-s009.pdf]

Table S9 Annotation of far-red light–responsive targets

#GeneID	Nr annotation
gene-MA16_Dca015431	Premnaspirodiene oxygenase [Dendrobium catenatum]
gene-MA16_Dca000301	methyl-CpG-binding domain-containing protein 9 [Dendrobium catenatum]
gene-MA16_Dca024127	hypothetical protein MA16_Dca024127 [Dendrobium catenatum]
gene-MA16_Dca010769	Cleavage and polyadenylation specificity factor subunit 3-I [Dendrobium catenatum]
gene-MA16_Dca019438	hypothetical protein MA16_Dca019438 [Dendrobium catenatum]
gene-MA16_Dca026041	Retrovirus-related Pol polyprotein from transposon TNT 1-94 [Dendrobium catenatum]
gene-MA16_Dca027813	ATP synthase subunit a [Dendrobium catenatum]
gene-MA16_Dca014899	putative mitochondrial protein [Dendrobium catenatum]
gene-MA16_Dca005380	Receptor-like serine/threonine-protein kinase ALE2 [Dendrobium catenatum]
gene-MA16_Dca011056	alpha,alpha-trehalose-phosphate synthase [UDP-forming] 5-like isoform X1 [Dendrobium catenatum]
gene-MA16_Dca019109	Putative disease resistance RPP13-like protein 1 [Dendrobium catenatum]
gene-MA16_Dca011273	LONP2 [Dendrobium catenatum]
gene-MA16_Dca015801	Putative WEB family protein [Dendrobium catenatum]
gene-MA16_Dca022093	cysteine proteinase inhibitor 1-like [Dendrobium catenatum]
gene-MA16_Dca005153	transcription factor bHLH49-like isoform X1 [Dendrobium catenatum]
gene-MA16_Dca003177	protein LURP-one-related 8-like [Dendrobium catenatum]
gene-MA16_Dca008305	S-adenosylmethionine decarboxylase proenzyme-like [Dendrobium catenatum]
gene-MA16_Dca009226	hypothetical protein MA16_Dca009226 [Dendrobium catenatum]
gene-MA16_Dca027017	uncharacterized protein LOC110112043 [Dendrobium catenatum]
gene-MA16_Dca025277	Putative F-box protein [Dendrobium catenatum]
gene-MA16_Dca012405	hypothetical protein MA16_Dca012405 [Dendrobium catenatum]
gene-MA16_Dca009889	DEAD-box ATP-dependent RNA helicase 13 isoform X1 [Dendrobium catenatum]
gene-MA16_Dca003356	hypothetical protein MA16_Dca003356 [Dendrobium catenatum]
gene-MA16_Dca019449	hypothetical protein MA16_Dca019449 [Dendrobium catenatum]
gene-MA16_Dca001651	isoamylase 2, chloroplastic [Dendrobium catenatum]
gene-MA16_Dca008190	BTB/POZ domain-containing protein At5g03250 [Dendrobium catenatum]
gene-MA16_Dca009632	Glutamate dehydrogenase 2 [Dendrobium catenatum]

gene-MA16_Dca022962	putative E3 ubiquitin-protein ligase LIN-1 [Dendrobium catenatum]
gene-MA16_Dca017913	Serine/threonine-protein phosphatase 7 long form like [Dendrobium catenatum]
gene-MA16_Dca019929	RGG repeats nuclear RNA binding protein A-like [Dendrobium catenatum]
gene-MA16_Dca014804	peroxisomal fatty acid beta-oxidation multifunctional protein-like isoform X2 [Dendrobium catenatum]
gene-MA16_Dca008662	NAC domain-containing protein 7 [Dendrobium catenatum]
gene-MA16_Dca005183	uncharacterized protein LOC110114487 [Dendrobium catenatum]
gene-MA16_Dca021320	putative ADP-ribosylation factor GTPase-activating protein AGD5 [Dendrobium catenatum]
gene-MA16_Dca008936	uncharacterized protein LOC110102608 [Dendrobium catenatum]
gene-MA16_Dca005929	Retrovirus-related Pol polyprotein from transposon TNT 1-94 [Dendrobium catenatum]
gene-MA16_Dca011025	hypothetical protein MA16_Dca011025 [Dendrobium catenatum]
gene-MA16_Dca013677	putative mitochondrial protein [Dendrobium catenatum]
gene-MA16_Dca000652	hypothetical protein MA16_Dca000652 [Dendrobium catenatum]
gene-MA16_Dca029006	Putative disease resistance protein RGA3 [Dendrobium catenatum]
gene-MA16_Dca015802	callose synthase 10 isoform X2 [Dendrobium catenatum]
gene-MA16_Dca024512	uncharacterized protein LOC110103038 isoform X1 [Dendrobium catenatum]
gene-MA16_Dca010924	choline-phosphate cytidylyltransferase 2-like [Dendrobium catenatum]
gene-MA16_Dca014444	beta-glucuronosyltransferase GlcAT14A [Dendrobium catenatum]
gene-MA16_Dca015677	putative transcription factor bHLH041 isoform X1 [Dendrobium catenatum]
gene-MA16_Dca021951	hypothetical protein MA16_Dca021951 [Dendrobium catenatum]
gene-MA16_Dca006858	lactation elevated protein 1 isoform X1 [Dendrobium catenatum]
gene-MA16_Dca021243	U-box domain-containing protein 33 [Dendrobium catenatum]
gene-MA16_Dca001757	probable tocopherol cyclase, chloroplastic isoform X1 [Dendrobium catenatum]
gene-MA16_Dca015808	Dynamin-related protein 1E [Dendrobium catenatum]
gene-MA16_Dca020366	hypothetical protein MA16_Dca020366 [Dendrobium catenatum]
gene-MA16_Dca012653	hypothetical protein MA16_Dca012653 [Dendrobium catenatum]
gene-MA16_Dca015758	putative mitochondrial protein [Dendrobium catenatum]
gene-MA16_Dca017025	Rop guanine nucleotide exchange factor 1 [Dendrobium catenatum]
gene-MA16_Dca017867	uncharacterized protein LOC110095911 [Dendrobium catenatum]
gene-MA16_Dca009494	Glutamate receptor 2.7 [Dendrobium catenatum]
gene-MA16_Dca024866	protein FAR1-RELATED SEQUENCE 11-like [Dendrobium catenatum]

gene-MA16_Dca024847	hypothetical protein MA16_Dca024847 [Dendrobium catenatum]
gene-MA16_Dca014760	uncharacterized protein LOC110103117 [Dendrobium catenatum]
gene-MA16_Dca008963	ARF guanine-nucleotide exchange factor GNOM-like [Dendrobium catenatum]
gene-MA16_Dca017908	probable trehalose-phosphate phosphatase 6 isoform X2 [Dendrobium catenatum]
gene-MA16_Dca007955	Protein ROOT HAIR DEFECTIVE 3 [Dendrobium catenatum]
gene-MA16_Dca002200	hypothetical protein MA16_Dca002200 [Dendrobium catenatum]
gene-MA16_Dca004014	Retrovirus-related Pol polyprotein from transposon TNT 1-94 [Dendrobium catenatum]
gene-MA16_Dca027485	hypothetical protein MA16_Dca027485 [Dendrobium catenatum]
gene-MA16_Dca008845	reticulon-like protein B2 [Dendrobium catenatum]
gene-MA16_Dca001177	dof zinc finger protein DOF5.7-like [Dendrobium catenatum]
gene-MA16_Dca024909	Growth-regulating factor 5 [Dendrobium catenatum]
gene-MA16_Dca009811	Actin-depolymerizing factor 4 [Dendrobium catenatum]
gene-MA16_Dca001231	Outer envelope pore protein 24, chloroplastic [Dendrobium catenatum]
gene-MA16_Dca005762	gibberellin 20-oxidase-like protein [Dendrobium catenatum]
gene-MA16_Dca019102	Organic cation/carnitine transporter 2 [Dendrobium catenatum]
gene-MA16_Dca014665	SNAP25 likeous protein SNAP33 [Dendrobium catenatum]
gene-MA16_Dca010245	transcription factor TCP4-like [Dendrobium catenatum]
gene-MA16_Dca006555	protein NRT1/ PTR FAMILY 5.8-like [Dendrobium catenatum]
gene-MA16_Dca019311	rRNA-processing protein UTP23 homolog [Dendrobium catenatum]
gene-MA16_Dca016490	hypothetical protein MA16_Dca016490 [Dendrobium catenatum]
gene-MA16_Dca006386	probable serine/threonine-protein kinase At1g54610 [Dendrobium catenatum]
gene-MA16_Dca003694	serine carboxypeptidase-like 45 [Dendrobium catenatum]
gene-MA16_Dca009651	uncharacterized protein LOC110115649 [Dendrobium catenatum]
gene-MA16_Dca022596	L-gulonolactone oxidase 2-like [Dendrobium catenatum]
gene-MA16_Dca003304	Retrovirus-related Pol polyprotein from transposon TNT 1-94 [Dendrobium catenatum]
gene-MA16_Dca009952	DNA-damage-repair/toleration protein DRT100 [Dendrobium catenatum]
gene-MA16_Dca020860	hypothetical protein MA16_Dca020860 [Dendrobium catenatum]
gene-MA16_Dca027407	hypothetical protein MA16_Dca027407 [Dendrobium catenatum]
gene-MA16_Dca010827	Aspartic proteinase Asp1 [Dendrobium catenatum]
gene-MA16_Dca014237	classical arabinogalactan protein 9-like [Dendrobium catenatum]

gene-MA16_Dca024947	Formin-like protein 6 [Dendrobium catenatum]
gene-MA16_Dca008993	Isoflavone 2'-hydroxylase [Dendrobium catenatum]
gene-MA16_Dca010838	putative zinc finger protein [Dendrobium catenatum]
gene-MA16_Dca013148	hypothetical protein MA16_Dca013148 [Dendrobium catenatum]
gene-MA16_Dca005161	syntaxin-32-like isoform X1 [Dendrobium catenatum]
gene-MA16_Dca016304	65-kDa microtubule-associated protein 7 [Dendrobium catenatum]
gene-MA16_Dca008496	Putative disease resistance protein RGA4 [Dendrobium catenatum]
gene-MA16_Dca022302	uncharacterized protein LOC110097256 [Dendrobium catenatum]
gene-MA16_Dca025299	protein IQ-DOMAIN 32-like [Dendrobium catenatum]
gene-MA16_Dca014786	uncharacterized protein LOC110095165 [Dendrobium catenatum]
gene-MA16_Dca027164	pentatricopeptide repeat-containing protein At1g71490 [Dendrobium catenatum]
gene-MA16_Dca005596	protein DETOXIFICATION 35 isoform X1 [Dendrobium catenatum]
gene-MA16_Dca015149	protein transport protein SEC23-like [Dendrobium catenatum]
gene-MA16_Dca019307	uncharacterized protein At1g04910-like isoform X2 [Dendrobium catenatum]
gene-MA16_Dca002529	binding partner of ACD11 1-like isoform X1 [Dendrobium catenatum]
gene-MA16_Dca005305	hypothetical protein MA16_Dca005305 [Dendrobium catenatum]
gene-MA16_Dca004093	Mitogen-activated protein kinase kinase kinase 1 [Dendrobium catenatum]
gene-MA16_Dca013209	CCR4-NOT transcription complex subunit 1 isoform X1 [Dendrobium catenatum]
gene-MA16_Dca025112	hypothetical protein MA16_Dca025112 [Dendrobium catenatum]
gene-MA16_Dca010365	Guanine nucleotide-binding protein subunit beta [Dendrobium catenatum]
gene-MA16_Dca022320	receptor-like protein kinase FERONIA [Dendrobium catenatum]
gene-MA16_Dca000787	Serine/threonine-protein phosphatase 7 long form like [Dendrobium catenatum]
gene-MA16_Dca021624	hypothetical protein MA16_Dca021624 [Dendrobium catenatum]
gene-MA16_Dca000146	probable ethylene response sensor 1 [Dendrobium catenatum]
gene-MA16_Dca015539	40S ribosomal protein S6-like [Dendrobium catenatum]
gene-MA16_Dca015287	transcription factor TCP4-like [Dendrobium catenatum]
gene-MA16_Dca000081	hypothetical protein MA16_Dca000081 [Dendrobium catenatum]
gene-MA16_Dca005043	probable WRKY transcription factor 70 isoform X2 [Dendrobium catenatum]
gene-MA16_Dca002985	hypothetical protein MA16_Dca002985 [Dendrobium catenatum]
gene-MA16_Dca017382	hypothetical protein MA16_Dca017382 [Dendrobium catenatum]

gene-MA16_Dca007808	Retrovirus-related Pol polyprotein from transposon TNT 1-94 [Dendrobium catenatum]
gene-MA16_Dca015381	uncharacterized protein LOC110101588 isoform X2 [Dendrobium catenatum]
gene-MA16_Dca008350	probable protein phosphatase 2C 11 isoform X1 [Dendrobium catenatum]
gene-MA16_Dca009267	hypothetical protein MA16_Dca009267 [Dendrobium catenatum]
gene-MA16_Dca021423	lysine-specific demethylase JMJ25 isoform X2 [Dendrobium catenatum]
gene-MA16_Dca023411	Protein AIG1 [Dendrobium catenatum]
gene-MA16_Dca011928	Retrovirus-related Pol polyprotein from transposon TNT 1-94 [Dendrobium catenatum]
gene-MA16_Dca010093	Protein AIG1 [Dendrobium catenatum]
gene-MA16_Dca028734	uncharacterized protein LOC110097469 [Dendrobium catenatum]
gene-MA16_Dca028117	Retrovirus-related Pol polyprotein from transposon TNT 1-94 [Dendrobium catenatum]
gene-MA16_Dca007395	S-adenosylmethionine decarboxylase proenzyme [Dendrobium catenatum]
gene-MA16_Dca004056	pentatricopeptide repeat-containing protein At1g11290, chloroplastic [Dendrobium catenatum]
gene-MA16_Dca003704	translocon-associated protein subunit beta [Dendrobium catenatum]
gene-MA16_Dca010016	hypothetical protein MA16_Dca010016 [Dendrobium catenatum]
gene-MA16_Dca006179	WAT1-related protein [Dendrobium catenatum]
gene-MA16_Dca000732	serine/threonine-protein kinase HT1-like isoform X1 [Dendrobium catenatum]
gene-MA16_Dca023779	UNC93-like protein 2 [Dendrobium catenatum]
gene-MA16_Dca001351	Protein PRD1 [Dendrobium catenatum]
gene-MA16_Dca022880	hypothetical protein MA16_Dca022880 [Dendrobium catenatum]
gene-MA16_Dca012643	Cation/calcium exchanger 1 [Dendrobium catenatum]
gene-MA16_Dca005906	125 kDa kinesin-related protein [Dendrobium catenatum]
gene-MA16_Dca014632	probable cellulose synthase A catalytic subunit 5 [UDP-forming] [Dendrobium catenatum]
gene-MA16_Dca009980	transcriptional adapter ADA2-like [Dendrobium catenatum]
gene-MA16_Dca006730	protein RNA-directed DNA methylation 3 [Dendrobium catenatum]
gene-MA16_Dca002420	CMP-sialic acid transporter 4 [Dendrobium catenatum]
gene-MA16_Dca002388	hypothetical protein MA16_Dca002388 [Dendrobium catenatum]
gene-MA16_Dca017151	Deoxyribodipyrimidine photo-lyase [Dendrobium catenatum]
gene-MA16_Dca014284	translocase of chloroplast 159, chloroplastic [Dendrobium catenatum]
gene-MA16_Dca020166	WEB family protein At2g38370-like [Dendrobium catenatum]
gene-MA16_Dca000685	probable ubiquitin-conjugating enzyme E2 37 isoform X1 [Dendrobium catenatum]

gene-MA16_Dca001826	peroxidase 40 [Dendrobium catenatum]
gene-MA16_Dca020372	putative mitochondrial protein [Dendrobium catenatum]
gene-MA16_Dca010139	mechanosensitive ion channel protein 6-like [Dendrobium catenatum]
gene-MA16_Dca022653	putative mitochondrial protein [Dendrobium catenatum]
gene-MA16_Dca014225	hypothetical protein MA16_Dca014225 [Dendrobium catenatum]
gene-MA16_Dca009935	SNARE-interacting protein KEULE [Dendrobium catenatum]
gene-MA16_Dca002055	hypothetical protein MA16_Dca002055 [Dendrobium catenatum]
gene-MA16_Dca021663	pectinesterase-like [Dendrobium catenatum]
gene-MA16_Dca021825	Petal death protein [Dendrobium catenatum]
gene-MA16_Dca027654	hypothetical protein MA16_Dca027654 [Dendrobium catenatum]
gene-MA16_Dca024366	hypothetical protein MA16_Dca024366 [Dendrobium catenatum]
gene-MA16_Dca000708	Autophagy-related protein 8g [Dendrobium catenatum]
gene-MA16_Dca009642	Retrovirus-related Pol polyprotein from transposon TNT 1-94 [Dendrobium catenatum]
gene-MA16_Dca018321	hypothetical protein MA16_Dca018321 [Dendrobium catenatum]
gene-MA16_Dca020021	mitogen-activated protein kinase kinase kinase 5-like [Dendrobium catenatum]
gene-MA16_Dca009619	hypothetical protein MA16_Dca009619 [Dendrobium catenatum]
gene-MA16_Dca020304	probable purine permease 11 [Dendrobium catenatum]
gene-MA16_Dca014769	Retrovirus-related Pol polyprotein from transposon TNT 1-94 [Dendrobium catenatum]
gene-MA16_Dca014182	UDP-glycosyltransferase 73C4 [Dendrobium catenatum]
gene-MA16_Dca023983	aspartyl protease family protein 1-like isoform X1 [Dendrobium catenatum]
gene-MA16_Dca002239	Abscisic stress-ripening protein 1 [Dendrobium catenatum]
gene-MA16_Dca001711	mannose-specific lectin 2-like [Dendrobium catenatum]
gene-MA16_Dca009629	RING-H2 finger protein ATL80 [Dendrobium catenatum]
gene-MA16_Dca011166	hypothetical protein MA16_Dca011166 [Dendrobium catenatum]
gene-MA16_Dca009386	hypothetical protein MA16_Dca009386 [Dendrobium catenatum]
gene-MA16_Dca007509	hypothetical protein MA16_Dca007509 [Dendrobium catenatum]
gene-MA16_Dca016099	Eukaryotic translation initiation factor 5A-4 [Dendrobium catenatum]
gene-MA16_Dca018835	hypothetical protein MA16_Dca018835 [Dendrobium catenatum]
gene-MA16_Dca014311	RNA-directed DNA polymerase [Dendrobium catenatum]
gene-MA16_Dca002354	stromal cell-derived factor 2-like protein [Dendrobium catenatum]

gene-MA16_Dca014211	hypothetical protein MA16_Dca014211 [Dendrobium catenatum]
gene-MA16_Dca018562	transcription factor MYB44-like [Dendrobium catenatum]
gene-MA16_Dca026102	Starch synthase 1, chloroplastic/amyloplastic [Dendrobium catenatum]
gene-MA16_Dca006432	galactoside 2-alpha-L-fucosyltransferase-like [Dendrobium catenatum]
gene-MA16_Dca002323	Piriformospora indica-insensitive protein 2 [Dendrobium catenatum]
gene-MA16_Dca012960	pectinesterase-like [Dendrobium catenatum]
gene-MA16_Dca001986	pentatricopeptide repeat-containing protein At2g27800, mitochondrial [Dendrobium catenatum]
gene-MA16_Dca026603	hypothetical protein MA16_Dca026603 [Dendrobium catenatum]
gene-MA16_Dca007071	hypothetical protein MA16_Dca007071 [Dendrobium catenatum]
gene-MA16_Dca019591	probable protein phosphatase 2C 12 [Dendrobium catenatum]
gene-MA16_Dca018434	hypothetical protein MA16_Dca018434 [Dendrobium catenatum]
gene-MA16_Dca007765	uncharacterized protein LOC110093255 [Dendrobium catenatum]
gene-MA16_Dca012903	hypothetical protein MA16_Dca012903 [Dendrobium catenatum]
gene-MA16_Dca028951	hypothetical protein MA16_Dca028951 [Dendrobium catenatum]
gene-MA16_Dca005171	Transcription factor GAMYB [Dendrobium catenatum]
gene-MA16_Dca006711	checkpoint serine/threonine-protein kinase [Dendrobium catenatum]
gene-MA16_Dca014967	Protein FAR1-RELATED SEQUENCE 5 [Dendrobium catenatum]
gene-MA16_Dca018364	protein gamma response 1 isoform X1 [Dendrobium catenatum]
gene-MA16_Dca013757	hypothetical protein MA16_Dca013757 [Dendrobium catenatum]
gene-MA16_Dca025628	hypothetical protein MA16_Dca025628 [Dendrobium catenatum]
gene-MA16_Dca014797	ER membrane protein complex subunit 1 isoform X1 [Dendrobium catenatum]
gene-MA16_Dca005159	NAD-dependent protein deacetylase SRT1 isoform X1 [Dendrobium catenatum]
gene-MA16_Dca021950	Mitogen-activated protein kinase kinase kinase YODA [Dendrobium catenatum]
gene-MA16_Dca024245	trifunctional UDP-glucose 4,6-dehydratase/UDP-4-keto-6-deoxy-D-glucose 3,5-epimerase/UDP-4-keto-L-rhamnose-reductase RHM1 [Dendrobium catenatum]
gene-MA16_Dca025306	Putative ribonuclease H protein [Dendrobium catenatum]
gene-MA16_Dca006187	ATP-dependent helicase BRM isoform X1 [Dendrobium catenatum]
gene-MA16_Dca008491	Flavonoid 3'-monooxygenase [Dendrobium catenatum]
gene-MA16_Dca014143	actin-related protein 2/3 complex subunit 1B-like [Dendrobium catenatum]
gene-MA16_Dca002786	hypothetical protein MA16_Dca002786 [Dendrobium catenatum]

gene-MA16_Dca001647	LRR receptor-like serine/threonine-protein kinase FEI 1 isoform X1 [Dendrobium catenatum]
gene-MA16_Dca028203	hypothetical protein MA16_Dca028203 [Dendrobium catenatum]
gene-MA16_Dca006426	bZIP transcription factor TRAB1-like [Dendrobium catenatum]
gene-MA16_Dca016487	Callose synthase 2 [Dendrobium catenatum]
gene-MA16_Dca011344	hypothetical protein MA16_Dca011344 [Dendrobium catenatum]
gene-MA16_Dca003622	uncharacterized protein LOC110097182 [Dendrobium catenatum]
gene-MA16_Dca020607	hypothetical protein MA16_Dca020607 [Dendrobium catenatum]
gene-MA16_Dca026755	Polypyrimidine tract-binding protein like 2 [Dendrobium catenatum]
gene-MA16_Dca008618	hypothetical protein MA16_Dca008618 [Dendrobium catenatum]
gene-MA16_Dca009185	uncharacterized protein LOC110115467 [Dendrobium catenatum]
gene-MA16_Dca000436	pentatricopeptide repeat-containing protein At4g39530-like [Dendrobium catenatum]
gene-MA16_Dca009990	brefeldin A-inhibited guanine nucleotide-exchange protein 1 [Dendrobium catenatum]
gene-MA16_Dca027333	hypothetical protein MA16_Dca027333 [Dendrobium catenatum]
gene-MA16_Dca003098	lysine-specific demethylase 3 [Dendrobium catenatum]
gene-MA16_Dca007933	Alpha,alpha-trehalose-phosphate synthase [UDP-forming] 6 [Dendrobium catenatum]
gene-MA16_Dca008028	uncharacterized protein LOC110111681 [Dendrobium catenatum]
gene-MA16_Dca003280	chitinase 2-like isoform X1 [Dendrobium catenatum]
gene-MA16_Dca027889	hypothetical protein MA16_Dca027889 [Dendrobium catenatum]
gene-MA16_Dca007177	glycerophosphodiester phosphodiesterase GDPDL4-like [Dendrobium catenatum]
gene-MA16_Dca009036	auxin response factor 18-like [Dendrobium catenatum]
gene-MA16_Dca006620	Phytochromobilin:ferredoxin oxidoreductase, chloroplastic [Dendrobium catenatum]
gene-MA16_Dca022454	Receptor-like serine/threonine-protein kinase SD1-6 [Dendrobium catenatum]
gene-MA16_Dca012689	Protein FAR1-RELATED SEQUENCE 5 [Dendrobium catenatum]
gene-MA16_Dca014504	Indole-3-acetic acid-amido synthetase GH3.17 [Dendrobium catenatum]
gene-MA16_Dca018053	WAT1-related protein At5g47470-like isoform X1 [Dendrobium catenatum]
gene-MA16_Dca021414	probable serine/threonine-protein kinase WNK9 isoform X2 [Dendrobium catenatum]
gene-MA16_Dca020018	hypothetical protein MA16_Dca020018 [Dendrobium catenatum]
gene-MA16_Dca023125	putative mannan endo-1,4-beta-mannosidase 9 [Dendrobium catenatum]
gene-MA16_Dca005077	hypothetical protein MA16_Dca005077 [Dendrobium catenatum]
gene-MA16_Dca019179	uncharacterized protein LOC110093585 [Dendrobium catenatum]

gene-MA16_Dca013703	probable ubiquitin-like-specific protease 2B [Dendrobium catenatum]
gene-MA16_Dca014784	3-ketoacyl-CoA synthase 11-like [Dendrobium catenatum]
gene-MA16_Dca024999	Uncharacterized protein MA16_Dca024999 [Dendrobium catenatum]
gene-MA16_Dca004322	hypothetical protein MA16_Dca004322 [Dendrobium catenatum]
gene-MA16_Dca006051	Transcription factor TCP2 [Dendrobium catenatum]
gene-MA16_Dca021724	hypothetical protein MA16_Dca021724 [Dendrobium catenatum]
gene-MA16_Dca001366	hypothetical protein MA16_Dca001366 [Dendrobium catenatum]
gene-MA16_Dca005617	DEAD-box ATP-dependent RNA helicase 3, chloroplastic [Dendrobium catenatum]
gene-MA16_Dca020825	ribosomal RNA methyltransferase Nop2 [Dendrobium catenatum]
gene-MA16_Dca015726	pentatricopeptide repeat-containing protein At1g71460, chloroplastic [Dendrobium catenatum]
gene-MA16_Dca015909	regulatory-associated protein of TOR 2 isoform X2 [Dendrobium catenatum]
gene-MA16_Dca014413	hypothetical protein MA16_Dca014413 [Dendrobium catenatum]
gene-MA16_Dca014018	Cytochrome P450 78A4 [Dendrobium catenatum]
gene-MA16_Dca027817	acylaminoacyl-peptidase [Dendrobium catenatum]
gene-MA16_Dca016652	Protein HASTY 1 [Dendrobium catenatum]
gene-MA16_Dca015353	Nodulation-signaling pathway 2 protein [Dendrobium catenatum]
gene-MA16_Dca008843	late embryogenesis abundant protein At1g64065-like [Dendrobium catenatum]
gene-MA16_Dca021085	beta-glucosidase 1-like [Dendrobium catenatum]
gene-MA16_Dca019111	hypothetical protein MA16_Dca019111 [Dendrobium catenatum]
gene-MA16_Dca011522	hypothetical protein MA16_Dca011522 [Dendrobium catenatum]
gene-MA16_Dca010045	Retrovirus-related Pol polyprotein from transposon TNT 1-94 [Dendrobium catenatum]
gene-MA16_Dca008955	Polyadenylate-binding protein 2 [Dendrobium catenatum]
gene-MA16_Dca015196	Mediator of RNA polymerase II transcription subunit 15a [Dendrobium catenatum]
gene-MA16_Dca015008	myosin-10 [Dendrobium catenatum]
gene-MA16_Dca007252	serine carboxypeptidase-like 27 [Dendrobium catenatum]
gene-MA16_Dca021760	pentatricopeptide repeat-containing protein At5g18475-like [Dendrobium catenatum]
gene-MA16_Dca001555	Disease resistance protein RGA2 [Dendrobium catenatum]
gene-MA16_Dca002934	ethylene-overproduction protein 1 [Dendrobium catenatum]
gene-MA16_Dca001955	transaldolase [Dendrobium catenatum]
gene-MA16_Dca024373	hypothetical protein MA16_Dca024373 [Dendrobium catenatum]

gene-MA16_Dca012780	Formin-like protein 5 [Dendrobium catenatum]
gene-MA16_Dca005927	hypothetical protein MA16_Dca005927 [Dendrobium catenatum]
gene-MA16_Dca014937	hypothetical protein MA16_Dca029169 [Dendrobium catenatum]
gene-MA16_Dca001387	hypothetical protein MA16_Dca001387 [Dendrobium catenatum]
gene-MA16_Dca015293	hypothetical protein MA16_Dca015293 [Dendrobium catenatum]
gene-MA16_Dca005799	serine/threonine-protein kinase RIPK-like [Dendrobium catenatum]
gene-MA16_Dca001114	hypothetical protein MA16_Dca001114 [Dendrobium catenatum]
gene-MA16_Dca016309	probable transcription factor RL9 isoform X1 [Dendrobium catenatum]
gene-MA16_Dca011426	scarecrow-like protein 9 [Dendrobium catenatum]
gene-MA16_Dca009793	uncharacterized protein LOC110092030 isoform X2 [Dendrobium catenatum]
gene-MA16_Dca007731	hypothetical protein MA16_Dca007731 [Dendrobium catenatum]
gene-MA16_Dca008798	calcium-dependent protein kinase 29-like [Dendrobium catenatum]
gene-MA16_Dca007682	pentatricopeptide repeat-containing protein At4g17616 [Dendrobium catenatum]
gene-MA16_Dca016237	hypothetical protein MA16_Dca016237 [Dendrobium catenatum]
gene-MA16_Dca014863	hypothetical protein MA16_Dca014863 [Dendrobium catenatum]
gene-MA16_Dca021441	hypothetical protein MA16_Dca021441 [Dendrobium catenatum]
gene-MA16_Dca004503	Regulator of nonsense transcripts 1 like [Dendrobium catenatum]
gene-MA16_Dca003585	putative cellulose synthase A catalytic subunit 8 [UDP-forming] [Dendrobium catenatum]
gene-MA16_Dca014181	UDP-glycosyltransferase 73D1-like [Dendrobium catenatum]
gene-MA16_Dca005521	UDP-glucose 4-epimerase GEPI48-like [Dendrobium catenatum]
gene-MA16_Dca009572	MATE efflux family protein 3, chloroplastic [Dendrobium catenatum]
gene-MA16_Dca010939	uncharacterized protein LOC110116329 isoform X2 [Dendrobium catenatum]
gene-MA16_Dca024707	polygalacturonate 4-alpha-galacturonosyltransferase-like [Dendrobium catenatum]
gene-MA16_Dca021050	C2 and GRAM domain-containing protein At1g03370 [Dendrobium catenatum]
gene-MA16_Dca019405	Protein argonaute 1B [Dendrobium catenatum]
gene-MA16_Dca019042	hypothetical protein MA16_Dca019042 [Dendrobium catenatum]
gene-MA16_Dca022532	protein RRP6-like 3 isoform X1 [Dendrobium catenatum]
gene-MA16_Dca026583	cytochrome P450 71A1-like [Dendrobium catenatum]
gene-MA16_Dca027910	hypothetical protein MA16_Dca027910 [Dendrobium catenatum]
gene-MA16_Dca000603	Myb family transcription factor APL [Dendrobium catenatum]

gene-MA16_Dca026857	hypothetical protein MA16_Dca026857 [Dendrobium catenatum]
gene-MA16_Dca010603	transcription factor MYB97-like [Dendrobium catenatum]
gene-MA16_Dca025598	hypothetical protein MA16_Dca025598 [Dendrobium catenatum]
gene-MA16_Dca016370	L-type lectin-domain containing receptor kinase IX.1-like [Dendrobium catenatum]
gene-MA16_Dca008399	putative phospholipid-transporting ATPase 9 isoform X1 [Dendrobium catenatum]
gene-MA16_Dca023953	hypothetical protein MA16_Dca023953 [Dendrobium catenatum]
gene-MA16_Dca017780	MLO-like protein 8 [Dendrobium catenatum]
gene-MA16_Dca013776	Pentatricopeptide repeat-containing protein [Dendrobium catenatum]
gene-MA16_Dca006650	E3 ubiquitin-protein ligase PUB23-like [Dendrobium catenatum]
gene-MA16_Dca016049	AP2-like ethylene-responsive transcription factor TOE3 isoform X1 [Dendrobium catenatum]
gene-MA16_Dca001025	probable prefoldin subunit 2 [Dendrobium catenatum]
gene-MA16_Dca002488	Putative E3 ubiquitin-protein ligase XBAT34 [Dendrobium catenatum]
gene-MA16_Dca008335	Lysine-specific demethylase REF6 [Dendrobium catenatum]
gene-MA16_Dca024342	KH domain-containing protein SPIN1 [Dendrobium catenatum]
gene-MA16_Dca025479	Putative ribonuclease H protein [Dendrobium catenatum]
gene-MA16_Dca027456	ATP-dependent DNA helicase RecG [Dendrobium catenatum]
gene-MA16_Dca020570	E3 ubiquitin-protein ligase ATL4-like [Dendrobium catenatum]
gene-MA16_Dca001523	Transcription factor ILR3 [Dendrobium catenatum]
gene-MA16_Dca011122	PsbB mRNA maturation factor Mbb1, chloroplastic [Dendrobium catenatum]
gene-MA16_Dca006661	hypothetical protein MA16_Dca006661 [Dendrobium catenatum]
gene-MA16_Dca012769	Vacuolar cation/proton exchanger 1a [Dendrobium catenatum]
gene-MA16_Dca015496	UPF0481 protein At3g47200-like [Dendrobium catenatum]
gene-MA16_Dca020556	E3 ubiquitin-protein ligase ATL4-like [Dendrobium catenatum]
gene-MA16_Dca000383	protein ELF4-LIKE 3-like isoform X2 [Dendrobium catenatum]
gene-MA16_Dca003396	RNA-binding protein 39-like [Dendrobium catenatum]
gene-MA16_Dca023030	hypothetical protein MA16_Dca023030 [Dendrobium catenatum]
gene-MA16_Dca004818	hypothetical protein MA16_Dca004818 [Dendrobium catenatum]
gene-MA16_Dca024281	exocyst complex component EXO70A1-like [Dendrobium catenatum]
gene-MA16_Dca026312	Putative disease resistance protein RGA4 [Dendrobium catenatum]
gene-MA16_Dca020179	hypothetical protein MA16_Dca020179 [Dendrobium catenatum]

gene-MA16_Dca024504	Serine/threonine-protein kinase KIPK [Dendrobium catenatum]
gene-MA16_Dca016271	hypothetical protein MA16_Dca016271 [Dendrobium catenatum]
gene-MA16_Dca008889	hypothetical protein MA16_Dca008889 [Dendrobium catenatum]
gene-MA16_Dca023559	catenatum]
gene-MA16_Dca005216	pentatricopeptide repeat-containing protein At1g77360, mitochondrial-like [Dendrobium catenatum]
gene-MA16_Dca023726	hypothetical protein MA16_Dca023726 [Dendrobium catenatum]
gene-MA16_Dca028508	Short-chain dehydrogenase TIC 32, chloroplastic [Dendrobium catenatum]
gene-MA16_Dca011125	cysteine-rich receptor-like protein kinase 42 [Dendrobium catenatum]
gene-MA16_Dca016354	Phosphopantothenate--cysteine ligase 2 [Dendrobium catenatum]
gene-MA16_Dca011276	two-component response regulator-like PRR95 isoform X1 [Dendrobium catenatum]
gene-MA16_Dca010402	basic leucine zipper 19-like [Dendrobium catenatum]
gene-MA16_Dca004453	pectin acetylesterase 10-like isoform X1 [Dendrobium catenatum]
gene-MA16_Dca006410	receptor-like protein kinase HERK 1 [Dendrobium catenatum]
gene-MA16_Dca015291	Taxadien-5-alpha-ol O-acetyltransferase [Dendrobium catenatum]
gene-MA16_Dca007882	uncharacterized protein LOC110106019 isoform X1 [Dendrobium catenatum]
gene-MA16_Dca016284	hypothetical protein MA16_Dca016284 [Dendrobium catenatum]
gene-MA16_Dca015838	Triacylglycerol lipase 2 [Dendrobium catenatum]
gene-MA16_Dca006235	hypothetical protein MA16_Dca006235 [Dendrobium catenatum]
gene-MA16_Dca002971	hypothetical protein MA16_Dca002971 [Dendrobium catenatum]
gene-MA16_Dca004658	Polyadenylate-binding protein 5 [Dendrobium catenatum]
gene-MA16_Dca004718	hypothetical protein MA16_Dca004718 [Dendrobium catenatum]
gene-MA16_Dca022451	receptor-like serine/threonine-protein kinase SD1-8 isoform X1 [Dendrobium catenatum]
gene-MA16_Dca007981	transcription termination factor MTERF4, chloroplastic-like [Dendrobium catenatum]
gene-MA16_Dca017513	hypothetical protein MA16_Dca017513 [Dendrobium catenatum]
gene-MA16_Dca001444	Retrovirus-related Pol polyprotein from transposon TNT 1-94 [Dendrobium catenatum]
gene-MA16_Dca014794	Uncharacterized protein ycf56 [Dendrobium catenatum]
gene-MA16_Dca022316	hypothetical protein MA16_Dca022316 [Dendrobium catenatum]
gene-MA16_Dca019609	Vacuolar protein sorting-associated protein 35A [Dendrobium catenatum]
gene-MA16_Dca023499	G-type lectin S-receptor-like serine/threonine-protein kinase RLK1 [Dendrobium catenatum]
gene-MA16_Dca025804	hypothetical protein MA16_Dca025804 [Dendrobium catenatum]

gene-MA16_Dca004622	Retrovirus-related Pol polyprotein from transposon TNT 1-94 [Dendrobium catenatum]
gene-MA16_Dca003816	catenatum]
gene-MA16_Dca016956	Cytokinin riboside 5'-monophosphate phosphoribohydrolase LOG8 [Dendrobium catenatum]
gene-MA16_Dca012782	uncharacterized protein LOC110103512 [Dendrobium catenatum]
gene-MA16_Dca010563	succinate-semialdehyde dehydrogenase, mitochondrial isoform X1 [Dendrobium catenatum]
gene-MA16_Dca026802	ras-related protein RABF1 [Dendrobium catenatum]
gene-MA16_Dca009002	alkylated DNA repair protein alkB 5 [Dendrobium catenatum]
gene-MA16_Dca008528	hypothetical protein MA16_Dca008528 [Dendrobium catenatum]
gene-MA16_Dca017188	transcription factor RAX2-like [Dendrobium catenatum]
gene-MA16_Dca017950	hypothetical protein MA16_Dca017950 [Dendrobium catenatum]
gene-MA16_Dca013441	hypothetical protein MA16_Dca013441 [Dendrobium catenatum]
gene-MA16_Dca002864	hypothetical protein MA16_Dca002864 [Dendrobium catenatum]
gene-MA16_Dca015837	hypothetical protein MA16_Dca015837 [Dendrobium catenatum]
gene-MA16_Dca024325	uncharacterized protein LOC110102882 [Dendrobium catenatum]
gene-MA16_Dca003347	pentatricopeptide repeat-containing protein At1g03100, mitochondrial [Dendrobium catenatum]
gene-MA16_Dca024902	hypothetical protein MA16_Dca024902 [Dendrobium catenatum]
gene-MA16_Dca028588	agamous-like MADS-box protein AGL65 [Dendrobium catenatum]
gene-MA16_Dca002338	hypothetical protein MA16_Dca002338 [Dendrobium catenatum]
gene-MA16_Dca002218	Protein ASPARTIC PROTEASE IN GUARD CELL 2 [Dendrobium catenatum]
gene-MA16_Dca002355	DNA mismatch repair protein MSH6 [Dendrobium catenatum]
gene-MA16_Dca019383	hypothetical protein MA16_Dca019383 [Dendrobium catenatum]
gene-MA16_Dca012946	hypothetical protein MA16_Dca012946 [Dendrobium catenatum]
gene-MA16_Dca025511	Sulfate transporter 2.1 [Dendrobium catenatum]
gene-MA16_Dca000737	hypothetical protein MA16_Dca000737 [Dendrobium catenatum]
gene-MA16_Dca028471	Retrovirus-related Pol polyprotein from transposon TNT 1-94 [Dendrobium catenatum]
gene-MA16_Dca021334	hypothetical protein MA16_Dca021334 [Dendrobium catenatum]
gene-MA16_Dca021158	Clustered mitochondria protein [Dendrobium catenatum]
gene-MA16_Dca009910	uncharacterized protein LOC110113468 isoform X1 [Dendrobium catenatum]
gene-MA16_Dca023610	hypothetical protein MA16_Dca023610 [Dendrobium catenatum]
gene-MA16_Dca028489	hypothetical protein MA16_Dca028489 [Dendrobium catenatum]

gene-MA16_Dca018212	Cell cycle checkpoint protein RAD17 [Dendrobium catenatum]
gene-MA16_Dca010165	pentatricopeptide repeat-containing protein At4g33170 isoform X1 [Dendrobium catenatum]
gene-MA16_Dca022005	putative mitochondrial protein [Dendrobium catenatum]
gene-MA16_Dca022181	asparagine--tRNA ligase, cytoplasmic 3-like [Dendrobium catenatum]
gene-MA16_Dca002377	auxin transport protein BIG [Dendrobium catenatum]
gene-MA16_Dca005798	transcription initiation factor TFIID subunit 9 [Dendrobium catenatum]
gene-MA16_Dca015824	hypothetical protein MA16_Dca015824 [Dendrobium catenatum]
gene-MA16_Dca019243	hypothetical protein MA16_Dca019243 [Dendrobium catenatum]
gene-MA16_Dca025577	uncharacterized protein LOC110099282 [Dendrobium catenatum]
gene-MA16_Dca021763	cysteine-rich receptor-like protein kinase 15 [Dendrobium catenatum]
gene-MA16_Dca026790	Protein PAIR1 [Dendrobium catenatum]
gene-MA16_Dca019261	exopolygalacturonase-like [Dendrobium catenatum]
gene-MA16_Dca027012	IAA-alanine resistance protein 1 [Dendrobium catenatum]
gene-MA16_Dca009003	uncharacterized protein LOC110097447 [Dendrobium catenatum]
gene-MA16_Dca012661	hypothetical protein MA16_Dca012661 [Dendrobium catenatum]
gene-MA16_Dca004361	hypothetical protein MA16_Dca004361 [Dendrobium catenatum]
gene-MA16_Dca012983	uncharacterized protein LOC110107094 [Dendrobium catenatum]
gene-MA16_Dca003651	ribonuclease P protein subunit p29 [Dendrobium catenatum]
gene-MA16_Dca020115	putative ADP-ribosylation factor GTPase-activating protein AGD11 [Dendrobium catenatum]
gene-MA16_Dca026047	hypothetical protein MA16_Dca026047 [Dendrobium catenatum]
gene-MA16_Dca003966	uncharacterized protein LOC110107815 [Dendrobium catenatum]
gene-MA16_Dca014606	Ribose-phosphate pyrophosphokinase 1 [Dendrobium catenatum]
gene-MA16_Dca026042	hypothetical protein MA16_Dca026042 [Dendrobium catenatum]
gene-MA16_Dca014741	transmembrane 9 superfamily member 8-like [Dendrobium catenatum]
gene-MA16_Dca024861	uncharacterized protein LOC110093686 [Dendrobium catenatum]
gene-MA16_Dca010817	putative 1-phosphatidylinositol-3-phosphate 5-kinase FAB1D isoform X1 [Dendrobium catenatum]
gene-MA16_Dca026020	uncharacterized protein LOC110094125 [Dendrobium catenatum]
gene-MA16_Dca012526	UPF0481 protein [Dendrobium catenatum]
gene-MA16_Dca022453	G-type lectin S-receptor-like serine/threonine-protein kinase At4g27290 isoform X1 [Dendrobium catenatum]
gene-MA16_Dca020801	pentatricopeptide repeat-containing protein At5g04780-like [Dendrobium catenatum]

gene-MA16_Dca000868	RNA exonuclease 1 [Dendrobium catenatum]
gene-MA16_Dca026137	hypothetical protein MA16_Dca026137 [Dendrobium catenatum]
gene-MA16_Dca010133	uncharacterized protein LOC110099129 isoform X7 [Dendrobium catenatum]
gene-MA16_Dca014260	xylose isomerase isoform X1 [Dendrobium catenatum]
gene-MA16_Dca021652	hypothetical protein MA16_Dca021652 [Dendrobium catenatum]
gene-MA16_Dca025512	hypothetical protein MA16_Dca025512 [Dendrobium catenatum]
gene-MA16_Dca002797	hypothetical protein MA16_Dca002797 [Dendrobium catenatum]
gene-MA16_Dca022493	hypothetical protein MA16_Dca022493 [Dendrobium catenatum]
gene-MA16_Dca001785	hypothetical protein MA16_Dca001785 [Dendrobium catenatum]
gene-MA16_Dca026823	hypothetical protein MA16_Dca026823 [Dendrobium catenatum]
gene-MA16_Dca025486	hypothetical protein MA16_Dca025486 [Dendrobium catenatum]
gene-MA16_Dca012128	hypothetical protein MA16_Dca012128 [Dendrobium catenatum]
gene-MA16_Dca022152	FACT complex subunit SPT16 [Dendrobium catenatum]
gene-MA16_Dca007413	Protein ROOT HAIR DEFECTIVE 3 [Dendrobium catenatum]
gene-MA16_Dca023746	receptor protein kinase-like protein ZAR1 [Dendrobium catenatum]
gene-MA16_Dca012099	ATP-dependent RNA helicase DEAH11, chloroplastic-like [Dendrobium catenatum]
gene-MA16_Dca017781	uncharacterized protein LOC110095018 [Dendrobium catenatum]
gene-MA16_Dca010665	formyltetrahydrofolate deformylase 2, mitochondrial-like isoform X1 [Dendrobium catenatum]
gene-MA16_Dca028626	DNA-directed RNA polymerase II subunit RPB7 [Dendrobium catenatum]
gene-MA16_Dca023590	scarecrow-like protein 15 [Dendrobium catenatum]
gene-MA16_Dca000364	Callose synthase 12 [Dendrobium catenatum]
gene-MA16_Dca013024	CRM-domain containing factor CFM3, chloroplastic/mitochondrial [Dendrobium catenatum]
gene-MA16_Dca018137	probable WRKY transcription factor 3 [Dendrobium catenatum]
gene-MA16_Dca003521	uncharacterized protein LOC110107998 [Dendrobium catenatum]
gene-MA16_Dca015366	hypothetical protein MA16_Dca015366 [Dendrobium catenatum]
gene-MA16_Dca007646	Uncharacterized protein MA16_Dca007646 [Dendrobium catenatum]
gene-MA16_Dca008388	putative beta-1,3-galactosyltransferase 8 [Dendrobium catenatum]
gene-MA16_Dca005251	Putative disease resistance protein RGA3 [Dendrobium catenatum]
gene-MA16_Dca013418	Protein disulfide-isomerase 5-4 [Dendrobium catenatum]
gene-MA16_Dca010207	Chaperone protein dnaJ 15 [Dendrobium catenatum]

gene-MA16_Dca025275	hypothetical protein MA16_Dca025275 [Dendrobium catenatum]
gene-MA16_Dca021697	S-acyltransferase TIP1 [Dendrobium catenatum]
gene-MA16_Dca005680	hypothetical protein MA16_Dca005680 [Dendrobium catenatum]
gene-MA16_Dca008911	Zinc finger protein MAGPIE [Dendrobium catenatum]
gene-MA16_Dca018536	uncharacterized protein LOC110093328 [Dendrobium catenatum]
gene-MA16_Dca004270	Protein ASPARTIC PROTEASE IN GUARD CELL 2 [Dendrobium catenatum]
gene-MA16_Dca002623	CSC1-like protein ERD4 [Dendrobium catenatum]
gene-MA16_Dca016851	Retrovirus-related Pol polyprotein from transposon TNT 1-94 [Dendrobium catenatum]
gene-MA16_Dca011481	uncharacterized protein LOC110112922 [Dendrobium catenatum]
gene-MA16_Dca006703	hypothetical protein MA16_Dca006703 [Dendrobium catenatum]
gene-MA16_Dca011847	pentatricopeptide repeat-containing protein At3g53360, mitochondrial-like isoform X1 [Dendrobium catenatum]
gene-MA16_Dca008436	Putative disease resistance RPP13-like protein 1 [Dendrobium catenatum]
gene-MA16_Dca012388	Putative ribonuclease H protein [Dendrobium catenatum]
gene-MA16_Dca020254	probable histidine kinase 2 [Dendrobium catenatum]
gene-MA16_Dca024943	hypothetical protein MA16_Dca024943 [Dendrobium catenatum]
gene-MA16_Dca015720	receptor protein kinase-like protein ZAR1 [Dendrobium catenatum]
gene-MA16_Dca024748	plant intracellular Ras-group-related LRR protein 4-like isoform X2 [Dendrobium catenatum]
gene-MA16_Dca022241	Beta-hexosaminidase 3 [Dendrobium catenatum]
gene-MA16_Dca024735	hypothetical protein MA16_Dca024735 [Dendrobium catenatum]
gene-MA16_Dca008592	hypothetical protein MA16_Dca008592 [Dendrobium catenatum]
gene-MA16_Dca024638	Fimbrin-like protein 2 [Dendrobium catenatum]
gene-MA16_Dca008310	Chaperonin CPN60-2, mitochondrial [Dendrobium catenatum]
gene-MA16_Dca015855	probable histidine kinase 3 isoform X1 [Dendrobium catenatum]
gene-MA16_Dca020005	hypothetical protein MA16_Dca020005 [Dendrobium catenatum]
gene-MA16_Dca025409	R3H domain-containing protein 1-like [Dendrobium catenatum]
gene-MA16_Dca009747	hypothetical protein MA16_Dca009747 [Dendrobium catenatum]
gene-MA16_Dca017271	uncharacterized protein LOC110094893 [Dendrobium catenatum]
gene-MA16_Dca025851	Eukaryotic translation initiation factor 3 subunit A [Dendrobium catenatum]
gene-MA16_Dca007184	brassinosteroid LRR receptor kinase BRL1-like isoform X1 [Dendrobium catenatum]
gene-MA16_Dca023572	hypothetical protein MA16_Dca023572 [Dendrobium catenatum]

gene-MA16_Dca017865	Levopimaradiene synthase, chloroplastic [Dendrobium catenatum]
gene-MA16_Dca028716	hypothetical protein MA16_Dca028716 [Dendrobium catenatum]
gene-MA16_Dca016675	nodulation-signaling pathway 2 protein-like [Dendrobium catenatum]
gene-MA16_Dca017707	DEAD-box ATP-dependent RNA helicase 39 isoform X1 [Dendrobium catenatum]
gene-MA16_Dca011973	uncharacterized protein LOC110110533 [Dendrobium catenatum]
gene-MA16_Dca015957	Nuclear transcription factor Y subunit B-8 [Dendrobium catenatum]
gene-MA16_Dca007852	zinc finger CCHC domain-containing protein 10 [Dendrobium catenatum]
gene-MA16_Dca024737	Growth-regulating factor 6 [Dendrobium catenatum]
gene-MA16_Dca025553	Hypersensitive-induced response protein 4 [Dendrobium catenatum]
gene-MA16_Dca022074	trafficking protein particle complex subunit 8 isoform X1 [Dendrobium catenatum]
gene-MA16_Dca024483	pentatricopeptide repeat-containing protein At1g11290, chloroplastic-like [Dendrobium catenatum]
gene-MA16_Dca019565	hypothetical protein MA16_Dca019565 [Dendrobium catenatum]
gene-MA16_Dca002672	cytochrome P450 86B1-like [Dendrobium catenatum]
gene-MA16_Dca016809	probable beta-D-xylosidase 2 [Dendrobium catenatum]
gene-MA16_Dca018071	calcium/calmodulin-regulated receptor-like kinase 2 [Dendrobium catenatum]
gene-MA16_Dca018603	hypothetical protein MA16_Dca018603 [Dendrobium catenatum]
gene-MA16_Dca011298	uncharacterized protein LOC110101678 [Dendrobium catenatum]
gene-MA16_Dca012467	Methyl-CpG-binding domain-containing protein 1 [Dendrobium catenatum]
gene-MA16_Dca010017	hypothetical protein MA16_Dca010017 [Dendrobium catenatum]
gene-MA16_Dca002538	probable transcription factor KAN2 [Dendrobium catenatum]
gene-MA16_Dca006088	hypothetical protein MA16_Dca006088 [Dendrobium catenatum]
gene-MA16_Dca011730	hypothetical protein MA16_Dca011730 [Dendrobium catenatum]
gene-MA16_Dca019933	Retrovirus-related Pol polyprotein from transposon TNT 1-94 [Dendrobium catenatum]
gene-MA16_Dca005330	ENHANCER OF AG-4 protein 2-like isoform X1 [Dendrobium catenatum]
gene-MA16_Dca020124	DSC E3 ubiquitin ligase complex subunit 1-like [Dendrobium catenatum]
gene-MA16_Dca026376	Cytochrome P450 71A1 [Dendrobium catenatum]
gene-MA16_Dca011443	L-type lectin-domain containing receptor kinase S.4-like [Dendrobium catenatum]
gene-MA16_Dca022358	hypothetical protein MA16_Dca022358 [Dendrobium catenatum]
gene-MA16_Dca004692	hypothetical protein MA16_Dca004692 [Dendrobium catenatum]
gene-MA16_Dca021285	probable protein S-acyltransferase 23 isoform X1 [Dendrobium catenatum]

gene-MA16_Dca017220	transmembrane protein 87A [Dendrobium catenatum]
gene-MA16_Dca023911	putative SNAP25 homologous protein SNAP30 isoform X1 [Dendrobium catenatum]
gene-MA16_Dca001810	putative serine/threonine-protein kinase [Dendrobium catenatum]
gene-MA16_Dca003804	cytochrome P450 71A1-like [Dendrobium catenatum]
gene-MA16_Dca013973	Retrovirus-related Pol polyprotein from transposon TNT 1-94 [Dendrobium catenatum]
gene-MA16_Dca016800	Regulator of nonsense transcripts UPF2 [Dendrobium catenatum]
gene-MA16_Dca024521	hypothetical protein MA16_Dca024521 [Dendrobium catenatum]
gene-MA16_Dca010335	RNA polymerase II C-terminal domain phosphatase-like 1 [Dendrobium catenatum]
gene-MA16_Dca015480	helicase-like transcription factor CHR28 [Dendrobium catenatum]
gene-MA16_Dca001333	uncharacterized protein LOC110100801 [Dendrobium catenatum]
gene-MA16_Dca016068	succinate dehydrogenase subunit 7, mitochondrial-like [Dendrobium catenatum]
gene-MA16_Dca008239	hypothetical protein MA16_Dca008239 [Dendrobium catenatum]
gene-MA16_Dca028785	hypothetical protein MA16_Dca028785 [Dendrobium catenatum]
gene-MA16_Dca000164	Aspartic proteinase nepenthesin-1 [Dendrobium catenatum]
gene-MA16_Dca013354	hypothetical protein MA16_Dca013354 [Dendrobium catenatum]
gene-MA16_Dca026671	hypothetical protein MA16_Dca026671 [Dendrobium catenatum]
gene-MA16_Dca008180	hypothetical protein MA16_Dca008180 [Dendrobium catenatum]
gene-MA16_Dca000084	putative disease resistance protein RGA3 [Dendrobium catenatum]
gene-MA16_Dca004101	WAT1-related protein At4g08290-like [Dendrobium catenatum]
gene-MA16_Dca026213	hypothetical protein MA16_Dca026213 [Dendrobium catenatum]
gene-MA16_Dca013892	hypothetical protein MA16_Dca013892 [Dendrobium catenatum]
gene-MA16_Dca009052	E3 ubiquitin-protein ligase ATL31-like [Dendrobium catenatum]
gene-MA16_Dca007591	Subtilisin-like protease SDD1 [Dendrobium catenatum]
gene-MA16_Dca020360	Mitogen-activated protein kinase kinase kinase YODA [Dendrobium catenatum]
gene-MA16_Dca013806	transcription initiation factor TFIID subunit 12b-like isoform X1 [Dendrobium catenatum]
gene-MA16_Dca019557	AT-rich interactive domain-containing protein 4-like isoform X1 [Dendrobium catenatum]
gene-MA16_Dca001553	putative disease resistance protein RGA1 isoform X1 [Dendrobium catenatum]
gene-MA16_Dca021620	uncharacterized protein LOC110113015 [Dendrobium catenatum]
gene-MA16_Dca000485	Squamosa promoter-binding-like protein 18 [Dendrobium catenatum]
gene-MA16_Dca007632	Serine/threonine-protein kinase [Dendrobium catenatum]

gene-MA16_Dca003224	hypothetical protein MA16_Dca003224 [Dendrobium catenatum]
gene-MA16_Dca022048	Retrovirus-related Pol polyprotein from transposon TNT 1-94 [Dendrobium catenatum]
gene-MA16_Dca022148	DEAD-box ATP-dependent RNA helicase 46-like [Dendrobium catenatum]
gene-MA16_Dca002932	putative Myb family transcription factor At1g14600 isoform X2 [Dendrobium catenatum]
gene-MA16_Dca010269	transforming growth factor-beta receptor-associated protein 1 [Dendrobium catenatum]
gene-MA16_Dca021955	hypothetical protein MA16_Dca021955 [Dendrobium catenatum]
gene-MA16_Dca019292	E1A-binding protein p400 [Dendrobium catenatum]
gene-MA16_Dca006313	Thioredoxin-like 4, chloroplastic [Dendrobium catenatum]
gene-MA16_Dca027425	Tropinone reductase 1 [Dendrobium catenatum]
gene-MA16_Dca025751	inactive LRR receptor-like serine/threonine-protein kinase BIR2 [Dendrobium catenatum]
gene-MA16_Dca015690	uncharacterized protein LOC110116180 [Dendrobium catenatum]
gene-MA16_Dca008901	3-oxoacyl-[acyl-carrier-protein] synthase I, chloroplastic [Dendrobium catenatum]
gene-MA16_Dca024649	hypothetical protein MA16_Dca024649 [Dendrobium catenatum]
gene-MA16_Dca017391	Retrovirus-related Pol polyprotein from transposon TNT 1-94 [Dendrobium catenatum]
gene-MA16_Dca019418	uncharacterized protein LOC110110300 isoform X1 [Dendrobium catenatum]
gene-MA16_Dca016539	uncharacterized protein LOC110113536 [Dendrobium catenatum]
gene-MA16_Dca001321	Retrovirus-related Pol polyprotein from transposon TNT 1-94 [Dendrobium catenatum]
gene-MA16_Dca004131	hypothetical protein MA16_Dca004131 [Dendrobium catenatum]
gene-MA16_Dca002118	Retrovirus-related Pol polyprotein from transposon TNT 1-94 [Dendrobium catenatum]
gene-MA16_Dca013906	Cycloeucalenol cycloisomerase [Dendrobium catenatum]
gene-MA16_Dca000995	Pentatricopeptide repeat-containing protein [Dendrobium catenatum]
gene-MA16_Dca013327	FT-interacting protein 1-like [Dendrobium catenatum]
gene-MA16_Dca007694	GATA transcription factor 26-like isoform X1 [Dendrobium catenatum]
gene-MA16_Dca027066	Retrovirus-related Pol polyprotein from transposon TNT 1-94 [Dendrobium catenatum]
gene-MA16_Dca027145	subtilisin-like protease SBT1.7 [Dendrobium catenatum]
gene-MA16_Dca024531	hypothetical protein MA16_Dca024531 [Dendrobium catenatum]
gene-MA16_Dca027076	hypothetical protein MA16_Dca027076 [Dendrobium catenatum]
gene-MA16_Dca000143	poly [ADP-ribose] polymerase 2-A [Dendrobium catenatum]
gene-MA16_Dca017476	putative flavin-containing monooxygenase 1 [Dendrobium catenatum]
gene-MA16_Dca014467	uncharacterized protein LOC110111620 [Dendrobium catenatum]

gene-MA16_Dca003559	uncharacterized protein LOC110110389 [Dendrobium catenatum]
gene-MA16_Dca028340	Cellulose synthase A catalytic subunit 9 [UDP-forming] [Dendrobium catenatum]
gene-MA16_Dca009669	uncharacterized protein LOC110115650 [Dendrobium catenatum]
gene-MA16_Dca007063	probable plastid-lipid-associated protein 7, chloroplastic isoform X1 [Dendrobium catenatum]
gene-MA16_Dca027976	hypothetical protein MA16_Dca027976 [Dendrobium catenatum]
gene-MA16_Dca028824	hypothetical protein MA16_Dca028824 [Dendrobium catenatum]
gene-MA16_Dca027301	G-type lectin S-receptor-like serine/threonine-protein kinase B120 isoform X2 [Dendrobium catenatum]
gene-MA16_Dca025989	tRNA(adenine(34)) deaminase, chloroplastic isoform X1 [Dendrobium catenatum]
gene-MA16_Dca004159	NAC domain-containing protein 8 [Dendrobium catenatum]
gene-MA16_Dca005928	Gibberellin 2-beta-dioxygenase 8 [Dendrobium catenatum]
gene-MA16_Dca020953	Uncharacterized protein MA16_Dca020953 [Dendrobium catenatum]
gene-MA16_Dca020178	hypothetical protein MA16_Dca020178 [Dendrobium catenatum]
gene-MA16_Dca010911	uncharacterized protein LOC110116323 [Dendrobium catenatum]
gene-MA16_Dca020219	60S ribosomal protein L8 [Dendrobium catenatum]
gene-MA16_Dca000783	hypothetical protein MA16_Dca000783 [Dendrobium catenatum]
gene-MA16_Dca023864	hypothetical protein MA16_Dca023864 [Dendrobium catenatum]
gene-MA16_Dca023565	ACT domain-containing protein ACR2 [Dendrobium catenatum]
gene-MA16_Dca006055	hypothetical protein MA16_Dca006055 [Dendrobium catenatum]
gene-MA16_Dca005016	hypothetical protein MA16_Dca005016 [Dendrobium catenatum]
gene-MA16_Dca019159	acetyl-CoA carboxylase 1-like [Dendrobium catenatum]
gene-MA16_Dca011435	Retrovirus-related Pol polyprotein from transposon TNT 1-94 [Dendrobium catenatum]
gene-MA16_Dca023526	hypothetical protein MA16_Dca023526 [Dendrobium catenatum]
gene-MA16_Dca015250	uncharacterized protein LOC110103465 isoform X1 [Dendrobium catenatum]
gene-MA16_Dca006017	hypothetical protein MA16_Dca006017 [Dendrobium catenatum]
gene-MA16_Dca010464	putative methyltransferase PMT2 [Dendrobium catenatum]
gene-MA16_Dca015874	hypothetical protein MA16_Dca015874 [Dendrobium catenatum]
gene-MA16_Dca009114	E3 ubiquitin-protein ligase BRE1-like 1 [Dendrobium catenatum]
gene-MA16_Dca022690	uncharacterized membrane protein At1g16860-like [Dendrobium catenatum]
gene-MA16_Dca011721	hypothetical protein MA16_Dca011721 [Dendrobium catenatum]
gene-MA16_Dca004010	probable serine/threonine-protein kinase PBL5 [Dendrobium catenatum]

gene-MA16_Dca013452	sialyltransferase-like protein 4 [Dendrobium catenatum]
gene-MA16_Dca011463	hypothetical protein MA16_Dca011463 [Dendrobium catenatum]
gene-MA16_Dca028102	hypothetical protein MA16_Dca028102 [Dendrobium catenatum]
gene-MA16_Dca004575	Pentatricopeptide repeat-containing protein [Dendrobium catenatum]
gene-MA16_Dca018279	uracil phosphoribosyltransferase isoform X2 [Dendrobium catenatum]
gene-MA16_Dca015735	Serine/threonine-protein kinase [Dendrobium catenatum]
gene-MA16_Dca012642	hypothetical protein MA16_Dca012642 [Dendrobium catenatum]
gene-MA16_Dca018094	putative disease resistance protein RGA3 [Dendrobium catenatum]
gene-MA16_Dca003339	uncharacterized protein LOC110099020 [Dendrobium catenatum]
gene-MA16_Dca023179	NAC domain-containing protein 21/22-like [Dendrobium catenatum]
gene-MA16_Dca013716	CTP synthase 1-like [Dendrobium catenatum]
gene-MA16_Dca000903	ATP-dependent DNA helicase At3g02060, chloroplastic isoform X1 [Dendrobium catenatum]
gene-MA16_Dca011371	pentatricopeptide repeat-containing protein At2g22410, mitochondrial-like [Dendrobium catenatum]
gene-MA16_Dca011962	subtilisin-like protease SBT1.4 [Dendrobium catenatum]
gene-MA16_Dca014129	P-loop NTPase domain-containing protein LPA1-like isoform X1 [Dendrobium catenatum]
gene-MA16_Dca026698	phospholipase A1-Ibeta2, chloroplastic-like [Dendrobium catenatum]
gene-MA16_Dca020338	Zinc finger protein CONSTANS-LIKE 5 [Dendrobium catenatum]
gene-MA16_Dca001587	ABC transporter B family member 1 [Dendrobium catenatum]
gene-MA16_Dca022156	Subtilisin-like protease SDD1 [Dendrobium catenatum]
gene-MA16_Dca013574	hypothetical protein MA16_Dca013574 [Dendrobium catenatum]
gene-MA16_Dca022508	phosphatidylserine decarboxylase proenzyme 2-like [Dendrobium catenatum]
gene-MA16_Dca026108	Uridine nucleosidase 1 [Dendrobium catenatum]
gene-MA16_Dca024903	Putative disease resistance protein RGA4 [Dendrobium catenatum]
gene-MA16_Dca003869	RPM1-interacting protein 4-like isoform X3 [Dendrobium catenatum]
gene-MA16_Dca010385	syntaxin-41-like [Dendrobium catenatum]
gene-MA16_Dca028429	Retrovirus-related Pol polyprotein from transposon TNT 1-94 [Dendrobium catenatum]
gene-MA16_Dca012031	TBC1 domain family member 15-like isoform X1 [Dendrobium catenatum]
gene-MA16_Dca001199	putative disease resistance protein RGA3 [Dendrobium catenatum]
gene-MA16_Dca020965	hypothetical protein MA16_Dca020965 [Dendrobium catenatum]
gene-MA16_Dca028021	DNA repair endonuclease UVH1-like [Dendrobium catenatum]

gene-MA16_Dca015466	B3 domain-containing protein [Dendrobium catenatum]
gene-MA16_Dca005551	tetratricopeptide repeat protein SKI3 [Dendrobium catenatum]
gene-MA16_Dca003709	NAC transcription factor ONAC010 [Dendrobium catenatum]
gene-MA16_Dca022001	fasciclin-like arabinogalactan protein 19 [Dendrobium catenatum]
gene-MA16_Dca000126	HSP-interacting protein [Dendrobium catenatum]
gene-MA16_Dca015361	ATPase family AAA domain-containing protein 3-like [Dendrobium catenatum]
gene-MA16_Dca009985	nuclear inhibitor of protein phosphatase 1 [Dendrobium catenatum]
gene-MA16_Dca015478	Retrovirus-related Pol polyprotein from transposon TNT 1-94 [Dendrobium catenatum]
gene-MA16_Dca020025	Putative ribonuclease H protein [Dendrobium catenatum]
gene-MA16_Dca006481	protein BZR1 homolog 1-like [Dendrobium catenatum]
gene-MA16_Dca018722	hypothetical protein MA16_Dca018722 [Dendrobium catenatum]
gene-MA16_Dca010789	ATP-dependent zinc metalloprotease FTSH 6, chloroplastic [Dendrobium catenatum]
gene-MA16_Dca009384	laccase-24-like [Dendrobium catenatum]
gene-MA16_Dca008739	Myb-related protein 3R-1 [Dendrobium catenatum]
gene-MA16_Dca002930	hypothetical protein MA16_Dca002930 [Dendrobium catenatum]
gene-MA16_Dca017666	Putative ribonuclease H protein [Dendrobium catenatum]
gene-MA16_Dca002554	ADP-ribosylation factor 1 [Dendrobium catenatum]
gene-MA16_Dca009542	hypothetical protein MA16_Dca009542 [Dendrobium catenatum]
gene-MA16_Dca011134	Retrovirus-related Pol polyprotein from transposon TNT 1-94 [Dendrobium catenatum]
gene-MA16_Dca027179	Retrovirus-related Pol polyprotein from transposon TNT 1-94 [Dendrobium catenatum]
gene-MA16_Dca024562	uncharacterized protein LOC110100796 [Dendrobium catenatum]
gene-MA16_Dca000422	UDP-glucuronate 4-epimerase 4-like [Dendrobium catenatum]
gene-MA16_Dca014491	uncharacterized protein LOC110093029 [Dendrobium catenatum]
gene-MA16_Dca003801	F-box protein [Dendrobium catenatum]
gene-MA16_Dca025424	disease resistance protein RGA2-like [Dendrobium catenatum]
gene-MA16_Dca009550	40S ribosomal protein S5 [Dendrobium catenatum]
gene-MA16_Dca002617	F-box protein [Dendrobium catenatum]
gene-MA16_Dca026176	hypothetical protein MA16_Dca026176 [Dendrobium catenatum]
gene-MA16_Dca017426	serotonin N-acetyltransferase 1, chloroplastic [Dendrobium catenatum]
gene-MA16_Dca028206	Retrovirus-related Pol polyprotein from transposon TNT 1-94 [Dendrobium catenatum]

gene-MA16_Dca008826	uncharacterized protein At1g27050 [Dendrobium catenatum]
gene-MA16_Dca014761	protein ECERIFERUM 26-like [Dendrobium catenatum]
gene-MA16_Dca015597	U-box domain-containing protein 20-like [Dendrobium catenatum]
gene-MA16_Dca007565	histone H4 [Zea mays]
gene-MA16_Dca000657	hypothetical protein MA16_Dca000657 [Dendrobium catenatum]
gene-MA16_Dca015846	branchpoint-bridging protein-like [Dendrobium catenatum]
gene-MA16_Dca005971	Leucine-rich repeat extensin-like protein 4 [Dendrobium catenatum]
gene-MA16_Dca017670	zinc finger A20 and AN1 domain-containing stress-associated protein 5-like [Dendrobium catenatum]
gene-MA16_Dca009232	uncharacterized protein LOC110115434 [Dendrobium catenatum]
gene-MA16_Dca000774	Disease resistance protein RGA2 [Dendrobium catenatum]
gene-MA16_Dca015912	putative pentatricopeptide repeat-containing protein At3g08820 [Dendrobium catenatum]
gene-MA16_Dca003270	zinc finger protein ZAT2-like [Dendrobium catenatum]
gene-MA16_Dca001748	hypothetical protein MA16_Dca001748 [Dendrobium catenatum]
gene-MA16_Dca002478	tetraspanin-3-like [Dendrobium catenatum]
gene-MA16_Dca026886	hypothetical protein MA16_Dca026886 [Dendrobium catenatum]
gene-MA16_Dca026328	Retrovirus-related Pol polyprotein from transposon TNT 1-94 [Dendrobium catenatum]
gene-MA16_Dca005595	ubiquitin-conjugating enzyme E2 4-like [Dendrobium catenatum]
gene-MA16_Dca023885	FT-interacting protein 1-like [Dendrobium catenatum]
gene-MA16_Dca022078	5-methyltetrahydropteroyltriglutamate--homocysteine methyltransferase [Dendrobium catenatum]
gene-MA16_Dca011533	hypothetical protein MA16_Dca011533 [Dendrobium catenatum]
gene-MA16_Dca013886	mitogen-activated protein kinase 10-like isoform X1 [Dendrobium catenatum]
gene-MA16_Dca022291	Serine/threonine-protein kinase GRIK1 [Dendrobium catenatum]
gene-MA16_Dca026801	Retrovirus-related Pol polyprotein from transposon TNT 1-94 [Dendrobium catenatum]
gene-MA16_Dca007917	CRM-domain containing factor CFM3, chloroplastic/mitochondrial isoform X1 [Dendrobium catenatum]
gene-MA16_Dca023237	hypothetical protein MA16_Dca023237 [Dendrobium catenatum]
gene-MA16_Dca011099	Cytochrome P450 71A9 [Dendrobium catenatum]
gene-MA16_Dca017831	Formate dehydrogenase, mitochondrial [Dendrobium catenatum]
gene-MA16_Dca008922	Premnaspirodiene oxygenase [Dendrobium catenatum]
gene-MA16_Dca014578	pentatricopeptide repeat-containing protein At3g54980, mitochondrial [Dendrobium catenatum]
gene-MA16_Dca011577	Mannan endo-1,4-beta-mannosidase 1 [Dendrobium catenatum]

gene-MA16_Dca026028	hypothetical protein MA16_Dca026028 [Dendrobium catenatum]
gene-MA16_Dca009628	hypothetical protein MA16_Dca009628 [Dendrobium catenatum]
gene-MA16_Dca007067	uncharacterized protein LOC110102921 [Dendrobium catenatum]
gene-MA16_Dca011106	hypothetical protein MA16_Dca011106 [Dendrobium catenatum]
gene-MA16_Dca026290	uncharacterized protein LOC110098520 [Dendrobium catenatum]
gene-MA16_Dca019228	uncharacterized protein LOC110095413 [Dendrobium catenatum]
gene-MA16_Dca008856	NAC domain-containing protein 10-like [Dendrobium catenatum]
gene-MA16_Dca027531	putative disease resistance protein RGA4 [Dendrobium catenatum]
gene-MA16_Dca005992	uncharacterized protein LOC110110891 [Dendrobium catenatum]
gene-MA16_Dca004888	putative TPR repeat-containing protein [Dendrobium catenatum]
gene-MA16_Dca000754	hypothetical protein MA16_Dca000754 [Dendrobium catenatum]
gene-MA16_Dca013351	putative 4-hydroxy-4-methyl-2-oxoglutarate aldolase 3 [Dendrobium catenatum]
gene-MA16_Dca021343	DNA (cytosine-5)-methyltransferase DRM2 [Dendrobium catenatum]
gene-MA16_Dca002514	hypothetical protein MA16_Dca002514 [Dendrobium catenatum]
gene-MA16_Dca013669	hypothetical protein MA16_Dca013669 [Dendrobium catenatum]
gene-MA16_Dca011586	RNA-directed DNA polymerase [Dendrobium catenatum]
gene-MA16_Dca012922	Putative ribonuclease H protein [Dendrobium catenatum]
gene-MA16_Dca009136	Retrovirus-related Pol polyprotein from transposon TNT 1-94 [Dendrobium catenatum]
gene-MA16_Dca028404	Putative ribonuclease H protein [Dendrobium catenatum]
gene-MA16_Dca017330	NADH dehydrogenase [ubiquinone] 1 alpha subcomplex assembly factor 3-like isoform X1 [Dendrobium catenatum]
gene-MA16_Dca020826	3-deoxy-manno-octulosonate cytidylyltransferase, mitochondrial isoform X1 [Dendrobium catenatum]
gene-MA16_Dca025550	cation/H(+) antiporter 15-like [Dendrobium catenatum]
gene-MA16_Dca018756	oligopeptide transporter 3 [Dendrobium catenatum]
gene-MA16_Dca007809	hippocampus abundant transcript 1 protein-like [Dendrobium catenatum]
gene-MA16_Dca020963	protein SUPPRESSOR OF PHYA-105 1-like [Dendrobium catenatum]
gene-MA16_Dca001628	hypothetical protein MA16_Dca001628 [Dendrobium catenatum]
gene-MA16_Dca010516	hypothetical protein MA16_Dca010516 [Dendrobium catenatum]
gene-MA16_Dca017160	WD repeat-containing protein DDB_G0290555 [Dendrobium catenatum]
gene-MA16_Dca019818	lactase-phlorizin hydrolase-like [Dendrobium catenatum]
gene-MA16_Dca016374	uncharacterized protein LOC110110994 [Dendrobium catenatum]

gene-MA16_Dca021278	Eukaryotic translation initiation factor isoform 4G-1 [Dendrobium catenatum]
gene-MA16_Dca013555	serine/arginine-rich splicing factor 4-like [Dendrobium catenatum]
gene-MA16_Dca010804	cleft lip and palate transmembrane protein 1 homolog [Dendrobium catenatum]
gene-MA16_Dca023524	hypothetical protein MA16_Dca023524 [Dendrobium catenatum]
gene-MA16_Dca014675	hypothetical protein MA16_Dca014675 [Dendrobium catenatum]
gene-MA16_Dca012972	hypothetical protein MA16_Dca012972 [Dendrobium catenatum]
gene-MA16_Dca021318	NAC domain-containing protein 21/22-like [Dendrobium catenatum]
gene-MA16_Dca028098	DNA polymerase [Dendrobium catenatum]
gene-MA16_Dca008352	hypothetical protein MA16_Dca008352 [Dendrobium catenatum]
gene-MA16_Dca014442	crocetin glucosyltransferase 3 [Dendrobium catenatum]
gene-MA16_Dca022853	hypothetical protein MA16_Dca022853 [Dendrobium catenatum]
gene-MA16_Dca027107	uncharacterized protein LOC110109377 [Dendrobium catenatum]
gene-MA16_Dca012324	bibenzyl synthase-like [Dendrobium catenatum]
gene-MA16_Dca025629	palmitoyl-CoA hydrolase [Dendrobium catenatum]
gene-MA16_Dca008594	putative LRR receptor-like serine/threonine-protein kinase [Dendrobium catenatum]
gene-MA16_Dca026076	hypothetical protein MA16_Dca026076 [Dendrobium catenatum]
gene-MA16_Dca006101	hypothetical protein MA16_Dca006101 [Dendrobium catenatum]
gene-MA16_Dca021079	ATP synthase subunit beta, mitochondrial-like [Dendrobium catenatum]
gene-MA16_Dca020263	hypothetical protein MA16_Dca020263 [Dendrobium catenatum]
gene-MA16_Dca011603	probable ATP-dependent DNA helicase CHR12 isoform X3 [Dendrobium catenatum]
gene-MA16_Dca026927	pyruvate dehydrogenase E1 component subunit alpha-3, chloroplastic [Dendrobium catenatum]
gene-MA16_Dca015264	leucine-rich repeat receptor-like serine/threonine/tyrosine-protein kinase SOBIR1 [Dendrobium catenatum]
gene-MA16_Dca021433	Serine/threonine-protein kinase [Dendrobium catenatum]
gene-MA16_Dca000289	hypothetical protein MA16_Dca019111 [Dendrobium catenatum]
gene-MA16_Dca016512	Ras-related protein RABC2a [Dendrobium catenatum]
gene-MA16_Dca021824	phospholipase A(1) LCAT3 [Dendrobium catenatum]
gene-MA16_Dca002026	putative mitochondrial protein [Dendrobium catenatum]
gene-MA16_Dca009198	hypothetical protein MA16_Dca009198 [Dendrobium catenatum]
gene-MA16_Dca021780	putative mitochondrial protein [Dendrobium catenatum]
gene-MA16_Dca018789	hypothetical protein MA16_Dca018789 [Dendrobium catenatum]

gene-MA16_Dca019854	aquaporin PIP1-2-like [Dendrobium catenatum]
gene-MA16_Dca020850	Protein ASPARTIC PROTEASE IN GUARD CELL 2 [Dendrobium catenatum]
gene-MA16_Dca024067	Zinc finger CCCH domain-containing protein 4 [Dendrobium catenatum]
gene-MA16_Dca027323	hypothetical protein MA16_Dca027323 [Dendrobium catenatum]
gene-MA16_Dca023808	Nucleobase-ascorbate transporter 6 [Dendrobium catenatum]
gene-MA16_Dca011854	ABC transporter C family member 8 [Dendrobium catenatum]
gene-MA16_Dca016727	hypothetical protein MA16_Dca016727 [Dendrobium catenatum]
gene-MA16_Dca014102	hypothetical protein MA16_Dca014102 [Dendrobium catenatum]
gene-MA16_Dca021909	putative peptide/nitrate transporter [Dendrobium catenatum]
gene-MA16_Dca010182	G-type lectin S-receptor-like serine/threonine-protein kinase RLK1 [Dendrobium catenatum]
gene-MA16_Dca027492	hypothetical protein MA16_Dca027492 [Dendrobium catenatum]
gene-MA16_Dca020619	Transcription factor HBP-1b(c1) [Dendrobium catenatum]
gene-MA16_Dca018098	Putative disease resistance protein RGA3 [Dendrobium catenatum]
gene-MA16_Dca000173	uncharacterized protein LOC110092196 [Dendrobium catenatum]
gene-MA16_Dca005690	uncharacterized protein LOC110092794 [Dendrobium catenatum]
gene-MA16_Dca016458	protein NRT1/ PTR FAMILY 5.10-like isoform X1 [Dendrobium catenatum]
gene-MA16_Dca008000	integrator complex subunit 11 [Dendrobium catenatum]
gene-MA16_Dca019207	uncharacterized protein LOC110093900 [Dendrobium catenatum]
gene-MA16_Dca016959	hypothetical protein MA16_Dca016959 [Dendrobium catenatum]
gene-MA16_Dca028385	Chorismate mutase 2 [Dendrobium catenatum]
gene-MA16_Dca003630	aldehyde dehydrogenase 22A1 [Dendrobium catenatum]
gene-MA16_Dca026399	transcription factor EMB1444-like [Dendrobium catenatum]
gene-MA16_Dca011151	Retrovirus-related Pol polyprotein from transposon TNT 1-94 [Dendrobium catenatum]
gene-MA16_Dca012706	trihelix transcription factor GTL1-like [Dendrobium catenatum]
gene-MA16_Dca017554	uncharacterized protein LOC110109560 isoform X11 [Dendrobium catenatum]
gene-MA16_Dca013981	type I inositol polyphosphate 5-phosphatase 8-like isoform X1 [Dendrobium catenatum]
gene-MA16_Dca000756	vesicle transport protein GOT1-like [Dendrobium catenatum]
gene-MA16_Dca025044	serine/threonine-protein kinase ATG1t isoform X2 [Dendrobium catenatum]
gene-MA16_Dca018345	eukaryotic translation initiation factor 2 subunit gamma-like [Dendrobium catenatum]
gene-MA16_Dca023122	Cytochrome P450 89A2 [Dendrobium catenatum]

gene-MA16_Dca003118	protein SUPPRESSOR OF K(+) TRANSPORT GROWTH DEFECT 1-like [Dendrobium catenatum]
gene-MA16_Dca008215	hypothetical protein MA16_Dca008215 [Dendrobium catenatum]
gene-MA16_Dca010241	uncharacterized protein LOC110116537 [Dendrobium catenatum]
gene-MA16_Dca023641	Retrovirus-related Pol polyprotein from transposon TNT 1-94 [Dendrobium catenatum]
gene-MA16_Dca019173	RNA-directed DNA polymerase [Dendrobium catenatum]
gene-MA16_Dca015141	hypothetical protein MA16_Dca015141 [Dendrobium catenatum]
gene-MA16_Dca004149	NAC transcription factor 25 [Dendrobium catenatum]
gene-MA16_Dca003342	hypothetical protein MA16_Dca003342 [Dendrobium catenatum]
gene-MA16_Dca026065	hypothetical protein MA16_Dca026065 [Dendrobium catenatum]
gene-MA16_Dca020713	hypothetical protein MA16_Dca020713 [Dendrobium catenatum]
gene-MA16_Dca026079	cytochrome P450 CYP749A22-like [Dendrobium catenatum]
gene-MA16_Dca020299	expansin-like B1 [Dendrobium catenatum]
gene-MA16_Dca002309	Embryogenesis-associated protein EMB8 [Dendrobium catenatum]
gene-MA16_Dca014625	Retrovirus-related Pol polyprotein from transposon TNT 1-94 [Dendrobium catenatum]
gene-MA16_Dca004728	Auxin response factor 18 [Dendrobium catenatum]
gene-MA16_Dca028513	RNA-directed DNA polymerase [Dendrobium catenatum]
gene-MA16_Dca025324	probable transcription factor KAN4 [Dendrobium catenatum]
gene-MA16_Dca006136	uncharacterized protein LOC110110740 isoform X1 [Dendrobium catenatum]
gene-MA16_Dca015903	Regulatory-associated protein of TOR 1 [Dendrobium catenatum]
gene-MA16_Dca011239	transcription factor SAC51-like [Dendrobium catenatum]
gene-MA16_Dca024189	hypothetical protein MA16_Dca024189 [Dendrobium catenatum]
gene-MA16_Dca014398	ELL-associated factor 2 [Dendrobium catenatum]
gene-MA16_Dca015839	dynamin-related protein 3A-like isoform X2 [Dendrobium catenatum]
gene-MA16_Dca021981	U-box domain-containing protein 27-like [Dendrobium catenatum]
gene-MA16_Dca004287	hypothetical protein MA16_Dca004287 [Dendrobium catenatum]
gene-MA16_Dca027962	Retrovirus-related Pol polyprotein from transposon TNT 1-94 [Dendrobium catenatum]
gene-MA16_Dca021406	hypothetical protein MA16_Dca021406 [Dendrobium catenatum]
gene-MA16_Dca000247	uncharacterized protein LOC110114931 [Dendrobium catenatum]
gene-MA16_Dca000505	uncharacterized membrane protein At1g06890-like [Dendrobium catenatum]
gene-MA16_Dca003450	hypothetical protein MA16_Dca003450 [Dendrobium catenatum]

gene-MA16_Dca022543	Uncharacterized protein MA16_Dca022543 [Dendrobium catenatum]
gene-MA16_Dca021083	PI-PLC X domain-containing protein At5g67130 isoform X4 [Dendrobium catenatum]
gene-MA16_Dca009655	isoflavone 2'-hydroxylase-like [Dendrobium catenatum]
gene-MA16_Dca025534	mannose-specific lectin-like [Dendrobium catenatum]
gene-MA16_Dca015763	hypothetical protein MA16_Dca015763 [Dendrobium catenatum]
gene-MA16_Dca000279	putative disease resistance protein RGA3 [Dendrobium catenatum]
gene-MA16_Dca024417	beta-glucosidase 12-like [Dendrobium catenatum]
gene-MA16_Dca024403	putative protein phosphatase 2C 27 [Dendrobium catenatum]
gene-MA16_Dca012290	Zinc finger CCCH domain-containing protein 55 [Dendrobium catenatum]
gene-MA16_Dca025262	CBL-interacting protein kinase 2 [Dendrobium catenatum]
gene-MA16_Dca026084	uncharacterized protein LOC110103293 [Dendrobium catenatum]
gene-MA16_Dca026762	hypothetical protein MA16_Dca026762 [Dendrobium catenatum]
gene-MA16_Dca022455	Receptor-like serine/threonine-protein kinase SD1-8 [Dendrobium catenatum]
gene-MA16_Dca025504	acyl-coenzyme A oxidase, peroxisomal [Dendrobium catenatum]
gene-MA16_Dca006366	hypothetical protein MA16_Dca006366 [Dendrobium catenatum]
gene-MA16_Dca015429	transcription factor PIF3-like [Dendrobium catenatum]
gene-MA16_Dca019648	Chaperone protein dnaJ 49 [Dendrobium catenatum]
gene-MA16_Dca015273	myosin-2 isoform X1 [Dendrobium catenatum]
gene-MA16_Dca012189	uncharacterized protein LOC110109190 [Dendrobium catenatum]
gene-MA16_Dca012725	RING-H2 finger protein ATL39-like [Dendrobium catenatum]
gene-MA16_Dca001153	mechanosensitive ion channel protein 10-like [Dendrobium catenatum]
gene-MA16_Dca020583	WEB family protein At5g16730, chloroplastic-like [Dendrobium catenatum]
gene-MA16_Dca013650	putative polyamine oxidase 2 [Dendrobium catenatum]
gene-MA16_Dca024732	Ammonium transporter 3 member 1 [Dendrobium catenatum]
gene-MA16_Dca005100	zinc-finger homeodomain protein 5-like [Dendrobium catenatum]
gene-MA16_Dca010954	hypothetical protein MA16_Dca010954 [Dendrobium catenatum]
gene-MA16_Dca025150	uncharacterized protein LOC110114729 [Dendrobium catenatum]
gene-MA16_Dca006906	cation/calcium exchanger 1-like [Dendrobium catenatum]
gene-MA16_Dca013585	hypothetical protein MA16_Dca013585 [Dendrobium catenatum]
gene-MA16_Dca010820	protein FAR1-RELATED SEQUENCE 5-like [Dendrobium catenatum]

gene-MA16_Dca005319	SCAR-like protein 2 [Dendrobium catenatum]
gene-MA16_Dca005372	Transcription factor PIF4 [Dendrobium catenatum]
gene-MA16_Dca005002	Patellin-3 [Dendrobium catenatum]
gene-MA16_Dca002342	Carbonic anhydrase, chloroplastic [Dendrobium catenatum]
gene-MA16_Dca002241	putative helicase MAGATAMA 3 [Dendrobium catenatum]
gene-MA16_Dca016785	hypothetical protein MA16_Dca016785 [Dendrobium catenatum]
gene-MA16_Dca012052	hypothetical protein MA16_Dca012052 [Dendrobium catenatum]
gene-MA16_Dca011153	LRR receptor-like serine/threonine-protein kinase GSO1 [Dendrobium catenatum]
gene-MA16_Dca008701	hypothetical protein MA16_Dca008701 [Dendrobium catenatum]
gene-MA16_Dca014238	hypothetical protein MA16_Dca014238 [Dendrobium catenatum]
gene-MA16_Dca002552	ruBisCO large subunit-binding protein subunit beta, chloroplastic [Dendrobium catenatum]
gene-MA16_Dca017065	Protein kinase 2B, chloroplastic [Dendrobium catenatum]
gene-MA16_Dca025880	protein CHROMATIN REMODELING 19 isoform X1 [Dendrobium catenatum]
gene-MA16_Dca009944	Putative ribonuclease H protein [Dendrobium catenatum]
gene-MA16_Dca002196	hypothetical protein MA16_Dca002196 [Dendrobium catenatum]
gene-MA16_Dca018681	B3 domain-containing protein Os07g0563300-like isoform X1 [Dendrobium catenatum]
gene-MA16_Dca013035	hypothetical protein MA16_Dca013035 [Dendrobium catenatum]
gene-MA16_Dca026072	L-ascorbate peroxidase, cytosolic [Dendrobium catenatum]
gene-MA16_Dca022287	hypothetical protein MA16_Dca022287 [Dendrobium catenatum]
gene-MA16_Dca019813	putative rRNA-processing protein EBP2 like [Dendrobium catenatum]
gene-MA16_Dca018101	conserved oligomeric Golgi complex subunit 7 [Dendrobium catenatum]
gene-MA16_Dca016862	hypothetical protein MA16_Dca016862 [Dendrobium catenatum]
gene-MA16_Dca002016	probable LRR receptor-like serine/threonine-protein kinase At5g45780 isoform X1 [Dendrobium catenatum]
gene-MA16_Dca014266	anaphase-promoting complex subunit 5 [Dendrobium catenatum]
gene-MA16_Dca013722	L-type lectin-domain containing receptor kinase IV.2 [Dendrobium catenatum]
gene-MA16_Dca000532	D-xylose-proton symporter-like 3, chloroplastic [Dendrobium catenatum]
gene-MA16_Dca000024	CTP synthase-like [Dendrobium catenatum]
gene-MA16_Dca017889	E3 ubiquitin-protein ligase HERC2-like [Dendrobium catenatum]
gene-MA16_Dca014566	F-box protein [Dendrobium catenatum]
gene-MA16_Dca005825	beta-glucosidase [Dendrobium catenatum]

gene-MA16_Dca009831	hypothetical protein MA16_Dca009831 [Dendrobium catenatum]
gene-MA16_Dca001741	mannose-specific lectin-like [Dendrobium catenatum]
gene-MA16_Dca000373	Cleavage and polyadenylation specificity factor subunit 3-II [Dendrobium catenatum]
gene-MA16_Dca013836	Outer envelope protein 64, mitochondrial [Dendrobium catenatum]
gene-MA16_Dca010212	protein PLASTID MOVEMENT IMPAIRED 2 [Dendrobium catenatum]
gene-MA16_Dca009514	geraniol 8-hydroxylase-like [Dendrobium catenatum]
gene-MA16_Dca009077	hypothetical protein MA16_Dca009077 [Dendrobium catenatum]
gene-MA16_Dca008286	protein trichome birefringence-like 28 [Dendrobium catenatum]
gene-MA16_Dca012210	BEL1-like homeodomain protein 6 [Dendrobium catenatum]
gene-MA16_Dca011551	putative mitochondrial protein [Dendrobium catenatum]
gene-MA16_Dca023283	Aspartic proteinase Asp1 [Dendrobium catenatum]
gene-MA16_Dca014602	putative cation-transporting ATPase [Dendrobium catenatum]
gene-MA16_Dca007693	hypothetical protein MA16_Dca007693 [Dendrobium catenatum]
gene-MA16_Dca010839	uncharacterized protein LOC110093216 [Dendrobium catenatum]
gene-MA16_Dca028126	Putative ribonuclease H protein [Dendrobium catenatum]
gene-MA16_Dca018635	ADP-ribosylation factor GTPase-activating protein AGD12 [Dendrobium catenatum]
gene-MA16_Dca027557	hypothetical protein MA16_Dca027557 [Dendrobium catenatum]
gene-MA16_Dca024868	myb family transcription factor EFM-like [Dendrobium catenatum]
gene-MA16_Dca010584	uncharacterized protein LOC110100289 [Dendrobium catenatum]
gene-MA16_Dca002874	putative pre-mRNA-splicing factor ATP-dependent RNA helicase [Dendrobium catenatum]
gene-MA16_Dca010234	hypothetical protein MA16_Dca010234 [Dendrobium catenatum]
gene-MA16_Dca021533	putative serine/threonine-protein kinase [Dendrobium catenatum]
gene-MA16_Dca008850	dirigent protein 6-like [Dendrobium catenatum]
gene-MA16_Dca021336	hypothetical protein MA16_Dca021336 [Dendrobium catenatum]
gene-MA16_Dca027918	hypothetical protein MA16_Dca027918 [Dendrobium catenatum]
gene-MA16_Dca026772	Retrovirus-related Pol polyprotein from transposon TNT 1-94 [Dendrobium catenatum]
gene-MA16_Dca015971	Putative ribonuclease H protein [Dendrobium catenatum]
gene-MA16_Dca010408	la-related protein 1B-like [Dendrobium catenatum]
gene-MA16_Dca016207	auxin-responsive protein SAUR50-like [Dendrobium catenatum]
gene-MA16_Dca005359	hypothetical protein MA16_Dca005359 [Dendrobium catenatum]

gene-MA16_Dca026221	protein farnesyltransferase subunit beta isoform X1 [Dendrobium catenatum]
gene-MA16_Dca014685	Transcription factor TCP13 [Dendrobium catenatum]
gene-MA16_Dca006200	protein WALLS ARE THIN 1 [Dendrobium catenatum]
gene-MA16_Dca004760	Heat shock cognate 70 kDa protein 2 [Dendrobium catenatum]
gene-MA16_Dca016504	Flap endonuclease GEN-like 1 [Dendrobium catenatum]
gene-MA16_Dca012778	putative LRR receptor-like serine/threonine-protein kinase [Dendrobium catenatum]
gene-MA16_Dca006532	hypothetical protein MA16_Dca006532 [Dendrobium catenatum]
gene-MA16_Dca021611	uncharacterized protein LOC110096793 [Dendrobium catenatum]
gene-MA16_Dca021947	hypothetical protein MA16_Dca021947 [Dendrobium catenatum]
gene-MA16_Dca011643	Transcription factor TCP3 [Dendrobium catenatum]
gene-MA16_Dca019943	Growth-regulating factor 5 [Dendrobium catenatum]
gene-MA16_Dca012469	Pyruvate, phosphate dikinase, chloroplastic [Dendrobium catenatum]
gene-MA16_Dca011938	hypothetical protein MA16_Dca011938 [Dendrobium catenatum]
gene-MA16_Dca005699	Putative disease resistance protein RGA4 [Dendrobium catenatum]
gene-MA16_Dca002738	proline iminopeptidase isoform X2 [Dendrobium catenatum]
gene-MA16_Dca002321	mitogen-activated protein kinase 9-like isoform X1 [Dendrobium catenatum]
gene-MA16_Dca024248	Subtilisin-like protease [Dendrobium catenatum]
gene-MA16_Dca000925	Ethylene-responsive transcription factor ERF025 [Dendrobium catenatum]
gene-MA16_Dca010432	hypothetical protein MA16_Dca010432 [Dendrobium catenatum]
gene-MA16_Dca023386	putative phytol kinase 2, chloroplastic [Dendrobium catenatum]
gene-MA16_Dca008623	polygalacturonase inhibitor 2-like [Dendrobium catenatum]
gene-MA16_Dca017447	Homeobox protein ATH1 [Dendrobium catenatum]
gene-MA16_Dca011335	Protein ACCUMULATION AND REPLICATION OF CHLOROPLASTS 6, chloroplastic [Dendrobium catenatum]
gene-MA16_Dca004006	BRCT domain-containing protein [Dendrobium catenatum]
gene-MA16_Dca015559	Pentatricopeptide repeat-containing protein [Dendrobium catenatum]
gene-MA16_Dca008877	hypothetical protein MA16_Dca008877 [Dendrobium catenatum]
gene-MA16_Dca008791	LRR receptor-like serine/threonine-protein kinase RPK2 [Dendrobium catenatum]
gene-MA16_Dca022638	DNA ligase 1 [Dendrobium catenatum]
gene-MA16_Dca018548	probable transcription factor At5g28040 [Dendrobium catenatum]
gene-MA16_Dca006828	uncharacterized protein LOC110092462 [Dendrobium catenatum]

gene-MA16_Dca017338	Filament-like plant protein 4 [Dendrobium catenatum]
gene-MA16_Dca015890	hypothetical protein MA16_Dca015890 [Dendrobium catenatum]
gene-MA16_Dca000766	hypothetical protein MA16_Dca000766 [Dendrobium catenatum]
gene-MA16_Dca023380	hypothetical protein MA16_Dca023380 [Dendrobium catenatum]
gene-MA16_Dca014756	Retrovirus-related Pol polyprotein from transposon TNT 1-94 [Dendrobium catenatum]
gene-MA16_Dca012694	Pumilio like 3 [Dendrobium catenatum]
gene-MA16_Dca011253	2-alkenal reductase (NADP(+)-dependent)-like isoform X1 [Dendrobium catenatum]
gene-MA16_Dca003268	uncharacterized protein LOC110093937 isoform X1 [Dendrobium catenatum]
gene-MA16_Dca021600	Pentatricopeptide repeat-containing protein [Dendrobium catenatum]
gene-MA16_Dca027711	Retrovirus-related Pol polyprotein from transposon TNT 1-94 [Dendrobium catenatum]
gene-MA16_Dca000936	Beta-fructofuranosidase, soluble isoenzyme I [Dendrobium catenatum]
gene-MA16_Dca021232	Disease resistance protein RGA2 [Dendrobium catenatum]
gene-MA16_Dca011708	Structural maintenance of chromosomes protein 2-1 [Dendrobium catenatum]
gene-MA16_Dca012991	hypothetical protein MA16_Dca012991 [Dendrobium catenatum]
gene-MA16_Dca001992	mitochondrial outer membrane porin-like [Dendrobium catenatum]
gene-MA16_Dca005769	uncharacterized protein LOC110099318 [Dendrobium catenatum]
gene-MA16_Dca019855	putative anion transporter 4, chloroplastic [Dendrobium catenatum]
gene-MA16_Dca006006	Protein argonaute 7 [Dendrobium catenatum]
gene-MA16_Dca025915	Short-chain dehydrogenase reductase 3b [Dendrobium catenatum]
gene-MA16_Dca023379	ERBB-3 BINDING PROTEIN 1 [Dendrobium catenatum]
gene-MA16_Dca021649	GDT1-like protein 2, chloroplastic [Dendrobium catenatum]
gene-MA16_Dca005141	DNA topoisomerase 2-like [Dendrobium catenatum]
gene-MA16_Dca021231	disease resistance protein RGA2-like [Dendrobium catenatum]
gene-MA16_Dca006228	hypothetical protein MA16_Dca006228 [Dendrobium catenatum]
gene-MA16_Dca019655	ER lumen protein retaining receptor [Dendrobium catenatum]
gene-MA16_Dca026631	Leucine-rich repeat receptor-like tyrosine-protein kinase [Dendrobium catenatum]
gene-MA16_Dca003391	uncharacterized protein LOC110100298 isoform X1 [Dendrobium catenatum]
gene-MA16_Dca001915	putative glucan endo-1,3-beta-glucosidase GVI [Dendrobium catenatum]
gene-MA16_Dca022009	hypothetical protein MA16_Dca022009 [Dendrobium catenatum]
gene-MA16_Dca008320	Retrovirus-related Pol polyprotein from transposon TNT 1-94 [Dendrobium catenatum]

gene-MA16_Dca027522	hypothetical protein MA16_Dca027522 [Dendrobium catenatum]
gene-MA16_Dca021729	hypothetical protein MA16_Dca021729 [Dendrobium catenatum]
gene-MA16_Dca005855	LOB domain-containing protein 6 [Dendrobium catenatum]
gene-MA16_Dca015660	long chain acyl-CoA synthetase 4-like [Dendrobium catenatum]
gene-MA16_Dca022026	probable inactive DNA (cytosine-5)-methyltransferase DRM3 [Dendrobium catenatum]
gene-MA16_Dca010331	Zinc finger CCCH domain-containing protein 37 [Dendrobium catenatum]
gene-MA16_Dca028537	Serine/threonine-protein phosphatase 7 long form like [Dendrobium catenatum]
gene-MA16_Dca017689	ABC transporter C family member 4-like isoform X1 [Dendrobium catenatum]
gene-MA16_Dca023581	Zinc finger CCCH domain-containing protein 18 [Dendrobium catenatum]
gene-MA16_Dca006479	Retrovirus-related Pol polyprotein from transposon TNT 1-94 [Dendrobium catenatum]
gene-MA16_Dca027705	SNARE-interacting protein KEULE-like [Dendrobium catenatum]
gene-MA16_Dca003190	protein MIZU-KUSSEI 1-like [Dendrobium catenatum]
gene-MA16_Dca007683	DNA repair endonuclease UVH1 [Dendrobium catenatum]
gene-MA16_Dca007578	LRR repeats and ubiquitin-like domain-containing protein [Dendrobium catenatum]
gene-MA16_Dca015921	laccase-3-like isoform X1 [Dendrobium catenatum]
gene-MA16_Dca017356	Receptor-like protein kinase HSL1 [Dendrobium catenatum]
gene-MA16_Dca002499	Uncharacterized protein MA16_Dca002499 [Dendrobium catenatum]
gene-MA16_Dca012886	kinesin-like protein KIN-7K, chloroplastic [Dendrobium catenatum]
gene-MA16_Dca010222	hypothetical protein MA16_Dca010222 [Dendrobium catenatum]
gene-MA16_Dca002534	oligopeptide transporter 4-like [Dendrobium catenatum]
gene-MA16_Dca023290	Protein translocase subunit SECA2, chloroplastic [Dendrobium catenatum]
gene-MA16_Dca001924	hypothetical protein MA16_Dca001924 [Dendrobium catenatum]
gene-MA16_Dca001616	putative LRR receptor-like serine/threonine-protein kinase [Dendrobium catenatum]
gene-MA16_Dca027576	hypothetical protein MA16_Dca027576 [Dendrobium catenatum]
gene-MA16_Dca004957	myb-related protein 330-like [Dendrobium catenatum]
gene-MA16_Dca017474	putative disease resistance protein RGA4 [Dendrobium catenatum]
gene-MA16_Dca011278	uncharacterized protein LOC110101662 [Dendrobium catenatum]
gene-MA16_Dca011630	RNA-dependent RNA polymerase 1 [Dendrobium catenatum]
gene-MA16_Dca009034	uncharacterized protein LOC110102985 [Dendrobium catenatum]
gene-MA16_Dca021948	mitogen-activated protein kinase kinase kinase YODA-like [Dendrobium catenatum]

gene-MA16_Dca021851	inositol-tetrakisphosphate 1-kinase 3-like isoform X1 [Dendrobium catenatum]
gene-MA16_Dca013089	F-box protein At1g70590 isoform X1 [Dendrobium catenatum]
gene-MA16_Dca023974	hypothetical protein MA16_Dca023974 [Dendrobium catenatum]
gene-MA16_Dca003102	NDR1/HIN1-like protein 10 [Dendrobium catenatum]
gene-MA16_Dca004266	Protein ASPARTIC PROTEASE IN GUARD CELL 2 [Dendrobium catenatum]
gene-MA16_Dca019888	scarecrow-like protein 23 [Dendrobium catenatum]
gene-MA16_Dca018300	putative disease resistance protein RGA4 [Dendrobium catenatum]
gene-MA16_Dca022585	SRSF protein kinase 2-like [Dendrobium catenatum]
gene-MA16_Dca012416	Heat stress transcription factor A-4b [Dendrobium catenatum]
gene-MA16_Dca011959	Growth-regulating factor 1 [Dendrobium catenatum]
gene-MA16_Dca021919	Retrovirus-related Pol polyprotein from transposon TNT 1-94 [Dendrobium catenatum]
gene-MA16_Dca007306	hypothetical protein MA16_Dca007306 [Dendrobium catenatum]
gene-MA16_Dca013237	uncharacterized protein LOC110107641 isoform X1 [Dendrobium catenatum]
gene-MA16_Dca026794	tonoplast dicarboxylate transporter [Dendrobium catenatum]
gene-MA16_Dca006397	Transcription factor GAMYB [Dendrobium catenatum]
gene-MA16_Dca001763	LEAF RUST 10 DISEASE-RESISTANCE LOCUS RECEPTOR-LIKE PROTEIN KINASE-like 1.2 [Dendrobium catenatum]
gene-MA16_Dca000956	Putative ribonuclease H protein [Dendrobium catenatum]
gene-MA16_Dca015434	probable pectinesterase/pectinesterase inhibitor 51 [Dendrobium catenatum]
gene-MA16_Dca005619	hypothetical protein MA16_Dca005619 [Dendrobium catenatum]
gene-MA16_Dca020425	cinnamoyl-CoA reductase-like SNL6 [Dendrobium catenatum]
gene-MA16_Dca010637	Myb-related protein 3R-1 [Dendrobium catenatum]
gene-MA16_Dca021033	bifunctional 3-dehydroquinate dehydratase/shikimate dehydrogenase, chloroplastic isoform X3 [Dendrobium catenatum]
gene-MA16_Dca026847	Protein translocase subunit SECA2, chloroplastic [Dendrobium catenatum]
gene-MA16_Dca001618	cystinosin homolog [Dendrobium catenatum]
gene-MA16_Dca004669	myb-related protein Myb4-like [Dendrobium catenatum]
gene-MA16_Dca020827	transcriptional regulator DEF1-like [Dendrobium catenatum]
gene-MA16_Dca018129	hypothetical protein MA16_Dca018129 [Dendrobium catenatum]
gene-MA16_Dca020948	26S proteasome non-ATPase regulatory subunit 1 homolog A-like [Dendrobium catenatum]
gene-MA16_Dca022587	cytochrome P450 93A3-like [Dendrobium catenatum]
gene-MA16_Dca020196	patellin-3-like [Dendrobium catenatum]

gene-MA16_Dca015938	protein MALE DISCOVERER 2-like [Dendrobium catenatum]
gene-MA16_Dca013596	hypothetical protein MA16_Dca013596 [Dendrobium catenatum]
gene-MA16_Dca006494	hypothetical protein MA16_Dca006494 [Dendrobium catenatum]
gene-MA16_Dca004354	hypothetical protein MA16_Dca004354 [Dendrobium catenatum]
gene-MA16_Dca022608	ATP-dependent DNA helicase PIF1 [Dendrobium catenatum]
gene-MA16_Dca024496	uncharacterized protein LOC110114634 [Dendrobium catenatum]
gene-MA16_Dca000558	hypothetical protein MA16_Dca000558 [Dendrobium catenatum]
gene-MA16_Dca008252	Fasciclin-like arabinogalactan protein 17 [Dendrobium catenatum]
gene-MA16_Dca005529	Formin-like protein 8 [Dendrobium catenatum]
gene-MA16_Dca003730	squamosa promoter-binding-like protein 18 [Dendrobium catenatum]
gene-MA16_Dca019935	hypothetical protein MA16_Dca019935 [Dendrobium catenatum]
gene-MA16_Dca006505	WRKY transcription factor SUSIBA2-like isoform X2 [Dendrobium catenatum]
gene-MA16_Dca011967	hypothetical protein MA16_Dca011967 [Dendrobium catenatum]
gene-MA16_Dca012195	chorismate mutase 1, chloroplastic [Dendrobium catenatum]
gene-MA16_Dca014645	Serine carboxypeptidase-like 6 [Dendrobium catenatum]
gene-MA16_Dca009661	3-oxo-Delta(4,5)-steroid 5-beta-reductase [Dendrobium catenatum]
gene-MA16_Dca029126	hypothetical protein MA16_Dca029126 [Dendrobium catenatum]
gene-MA16_Dca014542	protein SABRE isoform X1 [Dendrobium catenatum]
gene-MA16_Dca019890	Putative disease resistance protein RGA4 [Dendrobium catenatum]
gene-MA16_Dca012932	E3 ubiquitin-protein ligase MBR2-like [Dendrobium catenatum]
gene-MA16_Dca026559	auxin efflux carrier component 7-like isoform X1 [Dendrobium catenatum]
gene-MA16_Dca021281	TPR repeat-containing thioredoxin TTL2 [Dendrobium catenatum]
gene-MA16_Dca006965	hypothetical protein MA16_Dca006965 [Dendrobium catenatum]
gene-MA16_Dca009296	Serine/threonine-protein kinase ATM [Dendrobium catenatum]
gene-MA16_Dca016000	putative UDP-sugar transporter DDB_G0278631 [Dendrobium catenatum]
gene-MA16_Dca028825	hypothetical protein MA16_Dca028825 [Dendrobium catenatum]
gene-MA16_Dca023972	ubiquitin-40S ribosomal protein S27a [Dendrobium catenatum]
gene-MA16_Dca018087	GATA transcription factor 4-like [Dendrobium catenatum]
gene-MA16_Dca012140	hypothetical protein MA16_Dca012140 [Dendrobium catenatum]
gene-MA16_Dca016761	squamosa promoter-binding-like protein 18 isoform X1 [Dendrobium catenatum]

gene-MA16_Dca001782	Potassium transporter 26 [Dendrobium catenatum]
gene-MA16_Dca012607	uncharacterized protein LOC110099029 [Dendrobium catenatum]
gene-MA16_Dca005916	anamorsin homolog [Dendrobium catenatum]
gene-MA16_Dca024782	DNA polymerase epsilon subunit 4 isoform X1 [Dendrobium catenatum]
gene-MA16_Dca018237	probable LRR receptor-like serine/threonine-protein kinase At5g10290 isoform X2 [Dendrobium catenatum]
gene-MA16_Dca018980	hypothetical protein MA16_Dca018980 [Dendrobium catenatum]
gene-MA16_Dca019265	oligopeptide transporter 1-like [Dendrobium catenatum]
gene-MA16_Dca004861	RING-H2 finger protein ATL60 [Dendrobium catenatum]
gene-MA16_Dca007845	LRR receptor-like serine/threonine-protein kinase EFR [Dendrobium catenatum]
gene-MA16_Dca003306	epoxide hydrolase 4 [Dendrobium catenatum]
gene-MA16_Dca028599	BTB/POZ domain-containing protein [Dendrobium catenatum]
gene-MA16_Dca010312	hypothetical protein MA16_Dca010312 [Dendrobium catenatum]
gene-MA16_Dca008224	Putative disease resistance protein RGA3 [Dendrobium catenatum]
gene-MA16_Dca021264	pentatricopeptide repeat-containing protein At1g01970 isoform X1 [Dendrobium catenatum]
gene-MA16_Dca003454	hypothetical protein MA16_Dca003454 [Dendrobium catenatum]
gene-MA16_Dca013843	Putative ribonuclease H protein [Dendrobium catenatum]
gene-MA16_Dca024442	probable UDP-arabinose 4-epimerase 2 [Dendrobium catenatum]
gene-MA16_Dca024079	protein DJ-1 homolog B [Dendrobium catenatum]
gene-MA16_Dca021440	putative mitochondrial protein [Dendrobium catenatum]
gene-MA16_Dca024872	hypothetical protein MA16_Dca024872 [Dendrobium catenatum]
gene-MA16_Dca014483	hypothetical protein MA16_Dca014483 [Dendrobium catenatum]
gene-MA16_Dca004340	arginyl-tRNA--protein transferase 2-like isoform X1 [Dendrobium catenatum]
gene-MA16_Dca001502	hypothetical protein MA16_Dca001502 [Dendrobium catenatum]
gene-MA16_Dca018794	uncharacterized protein LOC110100477 [Dendrobium catenatum]
gene-MA16_Dca016389	Diaminopimelate decarboxylase 1, chloroplastic [Dendrobium catenatum]
gene-MA16_Dca027963	ATP synthase subunit alpha, mitochondrial [Dendrobium catenatum]
gene-MA16_Dca003646	glycerol-3-phosphate acyltransferase 1-like [Dendrobium catenatum]
gene-MA16_Dca008655	hypothetical protein MA16_Dca008655 [Dendrobium catenatum]
gene-MA16_Dca010688	GDSL esterase/lipase At1g71691-like [Dendrobium catenatum]
gene-MA16_Dca012598	uncharacterized protein LOC110099064 [Dendrobium catenatum]

gene-MA16_Dca021754	hypothetical protein MA16_Dca021754 [Dendrobium catenatum]
gene-MA16_Dca018178	hypothetical protein MA16_Dca018178 [Dendrobium catenatum]
gene-MA16_Dca017527	DNA repair helicase UVH6 [Dendrobium catenatum]
gene-MA16_Dca025192	spindle assembly checkpoint component MAD1 [Dendrobium catenatum]
gene-MA16_Dca011382	putative mitochondrial protein [Dendrobium catenatum]
gene-MA16_Dca024277	uncharacterized protein LOC110103864 [Dendrobium catenatum]
gene-MA16_Dca021112	hypothetical protein MA16_Dca021112 [Dendrobium catenatum]
gene-MA16_Dca006726	LYR motif-containing protein 4 [Dendrobium catenatum]
gene-MA16_Dca022778	hypothetical protein MA16_Dca022778 [Dendrobium catenatum]
gene-MA16_Dca011500	Protein SCAR2 [Dendrobium catenatum]
gene-MA16_Dca006942	putative mitochondrial protein [Dendrobium catenatum]
gene-MA16_Dca027664	hypothetical protein MA16_Dca027664 [Dendrobium catenatum]
gene-MA16_Dca005998	hypothetical protein MA16_Dca005998 [Dendrobium catenatum]
gene-MA16_Dca007518	1-aminocyclopropane-1-carboxylate synthase-like [Dendrobium catenatum]
gene-MA16_Dca003158	putative peptide/nitrate transporter [Dendrobium catenatum]
gene-MA16_Dca018248	Inactive TPR repeat-containing thioredoxin TTL3 [Dendrobium catenatum]
gene-MA16_Dca010390	putative mitochondrial protein [Dendrobium catenatum]
gene-MA16_Dca003429	Serine carboxypeptidase-like 36 [Dendrobium catenatum]
gene-MA16_Dca011523	hypothetical protein MA16_Dca011523 [Dendrobium catenatum]
gene-MA16_Dca012021	Beta-glucosidase 6 [Dendrobium catenatum]
gene-MA16_Dca017709	trans-resveratrol di-O-methyltransferase-like [Dendrobium catenatum]
gene-MA16_Dca000262	F-box protein SKIP14 [Dendrobium catenatum]
gene-MA16_Dca012310	pectinesterase-like [Dendrobium catenatum]
gene-MA16_Dca011228	probable xyloglucan galactosyltransferase GT17 [Dendrobium catenatum]
gene-MA16_Dca018102	integrator complex subunit 11 [Dendrobium catenatum]
gene-MA16_Dca024450	ectonucleotide pyrophosphatase/phosphodiesterase family member 3-like [Dendrobium catenatum]
gene-MA16_Dca020442	putative protein phosphatase 2C 77 [Dendrobium catenatum]
gene-MA16_Dca022876	Squamosa promoter-binding-like protein 15 [Dendrobium catenatum]
gene-MA16_Dca006916	Agamous-like MADS-box protein AGL61 [Dendrobium catenatum]
gene-MA16_Dca023861	Putative disease resistance protein RGA3 [Dendrobium catenatum]

gene-MA16_Dca010485	hypothetical protein MA16_Dca010485 [Dendrobium catenatum]
gene-MA16_Dca021254	hypothetical protein MA16_Dca021254 [Dendrobium catenatum]
gene-MA16_Dca019802	rRNA biogenesis protein RRP5 isoform X2 [Dendrobium catenatum]
gene-MA16_Dca004396	9-cis-epoxycarotenoid dioxygenase, chloroplastic [Dendrobium catenatum]
gene-MA16_Dca018127	E3 ubiquitin protein ligase DRIP2-like [Dendrobium catenatum]
gene-MA16_Dca008112	putative pentatricopeptide repeat-containing protein At5g06400, mitochondrial [Dendrobium catenatum]
gene-MA16_Dca026614	sorting nexin 2B-like isoform X2 [Dendrobium catenatum]
gene-MA16_Dca008882	splicing factor 3B subunit 3-like [Dendrobium catenatum]
gene-MA16_Dca019551	hypothetical protein MA16_Dca019551 [Dendrobium catenatum]
gene-MA16_Dca025147	hypothetical protein MA16_Dca025147 [Dendrobium catenatum]
gene-MA16_Dca001544	hypothetical protein MA16_Dca001544 [Dendrobium catenatum]
gene-MA16_Dca015701	hypothetical protein MA16_Dca015701 [Dendrobium catenatum]
gene-MA16_Dca014628	uncharacterized protein PFB0145c-like isoform X1 [Dendrobium catenatum]
gene-MA16_Dca014256	Serine/threonine-protein kinase PBS1 [Dendrobium catenatum]
gene-MA16_Dca009355	ATP sulfurylase 1, chloroplastic-like [Dendrobium catenatum]
gene-MA16_Dca008355	hypothetical protein MA16_Dca008355 [Dendrobium catenatum]
gene-MA16_Dca013012	uncharacterized protein LOC110097362 isoform X1 [Dendrobium catenatum]
gene-MA16_Dca001585	hypothetical protein MA16_Dca001585 [Dendrobium catenatum]
gene-MA16_Dca006571	hypothetical protein MA16_Dca006571 [Dendrobium catenatum]
gene-MA16_Dca006076	probable E3 ubiquitin-protein ligase XBOS34 isoform X1 [Dendrobium catenatum]
gene-MA16_Dca022269	Serine/threonine-protein kinase TOR [Dendrobium catenatum]
gene-MA16_Dca009503	hypothetical protein MA16_Dca009503 [Dendrobium catenatum]
gene-MA16_Dca018731	hypothetical protein MA16_Dca018731 [Dendrobium catenatum]
gene-MA16_Dca023966	Retrovirus-related Pol polyprotein from transposon TNT 1-94 [Dendrobium catenatum]
gene-MA16_Dca017000	target of Myb protein 1 isoform X1 [Dendrobium catenatum]
gene-MA16_Dca021446	heat shock protein 81-1-like [Dendrobium catenatum]
gene-MA16_Dca021677	hypothetical protein MA16_Dca021677 [Dendrobium catenatum]
gene-MA16_Dca018172	ETHYLENE INSENSITIVE 3-like 1 protein [Dendrobium catenatum]
gene-MA16_Dca000690	hypothetical protein MA16_Dca000690 [Dendrobium catenatum]
gene-MA16_Dca008526	Phospholipid-transporting ATPase 1 [Dendrobium catenatum]

gene-MA16_Dca013368	auxin response factor 17-like [Dendrobium catenatum]
gene-MA16_Dca011199	Ent-kaurene oxidase, chloroplastic [Dendrobium catenatum]
gene-MA16_Dca003493	uncharacterized protein LOC110094340 isoform X1 [Dendrobium catenatum]
gene-MA16_Dca008478	VAN3-binding protein [Dendrobium catenatum]
gene-MA16_Dca003133	uncharacterized protein LOC110094072 [Dendrobium catenatum]
gene-MA16_Dca003495	putative vesicle-associated membrane protein 726 [Dendrobium catenatum]
gene-MA16_Dca010505	Tetrahydrocannabinolic acid synthase [Dendrobium catenatum]
gene-MA16_Dca003855	uncharacterized protein LOC110104967 [Dendrobium catenatum]
gene-MA16_Dca006119	peptide-N4-(N-acetyl-beta-glucosaminyl)asparagine amidase A-like [Dendrobium catenatum]
gene-MA16_Dca026967	E3 ubiquitin-protein ligase SINA-like 10 [Dendrobium catenatum]
gene-MA16_Dca007810	probably inactive receptor-like protein kinase At2g46850 [Dendrobium catenatum]
gene-MA16_Dca005803	translation initiation factor eIF-2B subunit beta isoform X1 [Dendrobium catenatum]
gene-MA16_Dca008680	Putative ribonuclease H protein [Dendrobium catenatum]
gene-MA16_Dca014980	Beta-D-xylosidase 4 [Dendrobium catenatum]
gene-MA16_Dca003355	phospholipase D alpha 1-like [Dendrobium catenatum]
gene-MA16_Dca024651	hypothetical protein MA16_Dca024651 [Dendrobium catenatum]
gene-MA16_Dca011697	Pentatricopeptide repeat-containing protein [Dendrobium catenatum]
gene-MA16_Dca020359	disease resistance protein RGA2-like [Dendrobium catenatum]
gene-MA16_Dca016456	squamosa promoter-binding-like protein 14 isoform X2 [Dendrobium catenatum]
gene-MA16_Dca019642	uncharacterized protein LOC110109304 isoform X1 [Dendrobium catenatum]
gene-MA16_Dca001622	Eukaryotic translation initiation factor isoform 4G-1 [Dendrobium catenatum]
gene-MA16_Dca016864	Long chain acyl-CoA synthetase 9, chloroplastic [Dendrobium catenatum]
gene-MA16_Dca004993	putative disease resistance protein RGA1 [Dendrobium catenatum]
gene-MA16_Dca004403	piriformospora indica-insensitive protein 2-like [Dendrobium catenatum]
gene-MA16_Dca004293	pectinesterase-like [Dendrobium catenatum]
gene-MA16_Dca015383	transport inhibitor response 1-like protein [Dendrobium catenatum]
gene-MA16_Dca007851	Pleiotropic drug resistance protein 12 [Dendrobium catenatum]
gene-MA16_Dca009995	Clustered mitochondria protein [Dendrobium catenatum]
gene-MA16_Dca014331	protein trichome birefringence-like 26 [Dendrobium catenatum]
gene-MA16_Dca006234	hypothetical protein MA16_Dca006234 [Dendrobium catenatum]

gene-MA16_Dca009490	hypothetical protein MA16_Dca009490 [Dendrobium catenatum]
gene-MA16_Dca007185	golgin candidate 5 isoform X2 [Dendrobium catenatum]
gene-MA16_Dca027712	F-box protein [Dendrobium catenatum]
gene-MA16_Dca003369	two-component response regulator ORR22-like [Dendrobium catenatum]
gene-MA16_Dca026478	aspartokinase 2, chloroplastic-like [Dendrobium catenatum]
gene-MA16_Dca020802	uncharacterized protein LOC110104429 isoform X2 [Dendrobium catenatum]
gene-MA16_Dca013506	universal stress protein PHOS34 [Dendrobium catenatum]
gene-MA16_Dca022196	U-box domain-containing protein 17-like [Dendrobium catenatum]
gene-MA16_Dca019875	hypothetical protein MA16_Dca019875 [Dendrobium catenatum]
gene-MA16_Dca001987	uncharacterized protein LOC110100094 [Dendrobium catenatum]
gene-MA16_Dca003862	hypothetical protein MA16_Dca003862 [Dendrobium catenatum]
gene-MA16_Dca003718	Flowering time control protein FCA [Dendrobium catenatum]
gene-MA16_Dca002878	Small ubiquitin-related modifier 1 [Dendrobium catenatum]
gene-MA16_Dca004133	31 kDa ribonucleoprotein, chloroplastic [Dendrobium catenatum]
gene-MA16_Dca006414	expansin-A18-like [Dendrobium catenatum]
gene-MA16_Dca018150	Transcriptional regulator TAC1 [Dendrobium catenatum]
gene-MA16_Dca012666	ubiquitin carboxyl-terminal hydrolase 12-like isoform X1 [Dendrobium catenatum]
gene-MA16_Dca003650	probable methyltransferase PMT23 isoform X5 [Dendrobium catenatum]
gene-MA16_Dca012190	probable serine/threonine-protein kinase At1g54610 [Dendrobium catenatum]
gene-MA16_Dca021000	Mannan endo-1,4-beta-mannosidase 1 [Dendrobium catenatum]
gene-MA16_Dca002089	Sucrose transport protein SUT1 [Dendrobium catenatum]
gene-MA16_Dca022649	Retrovirus-related Pol polyprotein from transposon TNT 1-94 [Dendrobium catenatum]
gene-MA16_Dca006258	VIN3-like protein 2 isoform X2 [Dendrobium catenatum]
gene-MA16_Dca003404	Trans-resveratrol di-O-methyltransferase [Dendrobium catenatum]
gene-MA16_Dca025220	uncharacterized protein LOC110106358 [Dendrobium catenatum]
gene-MA16_Dca000440	zinc finger protein ZAT4-like [Dendrobium catenatum]
gene-MA16_Dca010102	Retrovirus-related Pol polyprotein from transposon TNT 1-94 [Dendrobium catenatum]
gene-MA16_Dca028386	Putative AC transposase [Dendrobium catenatum]
gene-MA16_Dca028026	disease resistance protein RGA2-like [Dendrobium catenatum]
gene-MA16_Dca013631	MLO-like protein 10 [Dendrobium catenatum]

gene-MA16_Dca014352	diacylglycerol kinase 2 isoform X1 [Dendrobium catenatum]
gene-MA16_Dca017756	putative G3BP-like protein isoform X1 [Dendrobium catenatum]
gene-MA16_Dca000527	Protein TIC 62, chloroplastic [Dendrobium catenatum]
gene-MA16_Dca027342	RNA-directed DNA polymerase [Dendrobium catenatum]
gene-MA16_Dca001937	RNA-binding protein 1-like [Dendrobium catenatum]
gene-MA16_Dca007949	Protein ROOT HAIR DEFECTIVE 3 [Dendrobium catenatum]
gene-MA16_Dca010554	urease accessory protein D isoform X1 [Dendrobium catenatum]
gene-MA16_Dca003096	DNA repair protein REV1 [Dendrobium catenatum]
gene-MA16_Dca020624	transcription factor GAMYB-like isoform X1 [Dendrobium catenatum]
gene-MA16_Dca023804	Retrovirus-related Pol polyprotein from transposon TNT 1-94 [Dendrobium catenatum]
gene-MA16_Dca021431	putative inactive purple acid phosphatase 27 [Dendrobium catenatum]
gene-MA16_Dca019789	hypothetical protein MA16_Dca019789 [Dendrobium catenatum]
gene-MA16_Dca001074	TLC domain-containing protein At5g14285-like [Dendrobium catenatum]
gene-MA16_Dca020751	Homeobox-leucine zipper protein HOX32 [Dendrobium catenatum]
gene-MA16_Dca000621	hypothetical protein MA16_Dca000621 [Dendrobium catenatum]
gene-MA16_Dca017812	hypothetical protein MA16_Dca017812 [Dendrobium catenatum]
gene-MA16_Dca024139	auxin response factor 2-like [Dendrobium catenatum]
gene-MA16_Dca017977	betaine aldehyde dehydrogenase 1, chloroplastic-like [Dendrobium catenatum]
gene-MA16_Dca000803	Retrovirus-related Pol polyprotein from transposon TNT 1-94 [Dendrobium catenatum]
gene-MA16_Dca026346	riboflavin kinase [Dendrobium catenatum]
gene-MA16_Dca000885	uncharacterized protein LOC110096403 [Dendrobium catenatum]
gene-MA16_Dca025898	5'-3' exoribonuclease 3 [Dendrobium catenatum]
gene-MA16_Dca012420	Dynein assembly factor with WDR repeat domains 1 [Dendrobium catenatum]
gene-MA16_Dca004713	hypothetical protein MA16_Dca004713 [Dendrobium catenatum]
gene-MA16_Dca010185	Vacuolar iron transporter like 4 [Dendrobium catenatum]
gene-MA16_Dca001400	lecithin-cholesterol acyltransferase-like 4 [Dendrobium catenatum]
gene-MA16_Dca009426	mitochondrial uncoupling protein 1 [Dendrobium catenatum]
gene-MA16_Dca023997	CBL-interacting protein kinase 32-like isoform X1 [Dendrobium catenatum]
gene-MA16_Dca003182	exocyst complex component EXO70A1 [Dendrobium catenatum]
gene-MA16_Dca017222	protein NETWORKED 4B-like [Dendrobium catenatum]

gene-MA16_Dca024978	Signal recognition particle 54 kDa protein, chloroplastic [Dendrobium catenatum]
gene-MA16_Dca011869	Reticulon-like protein B3 [Dendrobium catenatum]
gene-MA16_Dca011924	hypothetical protein MA16_Dca011924 [Dendrobium catenatum]
gene-MA16_Dca014156	tubulin-folding cofactor C [Dendrobium catenatum]
gene-MA16_Dca007432	Fanconi anemia group D2 protein-like isoform X1 [Dendrobium catenatum]
gene-MA16_Dca006059	uncharacterized protein LOC110093267 isoform X1 [Dendrobium catenatum]
gene-MA16_Dca019302	putative protein phosphatase 2C 63 [Dendrobium catenatum]
gene-MA16_Dca020345	uncharacterized protein LOC110113002 [Dendrobium catenatum]
gene-MA16_Dca004037	Protein argonaute 1B [Dendrobium catenatum]
gene-MA16_Dca013152	transcription elongation factor TFIIS [Dendrobium catenatum]
gene-MA16_Dca024050	protein root UVB sensitive 3 isoform X1 [Dendrobium catenatum]
gene-MA16_Dca017945	putative protein phosphatase 2C 1 [Dendrobium catenatum]
gene-MA16_Dca003872	Trans-resveratrol di-O-methyltransferase [Dendrobium catenatum]
gene-MA16_Dca010932	putative mitochondrial protein [Dendrobium catenatum]
gene-MA16_Dca018814	sulfhydryl oxidase 2-like isoform X1 [Dendrobium catenatum]
gene-MA16_Dca011159	scarecrow-like protein 23 [Dendrobium catenatum]
gene-MA16_Dca003505	hypothetical protein MA16_Dca003505 [Dendrobium catenatum]
gene-MA16_Dca020889	Retrovirus-related Pol polyprotein from transposon TNT 1-94 [Dendrobium catenatum]
gene-MA16_Dca024344	putative TPR repeat-containing protein [Dendrobium catenatum]
gene-MA16_Dca012844	hypothetical protein MA16_Dca012844 [Dendrobium catenatum]
gene-MA16_Dca005545	hypothetical protein MA16_Dca005545 [Dendrobium catenatum]
gene-MA16_Dca012254	F-box protein [Dendrobium catenatum]
gene-MA16_Dca000721	hypothetical protein MA16_Dca000721 [Dendrobium catenatum]
gene-MA16_Dca010056	uncharacterized protein LOC110101953 isoform X2 [Dendrobium catenatum]
gene-MA16_Dca011830	vacuolar protein sorting-associated protein 18 homolog [Dendrobium catenatum]
gene-MA16_Dca023247	Molybdenum cofactor sulfurase [Dendrobium catenatum]
gene-MA16_Dca022824	hypothetical protein MA16_Dca022824 [Dendrobium catenatum]
gene-MA16_Dca021128	BURP domain-containing protein 12 [Dendrobium catenatum]
gene-MA16_Dca012774	uncharacterized protein LOC110103518 [Dendrobium catenatum]
gene-MA16_Dca006561	probable glucuronosyltransferase Os06g0687900 [Dendrobium catenatum]

gene-MA16_Dca004095	hypothetical protein MA16_Dca004095 [Dendrobium catenatum]
gene-MA16_Dca004775	protein OSCA1-like [Dendrobium catenatum]
gene-MA16_Dca016488	hypothetical protein MA16_Dca016488 [Dendrobium catenatum]
gene-MA16_Dca013923	Retrovirus-related Pol polyprotein from transposon TNT 1-94 [Dendrobium catenatum]
gene-MA16_Dca004416	protein PLASTID TRANSCRIPTIONALLY ACTIVE 12 isoform X1 [Dendrobium catenatum]
gene-MA16_Dca016707	endoglucanase 9-like [Dendrobium catenatum]
gene-MA16_Dca027629	hypothetical protein MA16_Dca027629 [Dendrobium catenatum]
gene-MA16_Dca006817	phospholipase A(1) DAD1, chloroplastic [Dendrobium catenatum]
gene-MA16_Dca026452	Fatty acyl-CoA reductase 3 [Dendrobium catenatum]
gene-MA16_Dca001936	growth-regulating factor 6-like [Dendrobium catenatum]
gene-MA16_Dca008127	ubiquitin carboxyl-terminal hydrolase 3-like [Dendrobium catenatum]
gene-MA16_Dca008125	putative lysine-specific demethylase ELF6 [Dendrobium catenatum]
gene-MA16_Dca028949	hypothetical protein MA16_Dca028949 [Dendrobium catenatum]
gene-MA16_Dca015009	putative mitochondrial protein [Dendrobium catenatum]
gene-MA16_Dca001894	Magnesium-chelatase subunit ChlD, chloroplastic [Dendrobium catenatum]
gene-MA16_Dca025801	Retrovirus-related Pol polyprotein from transposon TNT 1-94 [Dendrobium catenatum]
gene-MA16_Dca021804	uncharacterized protein LOC110102133 [Dendrobium catenatum]
gene-MA16_Dca016056	leucine-rich repeat extensin-like protein 2 [Dendrobium catenatum]
gene-MA16_Dca027203	hypothetical protein MA16_Dca027203 [Dendrobium catenatum]
gene-MA16_Dca010287	Retrovirus-related Pol polyprotein from transposon TNT 1-94 [Dendrobium catenatum]
gene-MA16_Dca023584	hypothetical protein MA16_Dca023584 [Dendrobium catenatum]
gene-MA16_Dca010966	Remorin [Dendrobium catenatum]
gene-MA16_Dca016860	uncharacterized protein LOC110116224 isoform X1 [Dendrobium catenatum]
gene-MA16_Dca003379	protein FAR1-RELATED SEQUENCE 5-like [Dendrobium catenatum]
gene-MA16_Dca015330	extracellular signal-regulated kinase 1 isoform X1 [Dendrobium catenatum]
gene-MA16_Dca015159	Putative ribonuclease H protein [Dendrobium catenatum]
gene-MA16_Dca019811	ubiquitin-like-specific protease ESD4 [Dendrobium catenatum]
gene-MA16_Dca005683	uncharacterized protein LOC110092832 [Dendrobium catenatum]
gene-MA16_Dca016373	hypothetical protein MA16_Dca016373 [Dendrobium catenatum]
gene-MA16_Dca013535	putative mitochondrial protein [Dendrobium catenatum]

gene-MA16_Dca016419	F-box/LRR-repeat protein 20-like [Dendrobium catenatum]
gene-MA16_Dca015563	hypothetical protein MA16_Dca015563 [Dendrobium catenatum]
gene-MA16_Dca010144	Ubiquitin-60S ribosomal protein L40-2 [Dendrobium catenatum]
gene-MA16_Dca008435	Putative disease resistance protein RGA3 [Dendrobium catenatum]
gene-MA16_Dca000308	uncharacterized protein LOC110111907 [Dendrobium catenatum]
gene-MA16_Dca005656	Receptor-like protein kinase HAIKU2 [Dendrobium catenatum]
gene-MA16_Dca022885	transcription factor bHLH68 isoform X1 [Dendrobium catenatum]
gene-MA16_Dca006429	abscisic acid 8'-hydroxylase 3 [Dendrobium catenatum]
gene-MA16_Dca008499	transcription factor TCP24-like [Dendrobium catenatum]
gene-MA16_Dca024081	Malate dehydrogenase 1, mitochondrial [Dendrobium catenatum]
gene-MA16_Dca013966	DNA polymerase alpha subunit B [Dendrobium catenatum]
gene-MA16_Dca010306	Secologanin synthase [Dendrobium catenatum]
gene-MA16_Dca003794	hypothetical protein MA16_Dca003794 [Dendrobium catenatum]
gene-MA16_Dca024328	uncharacterized protein LOC110115567 [Dendrobium catenatum]
gene-MA16_Dca006666	CBL-interacting protein kinase 18-like [Dendrobium catenatum]
gene-MA16_Dca010506	Retrovirus-related Pol polyprotein from transposon TNT 1-94 [Dendrobium catenatum]
gene-MA16_Dca008231	hypothetical protein MA16_Dca008231 [Dendrobium catenatum]
gene-MA16_Dca010022	histidine kinase 5 [Dendrobium catenatum]
gene-MA16_Dca010684	14 kDa proline-rich protein DC2.15 [Dendrobium catenatum]
gene-MA16_Dca018560	hypothetical protein MA16_Dca018560 [Dendrobium catenatum]
gene-MA16_Dca000121	peroxidase P7-like [Dendrobium catenatum]
gene-MA16_Dca017549	large proline-rich protein bag6-B isoform X3 [Dendrobium catenatum]
gene-MA16_Dca004013	Retrovirus-related Pol polyprotein from transposon TNT 1-94 [Dendrobium catenatum]
gene-MA16_Dca026000	hypothetical protein MA16_Dca026000 [Dendrobium catenatum]
gene-MA16_Dca023432	ABC transporter G family member 25 [Dendrobium catenatum]
gene-MA16_Dca020464	uncharacterized protein LOC110100595 [Dendrobium catenatum]
gene-MA16_Dca014017	Cytochrome P450 78A11 [Dendrobium catenatum]
gene-MA16_Dca001389	hypothetical protein MA16_Dca001389 [Dendrobium catenatum]
gene-MA16_Dca001377	dnaJ homolog subfamily B member 13-like [Dendrobium catenatum]
gene-MA16_Dca028745	Serine/threonine-protein phosphatase 7 long form like [Dendrobium catenatum]

gene-MA16_Dca026850	hypothetical protein MA16_Dca026850 [Dendrobium catenatum]
gene-MA16_Dca018330	uncharacterized protein LOC110105762 isoform X1 [Dendrobium catenatum]
gene-MA16_Dca006125	dnaJ homolog subfamily C member 2 [Dendrobium catenatum]
gene-MA16_Dca013294	Putative disease resistance protein RGA4 [Dendrobium catenatum]
gene-MA16_Dca006326	plastidic ATP/ADP-transporter-like [Dendrobium catenatum]
gene-MA16_Dca025965	endoglucanase 17-like [Dendrobium catenatum]
gene-MA16_Dca006033	uncharacterized protein At1g04910-like [Dendrobium catenatum]
gene-MA16_Dca000483	endoribonuclease Dicer homolog 2a-like [Dendrobium catenatum]
gene-MA16_Dca024599	putative pentatricopeptide repeat-containing protein At3g25060, mitochondrial isoform X1 [Dendrobium catenatum]
gene-MA16_Dca023412	protein NRT1/ PTR FAMILY 4.4-like [Dendrobium catenatum]
gene-MA16_Dca007135	Zinc finger protein HD1 [Dendrobium catenatum]
gene-MA16_Dca007455	hypothetical protein MA16_Dca007455 [Dendrobium catenatum]
gene-MA16_Dca018586	hypothetical protein MA16_Dca018586 [Dendrobium catenatum]
gene-MA16_Dca018922	pentatricopeptide repeat-containing protein At5g56310 [Dendrobium catenatum]
gene-MA16_Dca002003	homeobox-DDT domain protein RLT2-like isoform X2 [Dendrobium catenatum]
gene-MA16_Dca024854	Serine/threonine-protein kinase Nek2 [Dendrobium catenatum]
gene-MA16_Dca023206	putative leucine-rich repeat receptor-like protein kinase [Dendrobium catenatum]
gene-MA16_Dca007667	hypothetical protein MA16_Dca007667 [Dendrobium catenatum]
gene-MA16_Dca015443	Phosphoenolpyruvate carboxykinase [ATP] [Dendrobium catenatum]
gene-MA16_Dca023515	hypothetical protein MA16_Dca023515 [Dendrobium catenatum]
gene-MA16_Dca022858	pentatricopeptide repeat-containing protein At4g15720-like [Dendrobium catenatum]
gene-MA16_Dca007198	U3 small nucleolar ribonucleoprotein protein MPP10 [Dendrobium catenatum]
gene-MA16_Dca014134	FAD synthetase [Dendrobium catenatum]
gene-MA16_Dca019107	Putative disease resistance RPP13-like protein 1 [Dendrobium catenatum]
gene-MA16_Dca012435	alpha,alpha-trehalose-phosphate synthase [UDP-forming] 1-like [Dendrobium catenatum]
gene-MA16_Dca012251	Receptor-like serine/threonine-protein kinase SD1-8 [Dendrobium catenatum]
gene-MA16_Dca003873	hypothetical protein MA16_Dca003873 [Dendrobium catenatum]
gene-MA16_Dca026235	hypothetical protein MA16_Dca026235 [Dendrobium catenatum]
gene-MA16_Dca022185	uncharacterized protein LOC110112447 isoform X1 [Dendrobium catenatum]
gene-MA16_Dca016371	L-type lectin-domain containing receptor kinase IX.1-like [Dendrobium catenatum]

gene-MA16_Dca000943	putative aarF domain-containing protein kinase [Dendrobium catenatum]
gene-MA16_Dca009014	BTB/POZ domain-containing protein At5g67385 [Dendrobium catenatum]
gene-MA16_Dca020882	transcription factor MYBS3-like [Dendrobium catenatum]
gene-MA16_Dca008768	adenine/guanine permease AZG1 [Dendrobium catenatum]
gene-MA16_Dca012826	hypothetical protein MA16_Dca012826 [Dendrobium catenatum]
gene-MA16_Dca007417	two-component response regulator ORR9-like [Dendrobium catenatum]
gene-MA16_Dca019188	hypothetical protein MA16_Dca019188 [Dendrobium catenatum]
gene-MA16_Dca012870	DNA excision repair protein ERCC-1 [Dendrobium catenatum]
gene-MA16_Dca016432	hypothetical protein MA16_Dca016432 [Dendrobium catenatum]
gene-MA16_Dca012177	FACT complex subunit SPT16 [Dendrobium catenatum]
gene-MA16_Dca019039	uncharacterized protein LOC110103721 [Dendrobium catenatum]
gene-MA16_Dca004007	uncharacterized protein LOC110097597 [Dendrobium catenatum]
gene-MA16_Dca021333	hypothetical protein MA16_Dca021333 [Dendrobium catenatum]
gene-MA16_Dca000698	pentatricopeptide repeat-containing protein At3g49240-like [Dendrobium catenatum]
gene-MA16_Dca010826	protein CELLULOSE SYNTHASE INTERACTIVE 3 [Dendrobium catenatum]
gene-MA16_Dca027730	hypothetical protein MA16_Dca027730 [Dendrobium catenatum]
gene-MA16_Dca011968	phosphoinositide phosphatase SAC2-like [Dendrobium catenatum]
gene-MA16_Dca015376	hypothetical protein MA16_Dca015376 [Dendrobium catenatum]
gene-MA16_Dca011782	CLP protease regulatory subunit CLPX1, mitochondrial-like isoform X1 [Dendrobium catenatum]
gene-MA16_Dca002029	hypothetical protein MA16_Dca002029 [Dendrobium catenatum]
gene-MA16_Dca011627	hypothetical protein MA16_Dca011627 [Dendrobium catenatum]
gene-MA16_Dca018453	hypothetical protein MA16_Dca018453 [Dendrobium catenatum]
gene-MA16_Dca026877	zinc finger protein BRUTUS-like [Dendrobium catenatum]
gene-MA16_Dca013924	putative disease resistance protein RGA3 [Dendrobium catenatum]
gene-MA16_Dca022831	Cytochrome b-c1 complex subunit Rieske, mitochondrial [Dendrobium catenatum]
gene-MA16_Dca026271	Receptor-like protein 12 [Dendrobium catenatum]
gene-MA16_Dca012102	BRI1 kinase inhibitor 1-like [Dendrobium catenatum]
gene-MA16_Dca005930	hypothetical protein MA16_Dca005930 [Dendrobium catenatum]
gene-MA16_Dca005479	exocyst complex component EXO84B-like [Dendrobium catenatum]
gene-MA16_Dca019222	Growth-regulating factor 1 [Dendrobium catenatum]

gene-MA16_Dca014123	LOB domain-containing protein 29 [Dendrobium catenatum]
gene-MA16_Dca002451	Cysteine-rich receptor-like protein kinase 42 [Dendrobium catenatum]
gene-MA16_Dca012461	pentatricopeptide repeat-containing protein At1g73400, mitochondrial [Dendrobium catenatum]
gene-MA16_Dca017815	3-dehydrosphinganine reductase TSC10A-like [Dendrobium catenatum]
gene-MA16_Dca024966	Mediator of RNA polymerase II transcription subunit 23 [Dendrobium catenatum]
gene-MA16_Dca010619	putative anion transporter 4, chloroplastic [Dendrobium catenatum]
gene-MA16_Dca006453	uncharacterized protein LOC110098806 isoform X2 [Dendrobium catenatum]
gene-MA16_Dca020020	LOB domain-containing protein 6-like [Dendrobium catenatum]
gene-MA16_Dca009122	Retrovirus-related Pol polyprotein from transposon TNT 1-94 [Dendrobium catenatum]
gene-MA16_Dca018409	probable protein phosphatase 2C 6 [Dendrobium catenatum]
gene-MA16_Dca015263	31 kDa ribonucleoprotein, chloroplastic [Dendrobium catenatum]
gene-MA16_Dca003077	protein SOMBRERO-like [Dendrobium catenatum]
gene-MA16_Dca009926	squamosa promoter-binding-like protein 12 isoform X1 [Dendrobium catenatum]
gene-MA16_Dca018009	hypothetical protein MA16_Dca018009 [Dendrobium catenatum]
gene-MA16_Dca023796	calcium-transporting ATPase 1, plasma membrane-type-like isoform X1 [Dendrobium catenatum]
gene-MA16_Dca023812	hypothetical protein MA16_Dca023812 [Dendrobium catenatum]
gene-MA16_Dca001249	lanC-like protein GCL2 [Dendrobium catenatum]
gene-MA16_Dca014208	hypothetical protein MA16_Dca014208 [Dendrobium catenatum]
gene-MA16_Dca018597	hypothetical protein MA16_Dca018597 [Dendrobium catenatum]
gene-MA16_Dca011616	Nuclear transcription factor Y subunit A-6 [Dendrobium catenatum]
gene-MA16_Dca022134	hypothetical protein MA16_Dca022134 [Dendrobium catenatum]
gene-MA16_Dca006230	uncharacterized protein LOC110111645 isoform X1 [Dendrobium catenatum]
gene-MA16_Dca029082	Cannabidiolic acid synthase [Dendrobium catenatum]
gene-MA16_Dca009393	hypothetical protein MA16_Dca009393 [Dendrobium catenatum]
gene-MA16_Dca024439	tubulin beta chain [Dendrobium catenatum]
gene-MA16_Dca026893	hypothetical protein MA16_Dca026893 [Dendrobium catenatum]
gene-MA16_Dca001659	U-box domain-containing protein 35-like isoform X1 [Dendrobium catenatum]
gene-MA16_Dca005939	hypothetical protein MA16_Dca005939 [Dendrobium catenatum]
gene-MA16_Dca011510	putative exocyst complex component 4 [Dendrobium catenatum]
gene-MA16_Dca006167	uncharacterized protein LOC110099950 [Dendrobium catenatum]

gene-MA16_Dca005089	hypothetical protein MA16_Dca005089 [Dendrobium catenatum]
gene-MA16_Dca023733	Retrovirus-related Pol polyprotein from transposon TNT 1-94 [Dendrobium catenatum]
gene-MA16_Dca008604	isoleucyl-tRNA synthetase [Dendrobium catenatum]
gene-MA16_Dca009173	Protein FAR1-RELATED SEQUENCE 5 [Dendrobium catenatum]
gene-MA16_Dca000014	UDP-glycosyltransferase 89B1 [Dendrobium catenatum]
gene-MA16_Dca000639	Serine/threonine-protein phosphatase PP1 [Dendrobium catenatum]
gene-MA16_Dca007171	Clustered mitochondria protein [Dendrobium catenatum]
gene-MA16_Dca015814	reticuline oxidase-like [Dendrobium catenatum]
gene-MA16_Dca023178	CASP-like protein [Dendrobium catenatum]
gene-MA16_Dca016806	universal stress protein PHOS32 [Dendrobium catenatum]
gene-MA16_Dca014097	zinc-finger homeodomain protein 4-like [Dendrobium catenatum]
gene-MA16_Dca016510	Potassium channel AKT6 [Dendrobium catenatum]
gene-MA16_Dca011314	hypothetical protein MA16_Dca011314 [Dendrobium catenatum]
gene-MA16_Dca018703	RNA-directed DNA polymerase [Dendrobium catenatum]
gene-MA16_Dca028665	hypothetical protein MA16_Dca028665 [Dendrobium catenatum]
gene-MA16_Dca002651	hypothetical protein MA16_Dca002651 [Dendrobium catenatum]
gene-MA16_Dca005730	Pantothenate kinase 2 [Dendrobium catenatum]
gene-MA16_Dca008222	ETHYLENE INSENSITIVE 3-like 1 protein [Dendrobium catenatum]
gene-MA16_Dca004998	probable WRKY transcription factor 2 isoform X1 [Dendrobium catenatum]
gene-MA16_Dca019218	probable serine/threonine-protein kinase PBL17 [Dendrobium catenatum]
gene-MA16_Dca027391	hypothetical protein MA16_Dca027391 [Dendrobium catenatum]
gene-MA16_Dca005512	uncharacterized protein LOC110095435 [Dendrobium catenatum]
gene-MA16_Dca022492	hypothetical protein MA16_Dca022492 [Dendrobium catenatum]
gene-MA16_Dca010295	Protein FAR1-RELATED SEQUENCE 5 [Dendrobium catenatum]
gene-MA16_Dca017059	zinc protease PQQL-like [Dendrobium catenatum]
gene-MA16_Dca008510	putative leucine-rich repeat receptor-like protein kinase [Dendrobium catenatum]
gene-MA16_Dca017007	ubiquitin-like domain-containing CTD phosphatase [Dendrobium catenatum]
gene-MA16_Dca002762	60S acidic ribosomal protein P0-2 [Dendrobium catenatum]
gene-MA16_Dca013211	putative nitrite transporter [Dendrobium catenatum]
gene-MA16_Dca025776	Cytochrome c oxidase subunit 6a, mitochondrial [Dendrobium catenatum]

gene-MA16_Dca002053	embryogenesis-associated protein EMB8-like [Dendrobium catenatum]
gene-MA16_Dca003771	hypothetical protein MA16_Dca003771 [Dendrobium catenatum]
gene-MA16_Dca017393	C-type lectin receptor-like tyrosine-protein kinase At1g52310 [Dendrobium catenatum]
gene-MA16_Dca015000	Protein SCARECROW [Dendrobium catenatum]
gene-MA16_Dca002893	hypothetical protein MA16_Dca002893 [Dendrobium catenatum]
gene-MA16_Dca027852	MADS-box transcription factor 21 [Dendrobium catenatum]
gene-MA16_Dca005787	Pentatricopeptide repeat-containing protein [Dendrobium catenatum]
gene-MA16_Dca010315	ribosome biogenesis protein WDR12 homolog isoform X1 [Dendrobium catenatum]
gene-MA16_Dca020908	Pentatricopeptide repeat-containing protein [Dendrobium catenatum]
gene-MA16_Dca012104	probable protein phosphatase 2C 12 [Dendrobium catenatum]
gene-MA16_Dca007408	hypothetical protein MA16_Dca007408 [Dendrobium catenatum]
gene-MA16_Dca002199	Disease resistance protein RGA2 [Dendrobium catenatum]
gene-MA16_Dca013949	hypothetical protein MA16_Dca013949 [Dendrobium catenatum]
gene-MA16_Dca018547	protein S-acyltransferase 18 [Dendrobium catenatum]
gene-MA16_Dca009428	Putative ataxin-3 like [Dendrobium catenatum]
gene-MA16_Dca013084	plant intracellular Ras-group-related LRR protein 3-like [Dendrobium catenatum]
gene-MA16_Dca007613	S-norcoclaurine synthase 1-like [Dendrobium catenatum]
gene-MA16_Dca004667	Helicase protein MOM1 [Dendrobium catenatum]
gene-MA16_Dca006907	Histidine--tRNA ligase [Dendrobium catenatum]
gene-MA16_Dca010206	hydroquinone glucosyltransferase-like [Dendrobium catenatum]
gene-MA16_Dca026264	uncharacterized protein LOC110115093 isoform X1 [Dendrobium catenatum]
gene-MA16_Dca024941	uncharacterized protein LOC110093954 [Dendrobium catenatum]
gene-MA16_Dca024581	hypothetical protein MA16_Dca024581 [Dendrobium catenatum]
gene-MA16_Dca017287	multisite-specific tRNA:(cytosine-C5)-methyltransferase [Dendrobium catenatum]
gene-MA16_Dca007605	formyltetrahydrofolate deformylase 2, mitochondrial-like isoform X2 [Dendrobium catenatum]
gene-MA16_Dca004723	Protein CCA1 [Dendrobium catenatum]
gene-MA16_Dca013578	125 kDa kinesin-related protein [Dendrobium catenatum]
gene-MA16_Dca005087	putative metal-nicotianamine transporter YSL9 [Dendrobium catenatum]
gene-MA16_Dca010354	protein IQ-DOMAIN 1-like [Dendrobium catenatum]
gene-MA16_Dca026923	transcription factor bHLH53-like [Dendrobium catenatum]

gene-MA16_Dca023756	hypothetical protein MA16_Dca023756 [Dendrobium catenatum]
gene-MA16_Dca005261	hypothetical protein MA16_Dca005261 [Dendrobium catenatum]
gene-MA16_Dca006515	elongation factor 2 [Dendrobium catenatum]
gene-MA16_Dca021294	hypothetical protein MA16_Dca021294 [Dendrobium catenatum]
gene-MA16_Dca023691	Anthranilate synthase component I-1, chloroplastic [Dendrobium catenatum]
gene-MA16_Dca006593	hypothetical protein MA16_Dca006593 [Dendrobium catenatum]
gene-MA16_Dca009467	methylthioribose-1-phosphate isomerase isoform X1 [Dendrobium catenatum]
gene-MA16_Dca013400	MAG2-interacting protein 2 isoform X2 [Dendrobium catenatum]
gene-MA16_Dca021828	Petal death protein [Dendrobium catenatum]
gene-MA16_Dca018795	hypothetical protein MA16_Dca018795 [Dendrobium catenatum]
gene-MA16_Dca019234	putative acyl-[acyl-carrier-protein]--UDP-N-acetylglucosamine O-acyltransferase, mitochondrial [Dendrobium catenatum]
gene-MA16_Dca001003	F-box protein At3g54460 isoform X1 [Dendrobium catenatum]
gene-MA16_Dca015057	pectinesterase-like [Dendrobium catenatum]
gene-MA16_Dca001580	CSC1-like protein At1g32090 [Dendrobium catenatum]
gene-MA16_Dca016369	protein CHROMATIN REMODELING 4-like [Dendrobium catenatum]
gene-MA16_Dca011246	TBC1 domain family member 15-like [Dendrobium catenatum]
gene-MA16_Dca020471	Indole-3-pyruvate monooxygenase YUCCA2 [Dendrobium catenatum]
gene-MA16_Dca016841	hypothetical protein MA16_Dca016841 [Dendrobium catenatum]
gene-MA16_Dca005406	hypothetical protein MA16_Dca005406 [Dendrobium catenatum]
gene-MA16_Dca001258	probable LRR receptor-like serine/threonine-protein kinase At1g34110 isoform X1 [Dendrobium catenatum]
gene-MA16_Dca022681	putative mitochondrial protein [Dendrobium catenatum]
gene-MA16_Dca004912	zinc finger CCCH domain-containing protein ZFN-like isoform X1 [Dendrobium catenatum]
gene-MA16_Dca012624	hypothetical protein MA16_Dca012624 [Dendrobium catenatum]
gene-MA16_Dca019395	osmotin-like protein [Dendrobium catenatum]
gene-MA16_Dca005574	syndetin isoform X1 [Dendrobium catenatum]
gene-MA16_Dca028781	hypothetical protein MA16_Dca028781 [Dendrobium catenatum]
gene-MA16_Dca006933	Retrovirus-related Pol polyprotein from transposon TNT 1-94 [Dendrobium catenatum]
gene-MA16_Dca016668	uncharacterized protein At1g10890-like [Dendrobium catenatum]
gene-MA16_Dca005761	transcription factor EGL1-like [Dendrobium catenatum]
gene-MA16_Dca022323	E3 ubiquitin-protein ligase SHPRH isoform X1 [Dendrobium catenatum]

gene-MA16_Dca011294	pentatricopeptide repeat-containing protein At1g71210 [Dendrobium catenatum]
gene-MA16_Dca000406	Disease resistance protein RGA2 [Dendrobium catenatum]
gene-MA16_Dca015968	elongator complex protein 1 [Dendrobium catenatum]
gene-MA16_Dca027828	Disease resistance protein RGA2 [Dendrobium catenatum]
gene-MA16_Dca006530	uncharacterized protein At2g40430 [Dendrobium catenatum]
gene-MA16_Dca008684	hypothetical protein MA16_Dca008684 [Dendrobium catenatum]
gene-MA16_Dca000222	hypothetical protein MA16_Dca000222 [Dendrobium catenatum]
gene-MA16_Dca013496	Purine permease 3 [Dendrobium catenatum]
gene-MA16_Dca000598	uncharacterized protein LOC110112334 [Dendrobium catenatum]
gene-MA16_Dca012306	pre-mRNA-splicing factor ATP-dependent RNA helicase DEAH10-like [Dendrobium catenatum]
gene-MA16_Dca005791	putative receptor-like protein kinase [Dendrobium catenatum]
gene-MA16_Dca020985	Putative ribonuclease H protein [Dendrobium catenatum]
gene-MA16_Dca025963	histone-lysine N-methyltransferase setd3 [Dendrobium catenatum]
gene-MA16_Dca001761	Retrovirus-related Pol polyprotein from transposon TNT 1-94 [Dendrobium catenatum]
gene-MA16_Dca017910	LOW QUALITY PROTEIN: pentatricopeptide repeat-containing protein At5g39710-like [Dendrobium catenatum]
gene-MA16_Dca018154	WUSCHEL-related homeobox 11 [Dendrobium catenatum]
gene-MA16_Dca009028	hypothetical protein MA16_Dca009028 [Dendrobium catenatum]
gene-MA16_Dca005517	Transcription factor GTE12 [Dendrobium catenatum]
gene-MA16_Dca017961	fibroblast growth factor receptor 1-like [Dendrobium catenatum]
gene-MA16_Dca001496	Lysine-specific histone demethylase 1 like 2 [Dendrobium catenatum]
gene-MA16_Dca008181	hypothetical protein MA16_Dca008181 [Dendrobium catenatum]
gene-MA16_Dca001379	NAC domain-containing protein 73-like [Dendrobium catenatum]
gene-MA16_Dca019567	Strictosidine synthase 1 [Dendrobium catenatum]
gene-MA16_Dca013734	F-box protein FBX14 [Dendrobium catenatum]
gene-MA16_Dca019290	hypothetical protein MA16_Dca019290 [Dendrobium catenatum]
gene-MA16_Dca012297	hypothetical protein MA16_Dca012297 [Dendrobium catenatum]
gene-MA16_Dca008674	Histone-lysine N-methyltransferase ASHH1 [Dendrobium catenatum]
gene-MA16_Dca011171	hypothetical protein MA16_Dca011171 [Dendrobium catenatum]
gene-MA16_Dca007639	hypothetical protein MA16_Dca007639 [Dendrobium catenatum]
gene-MA16_Dca000668	sorting nexin 2B-like isoform X2 [Dendrobium catenatum]

gene-MA16_Dca020389	Uncharacterized protein MA16_Dca020389 [Dendrobium catenatum]
gene-MA16_Dca011457	probable apyrase 1 isoform X2 [Dendrobium catenatum]
gene-MA16_Dca012047	CST complex subunit CTC1 isoform X1 [Dendrobium catenatum]
gene-MA16_Dca002587	NAC transcription factor 47-like [Dendrobium catenatum]
gene-MA16_Dca026145	uncharacterized protein LOC110109109 [Dendrobium catenatum]
gene-MA16_Dca014751	limonoid UDP-glucosyltransferase-like [Dendrobium catenatum]
gene-MA16_Dca001901	ubiquitin carboxyl-terminal hydrolase 24 [Dendrobium catenatum]
gene-MA16_Dca023413	auxin response factor 7-like [Dendrobium catenatum]
gene-MA16_Dca022435	dehydrodolichyl diphosphate synthase 2-like [Dendrobium catenatum]
gene-MA16_Dca002770	RNA exonuclease 1 [Dendrobium catenatum]
gene-MA16_Dca025836	putative mitochondrial protein [Dendrobium catenatum]
gene-MA16_Dca001016	probable calcium-binding protein CML10 [Dendrobium catenatum]
gene-MA16_Dca018282	Pentatricopeptide repeat-containing protein [Dendrobium catenatum]
gene-MA16_Dca016085	phosphoenolpyruvate/phosphate translocator 2, chloroplastic [Dendrobium catenatum]
gene-MA16_Dca026118	hypothetical protein MA16_Dca026118 [Dendrobium catenatum]
gene-MA16_Dca001815	hypothetical protein MA16_Dca001815 [Dendrobium catenatum]
gene-MA16_Dca000408	Putative disease resistance protein RGA4 [Dendrobium catenatum]
gene-MA16_Dca003012	uncharacterized protein LOC110106860 isoform X2 [Dendrobium catenatum]
gene-MA16_Dca022315	hypothetical protein MA16_Dca022315 [Dendrobium catenatum]
gene-MA16_Dca019975	probable serine/threonine-protein kinase DDB_G0276461 isoform X2 [Dendrobium catenatum]
gene-MA16_Dca011459	putative leucine-rich repeat receptor-like protein kinase [Dendrobium catenatum]
gene-MA16_Dca012305	Chromatin structure-remodeling complex protein SYD [Dendrobium catenatum]
gene-MA16_Dca003212	uncharacterized protein LOC110113433 [Dendrobium catenatum]
gene-MA16_Dca026111	hypothetical protein MA16_Dca026111 [Dendrobium catenatum]
gene-MA16_Dca023054	hypothetical protein MA16_Dca023054 [Dendrobium catenatum]
gene-MA16_Dca010217	heat stress transcription factor A-4b-like [Dendrobium catenatum]
gene-MA16_Dca028025	60S acidic ribosomal protein P0-3 [Dendrobium catenatum]
gene-MA16_Dca021782	Peptide methionine sulfoxide reductase B1, chloroplastic [Dendrobium catenatum]
gene-MA16_Dca012371	hypothetical protein MA16_Dca012371 [Dendrobium catenatum]
gene-MA16_Dca012093	ABSCISIC ACID-INSENSITIVE 5-like protein 5 [Dendrobium catenatum]

gene-MA16_Dca028017	hypothetical protein MA16_Dca028017 [Dendrobium catenatum]
gene-MA16_Dca026494	Acyl carrier protein 2, mitochondrial [Dendrobium catenatum]
gene-MA16_Dca018025	endoribonuclease Dicer homolog 1 [Dendrobium catenatum]
gene-MA16_Dca002897	U2 small nuclear ribonucleoprotein B'' [Dendrobium catenatum]
gene-MA16_Dca024985	Uncharacterized protein MA16_Dca024985 [Dendrobium catenatum]
gene-MA16_Dca011813	E3 ubiquitin-protein ligase BRE1-like 1 [Dendrobium catenatum]
gene-MA16_Dca025882	hypothetical protein MA16_Dca025882 [Dendrobium catenatum]
gene-MA16_Dca019871	hypothetical protein MA16_Dca019871 [Dendrobium catenatum]
gene-MA16_Dca016917	cinnamoyl-CoA reductase-like SNL6 [Dendrobium catenatum]
gene-MA16_Dca017621	Gibberellin 2-beta-dioxygenase [Dendrobium catenatum]
gene-MA16_Dca009084	uncharacterized protein LOC110115781 [Dendrobium catenatum]
gene-MA16_Dca009790	Putative disease resistance protein RGA1 [Dendrobium catenatum]
gene-MA16_Dca027946	hypothetical protein MA16_Dca027946 [Dendrobium catenatum]
gene-MA16_Dca028230	hypothetical protein MA16_Dca028230 [Dendrobium catenatum]
gene-MA16_Dca002276	uncharacterized protein LOC110104350 [Dendrobium catenatum]
gene-MA16_Dca003052	Retrovirus-related Pol polyprotein from transposon TNT 1-94 [Dendrobium catenatum]
gene-MA16_Dca015047	steroid 5-alpha-reductase DET2 isoform X1 [Dendrobium catenatum]
gene-MA16_Dca019707	Retrovirus-related Pol polyprotein from transposon TNT 1-94 [Dendrobium catenatum]
gene-MA16_Dca020272	putative E3 ubiquitin-protein ligase ARI9 [Dendrobium catenatum]
gene-MA16_Dca022787	hypothetical protein MA16_Dca022787 [Dendrobium catenatum]
gene-MA16_Dca020204	uncharacterized protein LOC110105715 isoform X3 [Dendrobium catenatum]
gene-MA16_Dca002020	mitogen-activated protein kinase kinase 6-like [Dendrobium catenatum]
gene-MA16_Dca028002	Retrovirus-related Pol polyprotein from transposon TNT 1-94 [Dendrobium catenatum]
gene-MA16_Dca009622	Retrovirus-related Pol polyprotein from transposon TNT 1-94 [Dendrobium catenatum]
gene-MA16_Dca001290	protein NETWORKED 1D-like [Dendrobium catenatum]
gene-MA16_Dca021141	Protein NSP-INTERACTING KINASE 1 [Dendrobium catenatum]
gene-MA16_Dca011698	E3 ubiquitin-protein ligase MARCH6 [Dendrobium catenatum]
gene-MA16_Dca024989	ESF1 homolog [Dendrobium catenatum]
gene-MA16_Dca000275	hypothetical protein MA16_Dca000275 [Dendrobium catenatum]
gene-MA16_Dca015884	Auxin response factor 12 [Dendrobium catenatum]

gene-MA16_Dca022075	Retrovirus-related Pol polyprotein from transposon TNT 1-94 [Dendrobium catenatum]
gene-MA16_Dca016023	protein NRT1/ PTR FAMILY 7.2-like isoform X1 [Dendrobium catenatum]
gene-MA16_Dca005206	hypothetical protein MA16_Dca005206 [Dendrobium catenatum]
gene-MA16_Dca013489	E3 ubiquitin-protein ligase ATL31-like [Dendrobium catenatum]
gene-MA16_Dca007576	LRR repeats and ubiquitin-like domain-containing protein [Dendrobium catenatum]
gene-MA16_Dca004513	putative sulfate transporter 3.4 [Dendrobium catenatum]
gene-MA16_Dca008220	putative LRR receptor-like serine/threonine-protein kinase [Dendrobium catenatum]
gene-MA16_Dca022401	Putative ribonuclease H protein [Dendrobium catenatum]
gene-MA16_Dca022271	glutamate decarboxylase-like [Dendrobium catenatum]
gene-MA16_Dca013322	Actin-depolymerizing factor [Dendrobium catenatum]
gene-MA16_Dca014450	acetylornithine aminotransferase, mitochondrial [Dendrobium catenatum]
gene-MA16_Dca015731	transcriptional activator DEMETER-like isoform X1 [Dendrobium catenatum]
gene-MA16_Dca010708	Aluminum-activated malate transporter 10 [Dendrobium catenatum]
gene-MA16_Dca023672	E3 ubiquitin-protein ligase RFWD3-like isoform X2 [Dendrobium catenatum]
gene-MA16_Dca024729	putative pentatricopeptide repeat-containing protein At1g19290 [Dendrobium catenatum]
gene-MA16_Dca019401	protein argonaute 1A-like [Dendrobium catenatum]
gene-MA16_Dca005860	Putative protease Do-like 14 [Dendrobium catenatum]
gene-MA16_Dca003477	disease resistance protein RGA2-like [Dendrobium catenatum]
gene-MA16_Dca000260	Uncharacterized protein MA16_Dca000260 [Dendrobium catenatum]
gene-MA16_Dca022668	vesicle-associated membrane protein 724-like [Dendrobium catenatum]
gene-MA16_Dca013080	Retrovirus-related Pol polyprotein from transposon TNT 1-94 [Dendrobium catenatum]
gene-MA16_Dca018108	vacuolar protein sorting-associated protein 41 homolog isoform X1 [Dendrobium catenatum]
gene-MA16_Dca022795	pyrophosphate--fructose 6-phosphate 1-phosphotransferase subunit beta-like [Dendrobium catenatum]
gene-MA16_Dca003956	hypothetical protein MA16_Dca003956 [Dendrobium catenatum]
gene-MA16_Dca019882	protein TPLATE [Dendrobium catenatum]
gene-MA16_Dca018979	uncharacterized protein LOC110105902 isoform X1 [Dendrobium catenatum]
gene-MA16_Dca011983	Alpha-aminoadipic semialdehyde synthase [Dendrobium catenatum]
gene-MA16_Dca009131	transmembrane 9 superfamily member 7-like [Dendrobium catenatum]
gene-MA16_Dca018928	Myb family transcription factor APL [Dendrobium catenatum]
gene-MA16_Dca000871	Peptidyl-prolyl cis-trans isomerase CYP19-4 [Dendrobium catenatum]

gene-MA16_Dca004695	60S ribosomal protein L13a-4 [Dendrobium catenatum]
gene-MA16_Dca012938	hypothetical protein MA16_Dca012938 [Dendrobium catenatum]
gene-MA16_Dca003354	hypothetical protein MA16_Dca003354 [Dendrobium catenatum]
gene-MA16_Dca016890	COP9 signalosome complex subunit 8 isoform X2 [Dendrobium catenatum]
gene-MA16_Dca020253	Rho GDP-dissociation inhibitor 1 [Dendrobium catenatum]
gene-MA16_Dca014098	Retrovirus-related Pol polyprotein from transposon TNT 1-94 [Dendrobium catenatum]
gene-MA16_Dca020190	hypothetical protein MA16_Dca020190 [Dendrobium catenatum]
gene-MA16_Dca018646	pentatricopeptide repeat-containing protein At5g15980, mitochondrial-like [Dendrobium catenatum]
gene-MA16_Dca022711	glutamate receptor 3.7-like [Dendrobium catenatum]
gene-MA16_Dca022915	Putative magnesium transporter MRS2-G [Dendrobium catenatum]
gene-MA16_Dca014007	Protein TORNADO 1 [Dendrobium catenatum]
gene-MA16_Dca011727	Cytochrome P450 71D10 [Dendrobium catenatum]
gene-MA16_Dca024716	aluminum-activated malate transporter 12-like [Dendrobium catenatum]
gene-MA16_Dca022465	hypothetical protein MA16_Dca022465 [Dendrobium catenatum]
gene-MA16_Dca020080	hypothetical protein MA16_Dca014216 [Dendrobium catenatum]
gene-MA16_Dca015877	hypothetical protein MA16_Dca015877 [Dendrobium catenatum]
gene-MA16_Dca016418	transcription factor SRM1 [Dendrobium catenatum]
gene-MA16_Dca009144	squalene monooxygenase-like [Dendrobium catenatum]
gene-MA16_Dca017141	hypothetical protein MA16_Dca017141 [Dendrobium catenatum]
gene-MA16_Dca020137	NDR1/HIN1-like protein 13 [Dendrobium catenatum]
gene-MA16_Dca008336	uncharacterized protein LOC110093430 [Dendrobium catenatum]
gene-MA16_Dca014115	hypothetical protein MA16_Dca014115 [Dendrobium catenatum]
gene-MA16_Dca001590	cellulose synthase-like protein D2 [Dendrobium catenatum]
gene-MA16_Dca006086	hypothetical protein MA16_Dca006086 [Dendrobium catenatum]
gene-MA16_Dca016983	Retrovirus-related Pol polyprotein from transposon TNT 1-94 [Dendrobium catenatum]
gene-MA16_Dca019502	uncharacterized protein At4g18257-like [Dendrobium catenatum]
gene-MA16_Dca028733	putative peptide/nitrate transporter [Dendrobium catenatum]
gene-MA16_Dca028823	hypothetical protein MA16_Dca028823 [Dendrobium catenatum]
gene-MA16_Dca012579	small nuclear ribonucleoprotein Sm D1-like [Dendrobium catenatum]
gene-MA16_Dca025782	pentatricopeptide repeat-containing protein At4g16835, mitochondrial [Dendrobium catenatum]

gene-MA16_Dca007881	probable protein arginine N-methyltransferase 3 [Dendrobium catenatum]
gene-MA16_Dca005778	Chloride channel protein CLC-f [Dendrobium catenatum]
gene-MA16_Dca011418	Protein kinase APK1A, chloroplastic [Dendrobium catenatum]
gene-MA16_Dca015527	uncharacterized protein LOC110096417 isoform X2 [Dendrobium catenatum]
gene-MA16_Dca002954	zinc finger protein ZAT12-like [Dendrobium catenatum]
gene-MA16_Dca015045	fanconi-associated nuclease 1 homolog isoform X1 [Dendrobium catenatum]
gene-MA16_Dca001294	Single-stranded DNA-binding protein WHY2, mitochondrial [Dendrobium catenatum]
gene-MA16_Dca002633	homeobox-leucine zipper protein HOX32-like [Dendrobium catenatum]
gene-MA16_Dca000832	potassium transporter 7 isoform X1 [Dendrobium catenatum]
gene-MA16_Dca002787	Serine/threonine-protein kinase PBS1 [Dendrobium catenatum]
gene-MA16_Dca008935	ataxin-10 isoform X1 [Dendrobium catenatum]
gene-MA16_Dca008326	probable protein S-acyltransferase 7 isoform X1 [Dendrobium catenatum]
gene-MA16_Dca018766	hypothetical protein MA16_Dca018766 [Dendrobium catenatum]
gene-MA16_Dca003398	hypothetical protein MA16_Dca003398 [Dendrobium catenatum]
gene-MA16_Dca016163	ferric reduction oxidase 2-like isoform X2 [Dendrobium catenatum]
gene-MA16_Dca004765	Luminal-binding protein [Dendrobium catenatum]
gene-MA16_Dca005170	probable protein S-acyltransferase 1 [Dendrobium catenatum]
gene-MA16_Dca003489	putative H/ACA ribonucleoprotein complex subunit 1-like protein 1 [Dendrobium catenatum]
gene-MA16_Dca013643	putative glutamyl endopeptidase, chloroplastic [Dendrobium catenatum]
gene-MA16_Dca002996	hypothetical protein MA16_Dca002996 [Dendrobium catenatum]
gene-MA16_Dca021058	Cellulose synthase-like protein E2 [Dendrobium catenatum]
gene-MA16_Dca012296	U-box domain-containing protein 10-like isoform X1 [Dendrobium catenatum]
gene-MA16_Dca020944	exopolygalacturonase isoform X1 [Dendrobium catenatum]
gene-MA16_Dca020671	hypothetical protein MA16_Dca020671 [Dendrobium catenatum]
gene-MA16_Dca009506	hypothetical protein MA16_Dca009506 [Dendrobium catenatum]
gene-MA16_Dca000065	Uncharacterized protein MA16_Dca000065 [Dendrobium catenatum]
gene-MA16_Dca011329	hypothetical protein MA16_Dca011329 [Dendrobium catenatum]
gene-MA16_Dca006805	hypothetical protein MA16_Dca006805 [Dendrobium catenatum]
gene-MA16_Dca023035	hypothetical protein MA16_Dca023035 [Dendrobium catenatum]
gene-MA16_Dca006305	squamosa promoter-binding-like protein 12 [Dendrobium catenatum]

gene-MA16_Dca001135	putative serine/threonine-protein kinase-like protein CCR3 [Dendrobium catenatum]
gene-MA16_Dca007943	Diacylglycerol O-acyltransferase 2 [Dendrobium catenatum]
gene-MA16_Dca022385	Protein RALF-like 33 [Dendrobium catenatum]
gene-MA16_Dca003664	probable 2-oxoglutarate-dependent dioxygenase AOP1 [Dendrobium catenatum]
gene-MA16_Dca025505	uncharacterized protein LOC110103003 [Dendrobium catenatum]
gene-MA16_Dca019094	elongation factor G, mitochondrial-like [Dendrobium catenatum]
gene-MA16_Dca007249	RNA-directed DNA polymerase [Dendrobium catenatum]
gene-MA16_Dca018337	uncharacterized protein LOC110105771 [Dendrobium catenatum]
gene-MA16_Dca004766	hypothetical protein MA16_Dca004766 [Dendrobium catenatum]
gene-MA16_Dca017511	hypothetical protein MA16_Dca017511 [Dendrobium catenatum]
gene-MA16_Dca024771	hypothetical protein MA16_Dca024771 [Dendrobium catenatum]
gene-MA16_Dca013145	primary amine oxidase 1-like [Dendrobium catenatum]
gene-MA16_Dca014915	hypothetical protein MA16_Dca014915 [Dendrobium catenatum]
gene-MA16_Dca004712	AT-rich interactive domain-containing protein 1A-like isoform X1 [Dendrobium catenatum]
gene-MA16_Dca026781	Retrovirus-related Pol polyprotein from transposon TNT 1-94 [Dendrobium catenatum]
gene-MA16_Dca006168	Retrovirus-related Pol polyprotein from transposon TNT 1-94 [Dendrobium catenatum]
gene-MA16_Dca012265	glutamate receptor 3.4-like [Dendrobium catenatum]
gene-MA16_Dca006830	hypothetical protein MA16_Dca006830 [Dendrobium catenatum]
gene-MA16_Dca016013	abscisic acid 8'-hydroxylase 3-like [Dendrobium catenatum]
gene-MA16_Dca024728	Folate transporter 1, chloroplastic [Dendrobium catenatum]
gene-MA16_Dca024831	hypothetical protein MA16_Dca024831 [Dendrobium catenatum]
gene-MA16_Dca021885	hypothetical protein MA16_Dca021885 [Dendrobium catenatum]
gene-MA16_Dca007838	hypothetical protein MA16_Dca007838 [Dendrobium catenatum]
gene-MA16_Dca024385	receptor protein kinase TMK1-like [Dendrobium catenatum]
gene-MA16_Dca013632	hypothetical protein MA16_Dca013632 [Dendrobium catenatum]
gene-MA16_Dca002466	hypothetical protein MA16_Dca002466 [Dendrobium catenatum]
gene-MA16_Dca013914	Retrovirus-related Pol polyprotein from transposon TNT 1-94 [Dendrobium catenatum]
gene-MA16_Dca000976	hypothetical protein MA16_Dca000976 [Dendrobium catenatum]
gene-MA16_Dca006507	heterogeneous nuclear ribonucleoprotein 1-like [Dendrobium catenatum]
gene-MA16_Dca000965	long chain acyl-CoA synthetase 2 [Dendrobium catenatum]

gene-MA16_Dca028338	putative fatty acyl-CoA reductase 4 [Dendrobium catenatum]
gene-MA16_Dca006821	phytochrome A-like [Dendrobium catenatum]
gene-MA16_Dca023637	QWRF motif-containing protein 2-like [Dendrobium catenatum]
gene-MA16_Dca000773	putative disease resistance protein RGA3 [Dendrobium catenatum]
gene-MA16_Dca013389	Uncharacterized protein MA16_Dca013389 [Dendrobium catenatum]
gene-MA16_Dca011639	U-box domain-containing protein 21-like [Dendrobium catenatum]
gene-MA16_Dca007213	WAT1-related protein At5g45370-like isoform X1 [Dendrobium catenatum]
gene-MA16_Dca012781	protein argonaute 7-like [Dendrobium catenatum]
gene-MA16_Dca007439	homeobox-leucine zipper protein HOX6-like [Dendrobium catenatum]
gene-MA16_Dca003930	GATA transcription factor 26-like [Dendrobium catenatum]
gene-MA16_Dca006944	AT-rich interactive domain-containing protein 2 [Dendrobium catenatum]
gene-MA16_Dca014228	QWRF motif-containing protein 2-like [Dendrobium catenatum]
gene-MA16_Dca003014	Putative ribonuclease H protein [Dendrobium catenatum]
gene-MA16_Dca023002	Zeaxanthin epoxidase, chloroplastic [Dendrobium catenatum]
gene-MA16_Dca025223	putative pentatricopeptide repeat-containing protein At1g53330 [Dendrobium catenatum]
gene-MA16_Dca017400	protein TRANSPORT INHIBITOR RESPONSE 1-like [Dendrobium catenatum]
gene-MA16_Dca011751	hypothetical protein MA16_Dca011751 [Dendrobium catenatum]
gene-MA16_Dca021860	Growth-regulating factor 6 [Dendrobium catenatum]
gene-MA16_Dca021261	hypothetical protein MA16_Dca021261 [Dendrobium catenatum]
gene-MA16_Dca022511	GDP-L-galactose phosphorylase 1-like [Dendrobium catenatum]
gene-MA16_Dca025135	heme-binding protein 2-like [Dendrobium catenatum]
gene-MA16_Dca021268	uncharacterized protein LOC110102257 isoform X1 [Dendrobium catenatum]
gene-MA16_Dca016827	Flap endonuclease 1-A [Dendrobium catenatum]
gene-MA16_Dca007637	ABC transporter I family member 6, chloroplastic [Dendrobium catenatum]
gene-MA16_Dca011207	ubiquitin-conjugating enzyme E2 4-like [Dendrobium catenatum]
gene-MA16_Dca010161	hypothetical protein MA16_Dca010161 [Dendrobium catenatum]
gene-MA16_Dca001881	hypothetical protein MA16_Dca001881 [Dendrobium catenatum]
gene-MA16_Dca008446	hypothetical protein MA16_Dca008446 [Dendrobium catenatum]
gene-MA16_Dca022279	neo-calmodulin-like [Dendrobium catenatum]
gene-MA16_Dca019561	uncharacterized protein LOC110110421 isoform X1 [Dendrobium catenatum]

gene-MA16_Dca021043	probable serine/threonine protein kinase IRE [Dendrobium catenatum]
gene-MA16_Dca026853	Photosystem II stability/assembly factor HCF136, chloroplastic [Dendrobium catenatum]
gene-MA16_Dca004167	PAN domain-containing protein At5g03700 [Dendrobium catenatum]
gene-MA16_Dca005616	transmembrane protein 184B isoform X1 [Dendrobium catenatum]
gene-MA16_Dca025817	protein ROS1-like isoform X1 [Dendrobium catenatum]
gene-MA16_Dca001948	hypothetical protein MA16_Dca001948 [Dendrobium catenatum]
gene-MA16_Dca015498	UPF0481 protein [Dendrobium catenatum]
gene-MA16_Dca011860	uncharacterized protein LOC110112786 [Dendrobium catenatum]
gene-MA16_Dca020888	Alcohol dehydrogenase 3 [Dendrobium catenatum]
gene-MA16_Dca015281	Putative UDP-rhamnose:rhamnosyltransferase 1 [Dendrobium catenatum]
gene-MA16_Dca001581	4-coumarate--CoA ligase-like 6 [Dendrobium catenatum]
gene-MA16_Dca021654	hypothetical protein MA16_Dca021654 [Dendrobium catenatum]
gene-MA16_Dca001269	6-phosphofructokinase 1 [Dendrobium catenatum]
gene-MA16_Dca023196	protein SPIRRIG [Dendrobium catenatum]
gene-MA16_Dca002952	hypothetical protein MA16_Dca002952 [Dendrobium catenatum]
gene-MA16_Dca016409	lysine-specific demethylase REF6 isoform X1 [Dendrobium catenatum]
gene-MA16_Dca002894	beta-galactosidase 2-like isoform X1 [Dendrobium catenatum]
gene-MA16_Dca015394	putative serine/threonine-protein kinase [Dendrobium catenatum]
gene-MA16_Dca013684	Retrovirus-related Pol polyprotein from transposon TNT 1-94 [Dendrobium catenatum]
gene-MA16_Dca004139	Protein FAR1-RELATED SEQUENCE 5 [Dendrobium catenatum]
gene-MA16_Dca000144	malignant T-cell-amplified sequence 1 homolog [Dendrobium catenatum]
gene-MA16_Dca010066	hypothetical protein MA16_Dca010066 [Dendrobium catenatum]
gene-MA16_Dca003504	hypothetical protein MA16_Dca003504 [Dendrobium catenatum]
gene-MA16_Dca002593	cation/H(+) antiporter 15-like [Dendrobium catenatum]
gene-MA16_Dca001128	DNA (cytosine-5)-methyltransferase 1B-like [Dendrobium catenatum]
gene-MA16_Dca004204	Retrovirus-related Pol polyprotein from transposon TNT 1-94 [Dendrobium catenatum]
gene-MA16_Dca029169	hypothetical protein MA16_Dca029169 [Dendrobium catenatum]
gene-MA16_Dca017451	autophagy-related protein 18a [Dendrobium catenatum]
gene-MA16_Dca009576	hypothetical protein MA16_Dca009576 [Dendrobium catenatum]
gene-MA16_Dca019065	U-box domain-containing protein 44-like [Dendrobium catenatum]

gene-MA16_Dca019795	hypothetical protein MA16_Dca019795 [Dendrobium catenatum]
gene-MA16_Dca018194	probable 26S proteasome non-ATPase regulatory subunit 3 [Dendrobium catenatum]
gene-MA16_Dca022957	hypothetical protein MA16_Dca022957 [Dendrobium catenatum]
gene-MA16_Dca027198	G-type lectin S-receptor-like serine/threonine-protein kinase SD2-5 [Dendrobium catenatum]
gene-MA16_Dca012806	Monogalactosyldiacylglycerol synthase, chloroplastic [Dendrobium catenatum]
gene-MA16_Dca011824	Homeobox-leucine zipper protein HOX32 [Dendrobium catenatum]
gene-MA16_Dca019600	Aspartic proteinase nepenthesin-1 [Dendrobium catenatum]
gene-MA16_Dca005943	hypothetical protein MA16_Dca005943 [Dendrobium catenatum]
gene-MA16_Dca003058	sodium/hydrogen exchanger 2-like [Dendrobium catenatum]
gene-MA16_Dca002439	hypothetical protein MA16_Dca002439 [Dendrobium catenatum]
gene-MA16_Dca004547	hypothetical protein MA16_Dca004547 [Dendrobium catenatum]
gene-MA16_Dca014409	uncharacterized protein LOC110110813 [Dendrobium catenatum]
gene-MA16_Dca015648	Leucine-rich repeat receptor-like serine/threonine-protein kinase [Dendrobium catenatum]
gene-MA16_Dca003173	RNA polymerase II C-terminal domain phosphatase-like 3 isoform X1 [Dendrobium catenatum]
gene-MA16_Dca003551	probable beta-D-xylosidase 7 [Dendrobium catenatum]
gene-MA16_Dca021211	non-specific lipid-transfer protein-like protein At2g13820 [Dendrobium catenatum]
gene-MA16_Dca026931	DNA repair protein UVH3 isoform X2 [Dendrobium catenatum]
gene-MA16_Dca013243	exosome complex exonuclease RRP44 homolog A-like [Dendrobium catenatum]
gene-MA16_Dca026199	hypothetical protein MA16_Dca026199 [Dendrobium catenatum]
gene-MA16_Dca023407	hypothetical protein MA16_Dca023407 [Dendrobium catenatum]
gene-MA16_Dca017679	RING-H2 finger protein ATL70 [Dendrobium catenatum]
gene-MA16_Dca003197	hypothetical protein MA16_Dca003197 [Dendrobium catenatum]
gene-MA16_Dca013233	protein N-lysine methyltransferase METTL21A isoform X2 [Dendrobium catenatum]
gene-MA16_Dca015397	GDSL esterase/lipase [Dendrobium catenatum]
gene-MA16_Dca011747	hypothetical protein MA16_Dca011747 [Dendrobium catenatum]
gene-MA16_Dca010679	Scarecrow-like protein 6 [Dendrobium catenatum]
gene-MA16_Dca014230	uncharacterized protein LOC110096149 isoform X2 [Dendrobium catenatum]
gene-MA16_Dca005107	BEACH domain-containing protein C2-like isoform X1 [Dendrobium catenatum]
gene-MA16_Dca014245	putative LRR receptor-like serine/threonine-protein kinase [Dendrobium catenatum]
gene-MA16_Dca015359	origin of replication complex subunit 2 [Dendrobium catenatum]

gene-MA16_Dca023376	NEDD8-conjugating enzyme Ubc12-like [Dendrobium catenatum]
gene-MA16_Dca003010	probable galacturonosyltransferase 11 [Dendrobium catenatum]
gene-MA16_Dca010724	hypothetical protein MA16_Dca010724 [Dendrobium catenatum]
gene-MA16_Dca007376	sugar transporter ERD6-like 6 [Dendrobium catenatum]
gene-MA16_Dca024348	MADS-box transcription factor 22-like [Dendrobium catenatum]
gene-MA16_Dca004721	hypothetical protein MA16_Dca004721 [Dendrobium catenatum]
gene-MA16_Dca020870	uncharacterized protein LOC110095700 [Dendrobium catenatum]
gene-MA16_Dca013127	hypothetical protein MA16_Dca013127 [Dendrobium catenatum]
gene-MA16_Dca016611	synaptotagmin-2 [Dendrobium catenatum]
gene-MA16_Dca007799	xyloglucan galactosyltransferase KATAMARI1 homolog [Dendrobium catenatum]
gene-MA16_Dca009549	40S ribosomal protein S5-1 [Dendrobium catenatum]
gene-MA16_Dca008195	pectinesterase QRT1-like [Dendrobium catenatum]
gene-MA16_Dca008944	putative mitochondrial protein [Dendrobium catenatum]
gene-MA16_Dca010685	uncharacterized protein LOC110110571 [Dendrobium catenatum]
gene-MA16_Dca022643	cysteine-rich receptor-like protein kinase 2 [Dendrobium catenatum]
gene-MA16_Dca023418	NADPH-dependent pterin aldehyde reductase [Dendrobium catenatum]
gene-MA16_Dca014865	hypothetical protein MA16_Dca014865 [Dendrobium catenatum]
gene-MA16_Dca004920	uncharacterized protein At4g29660 [Dendrobium catenatum]
gene-MA16_Dca009701	Leucine-rich repeat receptor-like protein kinase PEPR1 [Dendrobium catenatum]
gene-MA16_Dca024901	Putative disease resistance protein RGA4 [Dendrobium catenatum]
gene-MA16_Dca003261	Pentatricopeptide repeat-containing protein [Dendrobium catenatum]
gene-MA16_Dca003721	Putative ribonuclease H protein [Dendrobium catenatum]
gene-MA16_Dca020488	hypothetical protein MA16_Dca020488 [Dendrobium catenatum]
gene-MA16_Dca007957	Protein ROOT HAIR DEFECTIVE 3 [Dendrobium catenatum]
gene-MA16_Dca022520	Kinesin-like protein KIN12B [Dendrobium catenatum]
gene-MA16_Dca006221	hypothetical protein MA16_Dca006221 [Dendrobium catenatum]
gene-MA16_Dca007756	hypothetical protein MA16_Dca007756 [Dendrobium catenatum]
gene-MA16_Dca025344	5'-3' exoribonuclease 2 [Dendrobium catenatum]
gene-MA16_Dca026005	Putative ribonuclease H protein [Dendrobium catenatum]
gene-MA16_Dca007050	BEL1-like homeodomain protein 1 [Dendrobium catenatum]

gene-MA16_Dca015741	hydroquinone glucosyltransferase-like [Dendrobium catenatum]
gene-MA16_Dca002781	hypothetical protein MA16_Dca002781 [Dendrobium catenatum]
gene-MA16_Dca021252	hypothetical protein MA16_Dca021252 [Dendrobium catenatum]
gene-MA16_Dca019763	pentatricopeptide repeat-containing protein At1g09220, mitochondrial [Dendrobium catenatum]
gene-MA16_Dca001340	polygalacturonase inhibitor 2-like [Dendrobium catenatum]
gene-MA16_Dca028435	hypothetical protein MA16_Dca028435 [Dendrobium catenatum]
gene-MA16_Dca022325	probable LRR receptor-like serine/threonine-protein kinase IRK [Dendrobium catenatum]
gene-MA16_Dca024381	uncharacterized protein At5g49945 [Dendrobium catenatum]
gene-MA16_Dca003059	tubby-like F-box protein 6 [Dendrobium catenatum]
gene-MA16_Dca019839	Ras-related protein Rab7 [Dendrobium catenatum]
gene-MA16_Dca010639	elicitor-responsive protein 1 isoform X2 [Dendrobium catenatum]
gene-MA16_Dca003017	snurportin-1 isoform X1 [Dendrobium catenatum]
gene-MA16_Dca010674	Putative glucuronosyltransferase PGSIP7 [Dendrobium catenatum]
gene-MA16_Dca016599	GDSL esterase/lipase EXL3 [Dendrobium catenatum]
gene-MA16_Dca028164	hypothetical protein MA16_Dca028164 [Dendrobium catenatum]
gene-MA16_Dca004160	6-phosphofructokinase 3 [Dendrobium catenatum]
gene-MA16_Dca020264	putative mitochondrial protein [Dendrobium catenatum]
gene-MA16_Dca028562	Leucine-rich repeat receptor-like serine/threonine-protein kinase BAM2 [Dendrobium catenatum]
gene-MA16_Dca015708	hypothetical protein MA16_Dca015708 [Dendrobium catenatum]
gene-MA16_Dca002744	NAC domain-containing protein 92-like [Dendrobium catenatum]
gene-MA16_Dca024228	UPF0678 fatty acid-binding protein-like protein At1g79260 [Dendrobium catenatum]
gene-MA16_Dca022487	uncharacterized protein LOC110102400 isoform X1 [Dendrobium catenatum]
gene-MA16_Dca018513	putative mannan synthase 4 [Dendrobium catenatum]
gene-MA16_Dca021241	myosin-6-like [Dendrobium catenatum]
gene-MA16_Dca006854	putative folate-biopterin transporter 2 [Dendrobium catenatum]
gene-MA16_Dca023833	protoporphyrinogen oxidase, mitochondrial isoform X2 [Dendrobium catenatum]
gene-MA16_Dca024982	hypothetical protein MA16_Dca024982 [Dendrobium catenatum]
gene-MA16_Dca004038	Protein argonaute 1A [Dendrobium catenatum]
gene-MA16_Dca002756	DNA (cytosine-5)-methyltransferase DRM2 [Dendrobium catenatum]
gene-MA16_Dca022384	calcium-transporting ATPase, endoplasmic reticulum-type [Dendrobium catenatum]

gene-MA16_Dca024106	hypothetical protein MA16_Dca024106 [Dendrobium catenatum]
gene-MA16_Dca000471	hypothetical protein MA16_Dca000471 [Dendrobium catenatum]
gene-MA16_Dca010160	uncharacterized protein LOC110111729 isoform X1 [Dendrobium catenatum]
gene-MA16_Dca028410	probable cellulose synthase A catalytic subunit 8 [UDP-forming] [Dendrobium catenatum]
gene-MA16_Dca024961	U-box domain-containing protein 10 [Dendrobium catenatum]
gene-MA16_Dca002697	DNA helicase INO80 [Dendrobium catenatum]
gene-MA16_Dca019184	BURP domain-containing protein 3 [Dendrobium catenatum]
gene-MA16_Dca008765	zinc finger CCCH domain-containing protein 59 isoform X3 [Dendrobium catenatum]
gene-MA16_Dca001131	hypothetical protein MA16_Dca001131 [Dendrobium catenatum]
gene-MA16_Dca010994	GATA transcription factor 26 [Dendrobium catenatum]
gene-MA16_Dca001427	Retrovirus-related Pol polyprotein from transposon TNT 1-94 [Dendrobium catenatum]
gene-MA16_Dca009044	DNA-directed RNA polymerase II subunit RPB1 [Dendrobium catenatum]
gene-MA16_Dca010021	hypothetical protein MA16_Dca010021 [Dendrobium catenatum]
gene-MA16_Dca015416	protein terminal ear1-like [Dendrobium catenatum]
gene-MA16_Dca015799	subtilisin-like protease SBT1.5 [Dendrobium catenatum]
gene-MA16_Dca012038	Transcription factor GAMYB [Dendrobium catenatum]
gene-MA16_Dca014296	clathrin light chain 1-like [Dendrobium catenatum]
gene-MA16_Dca025545	transcription factor bHLH35 [Dendrobium catenatum]
gene-MA16_Dca000545	replication protein A 70 kDa DNA-binding subunit A-like [Dendrobium catenatum]
gene-MA16_Dca024703	Mitochondrial carrier protein MTM1 [Dendrobium catenatum]
gene-MA16_Dca021298	Protein Mut11 [Dendrobium catenatum]
gene-MA16_Dca014772	Amino acid permease 3 [Dendrobium catenatum]
gene-MA16_Dca014216	hypothetical protein MA16_Dca014216 [Dendrobium catenatum]
gene-MA16_Dca001176	hypothetical protein MA16_Dca001176 [Dendrobium catenatum]
gene-MA16_Dca017444	hypothetical protein MA16_Dca017444 [Dendrobium catenatum]
gene-MA16_Dca023601	tricalbin-3-like [Dendrobium catenatum]
gene-MA16_Dca023667	protein DEHYDRATION-INDUCED 19 homolog 2-like isoform X1 [Dendrobium catenatum]
gene-MA16_Dca012576	enhancer of mRNA-decapping protein 4-like isoform X1 [Dendrobium catenatum]
gene-MA16_Dca000190	uncharacterized protein LOC110092184 [Dendrobium catenatum]
gene-MA16_Dca002998	putative disease resistance protein RGA4 [Dendrobium catenatum]

gene-MA16_Dca027486	Retrovirus-related Pol polyprotein from transposon TNT 1-94 [Dendrobium catenatum]
gene-MA16_Dca007839	Putative F-box protein [Dendrobium catenatum]
gene-MA16_Dca006324	putative mitochondrial protein [Dendrobium catenatum]
gene-MA16_Dca026879	receptor-like protein kinase HSL1 [Dendrobium catenatum]
gene-MA16_Dca003500	protein UPSTREAM OF FLC-like [Dendrobium catenatum]
gene-MA16_Dca014673	Flavonoid 3'-monooxygenase [Dendrobium catenatum]
gene-MA16_Dca011466	uncharacterized protein LOC110098751 [Dendrobium catenatum]
gene-MA16_Dca021850	leucine-rich repeat receptor-like serine/threonine-protein kinase BAM1 [Dendrobium catenatum]
gene-MA16_Dca027822	hypothetical protein MA16_Dca027822 [Dendrobium catenatum]
gene-MA16_Dca009127	putative polyamine oxidase 5 [Dendrobium catenatum]
gene-MA16_Dca000998	Nitrilase 2 [Dendrobium catenatum]
gene-MA16_Dca020793	kinesin-like protein KIN-13A [Dendrobium catenatum]
gene-MA16_Dca008171	Scarecrow-like protein 6 [Dendrobium catenatum]
gene-MA16_Dca001045	hypothetical protein MA16_Dca001045 [Dendrobium catenatum]
gene-MA16_Dca012323	bibenzyl synthase [Dendrobium catenatum]
gene-MA16_Dca020431	Uncharacterized protein MA16_Dca020431 [Dendrobium catenatum]
gene-MA16_Dca004955	NAC domain-containing protein 100 [Dendrobium catenatum]
gene-MA16_Dca023642	hypothetical protein MA16_Dca023642 [Dendrobium catenatum]
gene-MA16_Dca018132	Heparanase-like protein 2 [Dendrobium catenatum]
gene-MA16_Dca022280	65-kDa microtubule-associated protein 6 [Dendrobium catenatum]
gene-MA16_Dca015850	squamosa promoter-binding-like protein 18 isoform X1 [Dendrobium catenatum]
gene-MA16_Dca026323	auxin response factor 17-like [Dendrobium catenatum]
gene-MA16_Dca000152	protein NPG1-like [Dendrobium catenatum]
gene-MA16_Dca022503	photosystem I reaction center subunit N, chloroplastic [Dendrobium catenatum]
gene-MA16_Dca002047	hypothetical protein MA16_Dca002047 [Dendrobium catenatum]
gene-MA16_Dca019792	Retrovirus-related Pol polyprotein from transposon TNT 1-94 [Dendrobium catenatum]
gene-MA16_Dca004447	aspartic proteinase-like isoform X1 [Dendrobium catenatum]
gene-MA16_Dca010456	hypothetical protein MA16_Dca010456 [Dendrobium catenatum]
gene-MA16_Dca016363	protein ROOT PRIMORDIUM DEFECTIVE 1 [Dendrobium catenatum]
gene-MA16_Dca015477	hypothetical protein MA16_Dca015477 [Dendrobium catenatum]

gene-MA16_Dca004892	RHOMBOID-like protein 10, chloroplastic isoform X1 [Dendrobium catenatum]
gene-MA16_Dca003884	Retrovirus-related Pol polyprotein from transposon TNT 1-94 [Dendrobium catenatum]
gene-MA16_Dca025768	putative LRR receptor-like serine/threonine-protein kinase [Dendrobium catenatum]
gene-MA16_Dca003444	Calcium/calmodulin-dependent serine/threonine-protein kinase 1 [Dendrobium catenatum]
gene-MA16_Dca023929	uncharacterized protein LOC110104744 [Dendrobium catenatum]
gene-MA16_Dca003757	putative mitochondrial protein ymf40 [Dendrobium catenatum]
gene-MA16_Dca006786	DNA topoisomerase 2 [Dendrobium catenatum]
gene-MA16_Dca021952	uncharacterized protein LOC110116054 [Dendrobium catenatum]
gene-MA16_Dca005273	ABC transporter B family member 29, chloroplastic [Dendrobium catenatum]
gene-MA16_Dca010058	Calcium sensing receptor, chloroplastic [Dendrobium catenatum]
gene-MA16_Dca014470	Pumilio like 1 [Dendrobium catenatum]
gene-MA16_Dca006054	uncharacterized protein LOC110093273 [Dendrobium catenatum]
gene-MA16_Dca011492	MATE efflux family protein 2, chloroplastic [Dendrobium catenatum]
gene-MA16_Dca017308	hypothetical protein MA16_Dca017308 [Dendrobium catenatum]
gene-MA16_Dca025215	CDPK-related kinase 7-like [Dendrobium catenatum]
gene-MA16_Dca024101	hypothetical protein MA16_Dca024101 [Dendrobium catenatum]
gene-MA16_Dca012699	Putative white-brown complex like protein 30 [Dendrobium catenatum]
gene-MA16_Dca020884	protein LAZ1 homolog 1-like [Dendrobium catenatum]
gene-MA16_Dca021351	all-trans-nonaprenyl-diphosphate synthase [Dendrobium catenatum]
gene-MA16_Dca010909	uncharacterized protein LOC110116322 [Dendrobium catenatum]
gene-MA16_Dca023636	uncharacterized protein LOC110099350 [Dendrobium catenatum]
gene-MA16_Dca011489	protein trichome birefringence-like 34 [Dendrobium catenatum]
gene-MA16_Dca015756	putative mitochondrial protein [Dendrobium catenatum]
gene-MA16_Dca025956	Putative ribonuclease H protein [Dendrobium catenatum]
gene-MA16_Dca000047	hypothetical protein MA16_Dca000047 [Dendrobium catenatum]
gene-MA16_Dca021282	hypothetical protein MA16_Dca021282 [Dendrobium catenatum]
gene-MA16_Dca004836	F-box/LRR-repeat protein 14 isoform X1 [Dendrobium catenatum]
gene-MA16_Dca019643	probable transcription factor KAN2 isoform X1 [Dendrobium catenatum]
gene-MA16_Dca005888	Decapping nuclease Dom3z like, chloroplastic [Dendrobium catenatum]
gene-MA16_Dca013626	hypothetical protein MA16_Dca013626 [Dendrobium catenatum]

gene-MA16_Dca016920	probable inorganic phosphate transporter 1-10 [Dendrobium catenatum]
gene-MA16_Dca020657	pentatricopeptide repeat-containing protein At5g16860-like [Dendrobium catenatum]
gene-MA16_Dca008661	uncharacterized protein LOC110107461 [Dendrobium catenatum]
gene-MA16_Dca015927	mitoferrin [Dendrobium catenatum]
gene-MA16_Dca027794	hypothetical protein MA16_Dca027794 [Dendrobium catenatum]
gene-MA16_Dca012654	pentatricopeptide repeat-containing protein At4g02750-like [Dendrobium catenatum]
gene-MA16_Dca016601	F-box protein At4g35930 isoform X2 [Dendrobium catenatum]
gene-MA16_Dca015064	hypothetical protein MA16_Dca015064 [Dendrobium catenatum]
gene-MA16_Dca001239	Zinc finger CCCH domain-containing protein 18 [Dendrobium catenatum]
gene-MA16_Dca015517	target of Myb protein 1-like isoform X1 [Dendrobium catenatum]
gene-MA16_Dca005175	Acyl carrier protein 2, mitochondrial [Dendrobium catenatum]
gene-MA16_Dca010894	Protein ROOT HAIR DEFECTIVE 3 like 2 [Dendrobium catenatum]
gene-MA16_Dca012631	cytochrome P450 71D7-like [Dendrobium catenatum]
gene-MA16_Dca019690	Retrovirus-related Pol polyprotein from transposon TNT 1-94 [Dendrobium catenatum]
gene-MA16_Dca017713	hypothetical protein MA16_Dca017713 [Dendrobium catenatum]
gene-MA16_Dca007080	hypothetical protein MA16_Dca007080 [Dendrobium catenatum]
gene-MA16_Dca023773	WAT1-related protein At1g43650-like isoform X1 [Dendrobium catenatum]
gene-MA16_Dca022482	BTB/POZ domain-containing protein [Dendrobium catenatum]
gene-MA16_Dca000284	Disease resistance protein RPP13 [Dendrobium catenatum]
gene-MA16_Dca007191	Cellulose synthase-like protein D2 [Dendrobium catenatum]
gene-MA16_Dca010799	leucine-rich repeat receptor-like serine/threonine-protein kinase BAM1 [Dendrobium catenatum]
gene-MA16_Dca002039	Floral homeotic protein APETALA 2 [Dendrobium catenatum]
gene-MA16_Dca006501	Pentatricopeptide repeat-containing protein [Dendrobium catenatum]
gene-MA16_Dca022268	nuclear pore complex protein NUP155 [Dendrobium catenatum]
gene-MA16_Dca001649	putative mitochondrial protein [Dendrobium catenatum]
gene-MA16_Dca008070	3-isopropylmalate dehydratase large subunit, chloroplastic [Dendrobium catenatum]
gene-MA16_Dca019636	Phosphoinositide phosphatase SAC4 [Dendrobium catenatum]
gene-MA16_Dca014820	Ribulose bisphosphate carboxylase large chain [Dendrobium catenatum]
gene-MA16_Dca028548	putative mitochondrial protein [Dendrobium catenatum]
gene-MA16_Dca002308	eukaryotic translation initiation factor 3 subunit K [Dendrobium catenatum]

gene-MA16_Dca017130	putative transcriptional regulator RABBIT EARS [Dendrobium catenatum]
gene-MA16_Dca021994	hypothetical protein MA16_Dca021994 [Dendrobium catenatum]
gene-MA16_Dca000617	probable alpha,alpha-trehalose-phosphate synthase [UDP-forming] 7 [Dendrobium catenatum]
gene-MA16_Dca004804	protein TSS [Dendrobium catenatum]
gene-MA16_Dca005626	receptor-like protein kinase 2 [Dendrobium catenatum]
gene-MA16_Dca004316	L-ascorbate oxidase-like isoform X1 [Dendrobium catenatum]
gene-MA16_Dca023110	DNA-directed RNA polymerase subunit beta'' [Dendrobium catenatum]
gene-MA16_Dca015975	Sulfate transporter 4.1, chloroplastic [Dendrobium catenatum]
gene-MA16_Dca026411	hypothetical protein MA16_Dca026411 [Dendrobium catenatum]
gene-MA16_Dca027610	Geraniol 8-hydroxylase [Dendrobium catenatum]
gene-MA16_Dca022168	hypothetical protein MA16_Dca022168 [Dendrobium catenatum]
gene-MA16_Dca025429	hypothetical protein MA16_Dca025429 [Dendrobium catenatum]
gene-MA16_Dca012849	scarecrow-like protein 9 [Dendrobium catenatum]
gene-MA16_Dca021863	hypothetical protein MA16_Dca021863 [Dendrobium catenatum]
gene-MA16_Dca010588	putative ADP-ribosylation factor GTPase-activating protein AGD13 [Dendrobium catenatum]
gene-MA16_Dca023822	protein CLT1, chloroplastic-like [Dendrobium catenatum]
gene-MA16_Dca016598	GDSL esterase/lipase EXL3-like [Dendrobium catenatum]
gene-MA16_Dca000776	putative disease resistance protein RGA3 [Dendrobium catenatum]
gene-MA16_Dca020424	uncharacterized protein LOC110092974 [Dendrobium catenatum]
gene-MA16_Dca000537	E3 ubiquitin-protein ligase RDUF2-like [Dendrobium catenatum]
gene-MA16_Dca002837	TPR repeat-containing thioredoxin TTL2 [Dendrobium catenatum]
gene-MA16_Dca005051	WUSCHEL-related homeobox 8-like [Dendrobium catenatum]
gene-MA16_Dca001354	Cytoplasmic tRNA 2-thiolation protein 1 [Dendrobium catenatum]
gene-MA16_Dca000847	hypothetical protein MA16_Dca000847 [Dendrobium catenatum]
gene-MA16_Dca018986	ATP-dependent DNA helicase Q-like 5 [Dendrobium catenatum]
gene-MA16_Dca020483	LRR receptor-like serine/threonine-protein kinase GSO2 [Dendrobium catenatum]
gene-MA16_Dca020924	Fatty acyl-CoA reductase 2 [Dendrobium catenatum]
gene-MA16_Dca015948	carboxy-terminal domain RNA polymerase II polypeptide A small phosphatase 1-like [Dendrobium catenatum]
gene-MA16_Dca003911	hypothetical protein MA16_Dca003911 [Dendrobium catenatum]
gene-MA16_Dca017446	ABC transporter C family member 8-like [Dendrobium catenatum]

gene-MA16_Dca006890	ADP-ribosylation factor 1-like [Dendrobium catenatum]
gene-MA16_Dca005151	mannose-specific lectin-like [Dendrobium catenatum]
gene-MA16_Dca004841	putative monogalactosyldiacylglycerol synthase 3, chloroplastic [Dendrobium catenatum]
gene-MA16_Dca027789	Retrovirus-related Pol polyprotein from transposon TNT 1-94 [Dendrobium catenatum]
gene-MA16_Dca021366	hypothetical protein MA16_Dca021366 [Dendrobium catenatum]
gene-MA16_Dca006233	hypothetical protein MA16_Dca006233 [Dendrobium catenatum]
gene-MA16_Dca022933	hypothetical protein MA16_Dca022933 [Dendrobium catenatum]
gene-MA16_Dca002010	peroxidase P7-like [Dendrobium catenatum]
gene-MA16_Dca009302	hypothetical protein MA16_Dca009302 [Dendrobium catenatum]
gene-MA16_Dca023978	phosphatidylinositol-3-phosphatase myotubularin-1 isoform X1 [Dendrobium catenatum]
gene-MA16_Dca022292	protein VAC14 homolog [Dendrobium catenatum]
gene-MA16_Dca012490	putative mitochondrial protein [Dendrobium catenatum]
gene-MA16_Dca002332	hypothetical protein MA16_Dca002332 [Dendrobium catenatum]
gene-MA16_Dca013961	uncharacterized protein LOC110104445 [Dendrobium catenatum]
gene-MA16_Dca005053	putative disease resistance protein RGA3 [Dendrobium catenatum]
gene-MA16_Dca021416	uncharacterized protein LOC110096454 [Dendrobium catenatum]
gene-MA16_Dca004762	hypothetical protein MA16_Dca004762 [Dendrobium catenatum]
gene-MA16_Dca024809	general transcription factor IIH subunit 2 [Dendrobium catenatum]
gene-MA16_Dca008206	glutathione transferase GST 23-like [Dendrobium catenatum]
gene-MA16_Dca001930	Homeobox-leucine zipper protein HOX19 [Dendrobium catenatum]
gene-MA16_Dca023943	hypothetical protein MA16_Dca023943 [Dendrobium catenatum]
gene-MA16_Dca012611	protein phosphatase methylesterase 1 [Dendrobium catenatum]
gene-MA16_Dca024876	hypothetical protein MA16_Dca024876 [Dendrobium catenatum]
gene-MA16_Dca004736	Pentatricopeptide repeat-containing protein [Dendrobium catenatum]
gene-MA16_Dca026841	hypothetical protein MA16_Dca026841 [Dendrobium catenatum]
gene-MA16_Dca013047	peroxiredoxin-2E-1, chloroplastic [Dendrobium catenatum]
gene-MA16_Dca010252	histone-lysine N-methyltransferase ATX4-like isoform X1 [Dendrobium catenatum]
gene-MA16_Dca018995	Shaggy-related protein kinase theta [Dendrobium catenatum]
gene-MA16_Dca003147	6-phosphofructokinase 5, chloroplastic [Dendrobium catenatum]
gene-MA16_Dca019153	serine/arginine-rich splicing factor SC35 [Dendrobium catenatum]

gene-MA16_Dca000468	solute carrier family 35 member F1-like isoform X1 [Dendrobium catenatum]
gene-MA16_Dca001538	hypothetical protein MA16_Dca001538 [Dendrobium catenatum]
gene-MA16_Dca004600	Vesicle-associated membrane protein 721 [Dendrobium catenatum]
gene-MA16_Dca005341	Putative E3 ubiquitin-protein ligase XBAT31 [Dendrobium catenatum]
gene-MA16_Dca028352	Cellulose synthase A catalytic subunit 9 [UDP-forming] [Dendrobium catenatum]
gene-MA16_Dca010535	hypothetical protein MA16_Dca010535 [Dendrobium catenatum]
gene-MA16_Dca019932	uncharacterized protein LOC110107297 isoform X2 [Dendrobium catenatum]
gene-MA16_Dca026571	wall-associated receptor kinase-like 20 [Dendrobium catenatum]
gene-MA16_Dca004158	Phosphatidylinositol 4-phosphate 5-kinase 1 [Dendrobium catenatum]
gene-MA16_Dca019870	hypothetical protein MA16_Dca019870 [Dendrobium catenatum]
gene-MA16_Dca019346	uncharacterized protein LOC110110873 [Dendrobium catenatum]
gene-MA16_Dca000090	squamosa promoter-binding-like protein 7 [Dendrobium catenatum]
gene-MA16_Dca026234	hypothetical protein MA16_Dca026234 [Dendrobium catenatum]
gene-MA16_Dca027103	hypothetical protein MA16_Dca027103 [Dendrobium catenatum]
gene-MA16_Dca011850	Protein IQ-DOMAIN 32 [Dendrobium catenatum]
gene-MA16_Dca007582	hypothetical protein MA16_Dca007582 [Dendrobium catenatum]
gene-MA16_Dca005584	hypothetical protein MA16_Dca005584 [Dendrobium catenatum]
gene-MA16_Dca016440	putative protein phosphatase 2C 57 [Dendrobium catenatum]
gene-MA16_Dca004128	uncharacterized protein LOC110112561 isoform X4 [Dendrobium catenatum]
gene-MA16_Dca003381	protein BOBBER 1 [Dendrobium catenatum]
gene-MA16_Dca002142	hypothetical protein MA16_Dca002142 [Dendrobium catenatum]
gene-MA16_Dca026959	Organic cation/carnitine transporter 2 [Dendrobium catenatum]
gene-MA16_Dca013818	probable proline transporter 2 [Dendrobium catenatum]
gene-MA16_Dca026995	18.6 kDa class III heat shock protein [Dendrobium catenatum]
gene-MA16_Dca022452	G-type lectin S-receptor-like serine/threonine-protein kinase [Dendrobium catenatum]
gene-MA16_Dca001472	floral homeotic protein AGAMOUS-like [Dendrobium catenatum]
gene-MA16_Dca026808	PTI1-like tyrosine-protein kinase 2 [Dendrobium catenatum]
gene-MA16_Dca018400	Protein IQ-DOMAIN 1 [Dendrobium catenatum]
gene-MA16_Dca010930	hypothetical protein MA16_Dca010930 [Dendrobium catenatum]
gene-MA16_Dca010007	Xaa-Pro dipeptidase [Dendrobium catenatum]

gene-MA16_Dca003307	putative pentatricopeptide repeat-containing protein At5g59900 [Dendrobium catenatum]
gene-MA16_Dca011089	probable galacturonosyltransferase 7 [Dendrobium catenatum]
gene-MA16_Dca025353	threonine dehydratase [Dendrobium catenatum]
gene-MA16_Dca022914	putative fructose-bisphosphate aldolase 3, chloroplastic [Dendrobium catenatum]
gene-MA16_Dca000999	protein SMAX1-LIKE 2-like [Dendrobium catenatum]
gene-MA16_Dca001189	hypothetical protein MA16_Dca001189 [Dendrobium catenatum]
gene-MA16_Dca021175	putative pentatricopeptide repeat-containing protein At1g74580 [Dendrobium catenatum]
gene-MA16_Dca019151	uncharacterized protein LOC110092254 isoform X1 [Dendrobium catenatum]
gene-MA16_Dca004772	probable LRR receptor-like serine/threonine-protein kinase At1g06840 [Dendrobium catenatum]
gene-MA16_Dca018663	lysine-specific demethylase JMJ25-like [Dendrobium catenatum]
gene-MA16_Dca017411	protein IQ-DOMAIN 1-like isoform X1 [Dendrobium catenatum]
gene-MA16_Dca003194	transport inhibitor response 1-like protein Os05g0150500 [Dendrobium catenatum]
gene-MA16_Dca006595	receptor-like protein 12 isoform X1 [Dendrobium catenatum]
gene-MA16_Dca015811	Pentatricopeptide repeat-containing protein [Dendrobium catenatum]
gene-MA16_Dca001922	hypothetical protein MA16_Dca001922 [Dendrobium catenatum]
gene-MA16_Dca000347	glycerol-3-phosphate acyltransferase 1-like [Dendrobium catenatum]
gene-MA16_Dca016112	ATP-dependent DNA helicase 2 subunit KU80 [Dendrobium catenatum]
gene-MA16_Dca018211	hypothetical protein MA16_Dca018211 [Dendrobium catenatum]
gene-MA16_Dca013055	Primary amine oxidase [Dendrobium catenatum]
gene-MA16_Dca017541	prolyl-tRNA synthetase [Dendrobium catenatum]
gene-MA16_Dca011873	auxin response factor 12-like isoform X1 [Dendrobium catenatum]
gene-MA16_Dca022235	2-C-methyl-D-erythritol 4-phosphate cytidylyltransferase, chloroplastic [Dendrobium catenatum]
gene-MA16_Dca001550	probable methyltransferase PMT13 [Dendrobium catenatum]
gene-MA16_Dca014631	DNA repair protein RAD51 homolog 3 isoform X1 [Dendrobium catenatum]
gene-MA16_Dca002391	hypothetical protein MA16_Dca002391 [Dendrobium catenatum]
gene-MA16_Dca006616	protein CHROMATIN REMODELING 5 [Dendrobium catenatum]
gene-MA16_Dca024998	psbP domain-containing protein 4, chloroplastic [Dendrobium catenatum]
gene-MA16_Dca024322	BTB/POZ domain-containing protein [Dendrobium catenatum]
gene-MA16_Dca004156	hypothetical protein MA16_Dca004156 [Dendrobium catenatum]
gene-MA16_Dca010653	E3 ubiquitin-protein ligase RDUF2-like [Dendrobium catenatum]

gene-MA16_Dca027263	hypothetical protein MA16_Dca027263 [Dendrobium catenatum]
gene-MA16_Dca009400	uncharacterized protein LOC110104256 [Dendrobium catenatum]
gene-MA16_Dca022905	pentatricopeptide repeat-containing protein At5g15280 isoform X1 [Dendrobium catenatum]
gene-MA16_Dca021047	extradiol ring-cleavage dioxygenase-like [Dendrobium catenatum]
gene-MA16_Dca008557	uncharacterized protein LOC110097094 [Dendrobium catenatum]
gene-MA16_Dca015083	Putative ribonuclease H protein [Dendrobium catenatum]
gene-MA16_Dca017912	uncharacterized protein DDB_G0283697 [Dendrobium catenatum]
gene-MA16_Dca013338	CCR4-NOT transcription complex subunit 4 [Dendrobium catenatum]
gene-MA16_Dca004325	hypothetical protein MA16_Dca004325 [Dendrobium catenatum]
gene-MA16_Dca020077	mavicyanin-like [Dendrobium catenatum]
gene-MA16_Dca001828	uncharacterized protein LOC110114606 [Dendrobium catenatum]
gene-MA16_Dca008822	Serine/threonine-protein kinase Nek6 [Dendrobium catenatum]
gene-MA16_Dca007698	hypothetical protein MA16_Dca007698 [Dendrobium catenatum]
gene-MA16_Dca018219	hypothetical protein MA16_Dca018219 [Dendrobium catenatum]
gene-MA16_Dca014033	uncharacterized protein LOC110112570 [Dendrobium catenatum]
gene-MA16_Dca023970	hypothetical protein MA16_Dca023970 [Dendrobium catenatum]
gene-MA16_Dca022211	putative mitochondrial protein [Dendrobium catenatum]
gene-MA16_Dca004003	Retrovirus-related Pol polyprotein from transposon TNT 1-94 [Dendrobium catenatum]
gene-MA16_Dca021498	Cell division cycle protein 48 like [Dendrobium catenatum]
gene-MA16_Dca008238	ricin B-like lectin R40G3 [Dendrobium catenatum]
gene-MA16_Dca006742	putative alpha-mannosidase I MNS4 [Dendrobium catenatum]
gene-MA16_Dca005024	Gibberellin 2-beta-dioxygenase 2 [Dendrobium catenatum]
gene-MA16_Dca018017	1-acyl-sn-glycerol-3-phosphate acyltransferase 1, chloroplastic [Dendrobium catenatum]
gene-MA16_Dca012816	Retrovirus-related Pol polyprotein from transposon TNT 1-94 [Dendrobium catenatum]
gene-MA16_Dca006362	hypothetical protein MA16_Dca006362 [Dendrobium catenatum]
gene-MA16_Dca025530	ras-related protein RABE1c-like [Dendrobium catenatum]
gene-MA16_Dca027631	hypothetical protein MA16_Dca027631 [Dendrobium catenatum]
gene-MA16_Dca023928	hypothetical protein MA16_Dca023928 [Dendrobium catenatum]
gene-MA16_Dca010687	GDSL esterase/lipase [Dendrobium catenatum]
gene-MA16_Dca028211	125 kDa kinesin-related protein [Dendrobium catenatum]

gene-MA16_Dca019753	hypothetical protein MA16_Dca019753 [Dendrobium catenatum]
gene-MA16_Dca014704	Putative ribonuclease H protein [Dendrobium catenatum]
gene-MA16_Dca003210	peptide methionine sulfoxide reductase B5-like [Dendrobium catenatum]
gene-MA16_Dca003122	endoribonuclease Dicer homolog 4 isoform X2 [Dendrobium catenatum]
gene-MA16_Dca017043	hypothetical protein MA16_Dca017043 [Dendrobium catenatum]
gene-MA16_Dca018414	histone-lysine N-methyltransferase, H3 lysine-9 specific SUVH5-like [Dendrobium catenatum]
gene-MA16_Dca007798	AP3-complex subunit beta-A [Dendrobium catenatum]
gene-MA16_Dca011403	hypothetical protein MA16_Dca011403 [Dendrobium catenatum]
gene-MA16_Dca012397	protein ENHANCED DISEASE RESISTANCE 2-like [Dendrobium catenatum]
gene-MA16_Dca015180	Uncharacterized protein MA16_Dca015180 [Dendrobium catenatum]
gene-MA16_Dca014974	uncharacterized protein LOC110096268 isoform X1 [Dendrobium catenatum]
gene-MA16_Dca011997	Uncharacterized protein MA16_Dca011997 [Dendrobium catenatum]
gene-MA16_Dca005635	hypothetical protein MA16_Dca005635 [Dendrobium catenatum]
gene-MA16_Dca000891	heparan-alpha-glucosaminide N-acetyltransferase [Dendrobium catenatum]
gene-MA16_Dca005118	DEAD-box ATP-dependent RNA helicase 48 [Dendrobium catenatum]
gene-MA16_Dca003169	U3 small nucleolar ribonucleoprotein protein IMP4 [Dendrobium catenatum]
gene-MA16_Dca006776	UTP:RNA uridylyltransferase 1 isoform X2 [Dendrobium catenatum]
gene-MA16_Dca027594	putative mitochondrial protein [Dendrobium catenatum]
gene-MA16_Dca006091	Filament-like plant protein 1 [Dendrobium catenatum]
gene-MA16_Dca004606	hypothetical protein MA16_Dca004606 [Dendrobium catenatum]
gene-MA16_Dca020750	probable LRR receptor-like serine/threonine-protein kinase At4g20940 isoform X1 [Dendrobium catenatum]
gene-MA16_Dca019905	probable RNA methyltransferase At5g51130 [Dendrobium catenatum]
gene-MA16_Dca028464	Retrovirus-related Pol polyprotein from transposon TNT 1-94 [Dendrobium catenatum]
gene-MA16_Dca021165	Pentatricopeptide repeat-containing protein [Dendrobium catenatum]
gene-MA16_Dca014349	hypothetical protein MA16_Dca014349 [Dendrobium catenatum]
gene-MA16_Dca019273	katanin p80 WD40 repeat-containing subunit B1 homolog [Dendrobium catenatum]
gene-MA16_Dca023840	heat shock protein 81-1 [Dendrobium catenatum]
gene-MA16_Dca003056	hypothetical protein MA16_Dca003056 [Dendrobium catenatum]
gene-MA16_Dca015790	Uncharacterized protein MA16_Dca015790 [Dendrobium catenatum]
gene-MA16_Dca001078	zinc finger AN1 and C2H2 domain-containing stress-associated protein 16-like [Dendrobium catenatum]

gene-MA16_Dca021436	hypothetical protein MA16_Dca021436 [Dendrobium catenatum]
gene-MA16_Dca009679	hypothetical protein MA16_Dca009679 [Dendrobium catenatum]
gene-MA16_Dca019278	Alpha-galactosidase [Dendrobium catenatum]
gene-MA16_Dca005938	hypothetical protein MA16_Dca005938 [Dendrobium catenatum]
gene-MA16_Dca010730	hypothetical protein MA16_Dca010730 [Dendrobium catenatum]
gene-MA16_Dca009281	hypothetical protein MA16_Dca009281 [Dendrobium catenatum]
gene-MA16_Dca022573	Cytokinin dehydrogenase 2 [Dendrobium catenatum]
gene-MA16_Dca007128	putative pentatricopeptide repeat-containing protein At5g13230, mitochondrial [Dendrobium catenatum]
gene-MA16_Dca016572	protein LOW PSII ACCUMULATION 3, chloroplastic isoform X1 [Dendrobium catenatum]
gene-MA16_Dca021283	TPR repeat-containing thioredoxin TTL1 [Dendrobium catenatum]
gene-MA16_Dca020878	hypothetical protein MA16_Dca020878 [Dendrobium catenatum]
gene-MA16_Dca019385	serine/threonine-protein kinase D6PK-like [Dendrobium catenatum]
gene-MA16_Dca004182	hypothetical protein MA16_Dca004182 [Dendrobium catenatum]
gene-MA16_Dca015271	Ultraviolet-B receptor UVR8 [Dendrobium catenatum]
gene-MA16_Dca020111	scarecrow-like protein 6 [Dendrobium catenatum]
gene-MA16_Dca002586	Retrovirus-related Pol polyprotein from transposon TNT 1-94 [Dendrobium catenatum]
gene-MA16_Dca018734	hypothetical protein MA16_Dca018734 [Dendrobium catenatum]
gene-MA16_Dca028729	hypothetical protein MA16_Dca028729 [Dendrobium catenatum]
gene-MA16_Dca002207	Protein ASPARTIC PROTEASE IN GUARD CELL 2 [Dendrobium catenatum]
gene-MA16_Dca000753	putative mitochondrial protein [Dendrobium catenatum]
gene-MA16_Dca011281	Serine/threonine-protein kinase CTR1 [Dendrobium catenatum]
gene-MA16_Dca016286	Putative phospholipid-transporting ATPase 4 [Dendrobium catenatum]
gene-MA16_Dca017669	Retrovirus-related Pol polyprotein from transposon TNT 1-94 [Dendrobium catenatum]
gene-MA16_Dca020524	hypothetical protein MA16_Dca020524 [Dendrobium catenatum]
gene-MA16_Dca025663	putative LRR receptor-like serine/threonine-protein kinase [Dendrobium catenatum]
gene-MA16_Dca017777	hypothetical protein MA16_Dca017777 [Dendrobium catenatum]
gene-MA16_Dca007005	hypothetical protein MA16_Dca007005 [Dendrobium catenatum]
gene-MA16_Dca005473	Protochlorophyllide-dependent translocon component 52, chloroplastic [Dendrobium catenatum]
gene-MA16_Dca017215	hypothetical protein MA16_Dca017215 [Dendrobium catenatum]
gene-MA16_Dca027254	hypothetical protein MA16_Dca027254 [Dendrobium catenatum]

gene-MA16_Dca006898	uncharacterized protein LOC110111686 isoform X2 [Dendrobium catenatum]
gene-MA16_Dca020517	Secologanin synthase [Dendrobium catenatum]
gene-MA16_Dca025840	Polyadenylate-binding protein 2 [Dendrobium catenatum]
gene-MA16_Dca015518	hypothetical protein MA16_Dca015518 [Dendrobium catenatum]
gene-MA16_Dca008229	Retrovirus-related Pol polyprotein from transposon TNT 1-94 [Dendrobium catenatum]
gene-MA16_Dca023216	hypothetical protein MA16_Dca023216 [Dendrobium catenatum]
gene-MA16_Dca024036	29 kDa ribonucleoprotein A, chloroplastic [Dendrobium catenatum]
gene-MA16_Dca007864	thioredoxin F, chloroplastic-like [Dendrobium catenatum]
gene-MA16_Dca006773	uncharacterized protein LOC110107686 [Dendrobium catenatum]
gene-MA16_Dca012978	hypothetical protein MA16_Dca012978 [Dendrobium catenatum]
gene-MA16_Dca019891	Disease resistance protein RGA2 [Dendrobium catenatum]
gene-MA16_Dca015101	uncharacterized protein LOC110104281 [Dendrobium catenatum]
gene-MA16_Dca020738	F-box protein SKIP19-like [Dendrobium catenatum]
gene-MA16_Dca020349	Retrovirus-related Pol polyprotein from transposon TNT 1-94 [Dendrobium catenatum]
gene-MA16_Dca018035	Putative ribonuclease H protein [Dendrobium catenatum]
gene-MA16_Dca027199	G-type lectin S-receptor-like serine/threonine-protein kinase SD2-5 [Dendrobium catenatum]
gene-MA16_Dca002588	NAC domain-containing protein 68-like [Dendrobium catenatum]
gene-MA16_Dca019572	transcription factor TCP24-like [Dendrobium catenatum]
gene-MA16_Dca016699	SEC12-like protein 2 [Dendrobium catenatum]
gene-MA16_Dca002133	DEAD-box ATP-dependent RNA helicase 24 [Dendrobium catenatum]
gene-MA16_Dca014117	probable E3 ubiquitin-protein ligase RHB1A [Dendrobium catenatum]
gene-MA16_Dca001951	Xyloglucan galactosyltransferase KATAMARI1 like [Dendrobium catenatum]
gene-MA16_Dca024988	fatty-acid-binding protein 2 [Dendrobium catenatum]
gene-MA16_Dca017598	Zinc finger CCCH domain-containing protein 65 [Dendrobium catenatum]
gene-MA16_Dca011590	protein ODORANT1-like [Dendrobium catenatum]
gene-MA16_Dca006002	eukaryotic translation initiation factor 5A-2 [Dendrobium catenatum]
gene-MA16_Dca004236	protein CUP-SHAPED COTYLEDON 2-like [Dendrobium catenatum]
gene-MA16_Dca001411	cytochrome P450 71A1-like [Dendrobium catenatum]
gene-MA16_Dca003148	myb-related protein 306-like [Dendrobium catenatum]
gene-MA16_Dca026276	hypothetical protein MA16_Dca026276 [Dendrobium catenatum]

gene-MA16_Dca023073	hypothetical protein MA16_Dca023073 [Dendrobium catenatum]
gene-MA16_Dca014997	probable galacturonosyltransferase 7 isoform X2 [Dendrobium catenatum]
gene-MA16_Dca021807	hypothetical protein MA16_Dca021807 [Dendrobium catenatum]
gene-MA16_Dca008635	Aspartic proteinase Asp1 [Dendrobium catenatum]
gene-MA16_Dca015933	multiple inositol polyphosphate phosphatase 1 [Dendrobium catenatum]
gene-MA16_Dca016402	inorganic phosphate transporter 1-4-like [Dendrobium catenatum]
gene-MA16_Dca016634	Dol-P-Man:Man(5)GlcNAc(2)-PP-Dol alpha-1,3-mannosyltransferase [Dendrobium catenatum]
gene-MA16_Dca003076	CTP synthase [Dendrobium catenatum]
gene-MA16_Dca010931	myb family transcription factor EFM-like isoform X1 [Dendrobium catenatum]
gene-MA16_Dca012268	hypothetical protein MA16_Dca012268 [Dendrobium catenatum]
gene-MA16_Dca013261	uncharacterized protein LOC110112590 [Dendrobium catenatum]
gene-MA16_Dca022150	uncharacterized protein LOC110109896 [Dendrobium catenatum]
gene-MA16_Dca001069	hypothetical protein MA16_Dca001069 [Dendrobium catenatum]
gene-MA16_Dca010625	uncharacterized protein LOC110108420 [Dendrobium catenatum]
gene-MA16_Dca002337	E3 ubiquitin ligase BIG BROTHER [Dendrobium catenatum]
gene-MA16_Dca008457	Formin-like protein 11 [Dendrobium catenatum]
gene-MA16_Dca006665	hypothetical protein MA16_Dca006665 [Dendrobium catenatum]
gene-MA16_Dca002844	tubulin beta-2 chain [Dendrobium catenatum]
gene-MA16_Dca023657	hypothetical protein MA16_Dca023657 [Dendrobium catenatum]
gene-MA16_Dca007473	hypothetical protein MA16_Dca007473 [Dendrobium catenatum]
gene-MA16_Dca007732	hypothetical protein MA16_Dca007732 [Dendrobium catenatum]
gene-MA16_Dca022518	hypothetical protein MA16_Dca022518 [Dendrobium catenatum]
gene-MA16_Dca002693	thylakoid lumenal 16.5 kDa protein, chloroplastic [Dendrobium catenatum]
gene-MA16_Dca002968	hypothetical protein MA16_Dca002968 [Dendrobium catenatum]
gene-MA16_Dca020866	hypothetical protein MA16_Dca020866 [Dendrobium catenatum]
gene-MA16_Dca022176	hypothetical protein MA16_Dca022176 [Dendrobium catenatum]
gene-MA16_Dca008624	polygalacturonase inhibitor 2-like [Dendrobium catenatum]
gene-MA16_Dca000565	hypothetical protein MA16_Dca000565 [Dendrobium catenatum]
gene-MA16_Dca016687	hypothetical protein MA16_Dca016687 [Dendrobium catenatum]
gene-MA16_Dca004215	hypothetical protein MA16_Dca004215 [Dendrobium catenatum]

gene-MA16_Dca021565	uncharacterized protein LOC110092103 isoform X1 [Dendrobium catenatum]
gene-MA16_Dca021949	Mitogen-activated protein kinase kinase kinase ANP1 [Dendrobium catenatum]
gene-MA16_Dca021544	hypothetical protein MA16_Dca021544 [Dendrobium catenatum]
gene-MA16_Dca027105	pentatricopeptide repeat-containing protein At5g16860-like [Dendrobium catenatum]
gene-MA16_Dca021979	cleavage stimulation factor subunit 50 isoform X4 [Dendrobium catenatum]
gene-MA16_Dca016948	uncharacterized protein LOC110097744 isoform X1 [Dendrobium catenatum]
gene-MA16_Dca014981	Beta-D-xylosidase 4 [Dendrobium catenatum]
gene-MA16_Dca019880	Retrovirus-related Pol polyprotein from transposon TNT 1-94 [Dendrobium catenatum]
gene-MA16_Dca013976	UDP-glucuronic acid decarboxylase 5 [Dendrobium catenatum]
gene-MA16_Dca023276	eukaryotic peptide chain release factor GTP-binding subunit ERF3A-like [Dendrobium catenatum]
gene-MA16_Dca018725	growth-regulating factor 4-like isoform X2 [Dendrobium catenatum]
gene-MA16_Dca023223	protein LURP-one-related 6 [Dendrobium catenatum]
gene-MA16_Dca024154	uncharacterized protein LOC110107561 isoform X2 [Dendrobium catenatum]
gene-MA16_Dca021529	tRNA pseudouridine38/39 synthase [Dendrobium catenatum]
gene-MA16_Dca001413	probable potassium transporter 11 isoform X2 [Dendrobium catenatum]
gene-MA16_Dca017373	hypothetical protein MA16_Dca017373 [Dendrobium catenatum]
gene-MA16_Dca018457	hypothetical protein MA16_Dca018457 [Dendrobium catenatum]
gene-MA16_Dca023835	IN2-2 protein [Dendrobium catenatum]
gene-MA16_Dca014912	Protein GLUTAMINE DUMPER 4 [Dendrobium catenatum]
gene-MA16_Dca026585	hypothetical protein MA16_Dca026585 [Dendrobium catenatum]
gene-MA16_Dca023855	Glucose-6-phosphate 1-dehydrogenase 2, chloroplastic [Dendrobium catenatum]
gene-MA16_Dca003117	hypothetical protein MA16_Dca003117 [Dendrobium catenatum]
gene-MA16_Dca007512	probable protein S-acyltransferase 12 [Dendrobium catenatum]
gene-MA16_Dca021249	putative mitochondrial protein [Dendrobium catenatum]
gene-MA16_Dca010818	hypothetical protein MA16_Dca010818 [Dendrobium catenatum]
gene-MA16_Dca006046	uncharacterized protein LOC110093300 isoform X1 [Dendrobium catenatum]
gene-MA16_Dca025558	putative mitochondrial protein [Dendrobium catenatum]
gene-MA16_Dca024400	exosome complex exonuclease RRP44 homolog A [Dendrobium catenatum]
gene-MA16_Dca025093	pentatricopeptide repeat-containing protein At2g15980 [Dendrobium catenatum]
gene-MA16_Dca003193	UPF0613 protein PB24D3.06c isoform X1 [Dendrobium catenatum]

gene-MA16_Dca012776	homoserine kinase-like [Dendrobium catenatum]
gene-MA16_Dca024208	probable histidine kinase 2 isoform X1 [Dendrobium catenatum]
gene-MA16_Dca018497	protein HUA2-LIKE 3-like isoform X3 [Dendrobium catenatum]
gene-MA16_Dca016118	MAP3K epsilon protein kinase 1-like [Dendrobium catenatum]
gene-MA16_Dca021751	hypothetical protein MA16_Dca021751 [Dendrobium catenatum]
gene-MA16_Dca022572	hypothetical protein MA16_Dca022572 [Dendrobium catenatum]
gene-MA16_Dca026307	Cyclin-C1-1 [Dendrobium catenatum]
gene-MA16_Dca023243	hypothetical protein MA16_Dca023243 [Dendrobium catenatum]
gene-MA16_Dca007060	transcription factor VIP1 [Dendrobium catenatum]
gene-MA16_Dca007034	Retrovirus-related Pol polyprotein from transposon TNT 1-94 [Dendrobium catenatum]
gene-MA16_Dca026208	uncharacterized protein LOC110093226 [Dendrobium catenatum]
gene-MA16_Dca010441	hypothetical protein MA16_Dca010441 [Dendrobium catenatum]
gene-MA16_Dca007090	potassium transporter 7-like [Dendrobium catenatum]
gene-MA16_Dca019790	KH domain-containing protein [Dendrobium catenatum]
gene-MA16_Dca013902	MACPF domain-containing protein [Dendrobium catenatum]
gene-MA16_Dca022392	Retrovirus-related Pol polyprotein from transposon TNT 1-94 [Dendrobium catenatum]
gene-MA16_Dca009375	hypothetical protein MA16_Dca009375 [Dendrobium catenatum]
gene-MA16_Dca022838	S-linalool synthase [Dendrobium catenatum]
gene-MA16_Dca007879	putative mannitol dehydrogenase [Dendrobium catenatum]
gene-MA16_Dca008921	CHD3-type chromatin-remodeling factor PICKLE isoform X3 [Dendrobium catenatum]
gene-MA16_Dca024641	hypothetical protein MA16_Dca024641 [Dendrobium catenatum]
gene-MA16_Dca023665	hypothetical protein MA16_Dca023665 [Dendrobium catenatum]
gene-MA16_Dca026991	octanoyltransferase [Dendrobium catenatum]
gene-MA16_Dca009289	hypothetical protein MA16_Dca009289 [Dendrobium catenatum]
gene-MA16_Dca007307	pentatricopeptide repeat-containing protein At4g26680, mitochondrial [Dendrobium catenatum]
gene-MA16_Dca021203	hypothetical protein MA16_Dca021203 [Dendrobium catenatum]
gene-MA16_Dca019966	Putative ribonuclease H protein [Dendrobium catenatum]
gene-MA16_Dca012449	tRNA A64-2'-O-ribosylphosphate transferase isoform X1 [Dendrobium catenatum]
gene-MA16_Dca024776	Retrovirus-related Pol polyprotein from transposon TNT 1-94 [Dendrobium catenatum]
gene-MA16_Dca023326	protein LIKE COV 2-like isoform X1 [Dendrobium catenatum]

gene-MA16_Dca016176	Putative disease resistance protein RGA4 [Dendrobium catenatum]
gene-MA16_Dca009947	CDK5RAP3-like protein [Dendrobium catenatum]
gene-MA16_Dca020016	Endochitinase B [Dendrobium catenatum]
gene-MA16_Dca025929	protein STRICTOSIDINE SYNTHASE-LIKE 13 [Dendrobium catenatum]
gene-MA16_Dca018676	hypothetical protein MA16_Dca018676 [Dendrobium catenatum]
gene-MA16_Dca023402	protein trichome birefringence-like 5 [Dendrobium catenatum]
gene-MA16_Dca011027	Pentatricopeptide repeat-containing protein [Dendrobium catenatum]
gene-MA16_Dca011387	Amino acid permease 2 [Dendrobium catenatum]
gene-MA16_Dca021290	hypothetical protein MA16_Dca021290 [Dendrobium catenatum]
gene-MA16_Dca009335	Transcription factor TCP2 [Dendrobium catenatum]
gene-MA16_Dca002527	Retrovirus-related Pol polyprotein from transposon TNT 1-94 [Dendrobium catenatum]
gene-MA16_Dca018160	RNA-directed DNA polymerase [Dendrobium catenatum]
gene-MA16_Dca007248	scarecrow-like protein 27 [Dendrobium catenatum]
gene-MA16_Dca003426	RING-H2 finger protein ATL8 [Dendrobium catenatum]
gene-MA16_Dca028670	hypothetical protein MA16_Dca028670 [Dendrobium catenatum]
gene-MA16_Dca013437	DNA-directed RNA polymerase subunit beta'' [Dendrobium catenatum]
gene-MA16_Dca023878	B2 protein [Dendrobium catenatum]
gene-MA16_Dca005952	hypothetical protein MA16_Dca005952 [Dendrobium catenatum]
gene-MA16_Dca015430	Premnaspirodiene oxygenase [Dendrobium catenatum]
gene-MA16_Dca018844	lysine-specific demethylase 5A [Dendrobium catenatum]
gene-MA16_Dca020968	hydroxyproline O-galactosyltransferase HPGT1 isoform X2 [Dendrobium catenatum]
gene-MA16_Dca003634	hypothetical protein MA16_Dca003634 [Dendrobium catenatum]
gene-MA16_Dca014710	Retrovirus-related Pol polyprotein from transposon TNT 1-94 [Dendrobium catenatum]
gene-MA16_Dca017923	probable pectin methyltransferase QUA2 [Dendrobium catenatum]
gene-MA16_Dca004199	CBL-interacting serine/threonine-protein kinase 11 [Dendrobium catenatum]
gene-MA16_Dca003733	125 kDa kinesin-related protein [Dendrobium catenatum]
gene-MA16_Dca003689	Transcription factor bHLH82 [Dendrobium catenatum]
gene-MA16_Dca017805	protein CHROMATIN REMODELING 24 [Dendrobium catenatum]
gene-MA16_Dca024023	purple acid phosphatase 15 [Dendrobium catenatum]
gene-MA16_Dca015575	hypothetical protein MA16_Dca015575 [Dendrobium catenatum]

gene-MA16_Dca016262	Retrovirus-related Pol polyprotein from transposon TNT 1-94 [Dendrobium catenatum]
gene-MA16_Dca028500	hypothetical protein MA16_Dca028500 [Dendrobium catenatum]
gene-MA16_Dca001725	UPF0503 protein At3g09070, chloroplastic-like [Dendrobium catenatum]
gene-MA16_Dca026681	leucyl-tRNA synthetase [Dendrobium catenatum]
gene-MA16_Dca020890	hypothetical protein MA16_Dca020890 [Dendrobium catenatum]
gene-MA16_Dca004307	Gamma-glutamyltranspeptidase 1 [Dendrobium catenatum]
gene-MA16_Dca013450	Disease resistance protein RGA2 [Dendrobium catenatum]
gene-MA16_Dca010469	hypothetical protein MA16_Dca010469 [Dendrobium catenatum]
gene-MA16_Dca028394	Peroxidase 50 [Dendrobium catenatum]
gene-MA16_Dca013832	Pentatricopeptide repeat-containing protein [Dendrobium catenatum]
gene-MA16_Dca019865	hypothetical protein MA16_Dca019865 [Dendrobium catenatum]
gene-MA16_Dca017076	putative mitochondrial protein [Dendrobium catenatum]
gene-MA16_Dca002226	uncharacterized protein LOC110111878 isoform X1 [Dendrobium catenatum]
gene-MA16_Dca008063	hypothetical protein MA16_Dca008063 [Dendrobium catenatum]
gene-MA16_Dca003670	probable sodium-coupled neutral amino acid transporter 6 [Dendrobium catenatum]
gene-MA16_Dca022913	Trihelix transcription factor GT-3a [Dendrobium catenatum]
gene-MA16_Dca020114	uncharacterized protein LOC110096332 [Dendrobium catenatum]
gene-MA16_Dca024206	Protein CYPRO4 [Dendrobium catenatum]
gene-MA16_Dca015175	Shikimate O-hydroxycinnamoyltransferase [Dendrobium catenatum]
gene-MA16_Dca027327	pentatricopeptide repeat-containing protein At5g66520-like [Dendrobium catenatum]
gene-MA16_Dca004540	G-type lectin S-receptor-like serine/threonine-protein kinase SD2-5 [Dendrobium catenatum]
gene-MA16_Dca002895	uncharacterized protein LOC110116761 isoform X2 [Dendrobium catenatum]
gene-MA16_Dca013497	Pentatricopeptide repeat-containing protein [Dendrobium catenatum]
gene-MA16_Dca015918	uncharacterized protein LOC110101179 isoform X1 [Dendrobium catenatum]
gene-MA16_Dca003662	probable 2-oxoglutarate-dependent dioxygenase AOP1 [Dendrobium catenatum]
gene-MA16_Dca024404	uncharacterized protein LOC110111870 [Dendrobium catenatum]
gene-MA16_Dca005980	uncharacterized protein LOC110110813 [Dendrobium catenatum]
gene-MA16_Dca026139	auxin response factor 18-like isoform X2 [Dendrobium catenatum]
gene-MA16_Dca020534	protein TPX2 [Dendrobium catenatum]
gene-MA16_Dca016479	anthranilate O-methyltransferase 1-like [Dendrobium catenatum]

gene-MA16_Dca004477	Putative pentatricopeptide repeat-containing protein [Dendrobium catenatum]
gene-MA16_Dca025400	Polygalacturonase inhibitor [Dendrobium catenatum]
gene-MA16_Dca012863	uncharacterized protein LOC110108917 [Dendrobium catenatum]
gene-MA16_Dca017578	putative S-acyltransferase [Dendrobium catenatum]
gene-MA16_Dca000609	enhancer of mRNA-decapping protein 4-like [Dendrobium catenatum]
gene-MA16_Dca010322	60S ribosomal protein L4-1-like [Dendrobium catenatum]
gene-MA16_Dca028712	hypothetical protein MA16_Dca028712 [Dendrobium catenatum]
gene-MA16_Dca004486	hypothetical protein MA16_Dca004486 [Dendrobium catenatum]
gene-MA16_Dca012560	uncharacterized protein LOC110099068 isoform X2 [Dendrobium catenatum]
gene-MA16_Dca011528	receptor-like serine/threonine-protein kinase ALE2 isoform X2 [Dendrobium catenatum]
gene-MA16_Dca026280	uncharacterized protein LOC110110575 [Dendrobium catenatum]
gene-MA16_Dca015852	hypothetical protein MA16_Dca015852 [Dendrobium catenatum]
gene-MA16_Dca001020	hypothetical protein MA16_Dca001020 [Dendrobium catenatum]
gene-MA16_Dca011712	hypothetical protein MA16_Dca011712 [Dendrobium catenatum]
gene-MA16_Dca012677	U-box domain-containing protein 52-like [Dendrobium catenatum]
gene-MA16_Dca011682	Disease resistance protein RGA2 [Dendrobium catenatum]
gene-MA16_Dca011476	hypothetical protein MA16_Dca011476 [Dendrobium catenatum]
gene-MA16_Dca012338	Superoxide dismutase [Cu-Zn], chloroplastic [Dendrobium catenatum]
gene-MA16_Dca008376	uncharacterized protein LOC110099989 [Dendrobium catenatum]
gene-MA16_Dca012890	cytochrome P450 84A1 [Dendrobium catenatum]
gene-MA16_Dca023239	probable glycosyltransferase 2 [Dendrobium catenatum]
gene-MA16_Dca003039	uncharacterized protein LOC110108544 isoform X1 [Dendrobium catenatum]
gene-MA16_Dca019516	hypothetical protein MA16_Dca019516 [Dendrobium catenatum]
gene-MA16_Dca015612	putative 1-phosphatidylinositol-3-phosphate 5-kinase FAB1C [Dendrobium catenatum]
gene-MA16_Dca013972	uncharacterized protein LOC110104431 [Dendrobium catenatum]
gene-MA16_Dca006829	uncharacterized protein LOC110092461 [Dendrobium catenatum]
gene-MA16_Dca002597	transformation/transcription domain-associated protein-like [Dendrobium catenatum]
gene-MA16_Dca016667	Retrovirus-related Pol polyprotein from transposon TNT 1-94 [Dendrobium catenatum]
gene-MA16_Dca002789	uncharacterized protein LOC110097862 [Dendrobium catenatum]
gene-MA16_Dca015044	Isopentenyl-diphosphate Delta-isomerase I [Dendrobium catenatum]

gene-MA16_Dca016470	transcription factor bHLH130-like [Dendrobium catenatum]
gene-MA16_Dca018027	uncharacterized protein LOC110100879 [Dendrobium catenatum]
gene-MA16_Dca005243	Diaminopimelate epimerase, chloroplastic [Dendrobium catenatum]
gene-MA16_Dca007583	gamma-interferon-inducible-lysosomal thiol reductase-like [Dendrobium catenatum]
gene-MA16_Dca013957	eukaryotic translation initiation factor 4B1-like [Dendrobium catenatum]
gene-MA16_Dca017986	putative plastid-lipid-associated protein 8, chloroplastic [Dendrobium catenatum]
gene-MA16_Dca023863	hypothetical protein MA16_Dca023863 [Dendrobium catenatum]
gene-MA16_Dca011594	Transcription factor HBP-1b(c38) [Dendrobium catenatum]
gene-MA16_Dca017455	Chaperone protein dnaJ 1, mitochondrial [Dendrobium catenatum]
gene-MA16_Dca014023	replication protein A 70 kDa DNA-binding subunit B [Dendrobium catenatum]
gene-MA16_Dca010352	Retrovirus-related Pol polyprotein from transposon TNT 1-94 [Dendrobium catenatum]
gene-MA16_Dca007240	flavin-containing monooxygenase FMO GS-OX5-like isoform X1 [Dendrobium catenatum]
gene-MA16_Dca021525	hypothetical protein MA16_Dca021525 [Dendrobium catenatum]
gene-MA16_Dca010194	putative E3 ubiquitin-protein ligase UBR7 [Dendrobium catenatum]
gene-MA16_Dca027552	hypothetical protein MA16_Dca027552 [Dendrobium catenatum]
gene-MA16_Dca024114	BTB/POZ domain-containing protein POB1-like [Dendrobium catenatum]
gene-MA16_Dca018152	phospholipid-transporting ATPase 1-like [Dendrobium catenatum]
gene-MA16_Dca028558	hypothetical protein MA16_Dca028558 [Dendrobium catenatum]
gene-MA16_Dca004708	Retrovirus-related Pol polyprotein from transposon TNT 1-94 [Dendrobium catenatum]
gene-MA16_Dca014400	methionyl-tRNA formyltransferase [Dendrobium catenatum]
gene-MA16_Dca026575	mannose-specific lectin-like [Dendrobium catenatum]
gene-MA16_Dca001276	Leucine aminopeptidase 2, chloroplastic [Dendrobium catenatum]
gene-MA16_Dca014079	hypothetical protein MA16_Dca014079 [Dendrobium catenatum]
gene-MA16_Dca022332	hypothetical protein MA16_Dca022332 [Dendrobium catenatum]
gene-MA16_Dca003665	Nitrate transporter 1.3 [Dendrobium catenatum]
gene-MA16_Dca000326	calmodulin-binding protein 60 C-like [Dendrobium catenatum]
gene-MA16_Dca023056	Retrovirus-related Pol polyprotein from transposon TNT 1-94 [Dendrobium catenatum]
gene-MA16_Dca006834	hypothetical protein MA16_Dca006834 [Dendrobium catenatum]
gene-MA16_Dca006592	Retrovirus-related Pol polyprotein from transposon TNT 1-94 [Dendrobium catenatum]
gene-MA16_Dca012833	Retrovirus-related Pol polyprotein from transposon TNT 1-94 [Dendrobium catenatum]

gene-MA16_Dca015409	MLO-like protein 1 [Dendrobium catenatum]
gene-MA16_Dca021332	hypothetical protein MA16_Dca021332 [Dendrobium catenatum]
gene-MA16_Dca010130	cytochrome P450 704C1-like isoform X1 [Dendrobium catenatum]
gene-MA16_Dca002326	uncharacterized protein LOC110110061 [Dendrobium catenatum]
gene-MA16_Dca000984	Transcription factor GAMYB [Dendrobium catenatum]
gene-MA16_Dca005204	hypothetical protein MA16_Dca005204 [Dendrobium catenatum]
gene-MA16_Dca007315	hypothetical protein MA16_Dca007315 [Dendrobium catenatum]
gene-MA16_Dca018411	hypothetical protein MA16_Dca018411 [Dendrobium catenatum]
gene-MA16_Dca019205	F-box/kelch-repeat protein At5g42350-like [Dendrobium catenatum]
gene-MA16_Dca022051	beta-amylase 8 isoform X1 [Dendrobium catenatum]
gene-MA16_Dca024152	uncharacterized protein LOC110112086 [Dendrobium catenatum]
gene-MA16_Dca011647	hypothetical protein MA16_Dca011647 [Dendrobium catenatum]
gene-MA16_Dca002803	uncharacterized protein LOC110097748 [Dendrobium catenatum]
gene-MA16_Dca010411	putative UPF0496 protein 2 [Dendrobium catenatum]
gene-MA16_Dca018115	BTB/POZ domain-containing protein NPY2 [Dendrobium catenatum]
gene-MA16_Dca005914	hypothetical protein MA16_Dca005914 [Dendrobium catenatum]
gene-MA16_Dca004189	Protein FAR1-RELATED SEQUENCE 5 [Dendrobium catenatum]
gene-MA16_Dca003840	hypothetical protein MA16_Dca003840 [Dendrobium catenatum]
gene-MA16_Dca013403	putative clathrin assembly protein At5g35200 [Dendrobium catenatum]
gene-MA16_Dca021670	serine/threonine-protein kinase EDR1-like isoform X1 [Dendrobium catenatum]
gene-MA16_Dca012501	Centromere/kinetochore protein zw10 like [Dendrobium catenatum]
gene-MA16_Dca013337	floral homeotic protein APETALA 2-like isoform X1 [Dendrobium catenatum]
gene-MA16_Dca010934	Mitogen-activated protein kinase kinase kinase YODA [Dendrobium catenatum]
gene-MA16_Dca016882	hypothetical protein MA16_Dca016882 [Dendrobium catenatum]
gene-MA16_Dca019758	protein AE7-like 1 [Dendrobium catenatum]
gene-MA16_Dca013779	probable RNA helicase SDE3 [Dendrobium catenatum]
gene-MA16_Dca009269	glycylpeptide N-tetradecanoyltransferase 1-like [Dendrobium catenatum]
gene-MA16_Dca018822	MATE efflux family protein 9 [Dendrobium catenatum]
gene-MA16_Dca000122	peroxidase P7-like [Dendrobium catenatum]
gene-MA16_Dca013592	uncharacterized protein LOC110103622 [Dendrobium catenatum]

gene-MA16_Dca013460	hypothetical protein MA16_Dca013460 [Dendrobium catenatum]
gene-MA16_Dca006631	DNA helicase INO80 [Dendrobium catenatum]
gene-MA16_Dca015210	hypothetical protein MA16_Dca015210 [Dendrobium catenatum]
gene-MA16_Dca018870	hypothetical protein MA16_Dca018870 [Dendrobium catenatum]
gene-MA16_Dca012151	hypothetical protein MA16_Dca012151 [Dendrobium catenatum]
gene-MA16_Dca024232	receptor-like protein kinase HSL1 [Dendrobium catenatum]
gene-MA16_Dca021289	MATE efflux family protein 5 [Dendrobium catenatum]
gene-MA16_Dca020386	hypothetical protein MA16_Dca020386 [Dendrobium catenatum]
gene-MA16_Dca017938	uncharacterized protein LOC110108861 [Dendrobium catenatum]
gene-MA16_Dca016060	chaperone protein dnaJ 16-like [Dendrobium catenatum]
gene-MA16_Dca008999	suppressor of RPS4-RLD 1 isoform X1 [Dendrobium catenatum]
gene-MA16_Dca020663	type I inositol polyphosphate 5-phosphatase 1-like isoform X1 [Dendrobium catenatum]
gene-MA16_Dca024573	Uncharacterized protein MA16_Dca024573 [Dendrobium catenatum]
gene-MA16_Dca011783	hypothetical protein MA16_Dca011783 [Dendrobium catenatum]
gene-MA16_Dca024109	Retrovirus-related Pol polyprotein from transposon TNT 1-94 [Dendrobium catenatum]
gene-MA16_Dca015244	Laccase-14 [Dendrobium catenatum]
gene-MA16_Dca011374	putative glycosyltransferase [Dendrobium catenatum]
gene-MA16_Dca017326	Pentatricopeptide repeat-containing protein [Dendrobium catenatum]
gene-MA16_Dca025897	cycloartenol synthase [Dendrobium catenatum]
gene-MA16_Dca013465	Putative disease resistance protein RGA4 [Dendrobium catenatum]
gene-MA16_Dca003471	probable lysophospholipase BODYGUARD 3 [Dendrobium catenatum]
gene-MA16_Dca023138	Putative disease resistance RPP13-like protein 1 [Dendrobium catenatum]
gene-MA16_Dca020497	UV-stimulated scaffold protein A homolog [Dendrobium catenatum]
gene-MA16_Dca007049	transcription factor LHW-like isoform X1 [Dendrobium catenatum]
gene-MA16_Dca009502	hypothetical protein MA16_Dca009502 [Dendrobium catenatum]
gene-MA16_Dca009078	hypothetical protein MA16_Dca009078 [Dendrobium catenatum]
gene-MA16_Dca003297	cytochrome P450 71A1-like [Dendrobium catenatum]
gene-MA16_Dca004642	protein PNS1 [Dendrobium catenatum]
gene-MA16_Dca006688	hypothetical protein MA16_Dca006688 [Dendrobium catenatum]
gene-MA16_Dca024704	hypothetical protein MA16_Dca024704 [Dendrobium catenatum]

gene-MA16_Dca002484	Disease resistance protein RGA2 [Dendrobium catenatum]
gene-MA16_Dca013620	WPP domain-interacting protein 1 [Dendrobium catenatum]
gene-MA16_Dca015500	Pentatricopeptide repeat-containing protein [Dendrobium catenatum]
gene-MA16_Dca028061	Retrovirus-related Pol polyprotein from transposon TNT 1-94 [Dendrobium catenatum]
gene-MA16_Dca017878	uncharacterized protein LOC110114295 [Dendrobium catenatum]
gene-MA16_Dca011965	probable E3 ubiquitin-protein ligase RNF144A-B isoform X1 [Dendrobium catenatum]
gene-MA16_Dca013600	cullin-associated NEDD8-dissociated protein 1 [Dendrobium catenatum]
gene-MA16_Dca004102	uncharacterized protein C6G9.01c [Dendrobium catenatum]
gene-MA16_Dca000350	SCAR-like protein 2 [Dendrobium catenatum]
gene-MA16_Dca015205	zinc finger protein ZAT1-like [Dendrobium catenatum]
gene-MA16_Dca003931	uncharacterized protein LOC110110813 [Dendrobium catenatum]
gene-MA16_Dca001796	ribosomal RNA small subunit methyltransferase [Dendrobium catenatum]
gene-MA16_Dca001920	putative protein phosphatase 2C 4 [Dendrobium catenatum]
gene-MA16_Dca017029	RNA-directed DNA polymerase [Dendrobium catenatum]
gene-MA16_Dca021852	uncharacterized protein LOC110113946 [Dendrobium catenatum]
gene-MA16_Dca011516	hypothetical protein MA16_Dca011516 [Dendrobium catenatum]
gene-MA16_Dca000945	hypothetical protein MA16_Dca000945 [Dendrobium catenatum]
gene-MA16_Dca022812	protease Do-like 7 isoform X2 [Dendrobium catenatum]
gene-MA16_Dca020312	venom phosphodiesterase 2-like [Dendrobium catenatum]
gene-MA16_Dca026778	Nodulation-signaling pathway 2 protein [Dendrobium catenatum]
gene-MA16_Dca001352	receptor-like kinase TMK4 [Dendrobium catenatum]
gene-MA16_Dca002782	dof zinc finger protein DOF2.1-like [Dendrobium catenatum]
gene-MA16_Dca025304	Premnaspirodiene oxygenase [Dendrobium catenatum]
gene-MA16_Dca003942	hypothetical protein MA16_Dca003942 [Dendrobium catenatum]
gene-MA16_Dca026216	Lecithin-cholesterol acyltransferase-like 1 [Dendrobium catenatum]
gene-MA16_Dca009483	chlorophyll a-b binding protein CP29.1, chloroplastic-like [Dendrobium catenatum]
gene-MA16_Dca007629	protein NETWORKED 4B-like [Dendrobium catenatum]
gene-MA16_Dca015676	Retrovirus-related Pol polyprotein from transposon TNT 1-94 [Dendrobium catenatum]
gene-MA16_Dca014991	Protein MOR1 [Dendrobium catenatum]
gene-MA16_Dca015294	hypothetical protein MA16_Dca015294 [Dendrobium catenatum]

gene-MA16_Dca009670	60S ribosomal protein L15-1 [Dendrobium catenatum]
gene-MA16_Dca028455	hypothetical protein MA16_Dca028455 [Dendrobium catenatum]
gene-MA16_Dca022444	hypothetical protein MA16_Dca022444 [Dendrobium catenatum]
gene-MA16_Dca027734	DNA-directed RNA polymerase II subunit RPB7 [Dendrobium catenatum]
gene-MA16_Dca025030	RAN GTPase-activating protein 1 [Dendrobium catenatum]
gene-MA16_Dca028065	transcription factor GAMYB-like [Dendrobium catenatum]
gene-MA16_Dca018325	vacuolar protein sorting-associated protein 51 homolog [Dendrobium catenatum]
gene-MA16_Dca012188	Formin-like protein 12 [Dendrobium catenatum]
gene-MA16_Dca014524	hypothetical protein MA16_Dca014524 [Dendrobium catenatum]
gene-MA16_Dca003896	hypothetical protein MA16_Dca003896 [Dendrobium catenatum]
gene-MA16_Dca024692	uncharacterized protein LOC110098342 [Dendrobium catenatum]
gene-MA16_Dca015722	pentatricopeptide repeat-containing protein At5g48910-like [Dendrobium catenatum]
gene-MA16_Dca000100	Cellulose synthase A catalytic subunit 9 [UDP-forming] [Dendrobium catenatum]
gene-MA16_Dca015255	F-box only protein 6 isoform X3 [Dendrobium catenatum]
gene-MA16_Dca012928	squamosa promoter-binding-like protein 10 [Dendrobium catenatum]
gene-MA16_Dca013019	hypothetical protein MA16_Dca013019 [Dendrobium catenatum]
gene-MA16_Dca014423	uncharacterized protein LOC110116116 [Dendrobium catenatum]
gene-MA16_Dca003302	pentatricopeptide repeat-containing protein At3g24000, mitochondrial-like [Dendrobium catenatum]
gene-MA16_Dca026212	amino acid permease 3-like [Dendrobium catenatum]
gene-MA16_Dca008938	transcriptional regulator ATRX homolog [Dendrobium catenatum]
gene-MA16_Dca018091	Putative disease resistance protein RGA1 [Dendrobium catenatum]
gene-MA16_Dca003428	RING-H2 finger protein ATL1 [Dendrobium catenatum]
gene-MA16_Dca015910	target of Myb protein 1-like [Dendrobium catenatum]
gene-MA16_Dca013529	transcription factor GTE9-like [Dendrobium catenatum]
gene-MA16_Dca019322	probable polygalacturonase [Dendrobium catenatum]
gene-MA16_Dca000459	21 kDa protein [Dendrobium catenatum]
gene-MA16_Dca014626	pentatricopeptide repeat-containing protein MRL1, chloroplastic [Dendrobium catenatum]
gene-MA16_Dca023064	cytochrome b6-f complex iron-sulfur subunit, chloroplastic [Dendrobium catenatum]
gene-MA16_Dca013128	hypothetical protein MA16_Dca013128 [Dendrobium catenatum]
gene-MA16_Dca007967	hypothetical protein MA16_Dca007967 [Dendrobium catenatum]

gene-MA16_Dca017663	Disease resistance protein RPM1 [Dendrobium catenatum]
gene-MA16_Dca020435	pectinesterase-like [Dendrobium catenatum]
gene-MA16_Dca011409	uncharacterized protein LOC110110224 [Dendrobium catenatum]
gene-MA16_Dca019463	mitochondrial phosphate carrier protein 3, mitochondrial-like [Dendrobium catenatum]
gene-MA16_Dca007257	Retrovirus-related Pol polyprotein from transposon TNT 1-94 [Dendrobium catenatum]
gene-MA16_Dca000889	uncharacterized protein LOC110096393 [Dendrobium catenatum]
gene-MA16_Dca010381	probable metal-nicotianamine transporter YSL9 [Dendrobium catenatum]
gene-MA16_Dca020207	MATE efflux family protein DTX1 [Dendrobium catenatum]
gene-MA16_Dca014822	Ribulose bisphosphate carboxylase large chain [Dendrobium catenatum]
gene-MA16_Dca016750	PHD finger protein ALFIN-LIKE 3 [Dendrobium catenatum]
gene-MA16_Dca004126	Retrovirus-related Pol polyprotein from transposon TNT 1-94 [Dendrobium catenatum]
gene-MA16_Dca021924	uncharacterized protein LOC110116047 [Dendrobium catenatum]
gene-MA16_Dca014378	ABC transporter G family member 42-like [Dendrobium catenatum]
gene-MA16_Dca017403	hypothetical protein MA16_Dca017403 [Dendrobium catenatum]
gene-MA16_Dca025118	hypothetical protein MA16_Dca025118 [Dendrobium catenatum]
gene-MA16_Dca023553	squamosa promoter-binding-like protein 1 [Dendrobium catenatum]
gene-MA16_Dca004173	Putative disease resistance protein RGA4 [Dendrobium catenatum]
gene-MA16_Dca019925	Retrovirus-related Pol polyprotein from transposon TNT 1-94 [Dendrobium catenatum]
gene-MA16_Dca011525	NDR1/HIN1-like protein 13 [Dendrobium catenatum]
gene-MA16_Dca007347	E3 ubiquitin-protein ligase [Dendrobium catenatum]
gene-MA16_Dca019488	plant-specific TFIIB-related protein PTF2 [Dendrobium catenatum]
gene-MA16_Dca012831	RNA-directed DNA polymerase [Dendrobium catenatum]
gene-MA16_Dca017354	putative aldo-keto reductase 2 [Dendrobium catenatum]
gene-MA16_Dca009169	transmembrane protein 56-like isoform X1 [Dendrobium catenatum]
gene-MA16_Dca026606	uncharacterized protein LOC110101272 [Dendrobium catenatum]
gene-MA16_Dca009868	dof zinc finger protein DOF1.8-like [Dendrobium catenatum]
gene-MA16_Dca025367	HBS1-like protein isoform X2 [Dendrobium catenatum]
gene-MA16_Dca012698	putative receptor-like protein kinase [Dendrobium catenatum]
gene-MA16_Dca003260	agmatine coumaroyltransferase-1-like [Dendrobium catenatum]
gene-MA16_Dca013202	peroxisome biogenesis protein 19-2-like [Dendrobium catenatum]

gene-MA16_Dca012286	Proline-rich receptor-like protein kinase PERK13 [Dendrobium catenatum]
gene-MA16_Dca004586	uncharacterized protein LOC110093827 isoform X2 [Dendrobium catenatum]
gene-MA16_Dca025576	hypothetical protein MA16_Dca025576 [Dendrobium catenatum]
gene-MA16_Dca024053	50S ribosomal protein L14, chloroplastic [Dendrobium catenatum]
gene-MA16_Dca021072	hypothetical protein MA16_Dca021072 [Dendrobium catenatum]
gene-MA16_Dca019230	fasciclin-like arabinogalactan protein 9 [Dendrobium catenatum]
gene-MA16_Dca020910	hypothetical protein MA16_Dca020910 [Dendrobium catenatum]
gene-MA16_Dca004251	putative NOT transcription complex subunit VIP2 [Dendrobium catenatum]
gene-MA16_Dca022317	hypothetical protein MA16_Dca022317 [Dendrobium catenatum]
gene-MA16_Dca014554	hypothetical protein MA16_Dca014554 [Dendrobium catenatum]
gene-MA16_Dca020331	metal tolerance protein 1-like [Dendrobium catenatum]
gene-MA16_Dca000613	hypothetical protein MA16_Dca000613 [Dendrobium catenatum]
gene-MA16_Dca002242	putative helicase MAGATAMA 3 [Dendrobium catenatum]
gene-MA16_Dca021867	uncharacterized protein LOC110113937 [Dendrobium catenatum]
gene-MA16_Dca019194	putative Ufm1-specific protease [Dendrobium catenatum]
gene-MA16_Dca001220	Methyltransferase-like protein 2 [Dendrobium catenatum]
gene-MA16_Dca013655	hypothetical protein MA16_Dca013655 [Dendrobium catenatum]
gene-MA16_Dca003285	ATP sulfurylase 1, chloroplastic-like [Dendrobium catenatum]
gene-MA16_Dca009484	protein trichome birefringence-like 19 [Dendrobium catenatum]
gene-MA16_Dca002926	Putative ribonuclease H protein [Dendrobium catenatum]
gene-MA16_Dca008031	hypothetical protein MA16_Dca008031 [Dendrobium catenatum]
gene-MA16_Dca002476	hypothetical protein MA16_Dca002476 [Dendrobium catenatum]
gene-MA16_Dca026695	125 kDa kinesin-related protein [Dendrobium catenatum]
gene-MA16_Dca010444	Protein LONGIFOLIA 1 [Dendrobium catenatum]
gene-MA16_Dca003498	hypothetical protein MA16_Dca003498 [Dendrobium catenatum]
gene-MA16_Dca009093	transcriptional repressor ILP1 [Dendrobium catenatum]
gene-MA16_Dca011564	hypothetical protein MA16_Dca011564 [Dendrobium catenatum]
gene-MA16_Dca026803	MADS-box transcription factor 22 [Dendrobium catenatum]
gene-MA16_Dca011035	Transcription factor GAMYB [Dendrobium catenatum]
gene-MA16_Dca019334	Retrovirus-related Pol polyprotein from transposon TNT 1-94 [Dendrobium catenatum]

gene-MA16_Dca005346	Uncharacterized protein MA16_Dca005346 [Dendrobium catenatum]
gene-MA16_Dca010163	Syntaxin-112 [Dendrobium catenatum]
gene-MA16_Dca012062	Protein UNUSUAL FLORAL ORGANS [Dendrobium catenatum]
gene-MA16_Dca012896	Receptor-like protein kinase [Dendrobium catenatum]
gene-MA16_Dca005436	polyadenylation and cleavage factor homolog 4 isoform X2 [Dendrobium catenatum]
gene-MA16_Dca021773	hypothetical protein MA16_Dca021773 [Dendrobium catenatum]
gene-MA16_Dca010123	pentatricopeptide repeat-containing protein At2g36980, mitochondrial [Dendrobium catenatum]
gene-MA16_Dca003714	hypothetical protein MA16_Dca003714 [Dendrobium catenatum]
gene-MA16_Dca022381	hypothetical protein MA16_Dca022381 [Dendrobium catenatum]
gene-MA16_Dca003078	TPR repeat-containing thioredoxin TTL1 [Dendrobium catenatum]
gene-MA16_Dca008216	hypothetical protein MA16_Dca008216 [Dendrobium catenatum]
gene-MA16_Dca013867	UPF0481 protein [Dendrobium catenatum]
gene-MA16_Dca000376	Histone H1.2 [Dendrobium catenatum]
gene-MA16_Dca023769	hypothetical protein MA16_Dca023769 [Dendrobium catenatum]
gene-MA16_Dca023414	putative pentatricopeptide repeat-containing protein At5g08490 [Dendrobium catenatum]
gene-MA16_Dca011666	probable F-box protein At2g36090 [Dendrobium catenatum]
gene-MA16_Dca012030	DEAD-box ATP-dependent RNA helicase ISE2, chloroplastic [Dendrobium catenatum]
gene-MA16_Dca011565	uncharacterized protein LOC110093805 [Dendrobium catenatum]
gene-MA16_Dca004386	hypothetical protein MA16_Dca004386 [Dendrobium catenatum]
gene-MA16_Dca008729	protein DWARF AND LOW-TILLERING-like [Dendrobium catenatum]
gene-MA16_Dca005154	sodium-coupled neutral amino acid transporter 2-like isoform X1 [Dendrobium catenatum]
gene-MA16_Dca014326	pathogenesis-related genes transcriptional activator PTI6-like [Dendrobium catenatum]
gene-MA16_Dca006457	hypothetical protein MA16_Dca006457 [Dendrobium catenatum]
gene-MA16_Dca000619	uncharacterized protein LOC110115684 [Dendrobium catenatum]
gene-MA16_Dca000981	uncharacterized protein LOC110103751 [Dendrobium catenatum]
gene-MA16_Dca016865	Valine--tRNA ligase [Dendrobium catenatum]
gene-MA16_Dca018659	hypothetical protein MA16_Dca018659 [Dendrobium catenatum]
gene-MA16_Dca027577	hypothetical protein MA16_Dca027577 [Dendrobium catenatum]
gene-MA16_Dca021684	Pentatricopeptide repeat-containing protein [Dendrobium catenatum]
gene-MA16_Dca010749	mRNA-capping enzyme [Dendrobium catenatum]

gene-MA16_Dca001201	hypothetical protein MA16_Dca001201 [Dendrobium catenatum]
gene-MA16_Dca011895	NDR1/HIN1-like protein 2 [Dendrobium catenatum]
gene-MA16_Dca016967	hypothetical protein MA16_Dca016967 [Dendrobium catenatum]
