# Supplementary material for: RNA sequencing-based exploration of the effects of far-red light on microRNAs involved in the shade-avoidance response of D. officinale
Source: PeerJ. 2023 Mar 20;11:e15001. doi: 10.7717/peerj.15001 (PMC10035421; doi:10.7717/peerj.15001)
Supplement: Table S10 [file peerj-11-15001-s010.docx]

| Table S10 Folic acid contents of leaves in *D. officinale* under different light treatments | | | | | | | | |  |
| --- | --- | --- | --- | --- | --- | --- | --- | --- | --- |
| Light treatments | Light intensity (µmol·m^-2^·s^-1^) | Photoperiod (h) | Folic acid  contents 1  (pg·ml ^-1^ FW) | Folic acid  contents 2  (pg·ml ^-1^ FW) | Folic acid  contents 3  (pg·ml ^-1^ FW) | Average Folic acid  contents  (pg·ml ^-1^ FW) | Standard deviation | Duncan (5%) | Duncan (1%) |
| CK | 200 | 12 | 46.39 | 27.88 | 16.80 | 30.35 | 12.21 | c | C |
| FR2 | 200 | 12 | 28.49 | 41.92 | 36.56 | 35.66 | 5.52 | b | B |
| FR8 | 200 | 12 | 84.38 | 95.02 | 77.44 | 85.61 | 7.23 | a | A |
