# Supplementary material for: RNA sequencing-based exploration of the effects of far-red light on microRNAs involved in the shade-avoidance response of D. officinale
Source: PeerJ. 2023 Mar 20;11:e15001. doi: 10.7717/peerj.15001 (PMC10035421; doi:10.7717/peerj.15001)
Supplement: Table S11 [file peerj-11-15001-s011.docx]

| Table S11 Folic acid contents of stems in *D. officinale* under different light treatments | | | | | | | | |  |
| --- | --- | --- | --- | --- | --- | --- | --- | --- | --- |
| Light treatments | Light intensity (µmol·m^-2^·s^-1^) | Photoperiod (h) | Folic acid  contents 1  (pg·ml ^-1^ FW) | Folic acid  contents 2  (pg·ml ^-1^ FW) | Folic acid  contents 3  (pg·ml ^-1^ FW) | Average Folic acid  contents  (pg·ml ^-1^ FW) | Standard deviation | Duncan (5%) | Duncan (1%) |
| CK | 200 | 12 | 31.72 | 28.83 | 25.31 | 28.62 | 2.62 | c | C |
| FR2 | 200 | 12 | 68.46 | 60.16 | 69.22 | 65.95 | 4.10 | b | B |
| FR8 | 200 | 12 | 103.60 | 77.44 | 84.98 | 88.67 | 10.99 | a | A |
