# Supplementary material for: RNA sequencing-based exploration of the effects of far-red light on microRNAs involved in the shade-avoidance response of D. officinale
Source: PeerJ. 2023 Mar 20;11:e15001. doi: 10.7717/peerj.15001 (PMC10035421; doi:10.7717/peerj.15001)
Supplement: Table S12 [file peerj-11-15001-s012.docx]

| Table S12 Ascorbic acid contents of leaves in *D. officinale* under different light treatments | | | | | | | | |  |
| --- | --- | --- | --- | --- | --- | --- | --- | --- | --- |
| Light treatments | Light intensity (µmol·m^-2^·s^-1^) | Photoperiod (h) | Ascorbic acid contents 1  (μg·g ^-1^FW) | Ascorbic acid contents 2  (μg·g ^-1^FW) | Ascorbic acid contents 3  (μg·g ^-1^FW) | Average Ascorbic acid  contents  (μg·g ^-1^FW) | Standard deviation | Duncan (5%) | Duncan (1%) |
| CK | 200 | 12 | 9.60 | 9.26 | 9.77 | 9.54 | 0.21 | c | C |
| FR2 | 200 | 12 | 13.89 | 13.54 | 14.57 | 14.00 | 0.43 | b | B |
| FR8 | 200 | 12 | 19.54 | 19.20 | 18.86 | 19.20 | 0.28 | a | A |
