# Supplementary material for: RNA sequencing-based exploration of the effects of far-red light on microRNAs involved in the shade-avoidance response of D. officinale
Source: PeerJ. 2023 Mar 20;11:e15001. doi: 10.7717/peerj.15001 (PMC10035421; doi:10.7717/peerj.15001)
Supplement: Table S13 [file peerj-11-15001-s013.docx]

| Table S13 Ascorbic acid contents of stems in *D. officinale* under different light treatments | | | | | | | | |  |
| --- | --- | --- | --- | --- | --- | --- | --- | --- | --- |
| Light treatments | Light intensity (µmol·m^-2^·s^-1^) | Photoperiod (h) | Ascorbic acid  contents 1  (μg·g ^-1^FW) | Ascorbic acid  contents 2  (μg·g ^-1^FW) | Ascorbic acid  contents 3  (μg·g ^-1^FW) | Average Ascorbic acid  contents  (μg·g ^-1^FW) | Standard deviation | Duncan (5%) | Duncan (1%) |
| CK | 200 | 12 | 6.86 | 7.20 | 7.71 | 7.26 | 0.35 | c | C |
| FR2 | 200 | 12 | 9.94 | 9.43 | 8.91 | 9.43 | 0.42 | b | B |
| FR8 | 200 | 12 | 13.71 | 13.89 | 14.74 | 14.11 | 0.45 | a | A |
