# Supplementary material for: RNA sequencing-based exploration of the effects of far-red light on microRNAs involved in the shade-avoidance response of D. officinale
Source: PeerJ. 2023 Mar 20;11:e15001. doi: 10.7717/peerj.15001 (PMC10035421; doi:10.7717/peerj.15001)
Supplement: Table S14 [file peerj-11-15001-s014.pdf]

Table S14 The Ct mean of DE miRNAs and genes in *D. officinale* under different light treatments

Light treatment	novel_miR_53	miR395b	miR399c_5p	miR399t_3p	novel_miR_36	novel_miR_483	novel_miR_390	novel_miR_159	novel_miR_178	novel_miR_405	5.8 s rRNA
	Ct Mean	Ct Mean	Ct Mean	Ct Mean	Ct Mean	Ct Mean	Ct Mean	Ct Mean	Ct Mean	Ct Mean	Ct Mean
CK-1	21.23	20.48	19.72	20.03	21.18	22.96	19.45	18.45	22.75	21.19	21.42
CK-2	21.46	20.65	19.77	20.04	21.30	22.97	19.57	18.67	22.57	21.23	21.43
CK-3	21.27	20.55	19.80	20.18	21.21	22.76	19.66	18.66	22.66	21.3	21.56
FR2-1	22.83	21.42	18.07	19.54	22.96	20.33	24.41	20.21	22.21	20.12	21.03
FR2-2	22.79	21.52	18.39	19.48	22.68	20.48	24.40	20.43	22.36	20.32	21.02
FR2-3	22.37	21.47	18.36	19.51	22.89	20.31	24.61	20.45	22.35	20.23	21.11
FR8-1	22.50	21.98	16.32	18.07	20.32	18.12	23.21	21.11	19.11	17.57	20.93
FR8-2	22.48	21.80	16.37	18.04	20.27	18.23	23.34	21.34	19.16	17.67	20.97
FR8-3	22.29	21.97	16.16	18.09	20.06	18.35	23.38	21.38	19.13	17.65	20.98

Light treatment	*THF2*	*ASA1*	*GGP1*	*CYP86B1*	*YUCCA2*	*HKT11*	*DCL2a*	*PHYA*	*PIF3*	*PIF4*	*SPA1*	*Actin*
	Ct Mean	Ct Mean	Ct Mean	Ct Mean	Ct Mean	Ct Mean	Ct Mean	Ct Mean	Ct Mean	Ct Mean	Ct Mean	Ct Mean
CK-1	29.06	30.32	26.45	28.06	27.80	27.56	28.46	33.42	26.80	30.83	28.41	25.50
CK-2	28.98	30.46	26.54	28.18	27.09	27.60	28.43	33.28	26.76	30.58	28.30	25.63
CK-3	28.92	30.52	26.35	28.17	27.04	27.66	28.37	33.30	26.67	30.79	28.31	25.60
FR2-1	25.02	30.54	26.33	30.21	24.87	24.42	27.61	33.07	24.25	28.45	26.98	25.03
FR2-2	25.07	30.12	26.43	30.32	24.82	24.35	27.52	33.18	24.28	28.53	26.81	25.01
FR2-3	25.10	30.26	26.32	30.18	24.63	24.57	27.66	32.87	24.26	28.58	26.80	25.00
FR8-1	25.52	26.07	25.11	26.13	30.11	23.08	25.67	29.86	27.63	26.17	25.72	26.63
FR8-2	25.43	26.18	25.32	26.08	30.23	23.16	25.53	29.84	27.78	26.25	25.83	26.53
FR8-3	25.51	26.12	25.23	26.10	30.12	23.20	25.51	30.16	27.73	26.33	25.65	26.32
