# Supplementary material for: RNA sequencing-based exploration of the effects of far-red light on microRNAs involved in the shade-avoidance response of D. officinale
Source: PeerJ. 2023 Mar 20;11:e15001. doi: 10.7717/peerj.15001 (PMC10035421; doi:10.7717/peerj.15001)
Supplement: Table S15 [file peerj-11-15001-s015.pdf]

Table S15 The relative expression of DE miRNAs and genes in *D. officinale* under different light treatments

Light treatment	novel_miR_53		miR395b		miR399c_5p		miR399t_3p		novel_miR_36		novel_miR_483		novel_miR_390		novel_miR_159		novel_miR_178		novel_miR_405	
	Relative expression	SD	Relative expressio	SD	Relative expression	SD	Relative expressio	SD	Relative expression	SD	Relative expression	SD	Relative expressio	SD	Relative expression	SD	Relative expressio	SD	Relative expression	SD
CK	0.93	0.10	124.30	9.86	3.82	0.13	2.60	0.01	3.74	0.28	0.50	0.07	15.61	0.59	1.98	0.14	0.33	0.03	1.10	0.02
FR2	0.26	0.06	54.46	2.83	15.82	1.83	6.04	0.25	1.60	0.15	3.13	0.29	1.08	0.05	1.11	0.09	0.84	0.05	2.63	0.20
FR8	0.33	0.03	3.28	0.26	19.89	1.75	6.43	0.16	6.35	0.41	3.49	0.22	0.84	0.04	0.37	0.03	0.92	0.01	3.71	0.08

Light treatment	*THF*2		*ASA* 1		*GGP*1		*CYP86B1*		*YUCCA* 2		*HKT* 11		*DCL* 2*a*		*PHYA*		*PIF*3		*PIF*4		*SPA* 1	
	Relative expression	SD	Relative expressio	SD	Relative expression	SD	Relative expressio	SD	Relative expression	SD	Relative expression	SD	Relative expressio	SD	Relative expression	SD	Relative expressio	SD	Relative expression	SD	Relative expression	SD
CK	1.95	0.17	0.49	0.02	7.25	0.53	9.16	0.09	0.44	0.13	35.19	1.36	0.07	0.00	0.25	0.02	0.63	0.05	4.05	0.56	0.07	0.01
FR2	8.85	0.32	1.01	0.14	7.86	0.35	5.93	0.31	1.03	0.09	152.37	12.14	0.14	0.01	0.85	0.09	1.46	0.02	9.04	0.49	0.23	0.01
FR8	2.87	0.33	1.82	0.23	19.29	3.05	16.72	0.68	0.04	0.01	553.20	81.59	1.10	0.07	1.17	0.25	0.24	0.03	64.47	5.36	0.99	0.09
